# Integrated metabolomic and transcriptomic profiling elucidates the tissue-specific biosynthesis and regulation of flavonoids in *Machilus nanmu*

**DOI:** 10.3389/fpls.2025.1731446

**Published:** 2026-01-08

**Authors:** Xiao Zhang, Changying Xia, Huan Zhang, Wenqiao Li, Zhe Zhang, Nana Long, Renxiu Yao, Jian Li, Hongping Deng

**Affiliations:** School of Life Sciences, Southwest University, Chongqing, China

**Keywords:** *Machilus nanmu*, metabolomics, transcriptome, flavonoids, biosynthetic pathway, transcription factors

## Abstract

**Introduction:**

*Machilus nanmu* is a significant arborescent species of the genus *Machilus* (Lauraceae), exhibiting considerable potential for applications in industrial materials and healthcare. However, systematic investigations into its flavonoid metabolites and associated biosynthetic mechanisms remain limited, which significantly hinders the efficient exploitation and sustainable utilization of this species.

**Methods:**

This multi-omics study revealed the specific accumulation pattern of flavonoids in the tissues of *M. nanmu* and pinpointed key structural and regulatory genes underlying their biosynthesis by integrating widely targeted metabolomics and transcriptomics data from roots, stems, and leaves.

**Results:**

A total of 425 flavonoid compounds and 35,671 differentially expressed genes were detected. Further screening revealed 41 structural genes encoding 19 key enzymes (including PAL, CHS, FLS, UGTs, etc.), among which two UGTs (Cluster-69292 and Cluster-71935) were subcellularly localized to the cytoplasm. Furthermore, the weighted gene co-expression network analysis (WGCNA) revealed four key modules exhibiting strong correlations with flavonoid content. From these modules, four core transcription factors (TFs) from the MYB and bHLH families were identified as putative regulators of flavonoid biosynthesis.

**Discussion:**

Our findings offer the first comprehensive model of tissue-specific flavonoid accumulation in *M. nanmu*, enabling the dissection of its transcriptional machinery and advancing strategies for its genetic improvement and resource exploitation.

## 1 Introduction

*Machilus nanmu* is an arboreal species within the genus *Machilus* (Lauraceae) (Flora of China Editorial Committee, 2018). It is recognized for its rich profile of bioactive metabolites, which contribute to its efficacy in alleviating conditions such as dermatitis, edema, and diarrhea. Polysaccharides derived from its leaves have demonstrated notable antioxidant and antitumor activities (Zhao et al., 2011). The genus *Machilus* comprises a diverse array of species, many of which are valued for their high-quality timber and represent significant economic forest resources in southern China. These species exhibit broad application potential in industrial wood, landscaping, medicine, spice production, chemical engineering, and cosmetics (Xu et al., 2016; Thin and Thinh, 2024). Several species within the genus *Machilus* are also employed in traditional Chinese medicine for their anti-infective, anti-inflammatory, antimicrobial, and analgesic properties (Jiangsu New Medical College, 1977; Liu et al., 2012; Ma et al., 2009; Cheng et al., 2013). Despite its extensive traditional uses and industrial potential, the chemical constituents of *M. nanmu* remain systematically uncharacterized, which hinders the efficient exploitation and sustainable development of this resource. Therefore, a comprehensive investigation into its metabolome is of considerable scientific and practical importance. Recent studies have demonstrated that widely targeted metabolomics is characterized by high precision and broad coverage of metabolites. The integration of this method with high-throughput transcriptomics has been widely employed to investigate genes and metabolites, enabling a comprehensive understanding of biosynthetic pathways (Contrepois et al., 2020). The combined application of these approaches to *M. nanmu* is expected to provide a crucial explanation for the biosynthesis and regulation of its metabolites, thereby facilitating the sustainable utilization and genetic improvement of this valuable plant resource.

Plants of the genus *Machilus* contain flavonoids, lignans, terpenoids, alkaloids and so on (Yu et al., 2000; Gan et al., 2011; Lin et al., 2015; Xie et al., 2022). Flavonoids constitute an essential class of secondary metabolites and their structures can be classified as flavones, flavonols, isoflavones, anthocyanins, flavanols, flavanones, and chalcones (Shen et al., 2022). In humans, flavonoids have the functions of free radical scavengers, antimicrobial agents, and antioxidants (Kusano et al., 2011; Crozier et al., 2009). For instance, Apigenin (flavones) inhibits tumor angiogenesis by suppressing ARHGEF1-mediated microvesicle biogenesis (Zhang et al., 2024). It also ameliorates hyperuricemic nephropathy via inhibition of URAT1 and GLUT9 (Li et al., 2021). Myricetin (flavonols) exhibits cytotoxic effects on SNU-790 HPTC cells (Ha et al., 2017). Isoflavones are found predominantly in leguminous plants. Daidzein and genistein are known for their phytoestrogenic effects, which help alleviate menopausal symptoms and prevent osteoporosis (Tousen et al., 2013; Zhou et al., 2025). Serving as precursors in flavonoid biosynthesis, chalcones display antibacterial, antitumor, and antiviral activities (Ouyang et al., 2021), and their derivatives have shown potential in the treatment of Alzheimer’s disease (Haas et al., 2024). As an important tree species of the genus *Machilus* (Lauraceae), *M. nanmu* is widely distributed with abundant resources. However, current research on the species, content, pharmaceutical activities, and biosynthetic mechanisms of flavonoids in *M. nanmu* remains largely scarce, which has severely hindered the development and utilization of its pharmaceutical value. Therefore, conducting studies related to flavonoids in *M. nanmu* to fill these existing research gaps is of great significance for exploring its pharmaceutical potential and promoting innovations in natural medicinal resources.

Flavonoid compounds play a crucial role in the growth, development and stress responses of plants (Zhang et al., 2017). The enzymes that regulate the biosynthesis of flavonoids are also of great significance. For instance, overexpression of *BcPAL1* and *BcPAL2* in non-heading Chinese cabbage enhanced thermotolerance, accompanied by increased phenylalanine ammonia-lyase (PAL) activity (Gao et al., 2025). Under light stress conditions, *CsCHS* in tea plants can regulate the biosynthesis process of flavonoids (Li et al., 2024). *bHLHL74* negatively regulates the flavonoid biosynthesis process in rose by repressing *CHS1* expression under salt stress (Ren et al., 2024). In *Ginkgo biloba*, the antisense *LncNAT11* negatively regulates flavonol biosynthesis and reactive oxygen species (ROS) accumulation under salinity by suppressing *GbMYB11* expression and subsequently downregulating *GbF3’H* and *GbFLS* (Liu et al., 2025). Additionally, UDP-glycosyltransferases influence grain size and abiotic stress tolerance in rice by redirecting metabolic flux (Dong et al., 2020). *CHS* (*TT4*) and *CHI* (*TT5*) are central to flavonoid-mediated UV-B protection in *Arabidopsis thaliana* (Kusano et al., 2011). Collectively, these genes form a critical chemical defense network that enables plants to adapt to various environmental challenges. Notably, the expression of these structural genes is not autonomous but is precisely orchestrated by a sophisticated transcriptional regulatory network. For instance, the MBW complex is an important regulatory factor in the biosynthesis of anthocyanins in numerous plant species. It is composed of MYB, bHLH and WD40 proteins (Xie et al., 2016; Liu et al., 2021; Chen et al., 2023). The *MtMYB134* coordinates flavonol biosynthesis in *Medicago truncatula* (Naik et al., 2021). The *MdMYB305–MdbHLH33–MdMYB10* complex modulates anthocyanin homeostasis in apple by binding to *MdF3H*, *MdDFR*, and *MdUFGT* (Zhang et al., 2023). These findings illustrate how transcriptional regulators integrate stress signals with metabolic responses to regulate flavonoid biosynthesis. However, to date, research on the coordinated regulation of growth, development, and stress resistance by flavonoids and TFs in *M. nanmu* remains largely unexplored. The underlying regulatory pathways, key genes, and interaction modes involved in this process are still unclear. Filling this research gap will not only improve the molecular theoretical system governing the regulation of flavonoids and TFs in Lauraceae plants, but also provide a scientific basis for stress-resistant breeding and efficient resource utilization of *M. nanmu*. Furthermore, it holds great significance for advancing research on plant molecular regulatory mechanisms and promoting innovations in the forestry industry.

Therefore, we employed transcriptomics and metabolomics in this study to investigate the biosynthetic pathways in various tissues of *M. nanmu*. To clarify the mechanisms of the accumulation of tissue-specific flavonoids, we identified the genes that encode enzymes and TFs involved in flavonoid biosynthesis. Furthermore, qRT-PCR was conducted to validate the transcriptomic level. Our results systematically characterize the composition and spatial distribution of flavonoid metabolites in *M. nanmu*, revealing putative associations between critical metabolites and regulatory genes. These findings establish a molecular framework for exploring the biosynthesis and accumulation process of flavonoids in this species, thereby supporting future research into their regulatory mechanisms and biological functions.

## 2 Materials and methods

### 2.1 Materials and chemicals

In the present study, roots (R), stems (S) and leaves (L) were collected from three-year-old healthy *M. nanmu* plants growing naturally on Jinyun Mountain, Beibei, Chongqing, China. Specifically, roots (3–5 cm from the apices of taproots and lateral roots), stems (3–5 cm from the shoot apex), and the 1st to 3rd fully expanded leaves from the top of the plants were immediately wrapped in aluminum foil, labeled, and quickly placed in liquid nitrogen for subsequent metabolomic and transcriptomic analyses. Three individual trees were combined into a group, with three groups. HPLC reagents: formic acid, Aladdin Reagent Co., Ltd. (Shanghai, China); acetonitrile and methanol, Merck Group (Darmstadt, Germany).

### 2.2 Metabolite extraction, qualitative and quantitative, and screening analysis

*M. nanmu* samples were vacuum freeze-dried, then ground and crushed. 50 mg of the powder was weighed and extracted with 1200 μL of 70% methanol-water solution (containing an internal standard) for subsequent UPLC-MS/MS analysis. By comparing the MS/MS spectra from the experiments with the self-constructed Metware database (MWDB), metabolites were identified. Quantitative analysis was performed using the multiple reaction monitoring (MRM) method on a triple quadrupole mass spectrometer. Unsupervised PCA was conducted using the prcomp function in R. Sample and metabolite clustering patterns were visualized through HCA heatmaps. Inter-sample correlations were calculated using PCC and displayed in heatmaps. In the comparison groups, the differentially expressed metabolites were identified based on VIP > 1 and |Log_2_FC| ≥ 1.0.

### 2.3 RNA extraction, transcriptome sequencing and analysis

Total RNA was extracted from each tissue of *M. nanmu* tissues using a plant RNA extraction kit (Hua Yueyang, Beijing, China). Following a rigorous assessment of RNA quality, sequencing was performed on the Illumina HiSeq platform. The resultant raw reads were processed with fastp to obtain high-quality clean data (Chen et al., 2018). Then, Trinity software was used for *de novo* transcriptome assembly (Grabherr et al., 2011). The assembly results were refined by clustering and removing redundancies of the transcripts using Corset (Davidson and Oshlack, 2014). Putative coding regions (CDS) within these transcripts were identified using TransDecoder, which also facilitated the deduction of corresponding amino acid sequences. The assembled transcript sequences were compared with the KEGG, NR, Swiss-Prot, GO, COG/KOG, and Trembl databases using DIAMON (Buchfink et al., 2015). Protein domain prediction was conducted by searching the Pfam database with HMMER. To quantify gene expression, the transcript abundance was estimated by RSEM and normalized as FPKM values (Li and Dewey, 2011). DESeq2 was used to analyze the differential expression between sample groups (Love et al., 2014; Varet et al., 2016), with significance thresholds set at an adjusted *p*-value and |Log_2_FC|. Subsequently, enrichment analyses for KEGG pathways (Kanehisa et al., 2008) and GO terms (Ashburner et al., 2000) were carried out based on a hypergeometric distribution test. Finally, the iTAK software was used to screen for potential TFs (Zheng et al., 2016).

### 2.4 Weighted gene co-expression network analysis

The WGCNA R software package is used for weighted gene co-expression network analysis. PCA was conducted between the module eigengenes and the abundance of key flavonoids, and the relationships between modules and metabolites were visualized. Lastly, a regulatory network integrating metabolites, TFs, and structural genes was reconstructed and visualized using Cytoscape.

### 2.5 Quantitative real-time PCR validation

Twelve DEGs were randomly selected for qRT-PCR analysis to verify the accuracy and reliability of the transcriptome sequencing results. RNA extraction and reverse transcription of the roots, stems and leaves of *M. nanmu* were carried out using an RNA Kit (Quanshijin, Beijing, China) and a cDNA Synthesis Kit (TQ2501, OMEGA Bio-Tek). qRT-PCR was carried out using SYBR green master mix (TQ2300, OMEGA Bio-Tek). The reactions were executed on the BIO-RAD CFX Connect Real-Time System (Bio-Rad) under the following PCR conditions: 95°C for 3 min, followed by 39 cycles of 95°C for 5 sec, 60°C for 30 sec, and 60°C for 5 sec. The internal control was the actin (ACT, KM086738.1) of *Cinnamomum camphora*. The primer sequences are listed in **Supplementary Table S1**. The corresponding expression levels were calculated using the 2^−ΔΔCT^ method. Each reaction included three biological replicates and three technical replicates.

### 2.6 Subcellular localization

The subcellular localization of the proteins expressed transiently in *N. benthamiana* protoplasts was previously described by Rolland (2018). Briefly, Agrobacterium (*Agrobacterium tumefaciens*) strain EHA105 that contained constructs *35S-EGFP*, *35S-Cluster-69292-EGFP* and *35S-Cluster-71935-EGFP* was infiltrated into *N. benthamiana* leaves. Protoplasts were isolated 72h after infiltration. Images were obtained by the Zeiss 980 laser scanning confocal microscope (Zeiss GmbH, Oberkochen, Germany).

### 2.7 Statistical analysis

Microsoft Excel 2010 was used for data preprocessing, and subsequently, the GraphPad Prism software was employed to generate the graphical representations (GraphPad PRISM, Version 10.1.2).

## 3 Result

### 3.1 Metabolite analysis in different tissues of *M. nanmu*

**Figure 1:**
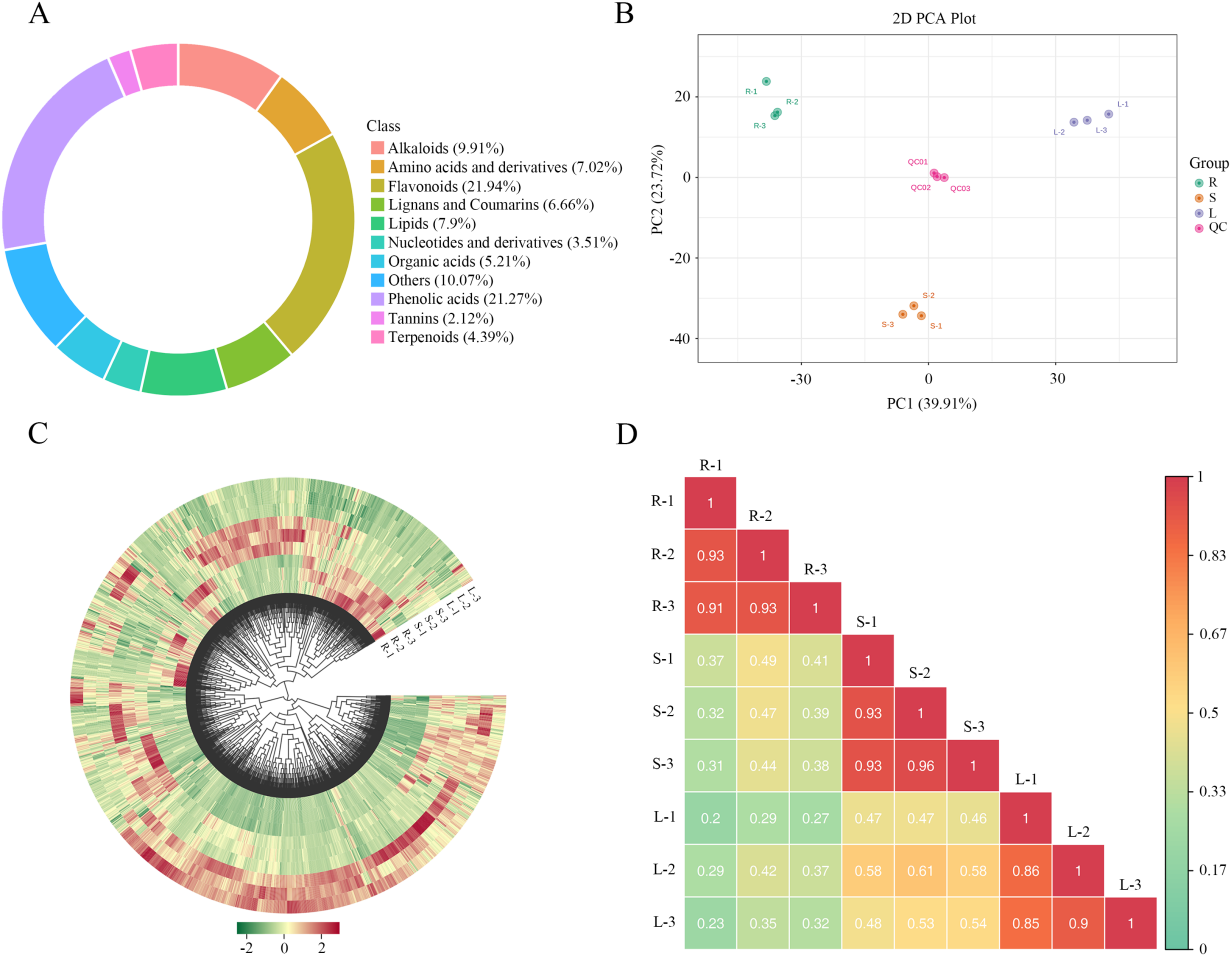
Metabolite composition, abundance, and correlation across different tissues of *M. nanmu*. **(A)** Metabolite classification in different tissues of *M. nanmu*. **(B)** PCA score plot derived from metabolite relative abundance. **(C)** HCA of all detected metabolites. Data are organized from the center to the edge by name, with green indicating relatively low intensity and red indicating relatively high intensity. **(D)** Pairwise Pearson correlation matrix among samples from different tissues. The color scale represents correlation coefficients. Red shades: positive correlations; Blue shades: negative correlations; Green shades: weaker correlations. Specific coefficient values are displayed within each quadrant.

UPLC-MS/MS was used to profile metabolites in different tissues of *M. nanm*. The total ion chromatograms (TIC) represented the summed intensity of all ions at each time point, which was obtained from quality control (QC) samples (**Supplementary Figures S1A, B**). In the extracted ion chromatograms (XIC), peaks of different colors corresponded to distinct metabolite classes (**Supplementary Figures S1C, D**). Overlaid TIC from QC samples showed that the data were reliable, as there is a significant overlap in both retention time and peak response intensity (**Supplementary Figures S1E, F**). In the roots, stems and leaves of *M. nanmu*, a total of 1937 metabolites were detected. Among them, 1036 were identified in the positive ion mode and 901 in the negative ion mode. (**Supplementary Table S2**). These metabolites were categorized into 11 major classes (**Figure 1A**), including flavonoids (21.94%), phenolic acids (21.27%), alkaloids (9.91%), amino acids and derivatives (7.02%), lignans and coumarins (6.66%), lipids (7.9%), nucleotides and derivatives (3.51%), organic acids (5.21%), tannins (2.12%), terpenoids (4.39%), and others (10.07%).

PCA revealed that PC1 and PC2 accounted for 39.91% and 23.72% of the total variance, respectively (**Figure 1B**), with a cumulative contribution rate of 63.63%. The three biological replicates of each tissue type formed tight clusters, and clear separations were observed among roots, stems, and leaves, indicating distinct metabolite profiles among the three tissues. OPLS-DA was applied to pairwise comparisons (R vs S, R vs L, S vs L) for identifying differentially accumulated metabolites. Score plots for each OPLS-DA model are shown in **Supplementary Figures S2A–C**. To validate the models, 200 permutation tests were performed. The values of R^2^X and R^2^Y represent the explained variance of the X and Y matrices, and Q^2^ indicates the predictive ability. Values closer to 1 indicate more stable and reliable models. If Q^2^ > 0.9, it is considered to be extremely excellent. In this study, the following model parameters were obtained: R vs S (R^2^X = 0.722, R^2^Y = 1, Q^2^ = 0.974), R vs L (R^2^X = 0.748, R^2^Y = 1, Q^2^ = 0.985), and S vs L (R^2^X = 0.684, R^2^Y = 1, Q^2^ = 0.97) (**Supplementary Figures S2D–F**). All R^2^Y and Q^2^ values exceeded 0.9, confirming the stability and appropriateness of the models. HCA, based on the relative abundance of all metabolites, showed that the majority of detected metabolites exhibited significant concentration differences among the different plant parts (**Figure 1C**). Furthermore, a correlation heatmap indicated high reproducibility among replicates within each tissue type (**Figure 1D**).

Among all detected metabolites, flavonoids represented the most abundant category, with a total of 425 compounds identified. These included 154 flavones, 140 flavonols, 45 flavanones, 32 flavanols, 23 chalcones, 14 flavanonols, 4 isoflavones, and 13 other flavonoids (**Supplementary Figure S3**).

### 3.2 Differential accumulated metabolites analysis in different tissues of *M. nanmu*

**Figure 2:**
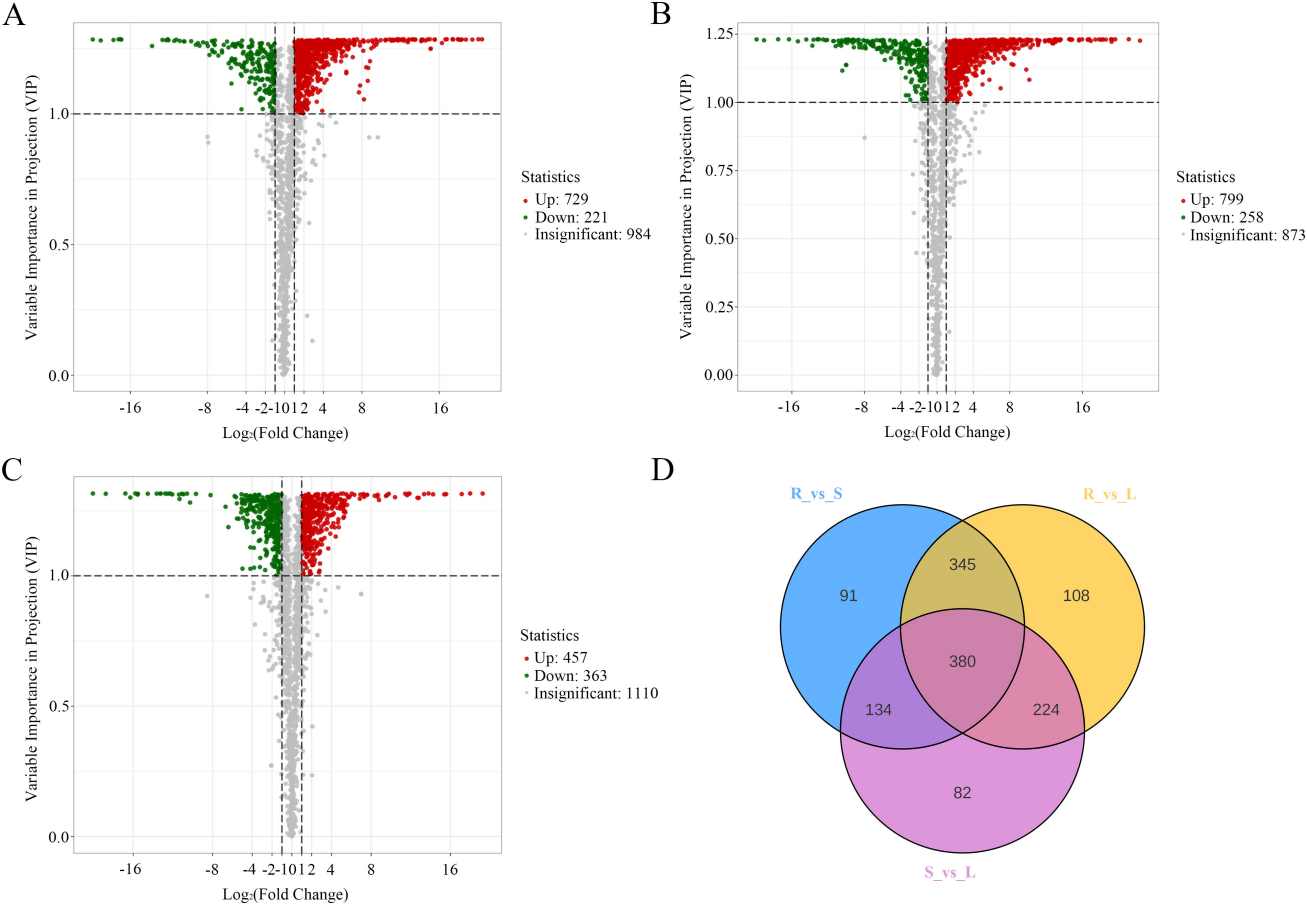
DAMs in different tissues of *M. nanmu*. **(A–C)** Volcano plots displaying DAMs between tissue comparisons: R vs S **(A)**, R vs L **(B)**, and S vs L **(C)**. Red points: up-regulated metabolites; Green points: down-regulated metabolites; Gray points: no significant differences. **(D)** Venn diagram illustrating the overlap and tissue-specific DAMs across the three comparison groups.

**Figure 3:**
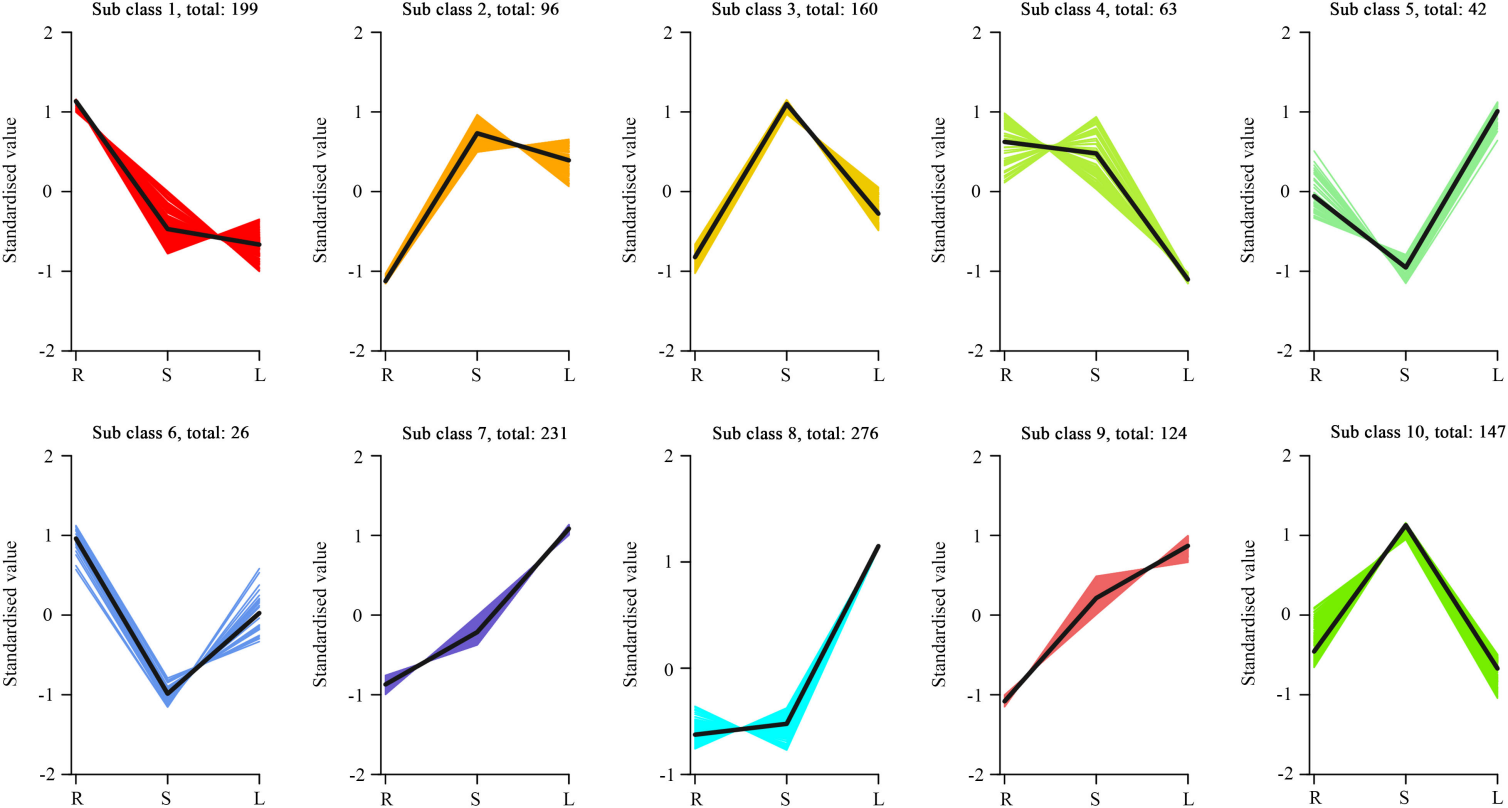
K-means clustering of differential metabolites across three *M. nanmu* tissues based on accumulation patterns. The x-axis indicates tissue type, while the y-axis shows Z-score normalized relative metabolite abundance.

To gain a deeper understanding of the metabolic differences between R vs S, R vs L, and S vs L, we identified DAMs using thresholds of Fold Change (FC) ≥ 2 or ≤ 0.5 and VIP ≥ 1. We had detected a total of 1,364 DAMs (**Supplementary Table S3**). The screening results are visualized in volcano plots (**Figures 2A–C**) and a Venn diagram (**Figure 2D**). Specifically, 950 DAMs (729 up- and 221 down-regulated) were identified between R vs S (**Figure 2A**); 1,057 DAMs (799 up- and 258 down-regulated) between R vs L (**Figure 2B**); and 820 DAMs (457 up- and 363 down-regulated) between S vs L (**Figure 2C**). To reveal major trends and tissue-specific accumulation patterns of these metabolites, all DAMs were subjected to K-means clustering analysis and grouped into 10 subclasses (subclasses 1 to 10, **Figure 3**). Subclasses 1, 4, and 6-containing 199, 63, and 26 DAMs, respectively,-showed the highest abundance in roots and lower levels in other tissues. These were predominantly alkaloids (32.66%), amino acids and derivatives (22.22%), and alkaloids (23.08%), respectively. Subclasses 2, 3, and 10, comprising 96, 160, and 147 DAMs, exhibited peak accumulation in stems, with the most abundant classes being flavonoids (26.04%), phenolic acids (32.50%), and phenolic acids (33.33%). Subclasses 5, 7, 8, and 9, consisting of 42, 231, 276, and 124 DAMs, were most abundant in leaves, and were mainly composed of phenolic acids (26.19%), flavonoids (34.63%), flavonoids (39.49%), and flavonoids (30.56%). Among these, subclass 8 contained the highest number of DAMs (**Supplementary Table S3**). DAMs from the three comparison groups (R vs S, R vs L, and S vs L) were categorized into 11 classes (**Supplementary Figure S4**). HCA indicated that flavonoids constituted the majority of DAMs (**Supplementary Figure S5**). Therefore, subsequent analysis focused on flavonoid variations across different tissues.

### 3.3 Variation in flavonoid compounds in different parts of *M. nanmu*

**Figure 4:**
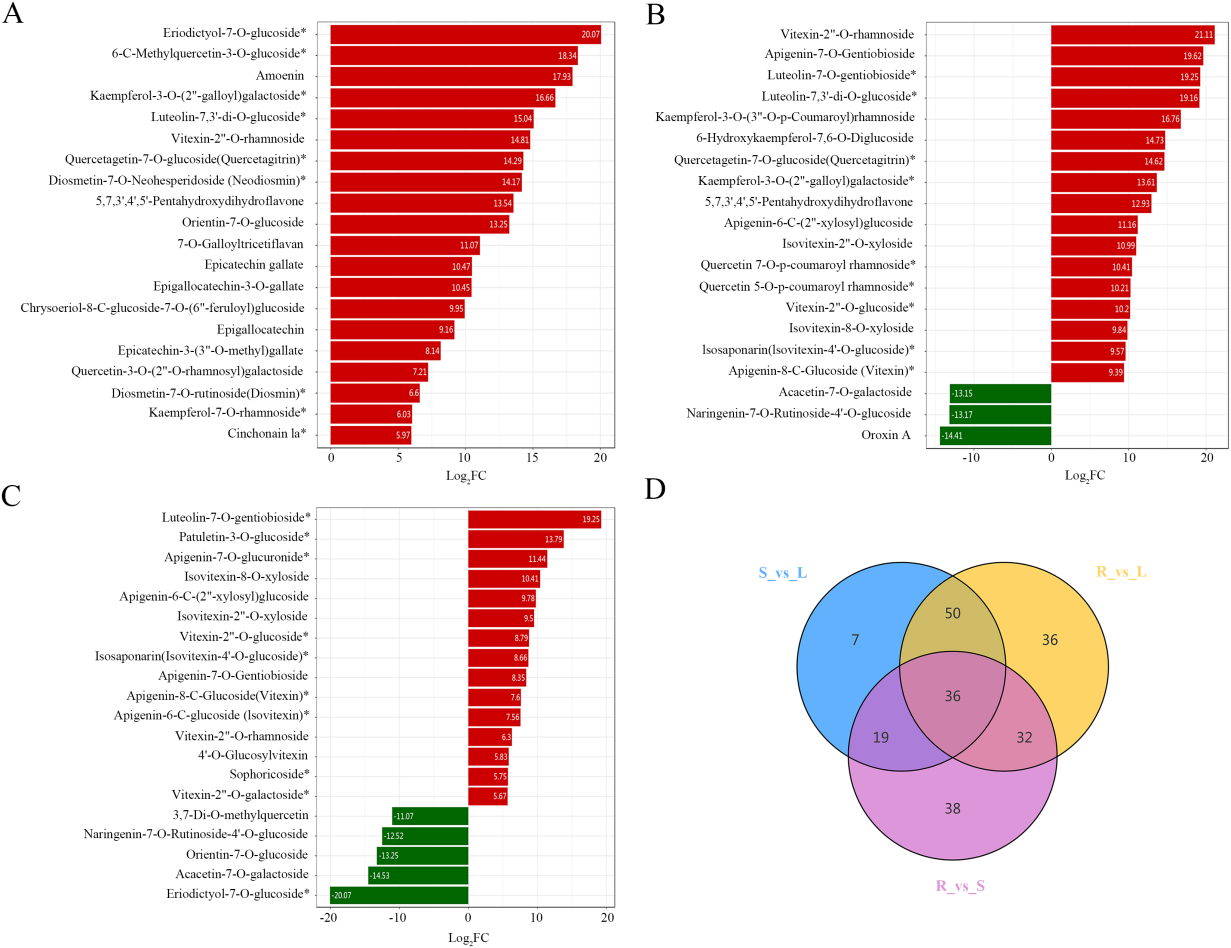
DAMs across *M. nanmu* tissues. **(A-C)** Top 20 flavonoids ranked by fold change in pairwise comparisons: R vs S **(A)**, R vs L **(B)**, and S vs L **(C)**. Red bars: up-regulated metabolites; Green bars: down-regulated metabolites. **(D)** Venn diagram of DAMs.

Flavonoids play a crucial role in plant adaptation and defense against environmental stresses. Besides their physiological functions in plants, they have notable medicinal and nutritional benefits (Zhang et al., 2017). Therefore, we further examined the composition and accumulation patterns of flavonoids in the roots, stems, and leaves of *M. nanmu*. Based on their accumulation patterns, flavonoids were generally most abundant in leaves, followed by stems, with lower levels in roots (**Supplementary Figure S6**). We further conducted pairwise comparisons of flavonoid metabolites for R vs S, R vs L, and S vs L (**Table 1**). Between R vs S, 125 flavonoids showed differential accumulation (121 up- and 4 down-regulated), with fold-change values ranging from 0.46 to 1,097,450.34 (**Figure 4A**). The top 3 flavonoids with the highest fold changes are Eriodictyol-7-O-glucoside (1,097,450.34-fold), 6-C-Methylquercetin-3-O-glucoside (332,494.62-fold), and Amoenin (249,958.15-fold). A total of 154 flavonoids exhibited differential accumulation between R vs L (148 up-regulated and 6 down-regulated), with fold changes ranging from 0.00 to 2,267,330.96 (**Figure 4B**). The 3 most differentially accumulated flavonoids are Vitexin-2’’-O-rhamnoside (2,267,330.96-fold), Apigenin-7-O-Gentiobioside (806,805.00-fold), and Luteolin-7-O-gentiobioside (621,552.05-fold). 112 flavonoids differentially accumulated between S vs L (83 up- and 29 down-regulated), exhibiting fold change values ranging from 0.00 to 621,552.05 (**Figure 4C**). The top 3 flavonoids ranked by fold change are Luteolin-7-O-gentiobioside (621,552.05-fold), Patuletin-3-O-glucoside (14,165.29-fold), and Apigenin-7-O-glucuronide (2,772.42-fold). Venn diagram analysis further identified 81 tissue-specific DAMs and 36 shared DAMs across all three tissue comparisons (**Figure 4D**).

**Table 1:** Statistics of DAM types across comparison groups.

Classification	Numbers	R vs S	R vs L	S vs L
Flavones	75	35	53	43
Flavanones	22	11	18	13
Flavonols	72	42	50	19
Flavanols	20	17	12	14
Flavanonols	8	5	6	6
Other Flavonoids	9	8	5	6
Chalcones	9	6	7	8
Isoflavones	3	1	3	3
Total	218	125	154	112

### 3.4 Transcriptome sequencing and functional characterization of DEGs

**Figure 5:**
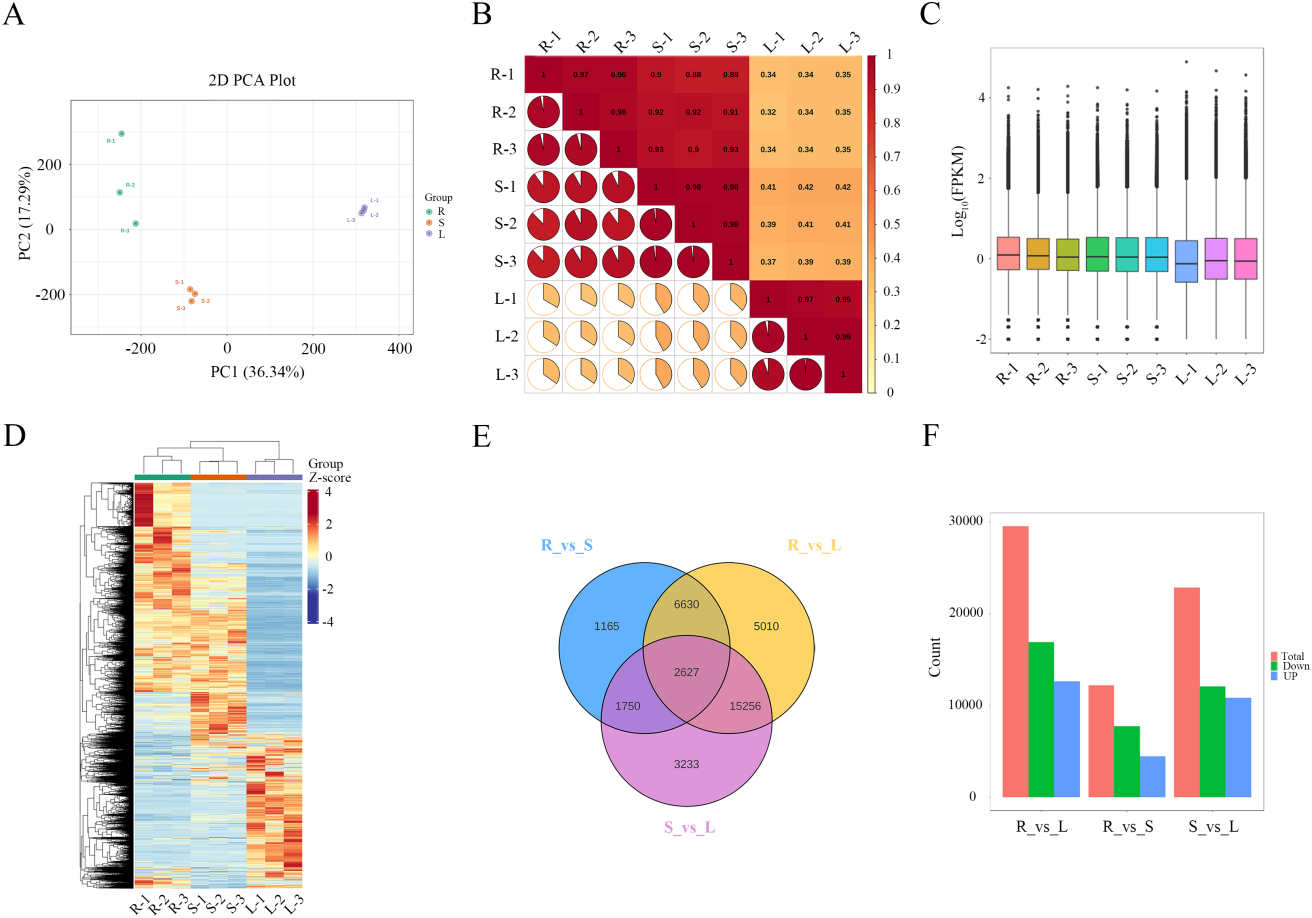
Transcriptomic analysis and identification of DEGs across *M. nanmu* tissues. **(A)** PCA of each sample. **(B)** Pairwise Pearson correlation analysis of gene expression profiles. **(C)** Box plot of all samples’ gene expression levels. **(D)** Expression of DEGs in different tissues. **(E)** Venn diagram. **(F)** Number of DEGs in each tissue.

To investigate the transcriptomic profiles of different tissues in *M. nanmu*, we performed RNA sequencing on nine samples. The RNA-seq dataset yielded 75.46 Gb of clean data, with each sample containing ≥ 6 Gb. The Q30 base percentage exceeded 94%, and GC content ranged from 45.15% to 47.14% (**Supplementary Table S4**). Clean reads were assembled using Trinity, and the resulting transcript sequences served as the reference for subsequent analyses. As shown in **Supplementary Figure S7**, the assembly exhibited high completeness; the longest cluster sequences obtained after Corset-based hierarchical clustering were defined as unigenes for downstream analysis. A total of 319,687 transcripts and 168,717 unigenes were generated (**Supplementary Table S5**), with length distributions displayed in **Supplementary Figure S8**. Unigene sequences were annotated by aligning them against the KEGG, NR, Swiss-Prot, TrEMBL, COG/KOG, and GO databases using DIAMOND BLASTX. Amino acid sequences predicted from unigenes were further analyzed with HMMER against the Pfam database. The annotation results were as follows: 71,313 (42.27%), 98,130 (58.16%), 66,303 (39.30%), 99,381 (58.90%), 57,048 (33.81%), 86,000 (50.97%), and 67,499 (40.01%) unigenes were annotated to the respective databases (**Supplementary Table S6**). PCA indicated that PC1 and PC2 accounted for 36.34% and 17.29% of the gene expression variance among samples, respectively (**Figure 5A**). Correlation analysis between biological replicates showed that an |r| value closer to 1 (represented by red color) indicates stronger reproducibility between replicates (**Figure 5B**). Gene expression levels spanned six orders of magnitude, from 10^-2^ to 10^4^ (**Figure 5C**). These results demonstrate that the sequencing quality was sufficient for further analysis.

To identify differentially expressed genes (DEGs) associated with flavonoid biosynthesis in different tissues of *M. nanmu*, we performed differential expression analysis between sample groups using DESeq2, with screening thresholds set at |log_2_FC| ≥ 1 and FDR < 0.05. A total of 35,671 DEGs were detected, showing significant expression variation among R, S, and L (**Figure 5D**). Pairwise comparisons revealed 12,172 DEGs (4,429 up-regulated, 7,743 down-regulated) between R vs S, including genes encoding UDP-glycosyltransferase, flavonol synthase, isoflavone 2’-hydroxylase, and anthocyanidin 3-O-glucosyltransferase. Between R vs L, a total of 29,523 DEGs (12,599 up-regulated, 16,924 down-regulated) were detected, encompassing leucoanthocyanidin reductase, UDP-glycosyltransferase, anthocyanidin synthase, flavonol synthase, chalcone synthase, flavonoid 3’-hydroxylase, flavanone 3-hydroxylase, and isoflavone 2’-hydroxylase. Between S vs L, 22,866 DEGs (10,802 up-regulated, 12,064 down-regulated) involving anthocyanidin reductase, leucoanthocyanidin reductase, UDP-glycosyltransferase, anthocyanidin synthase, flavonoid 3’-hydroxylase, flavonol synthase, anthocyanidin 3-O-glucosyltransferase, and isoflavone 2’-hydroxylase (**Figures 5E, F**). These DEGs are likely to play crucial roles in regulating tissue-specific flavonoid biosynthesis in *M. nanmu*.

**Figure 6:**
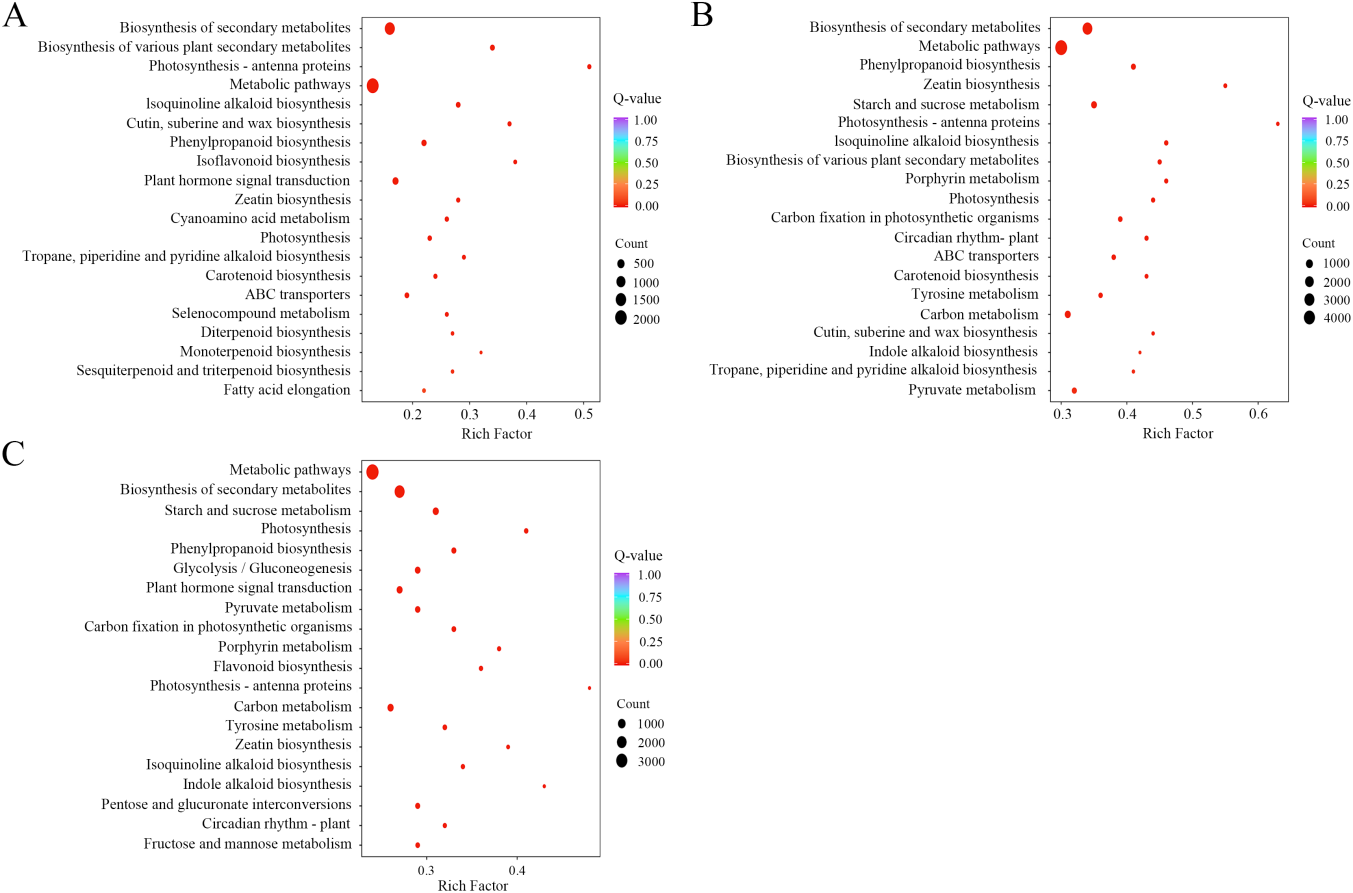
KEGG pathway enrichment analysis of DEGs across three tissue comparisons. R vs S **(A)**, R vs L **(B)** and S vs L **(C)** display the top 20 significantly enriched KEGG pathways for each comparison. The y-axis denotes pathway names, and the x-axis represents the richness factor, with greater values indicating higher enrichment levels. Point size corresponds to the number of DEGs mapped to a given pathway, and color intensity reflects the statistical significance of the enrichment.

We conducted GO and KEGG pathway enrichment analyses to elucidate the functional implications of the identified DEGs. The GO annotation categorized DEGs into three principal categories: Molecular Function (MF), Biological Process (BP), and Cellular Component (CC). Across the R vs S, R vs L, and S vs L comparisons, GO classification identified 44, 45, and 44 subcategories, respectively. These DEGs were further grouped into 32 functional subcategories based on homology mapping (**Supplementary Figures S9A–C**). In the BP categories, DEGs were predominantly associated with cellular processes, metabolic processes, and response to stimulus. Within the CC categories, the most represented terms encompassed cellular anatomical entities and protein-containing complexes. For MF, the majority of DEGs were implicated in binding, catalytic activity, and transcription regulator activity. KEGG pathway enrichment analysis revealed that the DEGs were mapped to 146, 147, and 145 pathways in the R vs S, R vs L, and S vs L comparisons, respectively. Notably, the biosynthesis of flavonoids (ko00941) and isoflavonoids (ko00943) was significantly enriched among these pathways (**Figures 6A–C**).

### 3.5 Key structural genes and pathway mapping in the flavonoid biosynthetic pathway

**Figure 7:**
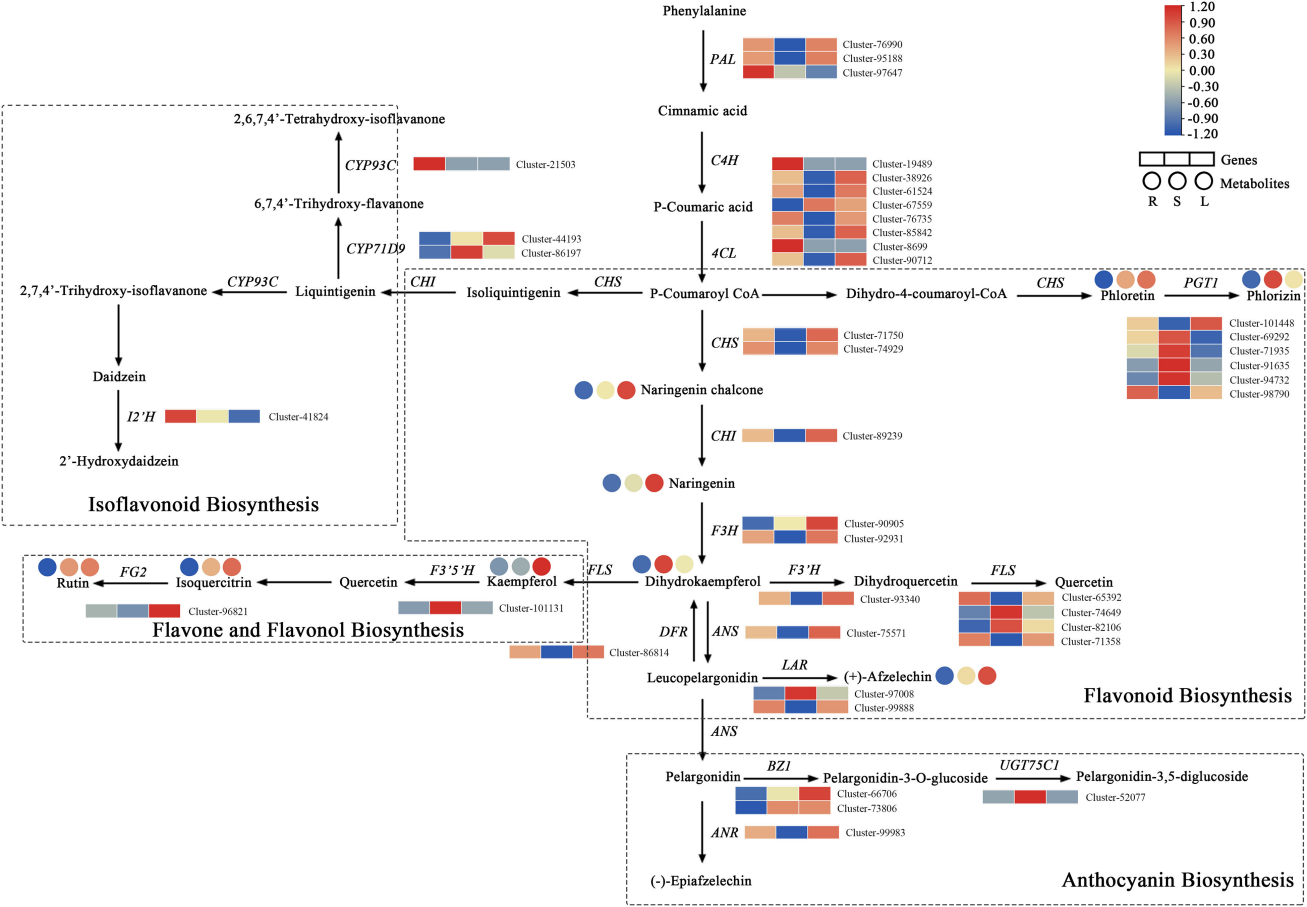
Coordinated expression patterns of structural genes and associated metabolites in the flavonoid biosynthetic pathway. Red: up-regulation; Blue: down-regulation; Squares: DEGs; Circles: DAMs.

Based on KEGG enrichment analysis and functional annotation of DEGs, a total of 41 genes encoding 19 key enzymes involved in flavonoid biosynthesis were identified (**Supplementary Table S7**). The flavonoid biosynthetic pathway in different tissues of *M. nanmu* was reconstructed based on these 41 genes and key enzymes from the KEGG pathway (**Figure 7**). The 19 key enzymes are as follows: phenylalanine ammonia-lyase (PAL), 4-coumarate-CoA ligase (4CL), chalcone synthase (CHS), chalcone isomerase (CHI), isoflavone 2’-hydroxylase (I2’H), flavanone 3-hydroxylase (F3H), flavonoid 3’-hydroxylase (F3’H), flavonoid 3’,5’-hydroxylase (F3’5’H), flavonol synthase (FLS), dihydroflavonol 4-reductase (DFR), leucoanthocyanidin reductase (LAR), anthocyanidin synthase (ANS), anthocyanidin reductase (ANR), anthocyanidin 3-O-glucosyltransferase (BZ1), flavonoid 6-hydroxylase (CYP71D9), 2-hydroxyisoflavanone synthase (CYP93C), flavonol-3-O-glucoside L-rhamnosyltransferase (FG2), anthocyanidin 3-O-glucoside 5-O-glucosyltransferase (UGT75C1), and phlorizin synthase (PGT1). Among these enzymes, UDP-glycosyltransferases (UGTs) were the most abundant, with 10 genes identified, including BZ1, FG2, UGT75C1, and PGT1, suggesting their crucial role in regulating tissue-specific flavonoid biosynthesis in *M. nanmu*.

**Figure 8:**
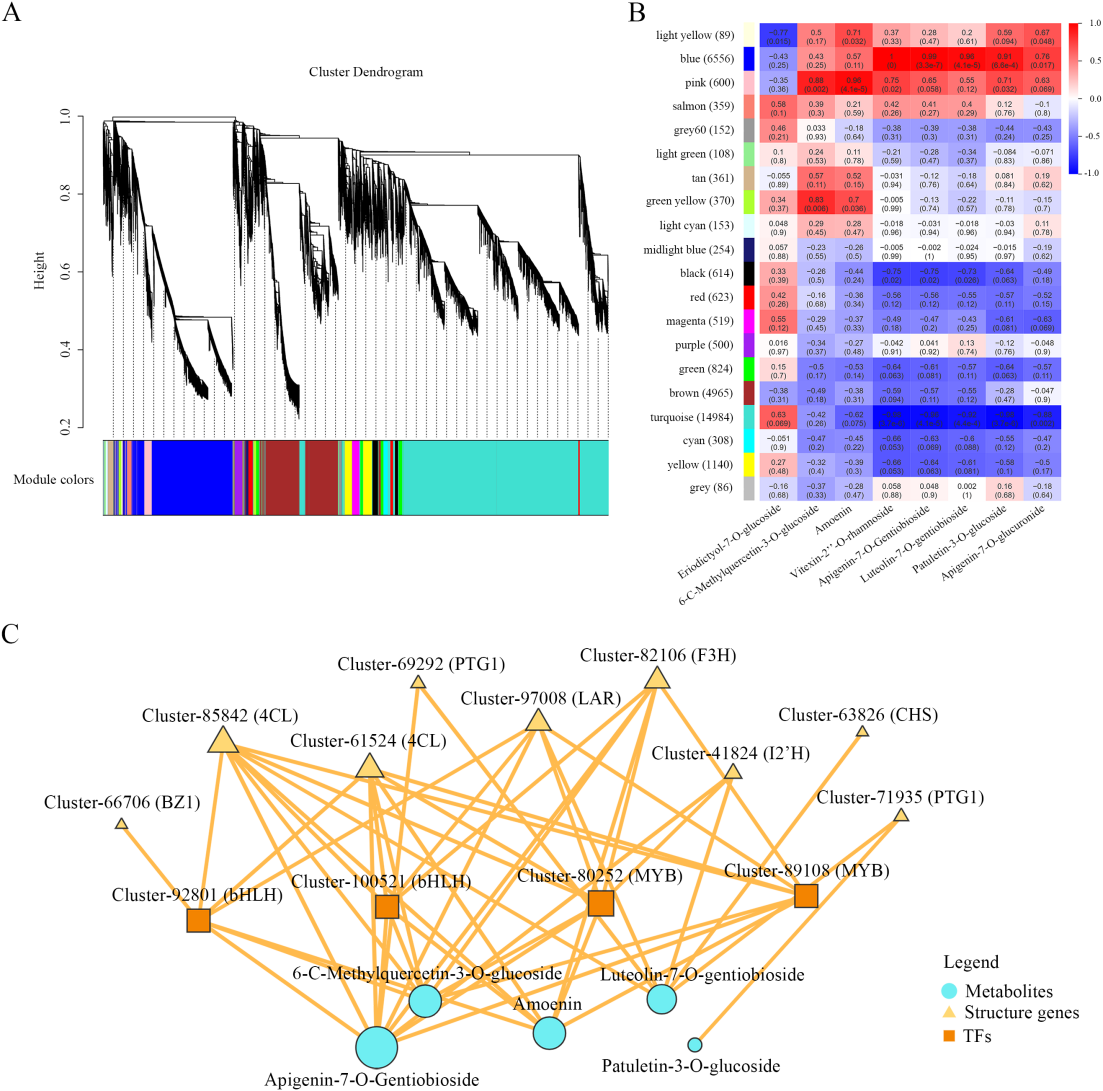
Gene co-expression network analysis. **(A)** Cluster dendrogram of genes grouped into 20 distinct co-expression modules, each designated by a unique color. **(B)** Correlation heatmap between module eigengenes and flavonoid metabolite levels. Red shades: positive correlations; Blue shades: negative correlations. **(C)** Relevance network diagram. Yellow triangles: structural genes. Orange squares: transcription factors. Blue circles: metabolites. Node size reflects the connectivity degree (number of significant correlations per node).

Previous studies have shown that UGTs play crucial biological roles in phytohormone homeostasis, detoxification, secondary metabolism, and stress responses (Ren et al., 2025). As shown in **Figure 8C**, Cluster-69292 and Cluster-71935 exhibited strong positive correlations with TFs and metabolites. Notably, Cluster-71935 showed the highest expression level among all differentially expressed UGTs. To investigate its potential biological function, we performed subcellular localization analysis for this gene. The amino acid sequences of Cluster-69292 and Cluster-71935 were analyzed using the online prediction tool TargetP-2.0, which indicated cytoplasmic localization for both proteins. This prediction was consistent with our experimental observations, as shown in **Supplementary Figure S10**, strong GFP fluorescence signals for 35S-69292-EGFP and 35S-71935-EGFP were predominantly detected in the cytoplasm.

### 3.6 Gene co-expression network analysis

Through WGCNA, 20 distinct modules exhibiting similar gene expression patterns were identified (**Figure 8A**). The number of genes per module ranged from 86 to 14,984, with four modules (blue, brown, turquoise, and yellow) all containing over 1,000 genes (**Figure 8B**). PCA between module eigengenes and the content of eight key flavonoids (Eriodictyol-7-O-glucoside, 6-C-Methylquercetin-3-O-glucoside, Amoenin, Vitexin-2’’-O-rhamnoside, Apigenin-7-O-Gentiobioside, Luteolin-7-O-gentiobioside, Patuletin-3-O-glucoside, and Apigenin-7-O-glucuronide, the top three significantly accumulated differential metabolites in each tissue) revealed that two modules (blue and pink) were positively correlated with flavonoid content, while two others (turquoise and black) were negatively correlated. This indicates that the eigengenes of these four modules are closely associated with flavonoid accumulation in the roots, stems, and leaves of *M. nanmu*. Therefore, the blue, pink, turquoise, and black modules were selected for further investigation. Previous studies have shown that MYB and bHLH TFs are involved in the regulation of flavonoid biosynthesis (Fang et al., 2022; Su et al., 2025). Accordingly, we performed correlation analysis on structural genes positively correlated with metabolite levels and MYB/bHLH TFs within these four modules, identifying 13 structural genes, 3 MYB-related genes, and 3 bHLH TFs (**Supplementary Table S8**). As shown in **Figure 8C**, two MYB (Cluster-80252, Cluster-89108) and two bHLH (Cluster-92801, Cluster-100521) genes showed strong positive correlations (r > 0.8, *p* < 0.05) with key structural genes, including 4CL (Cluster-85842, Cluster-61524), CHS (Cluster-63826), F3H (Cluster-82106), I2’H (Cluster-41824), LAR (Cluster-97008), PGT1 (Cluster-69292, Cluster-71935), and BZ1 (Cluster-66706). These results suggest that these TFs act as core regulators coordinating flavonoid biosynthesis.

### 3.7 Validation by qRT-PCR

To validate the accuracy of the transcriptomic data, twelve differentially expressed genes were selected and analyzed using qRT-PCR. The results demonstrated that the expression trends observed by qRT-PCR were largely consistent with those from the transcriptome sequencing, confirming the reliability and validity of the transcriptomic data (**Supplementary Figure S11**).

## 4 Discussion

*M. nanmu*, an ecologically and economically important timber species, has long been recognized for its commercial wood value (Flora of China Editorial Committee, 2018). Previous studies have shown that *M. nanmu* possesses bioactive properties, including antioxidant, antitumor, and free radical scavenging activities, suggesting considerable potential for healthcare, nutraceutical, and pharmaceutical applications (Liang et al., 2023). And flavonoid secondary metabolites play a significant role in the growth and development of plants as well as in their resistance to both biotic and abiotic stresses (Zhang et al., 2017). However, the tissue-specific distribution and biosynthetic mechanisms of flavonoids and other secondary metabolites in *M. nanmu* remain largely uncharacterized. Here, we present the first comprehensive study integrating widely targeted metabolomics with transcriptomics to systematically investigate metabolite accumulation and gene expression patterns in roots, stems, and leaves, three distinct tissues of *M. nanmu*. The research results not only provide multi-omics evidence for a deeper understanding of the tissue-specific differentiation of *M. nanmu*’s secondary metabolism, but also offer theoretical support and key targets for the development of its medicinal value and genetic improvement.

### 4.1 Metabolite characteristics of different tissues in *M. nanmu*

Metabolomic profiling revealed the identification of a total of 1937 metabolites across three tissues of *M. nanmu*, which were categorized into 11 major classes, including flavonoids, phenolic acids and alkaloids. Among these, flavonoids (21.94%) and phenolic acids (21.27%) represented the two most abundant categories, a distribution pattern consistent with that observed in most plant species such as *Taraxacum mongolicum* and *Lactuca indica* L (Hao et al., 2023; Zhao et al., 2024). Both PCA and OPLS-DA models (with Q² > 0.9) verified a marked metabolic divergence among roots, stems and leaves, coupled with a high correlation between biological replicates. These results indicated the tissue-specificity of metabolite accumulation in *M. nanmu*. Tissue-specific metabolic differentiation was further validated by K-means clustering analysis, alkaloids and amino acids and derivatives were preferentially enriched in roots, phenolic acids accounted for a dominant proportion in stems, while flavonoids served as the core differential metabolites in leaves. These findings suggest that the metabolites may have different biological functions in different tissue types (Dong et al., 2014). As underground organs, roots can interact with soil microorganisms and various compounds. Alkaloids typically act as antimicrobial, insect-resistant, and allelopathic agents in plants (Ziegler and Facchini, 2008). Thus, the accumulation of alkaloids at high concentrations in roots may facilitate stress resistance and the regulation of rhizosphere microorganisms. Phenolic acids and their derivatives (such as lignin precursors) are closely associated with plant mechanical strength, vascular system development, and antioxidant protection during long-distance transport (Vanholme et al., 2010). The enrichment of these metabolites in stems is likely an adaptive trait that has evolved in response to the roles of the stems in nutrient translocation, mechanical support, and defense. The significantly enriched flavonoids in leaves align with the dual function of this organ, which is the primary site of photosynthesis and the first line of defense against environmental stresses (such as ultraviolet radiation, herbivores, and pathogens). Luteolin-7-O-glucoside (cynaroside), the most abundant flavonoid in leaves, serves as an ultraviolet screen, antioxidant, and antimicrobial compound (Agati et al., 2012).

As the most numerous classes of secondary metabolites in *M. nanmu*, flavonoids accounted for 425 identified compounds across roots, stems, and leaves. These metabolites were predominantly flavones and flavonols, followed by flavanones, flavanols, and chalcones (**Supplementary Figure S3**). The most abundant flavonoid in roots was 3’,4,4’,5,7-Pentahydroxyflavan (Luteoforol), while stems exhibited the highest content of epicatechin gallate, a catechin derivative known for its antitumor, anti-inflammatory, and antioxidant properties, including the inhibition of cancer cells (Li et al., 2022). In leaves, luteolin-7-O-glucoside (cynaroside) was the predominant flavonoid. This compound has been reported to possess antimicrobial, anticancer, antifungal, hepatoprotective, antidiabetic, antioxidant, and anti-inflammatory activities, and may also participate in drought stress responses in plants (Bouyahya et al., 2023; Rao et al., 2024). Pairwise comparisons between tissue groups revealed 218 differentially accumulated flavonoids (**Table 1**). These metabolites exhibited distinct tissue-specific accumulation patterns, with the highest overall flavonoid content observed in leaves, followed by stems, and the lowest in roots (**Supplementary Figure S5**). This suggests that leaves are the primary site of flavonoid biosynthesis and accumulation in *M. nanmu*, a pattern consistent with findings in *Areca catechu*, *Artemisia argyi*, and *Ginkgo biloba* (Fu et al., 2022; Miao et al., 2022; Lai et al., 2023). In contrast, other species, such as *Hibiscus Manihot*, accumulate the highest flavonoid levels in flowers (Zhou et al., 2022), indicating species-specific metabolic allocation. It should be noted that the present study focused on roots, stems and leaves, future work incorporating floral and fruit tissues will provide a more comprehensive understanding of flavonoid accumulation patterns in *M. nanmu*.

### 4.2 Enzymes and key structural genes related to flavonoid biosynthesis

By integrating transcriptomic and metabolomic analyses, we identified 19 key enzymes (including PAL, 4CL, CHI, UGTs, etc.) encoded by 41 genes, and reconstructed the flavonoid biosynthetic pathway in *M. nanmu* (**Figure 7**). Among these, the CHS encoding gene Cluster-71750 was significantly up-regulated in leaves, consistent with the high accumulation of flavonoids in this tissue. This aligns with the established role of CHS as a rate-limiting enzyme in flavonoid biosynthesis (Dixon and Paiva, 1995), and corroborates findings in *Citrus* species (Wang et al., 2018; Borredá et al., 2022), indicating the conservation of this regulatory mechanism in plants. In future studies, molecular approaches such as gene editing could be employed to modulate the expression of this gene, to enhance total flavonoid content in leaves and provide a key target for breeding high-flavonoid *M. nanmu* varieties. Notably, UGTs constituted the largest group among the differentially expressed structural genes. Their encoded products catalyze glycosylation, which enhances the solubility and stability of flavonoids (Le Roy et al., 2016), underscoring glycosylation as a key biochemical mechanism underlying flavonoid structural diversity in plants (Heiling et al., 2021). This observation is consistent with the expression pattern of flavonoids in *M. nanmu*. Among the top 20 flavonoids with the highest fold changes in the comparative analysis of roots, stems and leaves, the majority were glycosylated flavonoids (such as O-glycosides) exhibiting substantial fold differences. These results indicate that glycosylation modification may serve as a key biochemical switch governing the tissue-specific accumulation, stability and biological activity of flavonoids in *M. nanmu* (Bowles et al., 2005). WGCNA further revealed a strong correlation between two specific UGT genes (Cluster-69292 and Cluster-71935), flavonoid accumulation and core TFs. Subsequent subcellular localization assays confirmed that these two key UGT proteins were localized to the cytoplasm (**Supplementary Figure S10**). This subcellular localization is of great significance, as it suggests that the glycosylation of flavonoid aglycones occurs in the cytoplasm; the glycosylated products may then be transported to vacuoles for storage or secreted into the extracellular space. This process not only prevents the potential cytotoxicity of aglycones to plant cells, but also enables their specific distribution and functional differentiation across distinct cellular compartments or tissues (Zhao, 2015). Collectively, these findings demonstrate that UGT-mediated glycosylation acts not only as a major driver of flavonoid structural diversification, but also as a core regulatory node underlying the tissue-specific accumulation and functional specialization of flavonoids, which is consistent with the findings of previous studies. Previous studies have demonstrated that UGTs are generally considered soluble cytosolic enzymes, as they lack clear transmembrane domains or membrane-targeting signals and often exhibit activity in the cytoplasmic compartment (Jones and Vogt, 2001). However, some UGTs have been localized to the endoplasmic reticulum lumen or vacuoles (Ullmann et al., 1993; D’Alessio et al., 2010), possibly to facilitate glycosylation of specific substrates (Offen et al., 2006). The substrate specificity and catalytic properties of UGT genes, as well as the biological functions of their flavonoid glycoside products, need to be further elucidated through *in vitro* enzyme activity assays, gene knockout/overexpression experiments and other related approaches.

### 4.3 TFs and candidate genes involved in the biosynthesis of flavonoids

TFs are DNA-binding proteins that can interact with promoter regions of target genes and other protein domains to execute specific regulatory functions. They serve as critical regulators influencing growth, development, physiological processes, and secondary metabolism in higher plants (Zhang et al., 2022). WGCNA has been widely adopted as a robust approach for identifying candidate genes and transcriptional regulators from transcriptomic datasets (Pei et al., 2017). WGCNA, in our study, was employed to delineate co-expression modules correlated with flavonoid accumulation, leading to the identification of four key modules (blue, pink, turquoise, and black) and four core TFs (two MYB and two bHLH factors) putatively involved in the regulation of flavonoid biosynthesis (**Figure 8C**). Previous studies have established the central role of specific TFs in flavonoid pathway regulation. For instance, SbMYB3 in *Scutellaria baicalensis* directly binds to and activates the promoter of SbFNSII-2, promoting root-specific accumulation of flavones such as baicalein and wogonin (Fang et al., 2023). Similarly, SlbHLH95 in tomato acts as a bifunctional regulator, directly binding the promoters of *SlF3H* and *SlFLS*, interacting with SlMYB12 to co-regulate their expression, and enhancing resistance to *Botrytis cinerea* by repressing *SlBG10* (Su et al., 2025). Consistent with the findings of the present study, MYB and bHLH FTs in *M. nanmu* exhibited a strong co-expression correlation with both upstream phenylpropanoid pathway genes (such as 4CL, CHS) and downstream modification genes (such as UGT, CHS, F3H, I2’H, and LAR), which were directly linked to the flavonoid glycosides highly accumulated in various tissues. These results revealed the potential regulatory pathway underlying tissue-specific flavonoid biosynthesis in *M. nanmu*, the activation of MYB and bHLH TFs upregulates a series of structural genes ranging from the synthesis of universal precursors to glycosylation modification, ultimately modulating the efficient biosynthesis and accumulation of flavonoid glycosides in specific tissues (Xu et al., 2015). However, the molecular characteristics of the transporters and the regulatory mechanisms governing how flavonoid glycosides are translocated from their sites of synthesis to storage compartments or transported long-distance between organs remain largely unknown. It should be emphasized that the TFs identified in this study were primarily screened through computational and statistical approaches. Further experimental validation can use molecular techniques, such as yeast one-hybrid assays to confirm DNA-binding activity and yeast two-hybrid systems to examine protein-protein interactions, which will be essential to verify their regulatory functions and physical interactions with target gene promoters. Interestingly, no WD40 component of the canonical MYB-bHLH-WD40 (MBW) complex was detected among the core TFs identified here. This contrasts with the well-established MBW-dependent regulatory model in *Arabidopsis thaliana* (Xie et al., 2016) and suggests that flavonoid regulation in *M. nanmu* may involve alternative mechanisms, potentially related to its specific morphogenesis, stress adaptation, hormone signaling, or metabolic regulation. This hypothesis warrants further investigation through physiological and stress-response experiments.

## 5 Conclusion

*M. nanmu* is rich in diverse bioactive metabolites and exhibits notable biological activities such as antioxidant and antitumor effects. This study provides a systematic elucidation of the metabolic and transcriptional basis underlying tissue-specific flavonoid biosynthesis in *M. nanmu*. Integrated metabolomic and transcriptomic profiling of roots, stems, and leaves identified 218 differentially accumulated flavonoids and 35,671 DEGs. Reconstruction of the flavonoid biosynthetic pathway revealed key structural genes, with UGTs representing the most abundant family. Subcellular localization in tobacco mesophyll protoplasts demonstrated cytoplasmic localization of two candidate proteins (Cluster-69292 and Cluster-71935). WGCNA further identified four core TFs (MYB and bHLH) as putative regulators of flavonoid biosynthesis. Finally, the reliability of the transcriptomic data was confirmed by qRT-PCR validation. These findings offer the first multi-omics insight into the regulatory mechanisms of flavonoid biosynthesis in *M. nanmu*, establishing a theoretical foundation for functional gene characterization and molecular breeding aimed at trait improvement.

## Data Availability

The datasets presented in this study can be found in online repositories. The names of the repository/repositories and accession number(s) can be found in the article/**Supplementary Material**.
