# Supplementary material for: Integrated metabolomic and transcriptomic profiling elucidates the tissue-specific biosynthesis and regulation of flavonoids in Machilus nanmu
Source: Front Plant Sci. 2026 Jan 8;16:1731446. doi: 10.3389/fpls.2025.1731446 (PMC12823935; doi:10.3389/fpls.2025.1731446)
Supplement: Supplementary file 1 [file DataSheet1.docx]

Supplementary Material

# Supplementary Figures and Tables

## Supplementary Figures


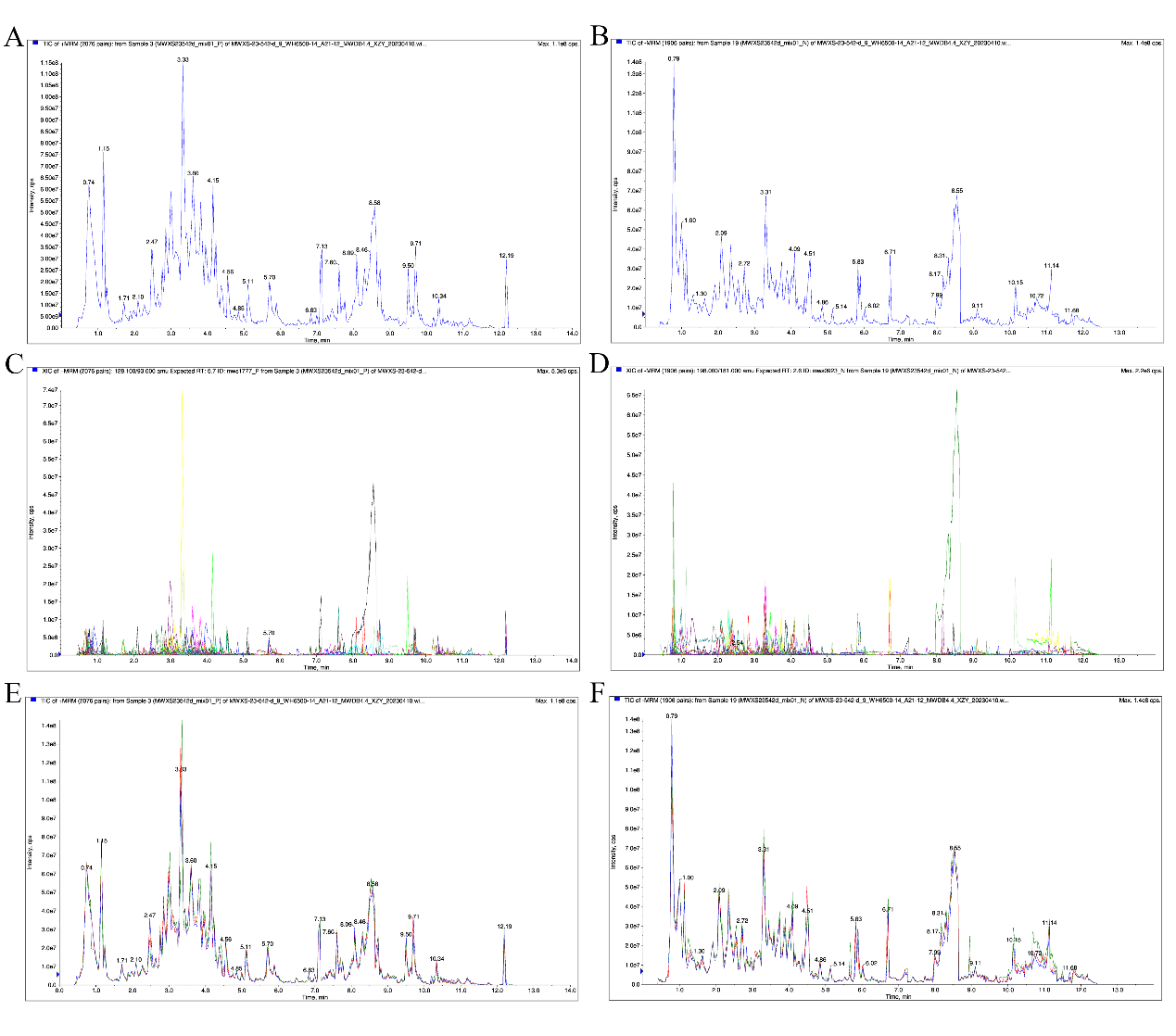


**Supplementary Figure S1.** Qualitative, quantitative and quality control analysis of metabolites. **(A-B)**: Analysis of positive and negative total ion flow diagrams of mixed samples by mass spectrometry. **(C-D)**: Positive and negative multi-peak map of MRM metabolite detection. **(E-F)**: Positive and negative TIC overlap map of QC samples detected by mass spectrometry.

Note: Abscissa is the retention time of metabolite detection (time), ordinate is the ion current intensity of ion detection (cps, count per second).


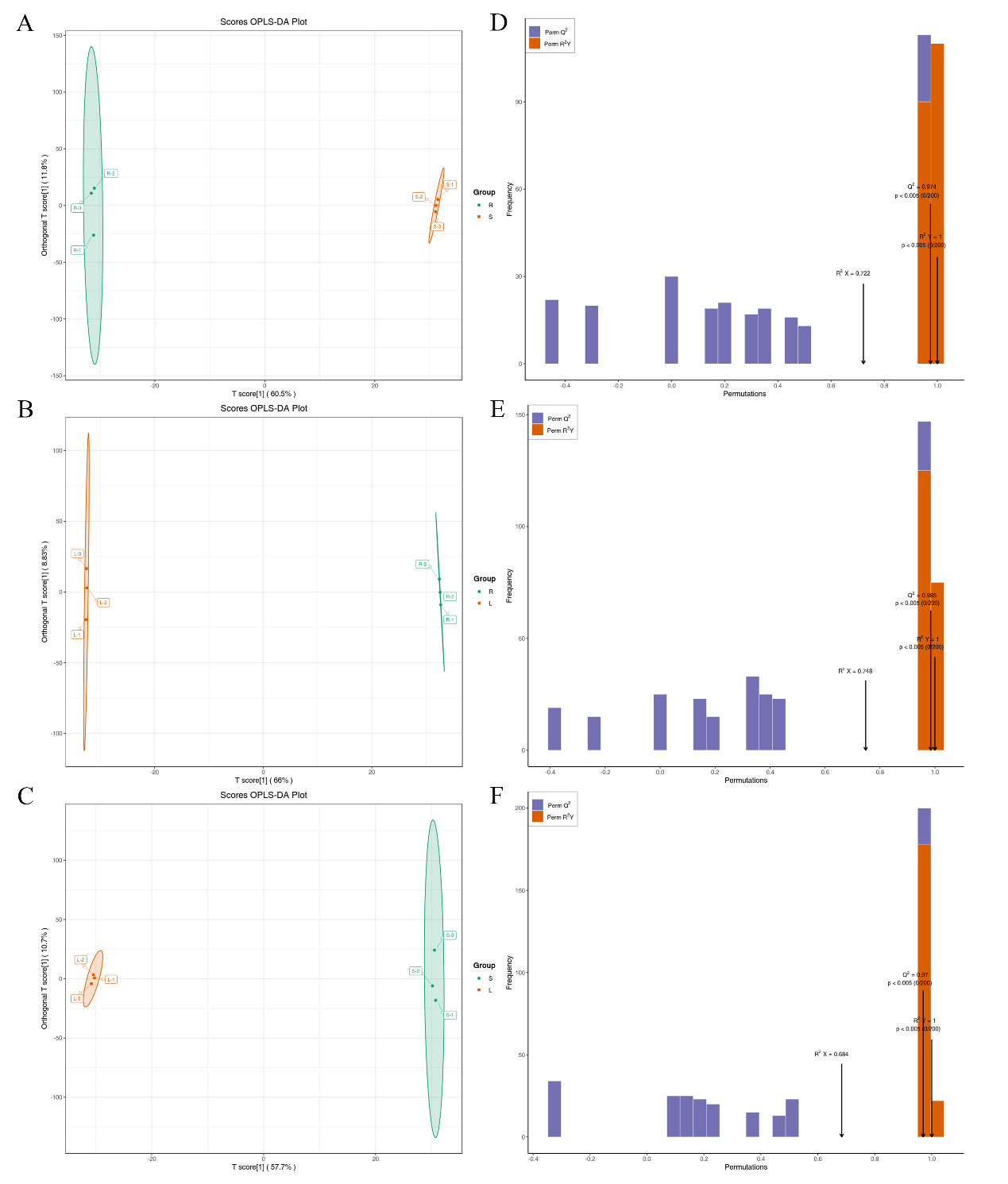


**Supplementary Figure S2.** OPLS-DA model. Score plots of the OPLS-DA model for R vs S **(A)**, R vs L **(B)**, and S vs L **(C)**. OPLS-DA permutation plot for R vs S **(D)**, R vs L **(E)**, and S vs L **(F)**.


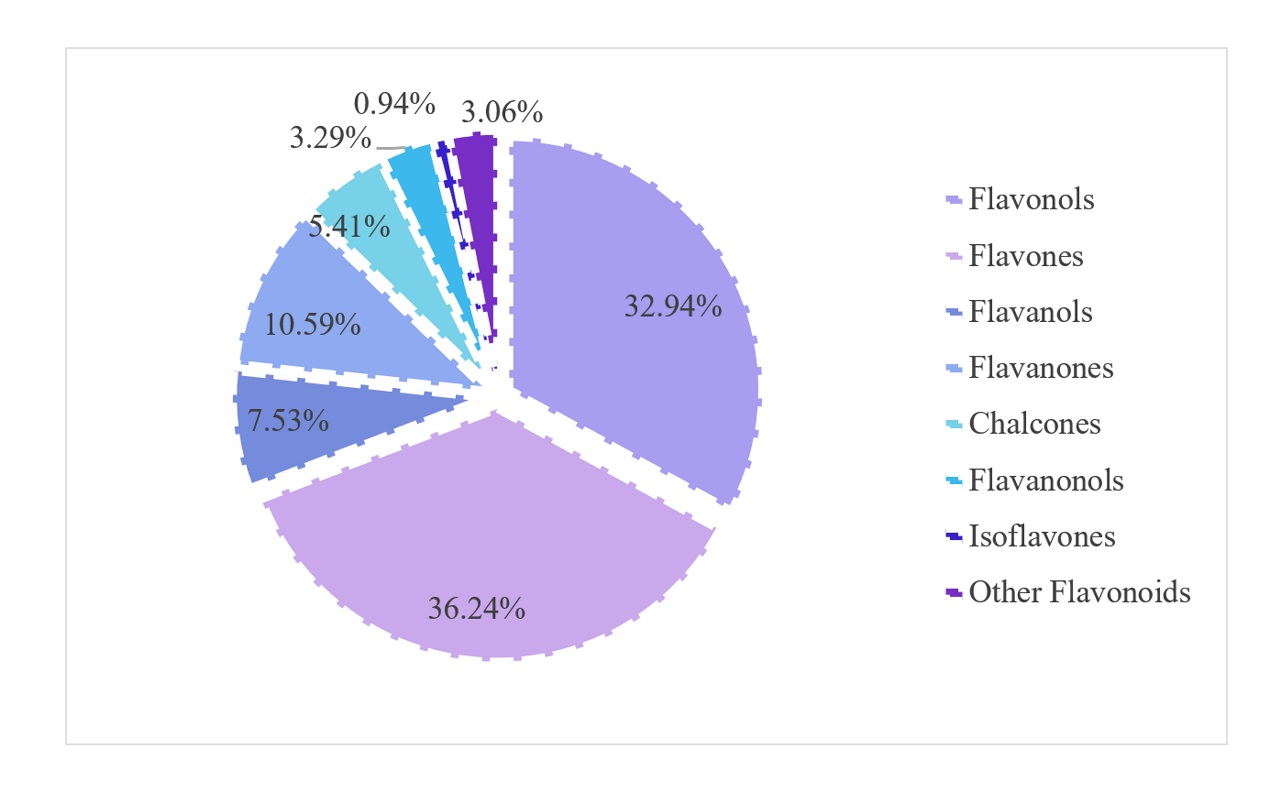


**Supplementary Figure S3.** The proportion of each component of flavonoids.


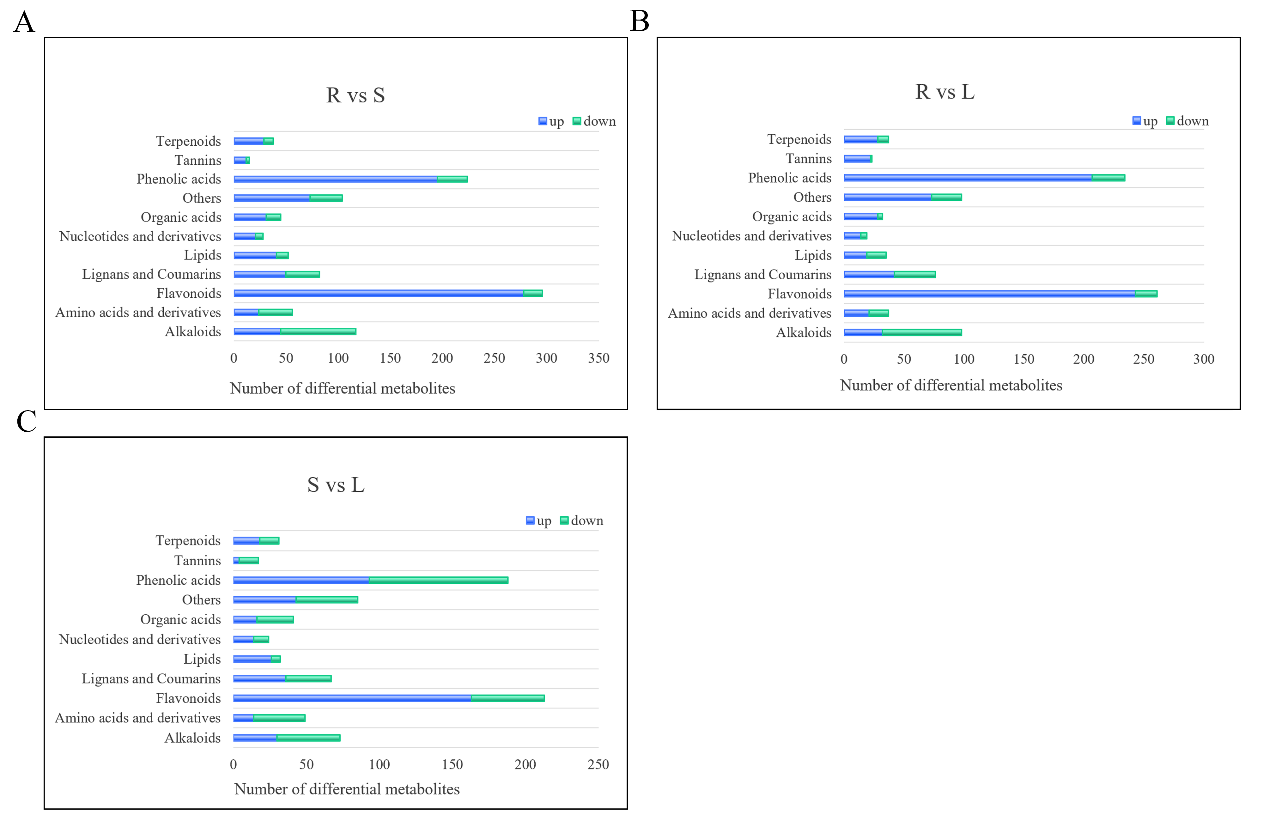


**Supplementary Figure S4.** Number of different types of differential metabolites for the comparison group R vs S **(A)**, R vs L **(B)**, S vs L **(C)**. Blue column indicates metabolites that were signiﬁcantly up-regulated; Green indicates metabolites that were signiﬁcantly down-regulated.


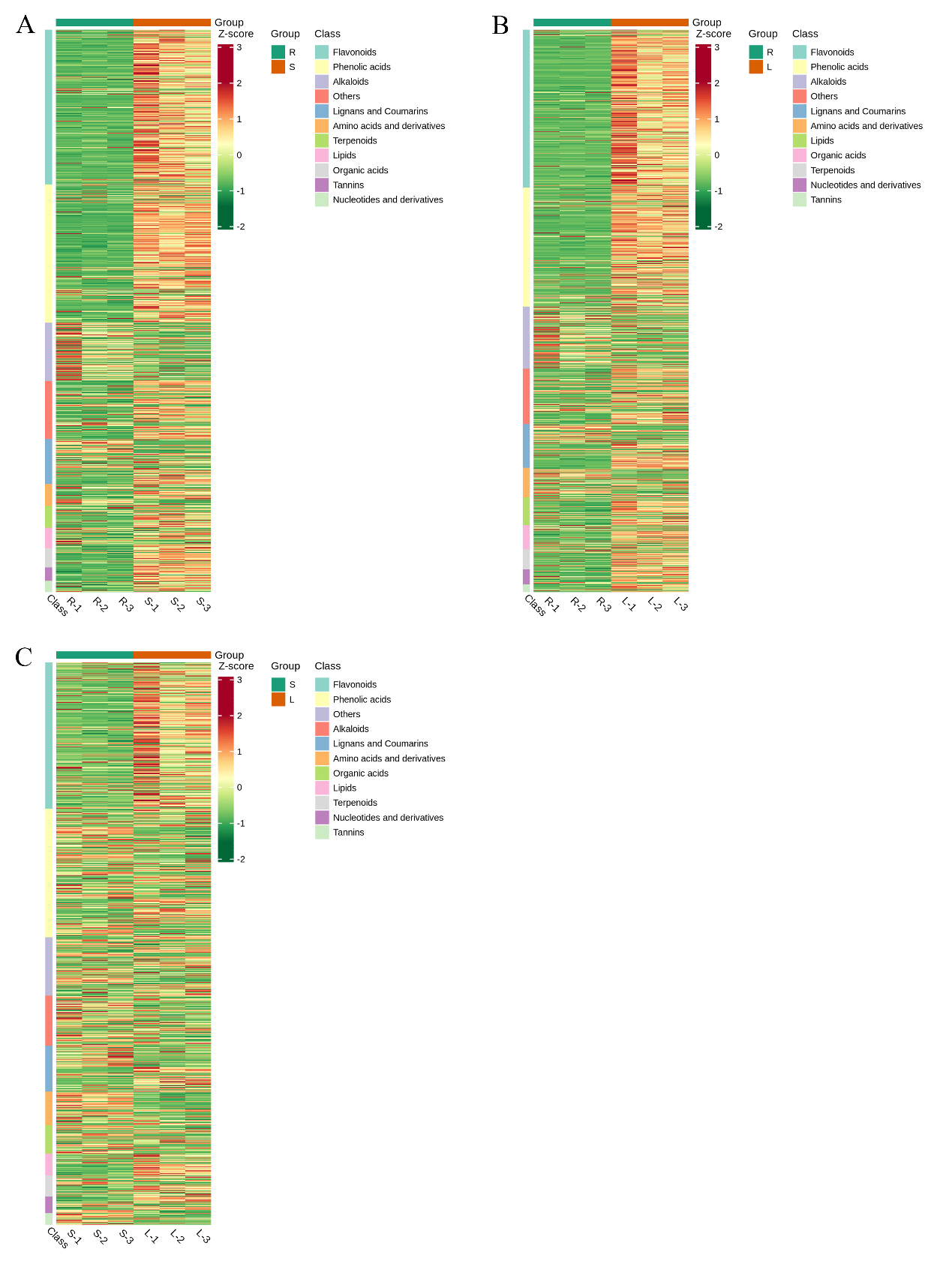


**Supplementary Figure S5.** Heat maps of differential accumulation of metabolites in R vs S **(A)**, R vs L **(B)**, and S vs L **(C)** comparison groups, respectively.


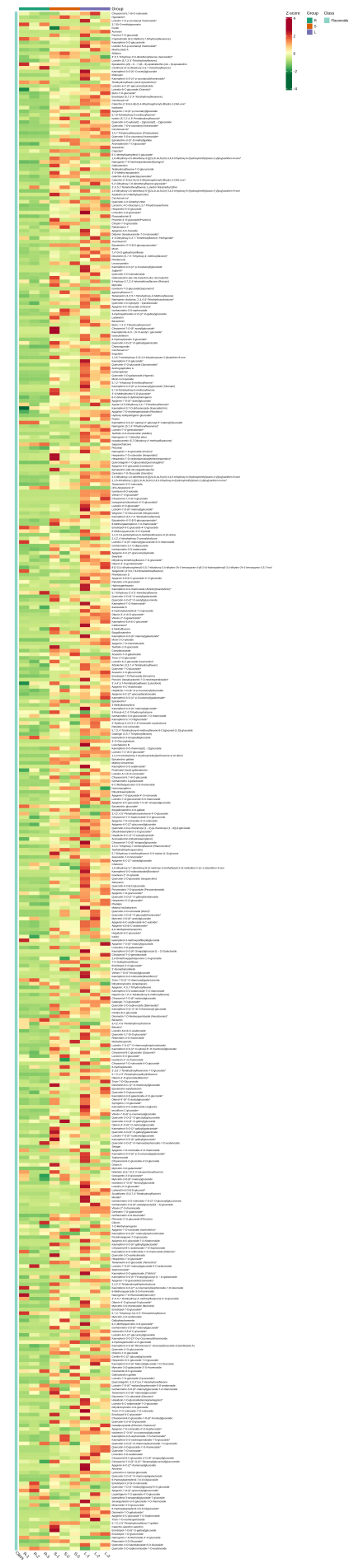


**Supplementary Figure S6.** Clustering heat map of all flavonoids. The values of metabolites were normalized and shown as a color scale. The high and low metabolite levels were represented as red and green scales.


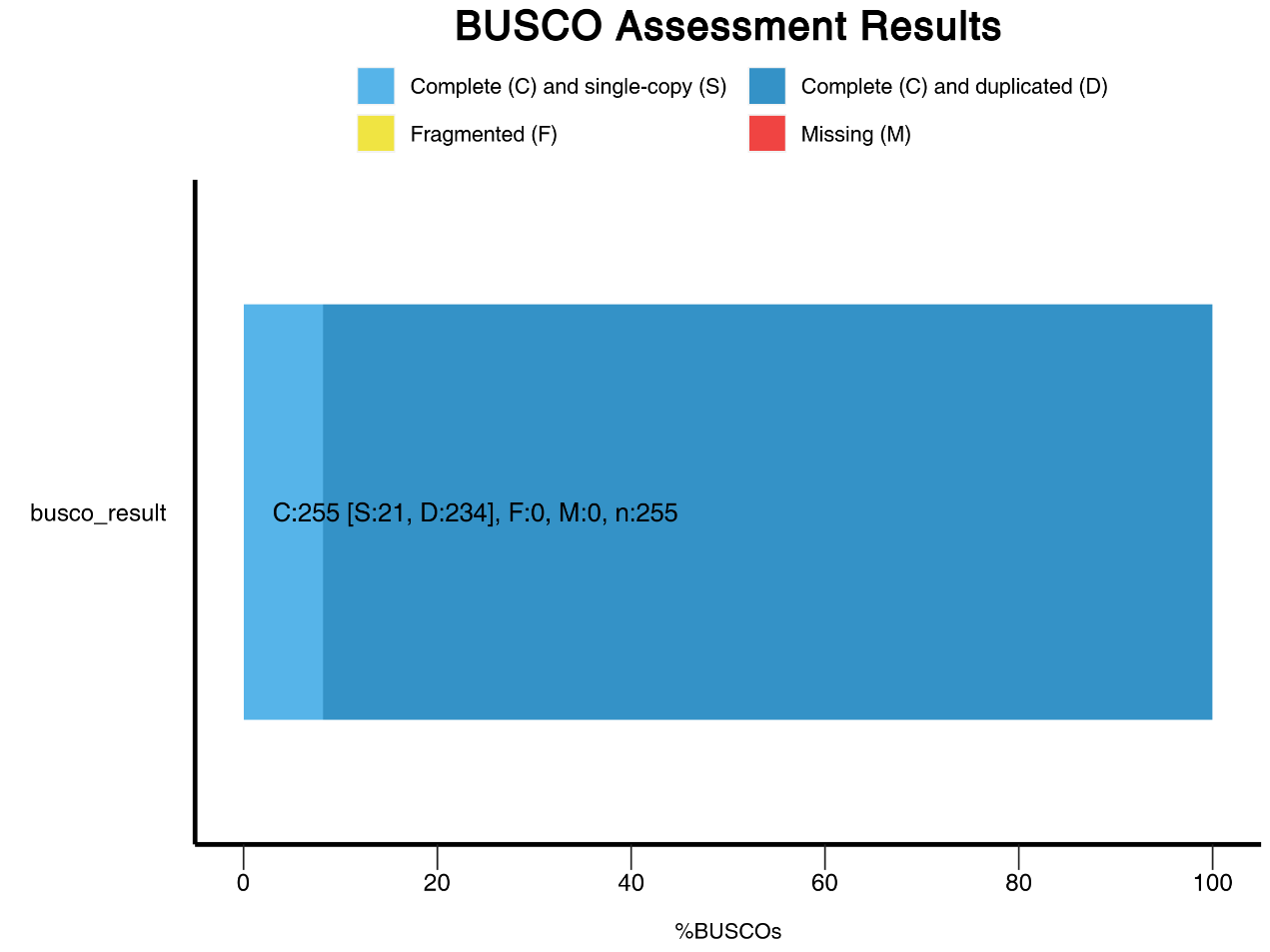


**Supplementary Figure S7.** USCO Assessment Results.


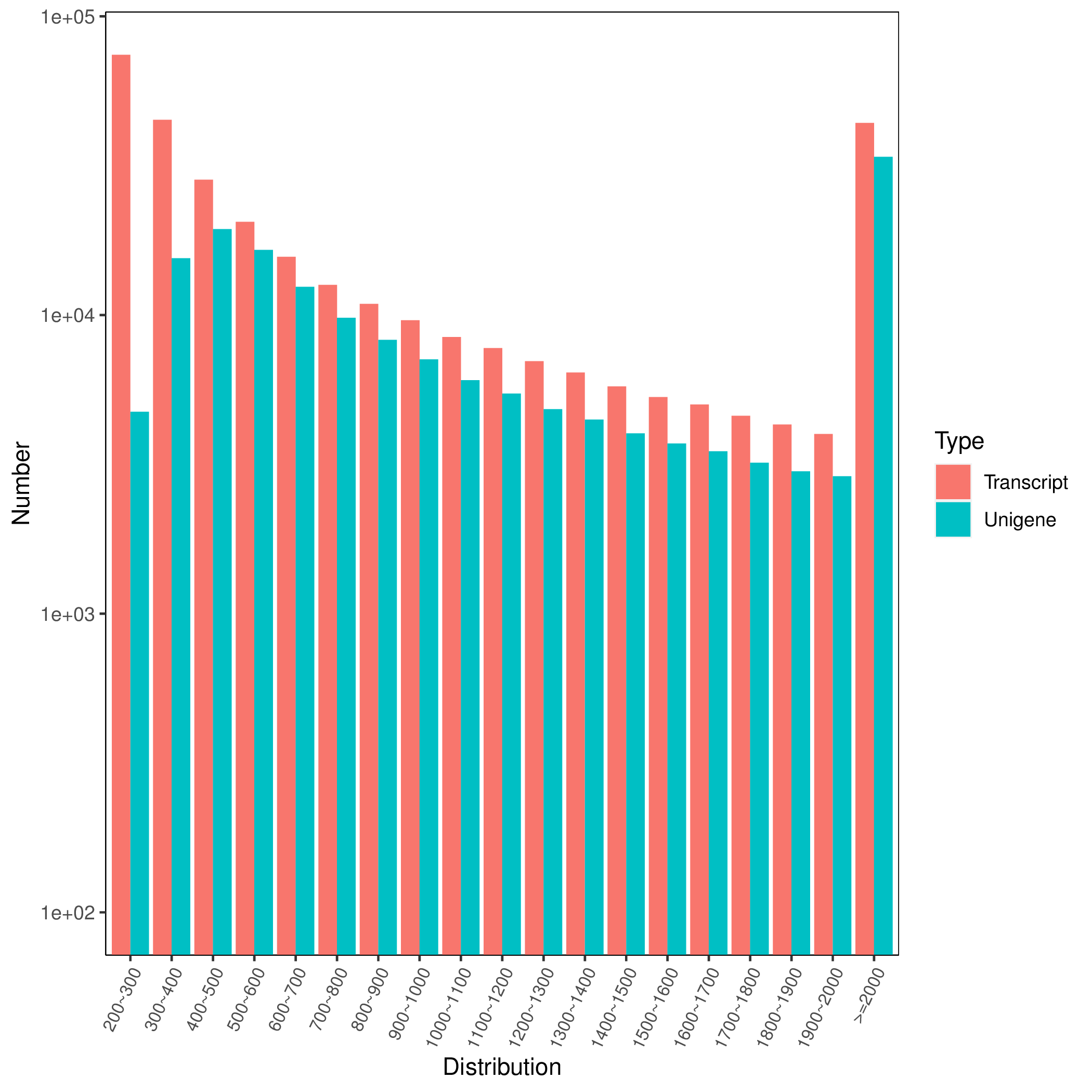


**Supplementary Figure S8.** Sequence length distribution.


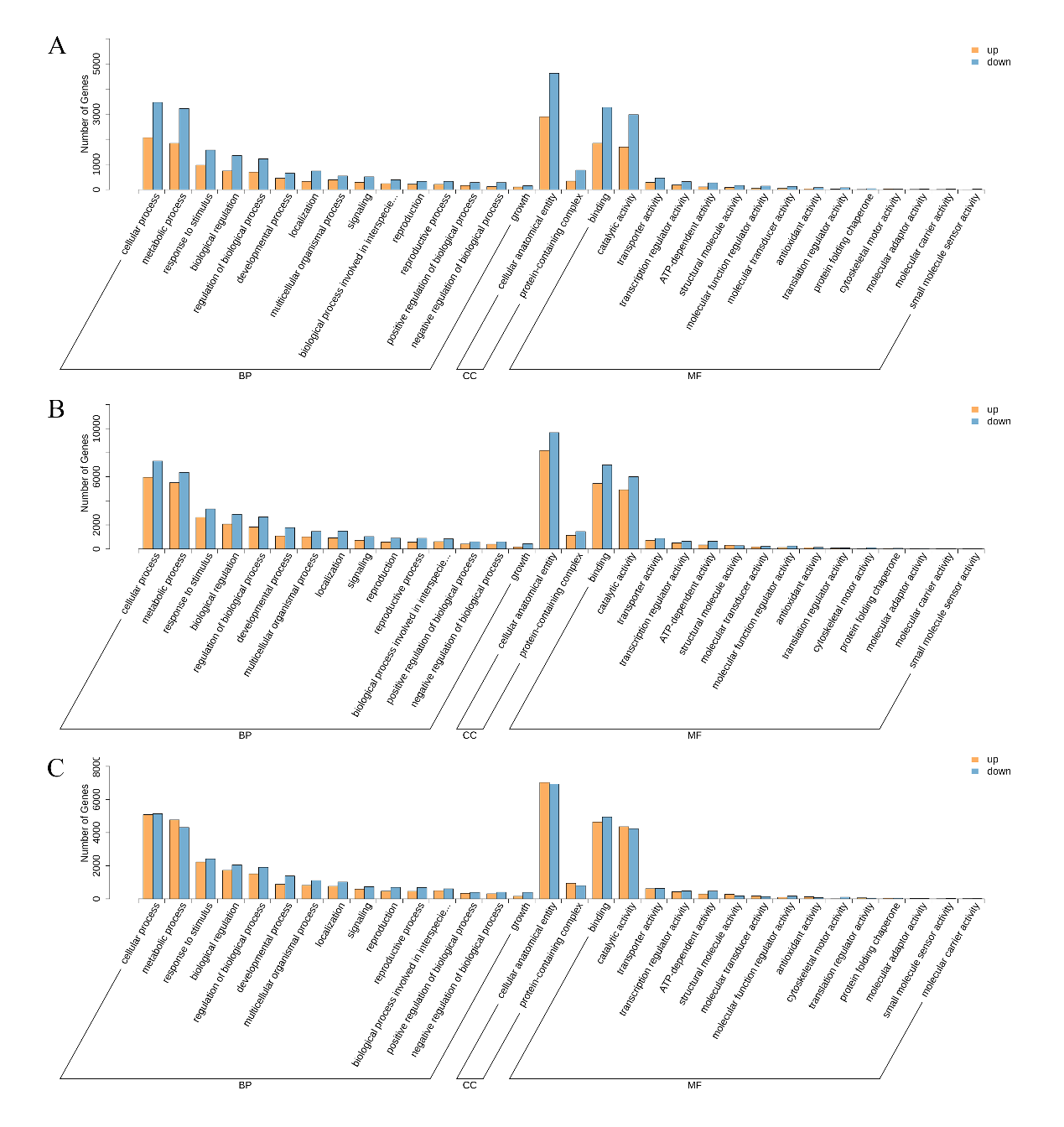


**Supplementary Figure S9.** GO classification of DEGs in the three comparison groups. R vs S **(A)**, R vs L **(B)**, S vs L **(C)**.


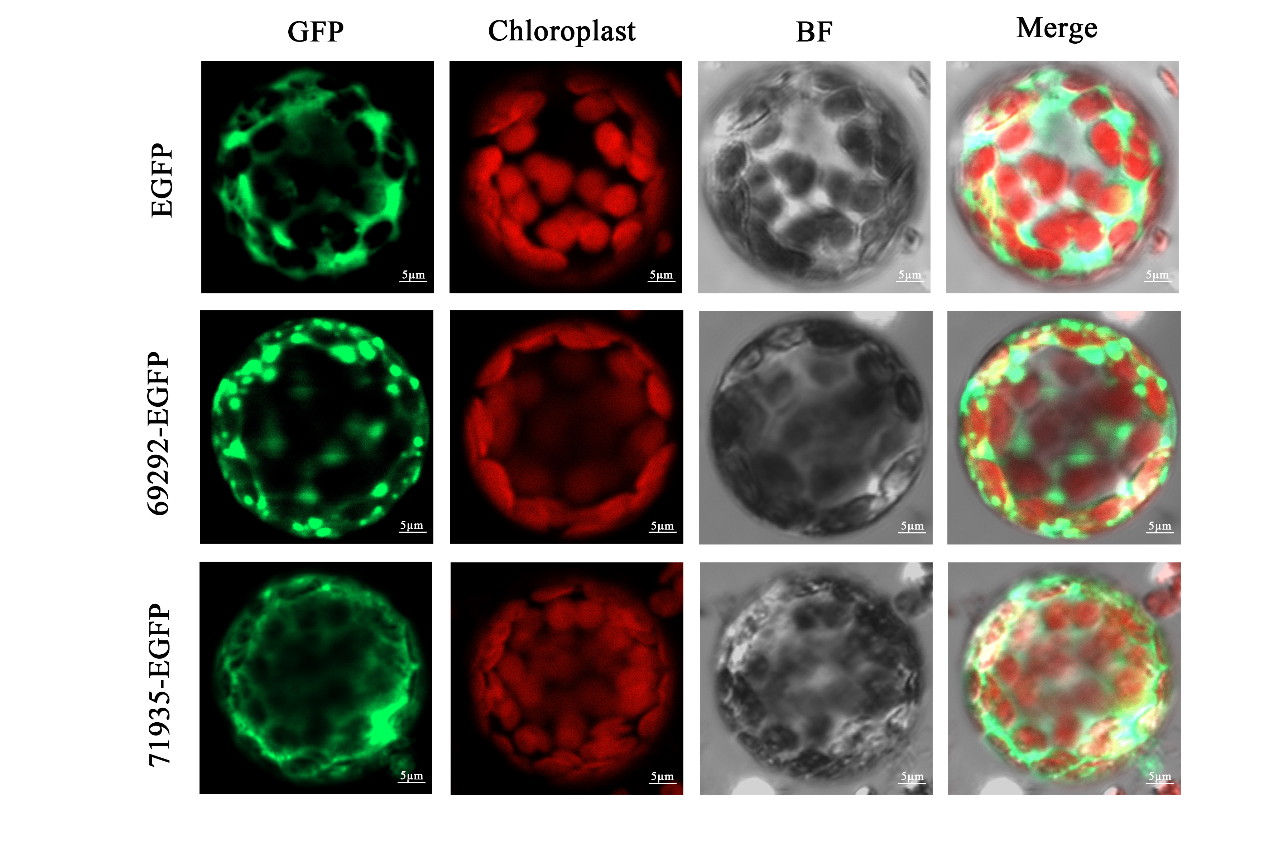


**Supplementary Figure S10.** Subcellular localization of Cluster-69292 and Cluster-71935 in tobacco (*N. benthamiana*) mesophyll protoplasts.


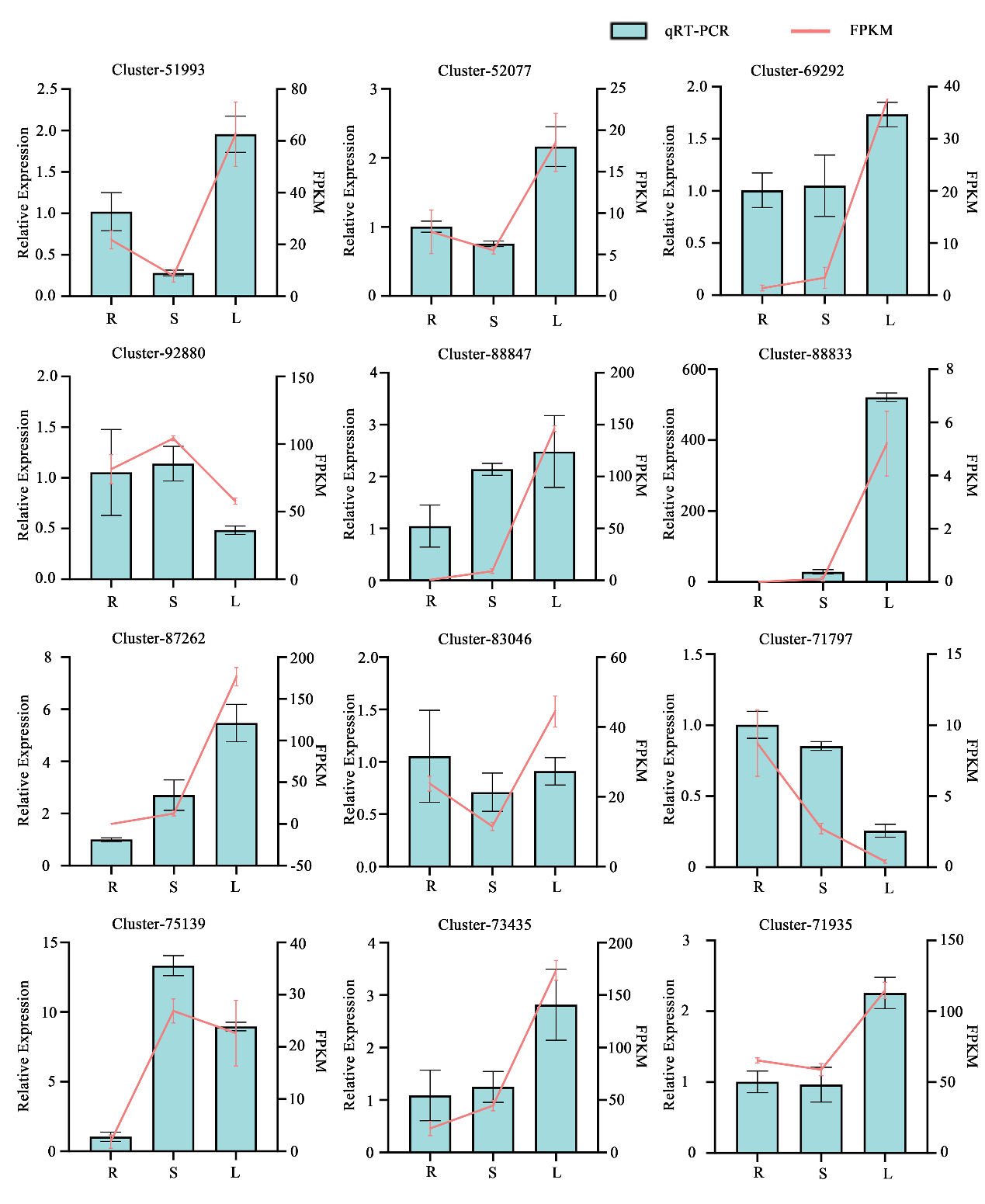


**Supplementary Figure S11.** The relative expression levels of twelve DEGs by qRT-PCR.

## Supplementary Tables

**Supplementary Table S1.** All primers used in qRT-PCR analysis.

| Gene ID | Sequence (5’-3’) |
| --- | --- |
| *Cluster-52077-F* | CCCGTTCTTGTGGGTCATCA |
| *Cluster-52077-R* | ATGCGTCACAAAACAACCCG |
| *Cluster-69292-F* | TCGGTTGGTGATGGTGAAGG |
| *Cluster-69292-R* | ACCGTCCGTTCTCTCAAACC |
| *Cluster-71935-F* | AAGGCCAGGGATTGGTGTTC |
| *Cluster-71935-R* | CGTACAAGGGCCAAGCCAAT |
| *Cluster-51993-F* | CGCTGACATACCCTCGTTCA |
| *Cluster-51993-R* | GATTCCACCAGCACCCAAGA |
| *Cluster-73435-F* | CGACAGTTGGTTTGCTGCTC |
| *Cluster-73435-R* | GTGTTCTTGACCCATTGCGG |
| *Cluster-83046-F* | TGGCTCAGTTCGAGACAACC |
| *Cluster-83046-R* | CTGATCCCATCTGGTCGGTG |
| *Cluster-87262-F* | ACAACCATCACAACAGCCGA |
| *Cluster-87262-R* | CAGTGCGTTAGGAAAACGGC |
| *Cluster-88847-F* | CAAGGCCACATCAACCCAAC |
| *Cluster-88847-R* | GGGCTGGGCTCGATTTCTAT |
| *Cluster-92880-F* | GGGGTATGTTAGTGGGCTGG |
| *Cluster-92880-R* | GCAGTTGGTTTGTTGCTCCC |
| *Cluster-71797-F* | AGGAACTGGTGTTGGCTTCA |
| *Cluster-71797-R* | GCCATTGCCAGAGTCAAGGA |
| *Cluster-88833-F* | CGTGGGAGAACCCTACTTCG |
| *Cluster-88833-R* | CACCATGCGAGAGGCTAGAG |
| *Cluster-75139-F* | GGGTGATCGCTTTGTTGCAT |
| *Cluster-75139-R* | GCCACTCCTAGACGTTGGAC |
| *ACTIN-F (KM086738.1)* | CCTCGACACACAGGCGTTAT |
| *ACTIN-R (KM086738.1)* | CCATGCTCGATGGGATATTTCA |

**Supplementary Table S2.** The metabolites information of *Machilus nanmu* with Different tissues.

| NO. | Compounds | Formula | Ionization model | Class I | NO. | Compounds | Formula | Ionization model | Class I |
| --- | --- | --- | --- | --- | --- | --- | --- | --- | --- |
| 1 | Spermidine | C7H19N3 | [M+H]+ | Alkaloids | 970 | LysoPC 18:1 | C26H52NO7P | [M+H]+ | Lipids |
| 2 | 5-Aminolevulinic Acid* | C5H9NO3 | [M+H]+ | Alkaloids | 971 | LysoPC 18:2(2n isomer) | C26H50NO7P | [M+H]+ | Lipids |
| 3 | 2-Amino-4,5-dihydro-1H-imidazole-4-acetic acid | C5H9N3O2 | [M+H]+ | Alkaloids | 972 | LysoPC(18:3(9Z,12Z,15Z)) | C26H48NO7P | [M+CH3COO]- | Lipids |
| 4 | Nicotianamine | C12H21N3O6 | [M+H]+ | Alkaloids | 973 | LysoPC 18:0(2n isomer) | C26H54NO7P | [M+H]+ | Lipids |
| 5 | 4-Methylazetidine-2-Carboxylic acid* | C5H9NO2 | [M+H]+ | Alkaloids | 974 | LysoPC 18:0 | C26H54NO7P | [M+H]+ | Lipids |
| 6 | Triethylamine | C6H15N | [M+H]+ | Alkaloids | 975 | LysoPC 17:0* | C25H52NO7P | [M+H]+ | Lipids |
| 7 | N-benzylformamide | C8H9NO | [M+H]+ | Alkaloids | 976 | LysoPC 19:2* | C27H52NO7P | [M+H]+ | Lipids |
| 8 | N-(4-oxopentyl)-acetamide | C7H13NO2 | [M+H]+ | Alkaloids | 977 | LysoPC 17:0(2n isomer)* | C25H52NO7P | [M+H]+ | Lipids |
| 9 | Histidinol | C6H11N3O | [M+H]+ | Alkaloids | 978 | LysoPC 16:1* | C24H48NO7P | [M+H]+ | Lipids |
| 10 | (3S,3'S)-N,N'-((1R,2R)-5-((3,4,5-trihydroxy-6-methyltetrahydro-2H-pyran-2-yl)oxy)cyclohex-3-ene-1,2-diyl)bis(2-methyl-2,3,4,9-tetrahydro-1H-pyrido[3,4-b]indole-3-carboxamide) | C38H46N6O7 | [M+H]+ | Alkaloids | 979 | LysoPC 19:2(2n isomer)* | C27H52NO7P | [M+H]+ | Lipids |
| 11 | 2-Ethyl-3-methylmaleimide-N-glucoside | C13H19NO7 | [M+H]+ | Alkaloids | 980 | LysoPC 17:2 | C25H48NO7P | [M+H]+ | Lipids |
| 12 | 4-Hydroxypipecolic acid* | C6H11NO3 | [M+H]+ | Alkaloids | 981 | LysoPC 16:1(2n isomer)* | C24H48NO7P | [M+H]+ | Lipids |
| 13 | N-Methylcolchamine | C22H27NO5 | [M+H]+ | Alkaloids | 982 | LysoPC 15:0(2n isomer)* | C23H48NO7P | [M+H]+ | Lipids |
| 14 | Betaine | C5H11NO2 | [M+H]+ | Alkaloids | 983 | LysoPC 20:2* | C28H54NO7P | [M+H]+ | Lipids |
| 15 | N-Benzylmethylene isomethylamine | C8H9N | [M+H]+ | Alkaloids | 984 | LysoPC 20:2(2n isomer)* | C28H54NO7P | [M+H]+ | Lipids |
| 16 | 10-Formyltetrahydrofolic Acid | C20H23N7O7 | [M+H]+ | Alkaloids | 985 | LysoPC 15:0* | C23H48NO7P | [M+H]+ | Lipids |
| 17 | Imidazole-4-Acetic Acid | C5H6N2O2 | [M+H]+ | Alkaloids | 986 | LysoPC 17:1 | C25H50NO7P | [M+H]+ | Lipids |
| 18 | Zarzissine | C5H5N5 | [M+H]+ | Alkaloids | 987 | LysoPC 14:0 | C22H46NO7P | [M+H]+ | Lipids |
| 19 | O-Phosphocholine | C5H15NO4P+ | [M]+ | Alkaloids | 988 | LysoPC 15:1 | C23H46NO7P | [M+H]+ | Lipids |
| 20 | Hercynine | C9H15N3O2 | [M+H]+ | Alkaloids | 989 | LysoPC 20:1 | C28H56NO7P | [M+H]+ | Lipids |
| 21 | L-Azetidine-2-carboxylic acid* | C4H7NO2 | [M+H]+ | Alkaloids | 990 | LysoPC 18:4 | C26H46NO7P | [M+H]+ | Lipids |
| 22 | Tetradecyldiethanolamine | C18H39NO2 | [M+H]+ | Alkaloids | 991 | LysoPC 20:3 | C28H52NO7P | [M+H]+ | Lipids |
| 23 | alanine betaine* | C5H11NO2 | [M+H]+ | Alkaloids | 992 | LysoPC 19:1 | C27H54NO7P | [M+H]+ | Lipids |
| 24 | (3S)-1,3-dihydroxy-2,3,3a,4-tetrahydropyrrolo[2,1-b]quinazolin-9(1H)-one | C11H12N2O3 | [M+H]+ | Alkaloids | 993 | LysoPC 12:0 | C20H42NO7P | [M+H]+ | Lipids |
| 25 | 3-amino-2-naphthoic acid* | C11H9NO2 | [M+H]+ | Alkaloids | 994 | LysoPE 18:2(2n isomer)* | C23H44NO7P | [M+H]+ | Lipids |
| 26 | Octadecadienamide | C18H33NO | [M+H]+ | Alkaloids | 995 | LysoPE 18:2* | C23H44NO7P | [M+H]+ | Lipids |
| 27 | (12S)-7,15,16,17-tetramethoxy-3,5-dioxa-11-azapentacyclo[10.7.1.02,6.08,20.014,19]icosa-1,6,8(20),14,16,18-hexaene | C21H23NO6 | [M+H]+ | Alkaloids | 996 | LysoPE 18:3(2n isomer)* | C23H42NO7P | [M+H]+ | Lipids |
| 28 | N,N-Dimethylformamide | C3H7NO | [M+H]+ | Alkaloids | 997 | LysoPE 16:0(2n isomer)* | C21H44NO7P | [M+H]+ | Lipids |
| 29 | 1-methoxy-6-methyl-10-propan-2-yloxy-5,6,6a,7-tetrahydro-4H-dibenzo[de,g]quinoline-2,9-diol* | C21H25NO4 | [M+H]+ | Alkaloids | 998 | LysoPE 16:0* | C21H44NO7P | [M+H]+ | Lipids |
| 30 | DL-2-Aminoadipic acid | C6H11NO4 | [M+H]+ | Alkaloids | 999 | LysoPE 18:3* | C23H42NO7P | [M+H]+ | Lipids |
| 31 | (E)-1-(3,4-dimethoxybenzylidene)-6,7-dimethoxy-2-methyl-1,2,3,4-tetrahydroisoquinoline* | C21H25NO4 | [M+H]+ | Alkaloids | 1000 | 2-(2,3-dihydroxypropoxy)-3-(((2-(dimethylamino)ethoxy)(hydroxy)phosphoryl)oxy)propyl (8E,11Z,14Z)-octadeca-8,11,14-trienoate* | C28H52NO9P | [M-H]- | Lipids |
| 32 | Hetidine | C21H27NO4 | [M+H]+ | Alkaloids | 1001 | 1-(2,3-dihydroxypropoxy)-3-(((2-(dimethylamino)ethoxy)(hydroxy)phosphoryl)oxy)propan-2-yl (8E,11Z,14Z)-octadeca-8,11,14-trienoate* | C28H52NO9P | [M-H]- | Lipids |
| 33 | Sarmentosin | C11H17NO7 | [M+H]+ | Alkaloids | 1002 | 2-(2,3-dihydroxypropoxy)-3-(((2-(dimethylamino)ethoxy)(hydroxy)phosphoryl)oxy)propyl (11Z,14Z)-octadeca-11,14-dienoate* | C28H54NO9P | [M-H]- | Lipids |
| 34 | L-Carnitine | C7H15NO3 | [M+H]+ | Alkaloids | 1003 | LysoPE 18:1* | C23H46NO7P | [M+H]+ | Lipids |
| 35 | Putrescine | C4H12N2 | [M+H]+ | Alkaloids | 1004 | 1-(2,3-dihydroxypropoxy)-3-(((2-(dimethylamino)ethoxy)(hydroxy)phosphoryl)oxy)propan-2-yl (11Z,14Z)-octadeca-11,14-dienoate* | C28H54NO9P | [M-H]- | Lipids |
| 36 | N-(4-Aminobutyl)benzamide | C11H16N2O | [M+H]+ | Alkaloids | 1005 | 1-(2,3-dihydroxypropoxy)-3-(((2-(dimethylamino)ethoxy)(hydroxy)phosphoryl)oxy)propan-2-yl palmitate | C26H54NO9P | [M-H]- | Lipids |
| 37 | Benzamide | C7H7NO | [M+H]+ | Alkaloids | 1006 | LysoPE 18:1(2n isomer)* | C23H46NO7P | [M+H]+ | Lipids |
| 38 | 4,5,6-Trihydroxy-2-cyclohexen-1-ylideneacetonitrile | C8H9NO3 | [M+H]+ | Alkaloids | 1007 | LysoPE 15:0(2n isomer)* | C20H42NO7P | [M+H]+ | Lipids |
| 39 | Acetylpyrazine | C6H6N2O | [M+H]+ | Alkaloids | 1008 | LysoPE 16:1* | C21H42NO7P | [M+H]+ | Lipids |
| 40 | (3S,3'S)-N,N'-((1R,2R)-4-((3,4,5-trihydroxy-6-methyltetrahydro-2H-pyran-2-yl)oxy)cyclohexane-1,2-diyl)bis(2-methyl-2,3,4,9-tetrahydro-1H-pyrido[3,4-b]indole-3-carboxamide) | C38H48N6O7 | [M+H]+ | Alkaloids | 1009 | LysoPE 15:0* | C20H42NO7P | [M+H]+ | Lipids |
| 41 | Hexadecanamide | C16H33NO | [M+H]+ | Alkaloids | 1010 | LysoPE 18:0 | C23H48NO7P | [M+H]+ | Lipids |
| 42 | 3,7,11-trimethyl-2-oxa-6,10,13-triazatricyclo[7.3.1.05,13]tridecane | C12H23N3O | [M+H]+ | Alkaloids | 1011 | LysoPE 16:1(2n isomer)* | C21H42NO7P | [M+H]+ | Lipids |
| 43 | Isorescinnamine, dihydro- | C35H44N2O9 | [M+H]+ | Alkaloids | 1012 | LysoPE 20:2(2n isomer) | C25H48NO7P | [M+H]+ | Lipids |
| 44 | Spermine | C10H26N4 | [M+H]+ | Alkaloids | 1013 | LysoPE 17:1(2n isomer) | C22H44NO7P | [M+H]+ | Lipids |
| 45 | 2,5-Dimethyl pyrazine | C6H8N2 | [M+H]+ | Alkaloids | 1014 | Choline Alfoscerate | C8H20NO6P | [M+H]+ | Lipids |
| 46 | O-Phosphorylethanolamine | C2H8NO4P | [M-H]- | Alkaloids | 1015 | 2-Aminohexadecane-1,4-diol* | C16H35NO2 | [M+H]+ | Lipids |
| 47 | 2-(Acetylamino)-3-phenyl-2-propenoic acid | C11H11NO3 | [M+H]+ | Alkaloids | 1016 | 4-Hydroxysphinganine; Phytosphingosine | C18H39NO3 | [M+H]+ | Lipids |
| 48 | Histamine | C5H9N3 | [M+H]+ | Alkaloids | 1017 | 2-AminoicoSane-1,5,7,19-tetraol | C20H43NO4 | [M+H]+ | Lipids |
| 49 | 11-[3,4,5-Trihydroxy-6-(hydroxymethyl)oxan-2-yl]oxy-1,6-diazatetracyclo[7.6.1.05,16.010,15]hexadeca-3,5(16),6,8,10,12,14-heptaen-2-one | C20H18N2O7 | [M+H]+ | Alkaloids | 1018 | 2-Aminohexadecane-1,15-diol* | C16H35NO2 | [M+H]+ | Lipids |
| 50 | Diethanolamine | C4H11NO2 | [M+H]+ | Alkaloids | 1019 | 2-Aminooctadecane-1,16,18,18-tetraol | C18H39NO4 | [M+H]+ | Lipids |
| 51 | 18-Demethylparaensidimerin C | C29H28N2O4 | [M+H]+ | Alkaloids | 1020 | 2-AminoicoSane-1,6,18,19,20-pentaol | C20H43NO5 | [M+H]+ | Lipids |
| 52 | N-Acetylcadaverine | C7H16N2O | [M+H]+ | Alkaloids | 1021 | 2-aminodocoSane-1,6,19,20,21-pentaol | C22H47NO5 | [M+H]+ | Lipids |
| 53 | 1-(4-((3,4,5-trihydroxy-6-(hydroxymethyl)tetrahydro-2H-pyran-2-yl)oxy)benzyl)-1,2,3,4-tetrahydroisoquinoline-5,6,7-triol | C22H27NO9 | [M+H]+ | Alkaloids | 1022 | 2-Aminohexadecane-1,5,15-triol | C16H35NO3 | [M+H]+ | Lipids |
| 54 | Pantetheine | C11H22N2O4S | [M-H]- | Alkaloids | 1023 | 2-Aminododecane-1,4-diol | C12H27NO2 | [M+H]+ | Lipids |
| 55 | Lumichrome | C12H10N4O2 | [M+H]+ | Alkaloids | 1024 | 2-Aminotetradecane-1,5,13-triol | C14H31NO3 | [M+H]+ | Lipids |
| 56 | Cadaverine | C5H14N2 | [M+H]+ | Alkaloids | 1025 | 2-aminodocoSane-1,5,7,21-tetraol | C22H47NO4 | [M+H]+ | Lipids |
| 57 | 2-(4-((6,7-dihydroxy-5-methoxy-1,2,3,4-tetrahydroisoquinolin-1-yl)methyl)phenoxy)-6-(hydroxymethyl)tetrahydro-2H-pyran-3,4,5-triol | C23H29NO9 | [M+H]+ | Alkaloids | 1026 | 2-Aminotetradecane-1,4-diol | C14H31NO2 | [M+H]+ | Lipids |
| 58 | Agmatine | C5H14N4 | [M+H]+ | Alkaloids | 1027 | 2-Aminotetradecan-1-ol* | C14H31NO | [M+H]+ | Lipids |
| 59 | Demethylcoclaurine* | C16H17NO3 | [M+H]+ | Alkaloids | 1028 | 2-Amino-7-methyltridecan-1-ol* | C14H31NO | [M+H]+ | Lipids |
| 60 | Huangjinjian | C19H21NO4 | [M+H]+ | Alkaloids | 1029 | Dihydrosphingosine | C18H39NO2 | [M+H]+ | Lipids |
| 61 | Prunasin | C14H17NO6 | [M-H]- | Alkaloids | 1030 | 3-Dehydrosphinganine | C18H37NO2 | [M+H]+ | Lipids |
| 62 | 1-Methylguanidine | C2H7N3 | [M+H]+ | Alkaloids | 1031 | 2-AminoicoSane-1,5,19-triol | C20H43NO3 | [M+H]+ | Lipids |
| 63 | 1-Methylhistamine | C6H11N3 | [M+H]+ | Alkaloids | 1032 | 2-Aminotetradecane-1,11,13-triol | C14H31NO3 | [M+H]+ | Lipids |
| 64 | N-Methyl-coclaurine | C18H21NO3 | [M+H]+ | Alkaloids | 1033 | 2-Aminooctadecane-1,5,7,17-tetraol | C18H39NO4 | [M+H]+ | Lipids |
| 65 | N-[7'-(4'-Methoxyphenyl)ethyl]-2-methoxybenzamide | C17H19NO3 | [M+H]+ | Alkaloids | 1034 | 2-Aminohexadecane-1,5,6-triol | C16H35NO3 | [M+H]+ | Lipids |
| 66 | 2(3H)-Benzothiazolone | C7H5NOS | [M-H]- | Alkaloids | 1035 | 2-aminotetracoSane-1,5,21,23-tetraol | C24H51NO4 | [M+H]+ | Lipids |
| 67 | 3-(1H-imidazol-5-yl)-2-(trimethylammonio)propanoate | C9H15N3O2 | [M+H]+ | Alkaloids | 1036 | Adenosine 5'-monophosphate | C10H14N5O7P | [M+H]+ | Nucleotides and derivatives |
| 68 | N-Acetylputrescine | C6H14N2O | [M+H]+ | Alkaloids | 1037 | 5'-Deoxy-5'-(methylthio)adenosine | C11H15N5O3S | [M+H]+ | Nucleotides and derivatives |
| 69 | 6-ethyl-1,10-dimethoxy-5,6,6a,7-tetrahydro-4H-dibenzo[de,g]quinoline-2,9-diol | C20H23NO4 | [M+H]+ | Alkaloids | 1038 | 2'-Deoxyinosine-5'-monophosphate | C10H13N4O7P | [M+H]+ | Nucleotides and derivatives |
| 70 | Caffeine | C8H10N4O2 | [M+H]+ | Alkaloids | 1039 | Succinyladenosine | C14H17N5O8 | [M+H]+ | Nucleotides and derivatives |
| 71 | Sambunigrin | C14H17NO6 | [M-H]- | Alkaloids | 1040 | 4-methyl-1,5,2,3-dioxadiazinan-2-amine | C3H9N3O2 | [M+H]+ | Nucleotides and derivatives |
| 72 | o-Carboxy-5-hydroxytryptamine | C11H12N2O3 | [M+H]+ | Alkaloids | 1041 | Nicotinate adenine dinucleotide phosphate | C21H27N6O18P3 | [M-H]- | Nucleotides and derivatives |
| 73 | 2-propenamide* | C19H21NO5 | [M+H]+ | Alkaloids | 1042 | 5-Aminoimidazole ribonucleotide | C8H14N3O7P | [M+H]+ | Nucleotides and derivatives |
| 74 | N-Isobutyl-4,5-epoxy-2E-decaenamide | C14H25NO2 | [M+H]+ | Alkaloids | 1043 | Barbituric acid;Malonylurea;2,4,6-Pyrimidinetrione | C4H4N2O3 | [M+H]+ | Nucleotides and derivatives |
| 75 | Guanidinoacetate | C3H7N3O2 | [M-H]- | Alkaloids | 1044 | 6-Chloropurine | C5H3ClN4 | [M+H]+ | Nucleotides and derivatives |
| 76 | (4,13-Dimethoxy-17-methyl-12-oxo-5-phenylmethoxy-17-azatetracyclo[7.5.3.01,10.02,7]heptadeca-2,4,6,10,13-pentaen-3-yl)acetate | C28H29NO6 | [M+H]+ | Alkaloids | 1045 | 2-Aminopurine | C5H5N5 | [M+H]+ | Nucleotides and derivatives |
| 77 | γ-Sanshool | C18H27NO | [M+H]+ | Alkaloids | 1046 | 9-Alpha-Ribofuranosyladenine* | C10H13N5O4 | [M+H]+ | Nucleotides and derivatives |
| 78 | Phenylethanolamine | C8H11NO | [M+H]+ | Alkaloids | 1047 | Inosine 5'-monophosphate | C10H13N4O8P | [M+H]+ | Nucleotides and derivatives |
| 79 | (R)-1,10-Dimethoxy-6-methyl-5,6,6a,7-tetrahydro-4H-dibenzo[de,g]quinoline-2,9-diol | C19H21NO4 | [M+H]+ | Alkaloids | 1048 | Vidarabine* | C10H13N5O4 | [M+H]+ | Nucleotides and derivatives |
| 80 | Caseadine* | C20H23NO4 | [M+H]+ | Alkaloids | 1049 | Isopentenyladenine-7-N-glucoside | C16H23N5O5 | [M+H]+ | Nucleotides and derivatives |
| 81 | Octadec-2-enamide | C18H35NO | [M+H]+ | Alkaloids | 1050 | Adenosine* | C10H13N5O4 | [M+H]+ | Nucleotides and derivatives |
| 82 | N-Oleoylethanolamine | C20H39NO2 | [M+H]+ | Alkaloids | 1051 | Crotonoside; 2-Hydroxyadenosine | C10H13N5O5 | [M+H]+ | Nucleotides and derivatives |
| 83 | 2-Glucosyloxy-2-phenylacetic acid amide | C14H19NO7 | [M-H]- | Alkaloids | 1052 | Ribosyladenosine | C15H21N5O8 | [M+H]+ | Nucleotides and derivatives |
| 84 | laudanine* | C20H25NO4 | [M+H]+ | Alkaloids | 1053 | Nicotinic acid adenine dinucleotide | C21H27N7O14P2 | [M+H]+ | Nucleotides and derivatives |
| 85 | Anthriscifoldine B | C25H39NO7 | [M+H]+ | Alkaloids | 1054 | Guanosine | C10H13N5O5 | [M+H]+ | Nucleotides and derivatives |
| 86 | 1,2,10-trimethoxy-6-methyl-5,6,6a,7-tetrahydro-4H-dibenzo[de,g]quinolin-11-ol | C20H23NO4 | [M+H]+ | Alkaloids | 1055 | Guanine | C5H5N5O | [M+H]+ | Nucleotides and derivatives |
| 87 | Dicentrine | C20H21NO4 | [M+H]+ | Alkaloids | 1056 | 2'-Deoxyadenosine-5'-monophosphate | C10H14N5O6P | [M+H]+ | Nucleotides and derivatives |
| 88 | Norisoboldine | C18H19NO4 | [M+H]+ | Alkaloids | 1057 | 2'-O-Methyladenosine | C11H15N5O4 | [M+H]+ | Nucleotides and derivatives |
| 89 | Laurotetanine | C19H21NO4 | [M+H]+ | Alkaloids | 1058 | Isoguanine | C5H5N5O | [M+H]+ | Nucleotides and derivatives |
| 90 | Anaxagoreine | C17H17NO3 | [M+H]+ | Alkaloids | 1059 | Flavin Single Nucleotide(FMN) | C17H21N4O9P | [M-H]- | Nucleotides and derivatives |
| 91 | Lauroscholtzine; N-Methyllaurotetanine | C20H23NO4 | [M+H]+ | Alkaloids | 1060 | Uridine 5'-monophosphate | C9H13N2O9P | [M-H]- | Nucleotides and derivatives |
| 92 | 2-Phenylethylamine | C8H11N | [M+H]+ | Alkaloids | 1061 | 1-Methyladenine | C6H7N5 | [M+H]+ | Nucleotides and derivatives |
| 93 | 6-[(7-hydroxy-2-methyl-6-oxo-3,4-dihydroisoquinolin-1-yl)methyl]-2,3-dimethoxybenzoic acid | C20H21NO6 | [M+H]+ | Alkaloids | 1062 | 2-Deoxyribose-1-phosphate* | C5H11O7P | [M-H]- | Nucleotides and derivatives |
| 94 | Stepharine | C18H19NO3 | [M+H]+ | Alkaloids | 1063 | Cytidine | C9H13N3O5 | [M+H]+ | Nucleotides and derivatives |
| 95 | 3',6-Dihydroxy-4',7-dimethoxyl-N,N-dimethyltetrahydroisoquinoline* | C20H25NO4 | [M+H]+ | Alkaloids | 1064 | Guanosine 5'-monophosphate | C10H14N5O8P | [M+H]+ | Nucleotides and derivatives |
| 96 | Isoquinoline | C9H7N | [M+H]+ | Alkaloids | 1065 | AICAR phosphate (Acadesine phosphate) | C9H17N4O9P | [M-H]- | Nucleotides and derivatives |
| 97 | 1-[(4-hydroxyphenyl)methyl]-7-methoxy-1,2,3,4-tetrahydroisoquinolin-8-ol | C17H19NO3 | [M+H]+ | Alkaloids | 1066 | Xanthosine | C10H12N4O6 | [M-H]- | Nucleotides and derivatives |
| 98 | 1-[(4-methoxyphenyl)methyl]-1,2,3,4-tetrahydroisoquinoline-6,7-diol* | C17H19NO3 | [M+H]+ | Alkaloids | 1067 | Cytidine 5'-monophosphate(Cytidylic acid) | C9H14N3O8P | [M+H]+ | Nucleotides and derivatives |
| 99 | Coclaurine* | C17H19NO3 | [M+H]+ | Alkaloids | 1068 | β-Pseudouridine | C9H12N2O6 | [M-H]- | Nucleotides and derivatives |
| 100 | 2-methyl-1betah-coclaurine | C18H21NO3 | [M+H]+ | Alkaloids | 1069 | 3-Methylxanthine | C6H6N4O2 | [M+H]+ | Nucleotides and derivatives |
| 101 | 1-(4-hydroxyphenyl)-7-methoxy-1,2,3,4-tetrahydroisoquinolin-8-ol* | C16H17NO3 | [M+H]+ | Alkaloids | 1070 | 9-(Arabinosyl)hypoxanthine | C10H12N4O5 | [M-H]- | Nucleotides and derivatives |
| 102 | Higenamine* | C16H17NO3 | [M+H]+ | Alkaloids | 1071 | 1-beta-D-Arabinofuranosyluracil | C9H12N2O6 | [M+H]+ | Nucleotides and derivatives |
| 103 | 1-[(4-hydroxyphenyl)methyl]-6-methoxy-2-methyl-3,4-dihydro-1h-isoquinolin-7-ol | C18H21NO3 | [M+H]+ | Alkaloids | 1072 | N-(1-Deoxy-1-fructosyl)Tryptophan | C17H22N2O7 | [M-H]- | Nucleotides and derivatives |
| 104 | (1s)-7-methoxy-1-[(4-methoxyphenyl)methyl]-2-methyl-3,4-dihydro-1h-isoquinolin-6-ol | C19H23NO3 | [M+H]+ | Alkaloids | 1073 | Uracil | C4H4N2O2 | [M-H]- | Nucleotides and derivatives |
| 105 | (S)-2,10,11-trimethoxy-6-methyl-5,6,6a,7-tetrahydro-4H-dibenzo[de,g]quinolin-1-ol* | C20H23NO4 | [M+H]+ | Alkaloids | 1074 | 2-(Dimethylamino)guanosine* | C12H17N5O5 | [M+H]+ | Nucleotides and derivatives |
| 106 | 6'-hydroxy-3,5'-dimethoxy-1'-methyl-2',3',8',8a'-tetrahydro-1'H-spiro[cyclohexane-1,7'-cyclopenta[ij]isoquinolin]-2-en-4-one | C19H23NO4 | [M+H]+ | Alkaloids | 1075 | 8-Azaguanine | C4H4N6O | [M+H]+ | Nucleotides and derivatives |
| 107 | 6-Acetylmorphine | C19H21NO4 | [M+H]+ | Alkaloids | 1076 | Allopurinol | C5H4N4O | [M+H]+ | Nucleotides and derivatives |
| 108 | N-(2-Hydroxy-4-methoxyphenyl)acetamide | C9H11NO3 | [M+H]+ | Alkaloids | 1077 | 6-O-methylguanine | C6H7N5O | [M+H]+ | Nucleotides and derivatives |
| 109 | N-Monocinnamoylputrescine* | C13H18N2O | [M+H]+ | Alkaloids | 1078 | β-Nicotinamide mononucleotide | C11H15N2O8P | [M+H]+ | Nucleotides and derivatives |
| 110 | N,N-cinnamoylbutanediamine* | C13H18N2O | [M+H]+ | Alkaloids | 1079 | 7-Methylxanthine | C6H6N4O2 | [M+H]+ | Nucleotides and derivatives |
| 111 | N-(gamma-L-glutamyl)tyramine O-glucoside | C19H28N2O9 | [M+H]+ | Alkaloids | 1080 | N6-(2-Hydroxyethyl)adenosine* | C12H17N5O5 | [M+H]+ | Nucleotides and derivatives |
| 112 | p-Coumaroylferuloylcadaverine | C24H28N2O5 | [M+H]+ | Alkaloids | 1081 | 2'-Deoxyadenosine* | C10H13N5O3 | [M+H]+ | Nucleotides and derivatives |
| 113 | Candicine | C11H18NO+ | [M]+ | Alkaloids | 1082 | Uridine 5'-diphosphate | C9H14N2O12P2 | [M-H]- | Nucleotides and derivatives |
| 114 | Caffeoylcholine | C14H20NO4+ | [M]+ | Alkaloids | 1083 | Uridine | C9H12N2O6 | [M-H]- | Nucleotides and derivatives |
| 115 | N',N'',N'''-p-Coumaroyl-cinnamoyl-caffeoyl spermidine | C34H37N3O6 | [M+H]+ | Alkaloids | 1084 | 2-Deoxyribose-5'-phosphate* | C5H11O7P | [M-H]- | Nucleotides and derivatives |
| 116 | N,N'-Diferuloylputrescine | C24H28N2O6 | [M+H]+ | Alkaloids | 1085 | 5-Methylcytosine | C5H7N3O | [M+H]+ | Nucleotides and derivatives |
| 117 | N-Feruloylagmatine | C15H22N4O3 | [M+H]+ | Alkaloids | 1086 | Hypoxanthine | C5H4N4O | [M+H]+ | Nucleotides and derivatives |
| 118 | Feruloylspermidine | C17H27N3O3 | [M+H]+ | Alkaloids | 1087 | Cordycepin (3'-Deoxyadenosine)* | C10H13N5O3 | [M+H]+ | Nucleotides and derivatives |
| 119 | Sinapine | C16H24NO5+ | [M]+ | Alkaloids | 1088 | N6-methyladenosine | C11H15N5O4 | [M+H]+ | Nucleotides and derivatives |
| 120 | 3-Hydroxyanthranilic acid | C7H7NO3 | [M+H]+ | Alkaloids | 1089 | 2'-Deoxycytidine | C9H13N3O4 | [M+H]+ | Nucleotides and derivatives |
| 121 | 4-Hydroxymandelonitrile | C8H7NO2 | [M+H]+ | Alkaloids | 1090 | Xanthine | C5H4N4O2 | [M-H]- | Nucleotides and derivatives |
| 122 | Feruloylcholine | C15H22NO4+ | [M]+ | Alkaloids | 1091 | 5-Methyl-2'-Deoxycytidine | C10H15N3O4 | [M+H]+ | Nucleotides and derivatives |
| 123 | N-Trans-Sinapoyltyramine | C19H21NO5 | [M+H]+ | Alkaloids | 1092 | 1-Methyladenosine | C11H15N5O4 | [M-H]- | Nucleotides and derivatives |
| 124 | Salicylamide | C7H7NO2 | [M+H]+ | Alkaloids | 1093 | N6-Isopentenyladenine | C10H13N5 | [M+H]+ | Nucleotides and derivatives |
| 125 | 1-(Dihydroxyphenyl)-N2,N3-bis(4-hydroxyphenethyl)-(5-8)-dimethoxy-1,2dihydronaphthalene-2,3-dicarboxamide | C36H36N2O8 | [M+H]+ | Alkaloids | 1094 | Cytosine | C4H5N3O | [M+H]+ | Nucleotides and derivatives |
| 126 | Dopamine | C8H11NO2 | [M+H]+ | Alkaloids | 1095 | 1-Methylxanthine | C6H6N4O2 | [M+H]+ | Nucleotides and derivatives |
| 127 | Caffeoylcholine-3-O-glucoside* | C20H30NO9+ | [M]+ | Alkaloids | 1096 | 1,7-Dimethylxanthine | C7H8N4O2 | [M+H]+ | Nucleotides and derivatives |
| 128 | N-Feruloyltyramine; Moupinamide | C18H19NO4 | [M+H]+ | Alkaloids | 1097 | 5,6-Dihydro-5-methyluracil | C5H8N2O2 | [M+H]+ | Nucleotides and derivatives |
| 129 | p-Coumaroylagmatine | C14H20N4O2 | [M+H]+ | Alkaloids | 1098 | Citicoline | C14H26N4O11P2 | [M+H]+ | Nucleotides and derivatives |
| 130 | p-Coumaroylputrescine | C13H18N2O2 | [M+H]+ | Alkaloids | 1099 | Adenosine 5'-diphosphate | C10H15N5O10P2 | [M-H]- | Nucleotides and derivatives |
| 131 | Caffeoylcholine-4-O-glucoside* | C20H30NO9+ | [M]+ | Alkaloids | 1100 | Cyclic 3',5'-Adenylic acid | C10H12N5O6P | [M-H]- | Nucleotides and derivatives |
| 132 | Sinapoylputrescine | C15H22N2O4 | [M+H]+ | Alkaloids | 1101 | 5'-Deoxyadenosine* | C10H13N5O3 | [M+H]+ | Nucleotides and derivatives |
| 133 | p-Coumaroylcadaverine | C14H20N2O2 | [M+H]+ | Alkaloids | 1102 | Uridine 5'-diphospho-D-glucose | C15H24N2O17P2 | [M-H]- | Nucleotides and derivatives |
| 134 | 2-(D-Glucosyloxy)-4-hydroxybenzeneacetonitrile | C14H17NO7 | [M-H]- | Alkaloids | 1103 | Riboprine | C15H21N5O4 | [M+H]+ | Nucleotides and derivatives |
| 135 | Grossamide | C36H36N2O8 | [M+H]+ | Alkaloids | 1104 | Isocitric Acid | C6H8O7 | [M-H]- | Organic acids |
| 136 | N1,N10-Bis(p-coumaroyl)spermidine | C25H31N3O4 | [M+H]+ | Alkaloids | 1105 | 5-Hydroxymethyl-2-furancarboxylic acid | C6H6O4 | [M-H]- | Organic acids |
| 137 | Sinapoylagmatine | C16H24N4O4 | [M+H]+ | Alkaloids | 1106 | 2-Keto-3-deoxygluconate | C6H10O6 | [M+CH3COO]- | Organic acids |
| 138 | N-Feruloylputrescine | C14H20N2O3 | [M+H]+ | Alkaloids | 1107 | Methylmalonic acid | C4H6O4 | [M-H]- | Organic acids |
| 139 | Vanillylamine | C8H11NO2 | [M+H]+ | Alkaloids | 1108 | L-Pipecolic Acid* | C6H11NO2 | [M+H]+ | Organic acids |
| 140 | N',N''-Diferuloylspermidine | C27H35N3O6 | [M+H]+ | Alkaloids | 1109 | Shikimic acid | C7H10O5 | [M-H]- | Organic acids |
| 141 | N-Feruloyl-3-methoxytyramine* | C19H21NO5 | [M+H]+ | Alkaloids | 1110 | Glucosyl 2,3-Dihydroxy-2-Methylbutanoic Acid | C11H20O9 | [M-H]- | Organic acids |
| 142 | N1-Caffeoyl-N10-Feruloylspermidine | C26H33N3O6 | [M+H]+ | Alkaloids | 1111 | Muconic acid | C6H6O4 | [M-H]- | Organic acids |
| 143 | N-Trans-Feruloyl-3'-O-methyldopamine* | C19H21NO5 | [M+H]+ | Alkaloids | 1112 | 2-Aminoisobutyric acid* | C4H9NO2 | [M+H]+ | Organic acids |
| 144 | Hordenine | C10H15NO | [M+H]+ | Alkaloids | 1113 | Quinic Acid | C7H12O6 | [M-H]- | Organic acids |
| 145 | Feruloylhistamine | C15H17N3O3 | [M+H]+ | Alkaloids | 1114 | trans-Aconitic acid | C6H6O6 | [M+H]+ | Organic acids |
| 146 | Caffeoyl-dihydrocaffeoyl spermidine | C25H33N3O6 | [M+H]+ | Alkaloids | 1115 | α-Ketoglutaric acid | C5H6O5 | [M-H]- | Organic acids |
| 147 | N1,N10-Dihydrocaffeoylspermidine | C25H35O6N3 | [M+H]+ | Alkaloids | 1116 | Keto-Deoxy-Nonulonic acid | C9H16O9 | [M-H]- | Organic acids |
| 148 | N1-Dihydrocaffeoyl-N10-coumaroylspermidine | C25H33N3O5 | [M+H]+ | Alkaloids | 1117 | Methanesulfonic acid | CH4O3S | [M-H]- | Organic acids |
| 149 | 4-Hydroxy-3-methoxy-β-phenethylamine | C9H13NO2 | [M+H]+ | Alkaloids | 1118 | 3-(2,5-dimethoxyphenyl)propanoic acid | C11H14O4 | [M-H]- | Organic acids |
| 150 | Tricaffeoylspermine | C37H44N4O9 | [M+H]+ | Alkaloids | 1119 | Fumaric acid | C4H4O4 | [M-H]- | Organic acids |
| 151 | Dobutamine | C18H23NO3 | [M+H]+ | Alkaloids | 1120 | 2-Amino-5-oxohexanoic acid* | C6H11NO3 | [M+H]+ | Organic acids |
| 152 | 6-Deoxyfagomine | C6H13NO2 | [M+H]+ | Alkaloids | 1121 | Malonic acid | C3H4O4 | [M-H]- | Organic acids |
| 153 | Fagomine | C6H13NO3 | [M+H]+ | Alkaloids | 1122 | 3-Dehydroshikimic acid | C7H8O5 | [M-H]- | Organic acids |
| 154 | Piperidine | C5H11N | [M+H]+ | Alkaloids | 1123 | DL-Glyceraldehyde-3-phosphate | C3H7O6P | [M-H]- | Organic acids |
| 155 | 3β-Isodihydrocadambine | C27H34N2O10 | [M+H]+ | Alkaloids | 1124 | 4-Guanidinobutyric acid | C5H11N3O2 | [M+H]+ | Organic acids |
| 156 | Hypaphorine | C14H18N2O2 | [M+H]+ | Alkaloids | 1125 | Urocanic acid | C6H6N2O2 | [M-H]- | Organic acids |
| 157 | Indole | C8H7N | [M+H]+ | Alkaloids | 1126 | 6-Acetamidohexanoic acid | C8H15NO3 | [M+H]+ | Organic acids |
| 158 | N-Acetylisatin | C10H7NO3 | [M-H]- | Alkaloids | 1127 | Citric Acid | C6H8O7 | [M-H]- | Organic acids |
| 159 | Methyl dioxindole-3-acetate | C11H11NO4 | [M+H]+ | Alkaloids | 1128 | Oroxylin A-7-o-beta-D-glucuronide | C22H20O11 | [M+CH3COO]- | Organic acids |
| 160 | 3-Indoleacrylic acid* | C11H9NO2 | [M+H]+ | Alkaloids | 1129 | 2,6-Diaminooimelic acid | C7H14N2O4 | [M+H]+ | Organic acids |
| 161 | N,N-Dimethyl-5-methoxytryptamine | C13H18N2O | [M+H]+ | Alkaloids | 1130 | Tuberonic acid glucoside* | C18H28O9 | [M-H]- | Organic acids |
| 162 | 1-Methoxy-indole-3-acetamide | C11H12N2O2 | [M+H]+ | Alkaloids | 1131 | Azelaic acid | C9H16O4 | [M-H]- | Organic acids |
| 163 | N-(p-Coumaroyl)serotonin Glucoside | C25H28N2O8 | [M+H]+ | Alkaloids | 1132 | L-Tartaric acid | C4H6O6 | [M-H]- | Organic acids |
| 164 | 3-Indole acetamide | C10H10N2O | [M+H]+ | Alkaloids | 1133 | D-Malic acid* | C4H6O5 | [M-H]- | Organic acids |
| 165 | Dioxindole-3-acetyl-3-O-glucoside | C16H19NO9 | [M-H]- | Alkaloids | 1134 | 2-Methylsuccinic acid | C5H8O4 | [M-H]- | Organic acids |
| 166 | Tryptamine | C10H12N2 | [M+H]+ | Alkaloids | 1135 | Mevalonic acid | C6H12O4 | [M-H]- | Organic acids |
| 167 | 5-Hydroxyindole-3-acetic acid | C10H9NO3 | [M-H]- | Alkaloids | 1136 | Aminomalonic acid | C3H5NO4 | [M-H]- | Organic acids |
| 168 | N-Acetyl-5-hydroxytryptamine | C12H14N2O2 | [M+H]+ | Alkaloids | 1137 | Piperonylic acid | C8H6O4 | [M+H]+ | Organic acids |
| 169 | Indole-5-carboxylic acid* | C9H7NO2 | [M-H]- | Alkaloids | 1138 | 2-Aminoethanesulfonic acid | C2H7NO3S | [M-H]- | Organic acids |
| 170 | Indole-3-carboxylic acid* | C9H7NO2 | [M-H]- | Alkaloids | 1139 | 1-Aminocyclopropane-1-carboxylic acid* | C4H7NO2 | [M+H]+ | Organic acids |
| 171 | Methoxyindoleacetic acid | C11H11NO3 | [M+H]+ | Alkaloids | 1140 | 3-Guanidinopropionic acid | C4H9N3O2 | [M-H]- | Organic acids |
| 172 | 4-Aminoindole | C8H8N2 | [M+H]+ | Alkaloids | 1141 | Tianshic acid | C18H34O5 | [M-H]- | Organic acids |
| 173 | Indole-3-cyano-2-O-glucoside | C16H18N2O5S | [M-H]- | Alkaloids | 1142 | 3-Ureidopropionic Acid | C4H8N2O3 | [M-H]- | Organic acids |
| 174 | Trigonelline | C7H7NO2 | [M+H]+ | Alkaloids | 1143 | 3-Hydroxyglutaric acid* | C5H8O5 | [M-H]- | Organic acids |
| 175 | Nicotine | C10H14N2 | [M+H]+ | Alkaloids | 1144 | 4-Hydroxy-2-oxoglutaric acid | C5H6O6 | [M-H]- | Organic acids |
| 176 | 6-Methylnicotinamide | C7H8N2O | [M+H]+ | Alkaloids | 1145 | Mandelic acid-β-glucoside | C14H18O8 | [M-H]- | Organic acids |
| 177 | Anabasine-glucoside | C16H24O5N2 | [M+H]+ | Alkaloids | 1146 | Glucosyl 2-Hydroxy-4-Methylpentanoic Acid | C12H22O8 | [M-H]- | Organic acids |
| 178 | 6-Hydroxynicotinic acid | C6H5NO3 | [M+H]+ | Alkaloids | 1147 | Abscisic acid | C15H20O4 | [M-H]- | Organic acids |
| 179 | trans-3'-Hydroxycotinine-O-glucoside | C16H22N2O7 | [M+H]+ | Alkaloids | 1148 | 6-Aminocaproic acid | C6H13NO2 | [M+H]+ | Organic acids |
| 180 | Nicotinic Acid Methyl Ester(Methyl Nicotinate) | C7H7NO2 | [M+H]+ | Alkaloids | 1149 | 2-Hydroxyhexadecanoic acid* | C16H32O3 | [M-H]- | Organic acids |
| 181 | Pyridylcarbonyl-2-glucosyloxy-2-phenylacetic acid amide | C20H22N2O8 | [M+H]+ | Alkaloids | 1150 | δ-Guanidinovaleric acid | C6H13N3O2 | [M+H]+ | Organic acids |
| 182 | Stachydrine | C7H13NO2 | [M+H]+ | Alkaloids | 1151 | Creatinine | C4H7N3O | [M+H]+ | Organic acids |
| 183 | Pterolactam* | C5H9NO2 | [M+H]+ | Alkaloids | 1152 | 2-Isopropylmalic Acid | C7H12O5 | [M-H]- | Organic acids |
| 184 | 3-hydroxy-1-methylpyrrolidin-2-one* | C5H9NO2 | [M+H]+ | Alkaloids | 1153 | 2-Hydroxyethylphosphonic acid | C2H7O4P | [M-H]- | Organic acids |
| 185 | 5-Hydroxy-2-pyrrolidinone | C4H7NO2 | [M+H]+ | Alkaloids | 1154 | (2S)-2-[(1R)-1-carboxyethoxy]propanoic acid | C6H10O5 | [M+H]+ | Organic acids |
| 186 | 3,5-Dihydro-2H-Furo[3,2-C]Quinolin-4-One* | C11H9NO2 | [M+H]+ | Alkaloids | 1155 | 2-Propylmalic Acid | C7H12O5 | [M-H]- | Organic acids |
| 187 | Quinine | C20H24O2N2 | [M+H]+ | Alkaloids | 1156 | Cis-Aconitic acid | C6H6O6 | [M-H]- | Organic acids |
| 188 | 5-Hydroxyquinoline | C11H11NO2 | [M+H]+ | Alkaloids | 1157 | β-Hydroxyisovaleric acid | C5H10O3 | [M-H]- | Organic acids |
| 189 | 2,4-Dihydroxyquinoline | C9H7NO2 | [M+H]+ | Alkaloids | 1158 | Phosphoenolpyruvate | C3H5O6P | [M-H]- | Organic acids |
| 190 | Cinchonidine | C19H22N2O | [M+H]+ | Alkaloids | 1159 | 2-Methylglutaric acid | C6H10O4 | [M-H]- | Organic acids |
| 191 | 4,6-Dihydroxyquinoline | C9H7NO2 | [M+H]+ | Alkaloids | 1160 | 4-Hydroxycyclohexylcarboxylic acid | C7H12O3 | [M-H]- | Organic acids |
| 192 | Quinidine | C20H24N2O2 | [M+H]+ | Alkaloids | 1161 | 2,2-Dimethylsuccinic acid | C6H10O4 | [M-H]- | Organic acids |
| 193 | N-Methylisoleucine | C7H15NO2 | [M+H]+ | Amino acids and derivatives | 1162 | 3-Furoic acid | C5H4O3 | [M-H]- | Organic acids |
| 194 | D-Allo-Isoleucine* | C6H13NO2 | [M+H]+ | Amino acids and derivatives | 1163 | L-Citramalic acid | C5H8O5 | [M-H]- | Organic acids |
| 195 | N-Methyl-L-proline* | C6H11NO2 | [M+H]+ | Amino acids and derivatives | 1164 | 3-(Beta-D-Glucopyranosyloxy)-5-Hydroxyhexanoic Acid Methyl Ester | C13H24O9 | [M-H]- | Organic acids |
| 196 | L-Norleucine* | C6H13NO2 | [M+H]+ | Amino acids and derivatives | 1165 | 2-Propylglutaric acid | C8H14O4 | [M-H]- | Organic acids |
| 197 | Oxiglutatione | C20H32N6O12S2 | [M-H]- | Amino acids and derivatives | 1166 | DL-Glyceric Acid | C3H6O4 | [M-H]- | Organic acids |
| 198 | Pyroglutamic acid | C5H7NO3 | [M+H]+ | Amino acids and derivatives | 1167 | 2-Oxoadipic acid | C6H8O5 | [M-H]- | Organic acids |
| 199 | Met-Ser-Ala | C11H21N3O5S1 | [M+H]+ | Amino acids and derivatives | 1168 | Oxalic acid | C2H2O4 | [M-H]- | Organic acids |
| 200 | L-Cyclopentylglycine | C7H13NO2 | [M+H]+ | Amino acids and derivatives | 1169 | Creatine | C4H9N3O2 | [M-H]- | Organic acids |
| 201 | L-Aspartic acid | C4H7NO4 | [M+H]+ | Amino acids and derivatives | 1170 | 2-Hydroxy-2-methyl-3-oxobutanoic acid | C5H8O4 | [M-H]- | Organic acids |
| 202 | S-Methyl-L-cysteine | C4H9NO2S | [M+H]+ | Amino acids and derivatives | 1171 | Citric Acid diglucoside | C18H28O17 | [M-H]- | Organic acids |
| 203 | Methionylvaline | C10H20N2O3S | [M+H]+ | Amino acids and derivatives | 1172 | 1-Naphthylacetic acid | C12H10O2 | [M+H]+ | Organic acids |
| 204 | Asn-Cys-Gly | C9H16N4O5S1 | [M+H]+ | Amino acids and derivatives | 1173 | 2-Hydroxyphenylacetic acid | C8H8O3 | [M-H]- | Organic acids |
| 205 | L-Phenylalanine | C9H11NO2 | [M+H]+ | Amino acids and derivatives | 1174 | 5-Acetamidopentanoic Acid | C7H13NO3 | [M-H]- | Organic acids |
| 206 | N-Ethylglycine* | C4H9NO2 | [M+H]+ | Amino acids and derivatives | 1175 | 2-(4-Aminobutanamido)-3-(1-methyl-1H-imidazol-5-yl)propanoic acid | C10H16N4O3 | [M+H]+ | Organic acids |
| 207 | L-Valine* | C5H11NO2 | [M+H]+ | Amino acids and derivatives | 1176 | 4-Hydroxy-2-Oxopentanoic Acid | C5H8O4 | [M-H]- | Organic acids |
| 208 | Cyclo(Ala-Gly) | C5H8N2O2 | [M+H]+ | Amino acids and derivatives | 1177 | 4,8-Dihydroxyquinoline-2-carboxylic acid | C10H7NO4 | [M-H]- | Organic acids |
| 209 | L-threo-3-Methylaspartate | C5H9NO4 | [M-H]- | Amino acids and derivatives | 1178 | 3,4,5-Trihydroxy-6-[5-hydroxy-2-(4-hydroxyphenyl)-4-oxochromen-7-yl]oxyoxane-2-carboxylic acid | C21H18O11 | [M-H]- | Organic acids |
| 210 | N-Acetyl-L-phenylalanine | C11H13NO3 | [M-H]- | Amino acids and derivatives | 1179 | Suberic Acid | C8H14O4 | [M-H]- | Organic acids |
| 211 | L-Glutamic acid | C5H9NO4 | [M+H]+ | Amino acids and derivatives | 1180 | 3-Hydroxypropanoic acid | C3H6O3 | [M-H]- | Organic acids |
| 212 | 2-Amino-4-hydroxy-3-methylpentanoic acid | C6H13NO3 | [M+H]+ | Amino acids and derivatives | 1181 | Oxalacetic acid | C4H4O5 | [M-H]- | Organic acids |
| 213 | Methyl 3-aminopropanoate | C4H9NO2 | [M+H]+ | Amino acids and derivatives | 1182 | Argininosuccinic acid | C10H18N4O6 | [M-H]- | Organic acids |
| 214 | 6-(((S)-1-carboxyethyl)amino)-4-hydroxyhexanoicacid* | C9H17NO5 | [M-H]- | Amino acids and derivatives | 1183 | D-Erythronolactone | C4H6O4 | [M-H]- | Organic acids |
| 215 | L-Histidine | C6H9N3O2 | [M+H]+ | Amino acids and derivatives | 1184 | γ-Aminobutyric acid | C4H9NO2 | [M+H]+ | Organic acids |
| 216 | N-Methyl-Trans-4-Hydroxy-L-Proline* | C6H11NO3 | [M+H]+ | Amino acids and derivatives | 1185 | Methyl jasmonate | C13H20O3 | [M+H]+ | Organic acids |
| 217 | N(6),N(6)-Dimethyl-L-lysine | C8H18N2O2 | [M+H]+ | Amino acids and derivatives | 1186 | Allantoin | C4H6N4O3 | [M-H]- | Organic acids |
| 218 | Cycloleucine* | C6H11NO2 | [M+H]+ | Amino acids and derivatives | 1187 | 2-Hydroxyisocaproic acid | C6H12O3 | [M-H]- | Organic acids |
| 219 | N-Acetyl-L-glutamic acid | C7H11NO5 | [M-H]- | Amino acids and derivatives | 1188 | Methyl dihydrojasmonate | C13H22O3 | [M+H]+ | Organic acids |
| 220 | L-Homoserine | C4H9NO3 | [M+H]+ | Amino acids and derivatives | 1189 | 2-Picolinic acid | C6H5NO2 | [M-H]- | Organic acids |
| 221 | DL-Leucine* | C6H13NO2 | [M+H]+ | Amino acids and derivatives | 1190 | 2-Acetyl-2-Hydroxybutanoic Acid | C6H10O4 | [M-H]- | Organic acids |
| 222 | L-Arginine | C6H14N4O2 | [M+H]+ | Amino acids and derivatives | 1191 | 2-Propylsuccinic acid* | C7H12O4 | [M-H]- | Organic acids |
| 223 | L-Tyrosine* | C9H11NO3 | [M+H]+ | Amino acids and derivatives | 1192 | Ethyl butyrate* | C6H12O2 | [M+H]+ | Organic acids |
| 224 | 3-Hydroxy-L-phenylalanine* | C9H11NO3 | [M+H]+ | Amino acids and derivatives | 1193 | Ethyl isobutyrate* | C6H12O2 | [M+H]+ | Organic acids |
| 225 | L-Valinol | C5H13NO | [M+H]+ | Amino acids and derivatives | 1194 | Pimelic acid* | C7H12O4 | [M-H]- | Organic acids |
| 226 | N-Acetyl-L-tyrosine | C11H13NO4 | [M+H]+ | Amino acids and derivatives | 1195 | Phytic acid | C6H18O24P6 | [M-H]- | Organic acids |
| 227 | 3-Hydroxy-3-methylpentane-1,5-dioic acid | C6H10O5 | [M-H]- | Amino acids and derivatives | 1196 | 2-Methyl-3-oxosuccinic acid | C5H6O5 | [M-H]- | Organic acids |
| 228 | Allysine(6-Oxo DL-Norleucine) | C6H11NO3 | [M-H]- | Amino acids and derivatives | 1197 | Itaconic acid | C5H6O4 | [M-H]- | Organic acids |
| 229 | Trans-4-Hydroxy-L-proline* | C5H9NO3 | [M+H]+ | Amino acids and derivatives | 1198 | DL-3-Phenyllactic acid* | C9H10O3 | [M-H]- | Organic acids |
| 230 | DL-Threonine | C4H9NO3 | [M+H]+ | Amino acids and derivatives | 1199 | 3-Hydroxy-3-Methyl-2-Oxopentanoic Acid | C6H10O4 | [M-H]- | Organic acids |
| 231 | D-Ornithine | C5H12N2O2 | [M+H]+ | Amino acids and derivatives | 1200 | 5-Aminovaleric acid | C5H11NO2 | [M+H]+ | Organic acids |
| 232 | 4-Hydroxy-L-Isoleucine | C6H13NO3 | [M+H]+ | Amino acids and derivatives | 1201 | Succinic semialdehyde | C4H6O3 | [M-H]- | Organic acids |
| 233 | cyclo-(Gly-Phe) | C11H12N2O2 | [M+H]+ | Amino acids and derivatives | 1202 | L-Lactic Acid | C3H6O3 | [M-H]- | Organic acids |
| 234 | N,N'-Dimethylarginine;SDMA | C8H18N4O2 | [M+H]+ | Amino acids and derivatives | 1203 | 2,3-Dimethylmaleic anhydride | C6H6O3 | [M-H]- | Organic acids |
| 235 | L-Lysine-Butanoic Acid | C10H22N2O4 | [M+H]+ | Amino acids and derivatives | 1204 | N-Methyl-4-aminobutyric acid | C5H11NO2 | [M+H]+ | Organic acids |
| 236 | Trimethyllysine | C9H20N2O2 | [M+H]+ | Amino acids and derivatives | 1205 | 2-Decanol* | C10H22O | [M-H]- | Others |
| 237 | Glutathione reduced form | C10H17N3O6S | [M-H]- | Amino acids and derivatives | 1206 | 1-Decanol* | C10H22O | [M-H]- | Others |
| 238 | N-Methyl-α-aminoisobutyric acid | C5H11NO2 | [2M+H]+ | Amino acids and derivatives | 1207 | 1,4-Benzodioxin-6-propanol | C11H12O3 | [M+H]+ | Others |
| 239 | N-α-Acetyl-L-ornithine | C7H14N2O3 | [M-H]- | Amino acids and derivatives | 1208 | 2,6-Dimethyl-7-octene-2,3,6-triol | C10H20O3 | [M-H]- | Others |
| 240 | Cyclo(Pro-Glu) | C10H14N2O4 | [M+H]+ | Amino acids and derivatives | 1209 | D-Panthenol | C9H19NO4 | [M+H]+ | Others |
| 241 | N-(acetyl)phenylalanine | C11H13NO3 | [M-H]- | Amino acids and derivatives | 1210 | 3,4-methylenedioxy cinnamyl alcohol | C10H10O3 | [M-H]- | Others |
| 242 | N-Acetyl-L-threonine | C6H11NO4 | [M-H]- | Amino acids and derivatives | 1211 | (R)-Citronellol | C10H20O | [M+H]+ | Others |
| 243 | L-Glutamine | C5H10N2O3 | [M+H]+ | Amino acids and derivatives | 1212 | 5-Hydroxymethylfurfural | C6H6O3 | [M+H]+ | Others |
| 244 | Tridecanoylglycine | C15H29NO3 | [M+H]+ | Amino acids and derivatives | 1213 | 4-Hydroxybenzaldehyde | C7H6O2 | [M-H]- | Others |
| 245 | O-Acetyl-L-homoserine | C6H11NO4 | [M+H]+ | Amino acids and derivatives | 1214 | Isovanillin | C8H8O3 | [M+H]+ | Others |
| 246 | L-Lysine | C6H14N2O2 | [M+H]+ | Amino acids and derivatives | 1215 | Protocatechualdehyde | C7H6O3 | [M-H]- | Others |
| 247 | 3,4-Dihydroxy-L-phenylalanine (L-Dopa) | C9H11NO4 | [M+H]+ | Amino acids and derivatives | 1216 | 3-Methylbenzaldehyde | C8H8O | [M-H]- | Others |
| 248 | L-Methionine Sulfoxide | C5H11NO3S | [M+H]+ | Amino acids and derivatives | 1217 | 3,4-Methylenedioxycinnamaldehyde | C10H8O3 | [M+H]+ | Others |
| 249 | S-(5'-Adenosy)-L-homocysteine | C14H20N6O5S | [M+H]+ | Amino acids and derivatives | 1218 | Vanillin; 4-Hydroxy-3-Methoxybenzaldehyde* | C8H8O3 | [M-H]- | Others |
| 250 | O-Acetylserine | C5H9NO4 | [M+H]+ | Amino acids and derivatives | 1219 | Mesitaldehyde | C10H12O | [M+H]+ | Others |
| 251 | Nitroarginine | C6H13N5O4 | [M+Na]+ | Amino acids and derivatives | 1220 | 4-Ethylbenzaldehyde | C9H10O | [M+H]+ | Others |
| 252 | Glycylphenylalanine | C11H14N2O3 | [M+H]+ | Amino acids and derivatives | 1221 | Syringaldehyde; 4-Hydroxy-3,5-Dimethoxybenzaldehyde | C9H10O4 | [M+H]+ | Others |
| 253 | L-α-Glutamyl-L-Glutamic Acid | C10H16N2O7 | [M+H]+ | Amino acids and derivatives | 1222 | 2,6-Dimethoxybenzaldehyde* | C9H10O3 | [M-H]- | Others |
| 254 | S-(Methyl)glutathione | C11H19N3O6S | [M+H]+ | Amino acids and derivatives | 1223 | Benzaldehyde | C7H6O | [M+H]+ | Others |
| 255 | 1-Methylhistidine | C7H11N3O2 | [M+H]+ | Amino acids and derivatives | 1224 | 3,5,7,4'-Tetrahydroxy-Coumaronochromone | C15H10O7 | [M+H]+ | Others |
| 256 | S-Ribosyl-L-homocysteine | C9H17NO6S | [M-H]- | Amino acids and derivatives | 1225 | 3,7-Dihydroxychromen-4-one | C9H6O4 | [M+H]+ | Others |
| 257 | L-Cystine | C6H12N2O4S2 | [M+H]+ | Amino acids and derivatives | 1226 | 5,7-Dihydroxychromone glucoside | C15H16O9 | [M+H]+ | Others |
| 258 | Arginine methyl ester* | C7H16N4O2 | [M+H]+ | Amino acids and derivatives | 1227 | Capillarisin | C16H12O7 | [M+H]+ | Others |
| 259 | L-Carnosine | C9H14N4O3 | [M+H]+ | Amino acids and derivatives | 1228 | 6,7-dimethoxy-2-[2-(4'-hydroxy-3'-methoxyphenyl)ethyl]chromone* | C20H20O6 | [M+H]+ | Others |
| 260 | Cyclo(D-Val-L-Pro) | C10H16N2O2 | [M+H]+ | Amino acids and derivatives | 1229 | 6,7-dimethoxy-2-[2-phenylethyl]chromone | C19H18O4 | [M+H]+ | Others |
| 261 | Homoarginine | C7H16N4O2 | [M+H]+ | Amino acids and derivatives | 1230 | 6-hydroxy-2-[2-(3,4-dimethoxyphenyl)ethyl]chromone | C19H18O5 | [M+H]+ | Others |
| 262 | γ-Glutamyl-L-valine | C10H18N2O5 | [M+H]+ | Amino acids and derivatives | 1231 | aloeresin H | C38H42O18 | [M-H]- | Others |
| 263 | L-Glutamic acid-O-glycoside | C11H19NO9 | [M-H]- | Amino acids and derivatives | 1232 | 7-Hydroxy-4-chromone | C9H6O3 | [M+H]+ | Others |
| 264 | N-Alpha-Acetyl-L-Asparagine | C6H10N2O4 | [M-H]- | Amino acids and derivatives | 1233 | xylosyl -5-hydroxychromone-7-O-β-D-glucoside | C20H24O13 | [M-H]- | Others |
| 265 | N-Acetyl-L-glycine | C4H7NO3 | [M-H]- | Amino acids and derivatives | 1234 | (3,5-dihydroxyphenyl)-(4-hydroxy-2,6-dimethoxyphenyl)methanone* | C15H14O6 | [M+H]+ | Others |
| 266 | S-Adenosylmethionine | C15H23N6O5S+ | [M]+ | Amino acids and derivatives | 1235 | 9-hydroxy-2-(2-hydroxy-3-methoxyphenyl)-3-(hydroxymethyl)-12-methoxy-2,3-dihydro-1,4,5-trioxatetraphen-10-one* | C24H20O9 | [M-H]- | Others |
| 267 | N-Acetyl-L-leucine | C8H15NO3 | [M+H]+ | Amino acids and derivatives | 1236 | 9-hydroxy-2-(3-hydroxy-4-methoxyphenyl)-3-(hydroxymethyl)-12-methoxy-2,3-dihydro-1,4,5-trioxatetraphen-10-one* | C24H20O9 | [M-H]- | Others |
| 268 | L-Serine | C3H7NO3 | [M+H]+ | Amino acids and derivatives | 1237 | Icariside E5* | C26H34O11 | [M-H]- | Others |
| 269 | N-ethyl-leucine | C8H17NO2 | [M+H]+ | Amino acids and derivatives | 1238 | 2,4-Dihydroxy-6-methoxyacetophenone* | C9H10O4 | [M+H]+ | Others |
| 270 | N-Monomethyl-L-arginine* | C7H16N4O2 | [M+H]+ | Amino acids and derivatives | 1239 | Koparin | C16H12O6 | [M+H]+ | Others |
| 271 | 5-Oxo-L-Proline | C5H7NO3 | [M-H]- | Amino acids and derivatives | 1240 | 3,5-Dihydroxy-2-(4-hydroxyphenyl)-7-[(3,4,5-trihydroxy-6-methyltetrahydro-2H-pyran-2-yl)oxy]-4H-1-benzopyran-4-one* | C21H20O10 | [M-H]- | Others |
| 272 | L-Isoleucyl-L-Aspartate | C10H18N2O5 | [M+H]+ | Amino acids and derivatives | 1241 | 7-(3,7-dimethylocta-2,6-dien-1-yl)-4-hydroxy-3-(3-hydroxybenzoyl)-8,8-dimethyl-1,5-bis(3-methylbut-2-en-1-yl)bicyclo[3.3.1]non-3-ene-2,9-dione | C38H50O5 | [M+H]+ | Others |
| 273 | L-Citrulline | C6H13N3O3 | [M+H]+ | Amino acids and derivatives | 1242 | Annuionone D | C13H20O3 | [M+H]+ | Others |
| 274 | N-Phenylacetylglycine | C10H11NO3 | [M+H]+ | Amino acids and derivatives | 1243 | Quercetin 3-O-alpha-L-rhamnoside | C21H20O11 | [M-H]- | Others |
| 275 | L-Tyrosine methyl ester | C10H13NO3 | [M+H]+ | Amino acids and derivatives | 1244 | Primin* | C12H16O3 | [M+H]+ | Others |
| 276 | L-Homocysteine | C4H9NO2S | [M+H]+ | Amino acids and derivatives | 1245 | Aquilegiolide | C8H8O3 | [M+H]+ | Others |
| 277 | N6-Acetyl-L-lysine | C8H16N2O3 | [M+H]+ | Amino acids and derivatives | 1246 | Dihydroquercetin | C15H12O7 | [M+H]+ | Others |
| 278 | L-Leucyl-L-phenylalanine | C15H22N2O3 | [M+H]+ | Amino acids and derivatives | 1247 | 1-phenyl-7-(4-hydroxyphenyl)-4-ene-3-heptanone | C19H20O2 | [M+H]+ | Others |
| 279 | 5-L-Glutamyl-L-amino acid | C8H14N2O5 | [M-H]- | Amino acids and derivatives | 1248 | Grevilloside Q | C15H18O9 | [M-H]- | Others |
| 280 | 1-(2-Amino-4-methylpentanoyl)pyrrolidine-2-carboxylic acid | C11H20N2O3 | [M+H]+ | Amino acids and derivatives | 1249 | 3-Hydroxy-1-(4-hydroxy-3,5-dimethoxyphenyl)propan-1-one | C11H14O5 | [M+H]+ | Others |
| 281 | L-Tryptophan | C11H12N2O2 | [M-H]- | Amino acids and derivatives | 1250 | 2-benzoyl-4-[(2r)-1-hydroxy-7-methyl-3-methylideneoct-6-en-1-yl]benzene-1,3,5-triol | C23H26O5 | [M+H]+ | Others |
| 282 | L-Glycyl-L-isoleucine | C8H16N2O3 | [M+H]+ | Amino acids and derivatives | 1251 | α-Cyperone | C15H22O | [M+H]+ | Others |
| 283 | L-Cystathionine | C7H14N2O4S | [M+H]+ | Amino acids and derivatives | 1252 | 4-Hydroxyacetophenone* | C8H8O2 | [M-H]- | Others |
| 284 | L-Alanyl-L-leucine | C9H18N2O3 | [M+H]+ | Amino acids and derivatives | 1253 | 1-(4-hydroxy-3-methoxyphenyl)-7-phenyl-3,5-diheptanone* | C20H22O4 | [M+H]+ | Others |
| 285 | γ-Glutamylphenylalanine | C14H18N2O5 | [M-H]- | Amino acids and derivatives | 1254 | 1-(4-hydroxyphenyl)-7-(4-hydroxy-3-methoxyphenyl)-4-ene-3-heptanone | C20H22O4 | [M+H]+ | Others |
| 286 | N-alanicacidalkyl-alanine | C6H11NO4 | [M-H]- | Amino acids and derivatives | 1255 | Benzylacetone | C10H12O | [M+H]+ | Others |
| 287 | L-Leucyl-L-Leucine | C12H24N2O3 | [M+H]+ | Amino acids and derivatives | 1256 | Deoxysappanone B trimethyl ether | C19H20O5 | [M+H]+ | Others |
| 288 | S-(5'-Adenosyl)-L-methionine | C15H22N6O5S | [M+H]+ | Amino acids and derivatives | 1257 | 3,4-Dihydroxyacetophenone | C8H8O3 | [M-H]- | Others |
| 289 | N-Acetyl-L-Aspartic Acid | C6H9NO5 | [M-H]- | Amino acids and derivatives | 1258 | Senkyunolide M | C16H22O4 | [M+H]+ | Others |
| 290 | L-Asparagine | C4H8N2O3 | [M+H]+ | Amino acids and derivatives | 1259 | Senkyunolide B | C12H12O3 | [M+H]+ | Others |
| 291 | L-Prolyl-L-Phenylalanine | C14H18N2O3 | [M+H]+ | Amino acids and derivatives | 1260 | Senkyunolide K | C12H16O3 | [M+H]+ | Others |
| 292 | L-Homocitrulline | C7H15N3O3 | [M+H]+ | Amino acids and derivatives | 1261 | Dihydroactinidiolide | C11H16O2 | [M+H]+ | Others |
| 293 | L-Valyl-L-Leucine | C11H22N2O3 | [M+H]+ | Amino acids and derivatives | 1262 | Lincomolide B | C17H26O3 | [M-H]- | Others |
| 294 | Gly-Pro-Arg | C13H24N6O4 | [M+H]+ | Amino acids and derivatives | 1263 | Hydroxydihydrobovolide* | C11H18O3 | [M-H]- | Others |
| 295 | Cyclo(Tyr-Ala) | C12H14N2O3 | [M+H]+ | Amino acids and derivatives | 1264 | Dihydrokaempferol | C15H12O6 | [M-H]- | Others |
| 296 | N-Methyl-L-Glutamate | C6H11NO4 | [M-H]- | Amino acids and derivatives | 1265 | 1-18:3-LysoPC | C26H48NO7P | [M+H]+ | Others |
| 297 | L-Saccharopine | C11H20N2O6 | [M-H]- | Amino acids and derivatives | 1266 | Monoacetyldapsone | C14H14N2O3S | [M+H]+ | Others |
| 298 | N-Methylglycine | C3H7NO2 | [M-H]- | Amino acids and derivatives | 1267 | Lauryldiethanolamine* | C16H35NO2 | [M+H]+ | Others |
| 299 | L-Glutamine-O-glycoside | C11H20N2O8 | [M-H]- | Amino acids and derivatives | 1268 | Noreugenin-7-O-glucoside* | C16H18O9 | [M+H]+ | Others |
| 300 | N-(2-Methylbenzoyl)glycine | C10H11NO3 | [M+H]+ | Amino acids and derivatives | 1269 | 3-Ethyl-7-hydroxyphthalide | C10H10O3 | [M+H]+ | Others |
| 301 | Teupolioside | C35H46O20 | [M-H]- | Amino acids and derivatives | 1270 | 2α,3α-epoxy-5,7,3',4'-tetrahydroxyflavan-(4b→8)-epicatechin | C30H24O12 | [M+H]+ | Others |
| 302 | L-Methionine methyl ester | C6H13NO2S | [M+H]+ | Amino acids and derivatives | 1271 | cis-coumarinic acid-beta-D-glucoside | C15H18O8 | [M-H]- | Others |
| 303 | N-Acetyl-L-Arginine | C8H16N4O3 | [M+H]+ | Amino acids and derivatives | 1272 | epiloliolide | C11H16O3 | [M+H]+ | Others |
| 304 | L-γ-Glutamyl-L-leucine | C11H20N2O5 | [M+H]+ | Amino acids and derivatives | 1273 | 4-Phenyl-1,2,3-thiadiazole | C8H6N2S | [M+H]+ | Others |
| 305 | O-Phospho-L-serine | C3H8NO6P | [M-H]- | Amino acids and derivatives | 1274 | Naringenin 7-0-glucoside* | C21H22O10 | [M-H]- | Others |
| 306 | Cyclo(Ser-Pro) | C8H12N2O3 | [M+H]+ | Amino acids and derivatives | 1275 | Melilotoside | C15H18O8 | [M-H]- | Others |
| 307 | Cyclo(L-Ala-L-Pro) | C8H12N2O2 | [M+H]+ | Amino acids and derivatives | 1276 | Butyl 1,3,5-trihydroxy-4-{[3-(4-hydroxy-3-methoxyphenyl)prop-2-enoyl]oxy}cyclohexane-1-carboxylate | C21H28O9 | [M+H]+ | Others |
| 308 | 4-Hydroxy-L-glutamic acid | C5H9NO5 | [M-H]- | Amino acids and derivatives | 1277 | 4-Methylumbelliferyl glucuronide | C16H16O9 | [M-H]- | Others |
| 309 | Oxamic acid | C2H3NO3 | [M-H]- | Amino acids and derivatives | 1278 | 5-methylfurfural | C6H6O2 | [M+H]+ | Others |
| 310 | N-[3-(4-Hydroxyphenyl)acryloyl]-L-tyrosine | C18H17NO5 | [M+H]+ | Amino acids and derivatives | 1279 | Eugenyl formate | C11H12O3 | [M+H]+ | Others |
| 311 | L-Phenylalanyl-L-phenylalanine | C18H20N2O3 | [M+H]+ | Amino acids and derivatives | 1280 | 7S-O-methyl morroniside | C18H28O11 | [M-H]- | Others |
| 312 | L-Valyl-L-Phenylalanine | C14H20N2O3 | [M+H]+ | Amino acids and derivatives | 1281 | 3,5-Dimethyl-2,3-dihydrobenzofuran | C10H12O | [M+H]+ | Others |
| 313 | D-Alanyl-D-Alanine | C6H12N2O3 | [M-H]- | Amino acids and derivatives | 1282 | (R)-3-ethyl-7-hydroxy-6-methoxyphthalide | C11H12O4 | [M+H]+ | Others |
| 314 | L-Homocystine | C8H16N2O4S2 | [M+H]+ | Amino acids and derivatives | 1283 | 1-(4'-Hydroxy-3'-methoxyphenyl)-2-[4''-(3-hydroxypropyl)-2'',6''-dimethoxyphenyl]-propane-1,3-Diol | C21H28O8 | [M-H]- | Others |
| 315 | N-Propionylglycine | C5H9NO3 | [M-H]- | Amino acids and derivatives | 1284 | acetophenone | C8H8O | [M+H]+ | Others |
| 316 | Val-Abu-OH | C14H18N2O6 | [M+H]+ | Amino acids and derivatives | 1285 | Lauramine oxide | C14H31NO | [M+H]+ | Others |
| 317 | His-Val-Ser | C14H23N5O5 | [M+H]+ | Amino acids and derivatives | 1286 | 3ξ-(1ξ-hydroxyethyl)-7-hydroxy-1-isobenzofuranone | C10H10O4 | [M+H]+ | Others |
| 318 | DL-Methionine | C5H11NO2S | [M+H]+ | Amino acids and derivatives | 1287 | Pyramidatin F | C22H24O7 | [M+H]+ | Others |
| 319 | N-Acetylneuraminic acid | C11H19NO9 | [M-H]- | Amino acids and derivatives | 1288 | 3-Hydroxy-5-Methoxyphenyl-6-O-Galloyl-Beta-D-Galactopyranoside* | C20H22O12 | [M-H]- | Others |
| 320 | Cinnamoylglycine | C11H11NO3 | [M+H]+ | Amino acids and derivatives | 1289 | Glucosyl 7-methyl-3-methyleneoctane-1,2,6,7-tetraol | C16H30O9 | [M-H]- | Others |
| 321 | Cyclo(L-Phe-trans-4-hydroxy-L-Pro) | C14H16N2O3 | [M+H]+ | Amino acids and derivatives | 1290 | 2,6-Dimethoxy-4-hydroxyphenol-1-O-ß-D-glucopyranoside | C14H20O9 | [M-H]- | Others |
| 322 | Cyclo(L-Leu-trans-4-hydroxy-L-Pro) | C11H18N2O3 | [M+H]+ | Amino acids and derivatives | 1291 | 2-(hydroxymethyl)-6-[4-(3-hydroxypropyl)-2,6-dimethoxyphenoxy]oxane-3,4,5-triol | C17H26O9 | [M+H]+ | Others |
| 323 | Cyclo(D-Phe-L-Pro) | C14H16N2O2 | [M+H]+ | Amino acids and derivatives | 1292 | Hydroxygeranyl 6-O-xylopyranosyl-glucopyranoside | C21H36O11 | [M-H]- | Others |
| 324 | Asp-Met | C9H16N2O5S | [M+H]+ | Amino acids and derivatives | 1293 | Glucosyl 3,7-dimethyloct-2-ene-1,6,7-triol | C16H30O8 | [M+CH3COOH-H]- | Others |
| 325 | 3-Cyano-L-alanine | C4H6N2O2 | [M-H]- | Amino acids and derivatives | 1294 | Salicylacyl Glucuronide | C13H14O9 | [M+H]+ | Others |
| 326 | S-Sulfo-L-Cysteine | C3H7NO5S2 | [M-H]- | Amino acids and derivatives | 1295 | Glucosyl 3,7-dimethylocta-2,6-diene-1,4,8-trio | C16H28O8 | [M+CH3COOH-H]- | Others |
| 327 | Cyclo(Pro-Leu) | C11H18N2O2 | [M+H]+ | Amino acids and derivatives | 1296 | AICA ribonucleotide | C9H15N4O8P | [M+CH3COO]- | Others |
| 328 | 6-Hydroxydopaquinone | C9H9NO5 | [M-H]- | Amino acids and derivatives | 1297 | 5-hydroxy-3,4-dimethyl-5-pentylfuran-2(5H)-one* | C11H18O3 | [M-H]- | Others |
| 329 | 3-Hydroxyphloretin-4'-O-glucoside | C21H24O11 | [M-H]- | Flavonoids | 1298 | 9-Oxodemethylmyricanane B | C20H20O5 | [M-H]- | Others |
| 330 | Okanin-4'-O-glucoside(Marein)* | C21H22O11 | [M+H]+ | Flavonoids | 1299 | D-Glucose-1-phosphate* | C6H13O9P | [M-H]- | Others |
| 331 | Carthamone* | C21H20O11 | [M-H]- | Flavonoids | 1300 | D-Fructose 6-Phosphate* | C6H13O9P | [M-H]- | Others |
| 332 | Sappanchalcone | C16H14O5 | [M-H]- | Flavonoids | 1301 | L-Gulonolactone | C6H10O6 | [M+CH3COO]- | Others |
| 333 | 3,4,2',4',6'-Pentahydroxychalcone | C15H12O6 | [M+H]+ | Flavonoids | 1302 | Gluconic acid | C6H12O7 | [M-H]- | Others |
| 334 | Hydroxy isoliquiritigenin glucoside* | C21H22O10 | [M-H]- | Flavonoids | 1303 | D-Xylonic acid | C5H10O6 | [M-H]- | Others |
| 335 | Phloretin-2'-O-glucoside (Phlorizin) | C21H24O10 | [M+H]+ | Flavonoids | 1304 | (2r,3s,4s,5r)-2,5-bis(hydroxymethyl)-2-methoxyoxolane-3,4-diol | C7H14O6 | [M-H]- | Others |
| 336 | 3,4,2',4',6'-Pentahydroxychalcone-4'-O-glucoside | C21H22O11 | [M-H]- | Flavonoids | 1305 | D-Sedoheptuiose 7-phosphate | C7H15O10P | [M-H]- | Others |
| 337 | 2,4,2',4'-tetrahydroxy-3'-prenylchalcone | C20H20O5 | [M+H]+ | Flavonoids | 1306 | D-Galacturonic acid* | C6H10O7 | [M-H]- | Others |
| 338 | Naringenin chalcone; 2',4,4',6'-Tetrahydroxychalcone* | C15H12O5 | [M+H]+ | Flavonoids | 1307 | D-Threose | C4H8O4 | [M+H]+ | Others |
| 339 | 3-Prenyl-4,2',4'-Trihydroxychalcone | C20H20O4 | [M+H]+ | Flavonoids | 1308 | D-Galactose* | C6H12O6 | [M-H]- | Others |
| 340 | 3-Hydroxyphloretin-4'-O-(2''-O-galloyl)glucoside | C28H28O15 | [M-H]- | Flavonoids | 1309 | Laminaran | C18H32O16 | [M-H]- | Others |
| 341 | Isosalipurposide (Phlorizin Chalcone)* | C21H22O10 | [M+H]+ | Flavonoids | 1310 | Glucaric acid-1-Phosphate | C6H11PO11 | [M-H]- | Others |
| 342 | 2,4,2',5'-Tetrahydroxydihydrochalcone | C15H14O5 | [M+H]+ | Flavonoids | 1311 | D-Glucoronic acid* | C6H10O7 | [M-H]- | Others |
| 343 | 2'-Hydroxy-3,4,5,3',4',6'-hexameth-oxychalcone | C21H24O8 | [M+H]+ | Flavonoids | 1312 | D-Mannose* | C6H12O6 | [M-H]- | Others |
| 344 | Okanin-4'-(6''-O-acetyl)glucoside* | C23H24O13 | [M+H]+ | Flavonoids | 1313 | D-Fructose* | C6H12O6 | [M-H]- | Others |
| 345 | Okanin-4'-O-(6''-O-malonyl)glucoside | C24H24O14 | [M+H]+ | Flavonoids | 1314 | Inositol* | C6H12O6 | [M-H]- | Others |
| 346 | Phloretin | C15H14O5 | [M-H]- | Flavonoids | 1315 | D-Melezitose | C18H32O16 | [M-H]- | Others |
| 347 | Licochalcone B | C16H14O5 | [M+H]+ | Flavonoids | 1316 | Ribulose-5-phosphate | C5H11O8P | [M-H]- | Others |
| 348 | Okanin-4'-O-glucosyl-O-glucoside* | C27H32O16 | [M+H]+ | Flavonoids | 1317 | Maltitol | C12H24O11 | [M-H]- | Others |
| 349 | Okanin-4'-O-gentiobioside* | C27H32O16 | [M+H]+ | Flavonoids | 1318 | Sucrose-6-phosphate | C12H23O14P | [M-H]- | Others |
| 350 | Chalcone | C15H12O | [M+H]+ | Flavonoids | 1319 | L-Fucitol | C6H14O5 | [M-H]- | Others |
| 351 | Okanin-3',4'-di-O-glucoside* | C27H32O16 | [M+H]+ | Flavonoids | 1320 | D-Saccharic acid* | C6H10O8 | [M-H]- | Others |
| 352 | Epicatechin gallate | C22H18O10 | [M-H]- | Flavonoids | 1321 | Trehalose 6-phosphate | C12H23O14P | [M-H]- | Others |
| 353 | 7-O-Galloyltricetiflavan | C22H18O10 | [M-H]- | Flavonoids | 1322 | Galactinol | C12H22O11 | [M-H]- | Others |
| 354 | Epigallocatechin | C15H14O7 | [M+H]+ | Flavonoids | 1323 | D-Galactaric acid* | C6H10O8 | [M-H]- | Others |
| 355 | 3',4,4',5,7-Pentahydroxyflavan (Luteoforol) | C15H14O6 | [M+H]+ | Flavonoids | 1324 | Erythrose | C4H8O4 | [M-H]- | Others |
| 356 | Epigallocatechin-3-O-gallate | C22H18O11 | [M-H]- | Flavonoids | 1325 | 3'-Fucosyllactose | C18H32O15 | [M-H]- | Others |
| 357 | Cinchonain Ia* | C24H20O9 | [M+H]+ | Flavonoids | 1326 | D-Cellobiose | C12H22O11 | [M-H]- | Others |
| 358 | Cinchonain Ib* | C24H20O9 | [M+H]+ | Flavonoids | 1327 | 3-Dehydro-L-Threonic Acid* | C4H6O5 | [M-H]- | Others |
| 359 | Catechin-catechin-catechin | C45H38O18 | [M-H]- | Flavonoids | 1328 | 3-Phospho-D-glyceric acid | C3H7O7P | [M-H]- | Others |
| 360 | Tetrahydroxyflavan-(4α-8-epicatechin)* | C30H24O12 | [M-H]- | Flavonoids | 1329 | Digalactosylglycerol | C15H28O13 | [M-H]- | Others |
| 361 | Epicatechin-3-(3''-O-methyl)gallate | C23H20O10 | [M-H]- | Flavonoids | 1330 | D-Sorbitol | C6H14O6 | [M-H]- | Others |
| 362 | Cinchonain Id* | C24H20O9 | [M-H]- | Flavonoids | 1331 | 1-(sn-Glycero-3-phospho)-1D-myo-inositol | C9H19O11P | [M-H]- | Others |
| 363 | Epicatechin-(4β->8)-epigallocatechin | C30H26O13 | [M+H]+ | Flavonoids | 1332 | D-Threonic Acid | C4H8O5 | [M-H]- | Others |
| 364 | Catechin-(7,8-bc)-4α-(3,4-dihydroxyphenyl)-dihydro-2-(3H)-one* | C24H20O9 | [M-H]- | Flavonoids | 1333 | Allitol | C6H14O6 | [M-H]- | Others |
| 365 | Epicatechin* | C15H14O6 | [M+H]+ | Flavonoids | 1334 | D-Maltose* | C12H22O11 | [M-H]- | Others |
| 366 | Catechin-(7,8-bc)-4β-(3,4-dihydroxyphenyl)-dihydro-2-(3H)-one* | C24H20O9 | [M-H]- | Flavonoids | 1335 | Sedoheptulose | C7H14O7 | [M-H]- | Others |
| 367 | Catechin* | C15H14O6 | [M+H]+ | Flavonoids | 1336 | D-Lactose* | C12H22O11 | [M-H]- | Others |
| 368 | Cinchonain Ic* | C24H20O9 | [M-H]- | Flavonoids | 1337 | D-Sucrose* | C12H22O11 | [M-H]- | Others |
| 369 | Epicatechin glucoside* | C21H24O11 | [M-H]- | Flavonoids | 1338 | Rutinose | C12H22O10 | [M-H]- | Others |
| 370 | Epicatechin-3'-O-β-D-glucopyranoside* | C21H24O11 | [M-H]- | Flavonoids | 1339 | N-Acetyl-D-glucosamine-1-phosphate | C8H16NO9P | [M-H]- | Others |
| 371 | catechin-4-β-D-galactopyranoside* | C21H24O11 | [M-H]- | Flavonoids | 1340 | L-Fucose | C6H12O5 | [M-H]- | Others |
| 372 | Gallocatechin | C15H14O7 | [M+H]+ | Flavonoids | 1341 | D-Panose | C18H32O16 | [M-H]- | Others |
| 373 | Epicatechin-4'-O-β-D-glucopyranoside* | C21H24O11 | [M-H]- | Flavonoids | 1342 | D-Trehalose* | C12H22O11 | [M-H]- | Others |
| 374 | 3'-O-Methyl-epicatechin | C16H16O6 | [M+H]+ | Flavonoids | 1343 | Maltotriose | C18H32O16 | [M+Na]+ | Others |
| 375 | 8-[2-(3,4-dihydroxyphenyl)-3,5,7-trihydroxy-3,4-dihydro-2h-1-benzopyran-4-yl]-2-(4-hydroxyphenyl)-3,4-dihydro-2h-1-benzopyran-3,5,7-triol | C30H26O11 | [M+H]+ | Flavonoids | 1344 | Butyl Beta-D-Fructopyranoside | C10H20O6 | [M-H]- | Others |
| 376 | Fisetinidol-(4α,6)-gallocatechin | C30H26O12 | [M+H]+ | Flavonoids | 1345 | D-Arabinono-1,4-lactone* | C5H8O5 | [M-H]- | Others |
| 377 | Epicatechin-(2β→O→7,4β→8)-epiafzelechin-(4α→8)-epicatechin | C45H36O17 | [M-H]- | Flavonoids | 1346 | DMelezitose O-rhamnoside | C24H42O20 | [M-H]- | Others |
| 378 | Epicatechin-epiafzelechin | C30H26O11 | [M-H]- | Flavonoids | 1347 | 1,5-Anhydro-D-glucitol | C6H12O5 | [M-H]- | Others |
| 379 | Gallocatechin gallate | C22H18O11 | [M+H]+ | Flavonoids | 1348 | 1,6-anhydro-β-D-glucose | C6H10O5 | [M-H]- | Others |
| 380 | 8,8'-Methylenebiscatechin | C31H28O12 | [M+H]+ | Flavonoids | 1349 | Quebrachitol | C7H14O6 | [M-H]- | Others |
| 381 | Gallocatechin-(4α->8)-Catechin-(4α->8)-Catechin | C45H38O19 | [M+H]+ | Flavonoids | 1350 | D-Mannitol | C6H14O6 | [M-H]- | Others |
| 382 | Afzelechin (3,5,7,4'-Tetrahydroxyflavan) | C15H14O5 | [M+H]+ | Flavonoids | 1351 | 2-Dehydro-3-deoxy-L-arabinonate | C5H8O5 | [M-H]- | Others |
| 383 | 7,4'-Di-O-galloyltricetiflavan | C29H22O14 | [M-H]- | Flavonoids | 1352 | 1-O-Acetyl-Glucopyranose 6-Hydroxydecanoate | C18H32O9 | [M-H]- | Others |
| 384 | Eriodictyol-7-O-glucoside* | C21H22O11 | [M+H]+ | Flavonoids | 1353 | Solatriose | C18H32O15 | [M-H]- | Others |
| 385 | 6-C-Glucosyl-2-Hydroxynaringenin | C21H22O11 | [M-H]- | Flavonoids | 1354 | Stachyose | C24H42O21 | [M-H]- | Others |
| 386 | Eriodictyol-3'-O-glucoside* | C21H22O11 | [M+H]+ | Flavonoids | 1355 | Nystose | C24H42O21 | [M-H]- | Others |
| 387 | Eriodictyol-8-C-glucoside* | C21H22O11 | [M-H]- | Flavonoids | 1356 | N-Acetyl-D-galactosamine | C8H15NO6 | [M+H]+ | Others |
| 388 | 3',5,5',7-Tetrahydroxyflavanone-7-O-glucoside* | C21H22O11 | [M+H]+ | Flavonoids | 1357 | D-Glucurono-6,3-lactone | C6H8O6 | [M-H]- | Others |
| 389 | Choerospondin | C21H22O10 | [M-H]- | Flavonoids | 1358 | Melibiose | C12H22O11 | [M-H]- | Others |
| 390 | Hesperetin-3'-O-glucoside* | C22H24O11 | [M+H]+ | Flavonoids | 1359 | D-Arabinose | C5H10O5 | [M-H]- | Others |
| 391 | Butin-7-O-glucoside* | C21H22O10 | [M-H]- | Flavonoids | 1360 | D-Glucosamine | C6H13NO5 | [M+H]+ | Others |
| 392 | Hesperetin-7-O-glucoside* | C22H24O11 | [M+H]+ | Flavonoids | 1361 | D-Glucono-1,5-lactone | C6H10O6 | [M-H]- | Others |
| 393 | Isohemiphloin | C21H22O10 | [M-H]- | Flavonoids | 1362 | Glucopyranose 6-Hydroxydecanoate | C16H30O8 | [M+CH3COOH-H]- | Others |
| 394 | Malonyl isoSakuranin | C25H26O13 | [M+H]+ | Flavonoids | 1363 | beta-L-fucose 1-phosphate | C6H13O8P | [M+CH3COO]- | Others |
| 395 | Andrographidine A | C23H26O10 | [M+H]+ | Flavonoids | 1364 | L-Arabitol | C5H12O5 | [M-H]- | Others |
| 396 | Hesperetin-5-O-glucoside | C22H24O11 | [M-H]- | Flavonoids | 1365 | D-Ribose | C5H10O5 | [M-H]- | Others |
| 397 | Naringenin-7-O-glucoside (Prunin)* | C21H22O10 | [M-H]- | Flavonoids | 1366 | N-Acetyl-D-mannosamine* | C8H15NO6 | [M+H]+ | Others |
| 398 | Farrerol-7-O-glucoside | C23H26O10 | [M+H]+ | Flavonoids | 1367 | D-Fructose-1,6-biphosphate | C6H14O12P2 | [M-H]- | Others |
| 399 | Isookanin | C15H12O6 | [M+H]+ | Flavonoids | 1368 | Dihydroxyacetone phosphate | C3H7O6P | [M-H]- | Others |
| 400 | Butin; 7,3',4'-Trihydroxyflavanone* | C15H12O5 | [M+H]+ | Flavonoids | 1369 | D-Maltotetraose | C24H42O21 | [M-H]- | Others |
| 401 | Naringenin (5,7,4'-Trihydroxyflavanone)* | C15H12O5 | [M+H]+ | Flavonoids | 1370 | Butyl beta-D-glucoside | C10H20O6 | [M+H]+ | Others |
| 402 | Sakuranin | C22H24O10 | [M-H]- | Flavonoids | 1371 | Dambonitol | C8H16O6 | [M-H]- | Others |
| 403 | Cirsilineol (4',5-Dihydroxy-3',6,7-trimethoxyflavone) | C18H16O7 | [M-H]- | Flavonoids | 1372 | Manninotriose | C18H32O16 | [M-H]- | Others |
| 404 | (2S)-Abyssinone II* | C20H20O5 | [M-H]- | Flavonoids | 1373 | D-Glucosamine 1-phosphate | C6H14NO8P | [M-H]- | Others |
| 405 | Phellodensin D | C20H20O6 | [M-H]- | Flavonoids | 1374 | N-Acetyl-D-glucosamine* | C8H15NO6 | [M+H]+ | Others |
| 406 | Eriodictyol (5,7,3',4'-Tetrahydroxyflavanone) | C15H12O6 | [M+H]+ | Flavonoids | 1375 | Glucan | C18H32O16 | [M+H]+ | Others |
| 407 | Eriodictyol-7-O-Rutinoside (Eriocitrin) | C27H32O15 | [M-H]- | Flavonoids | 1376 | 2,3-Dihydroxypropanal | C3H6O3 | [M-H]- | Others |
| 408 | Eriodictyol-8-C-glucoside-4'-O-glucoside | C27H32O16 | [M+H]+ | Flavonoids | 1377 | Verbascose | C30H52O26 | [M-H]- | Others |
| 409 | 5,7,3',4',5'-Pentahydroxydihydroflavone | C15H12O7 | [M-H]- | Flavonoids | 1378 | 2-O-α-D-Glucopyranosyl-L-ascorbic acid | C12H18O11 | [M-H]- | Others |
| 410 | Poncirin (Isosakuranetin-7-O-neohesperidoside)* | C28H34O14 | [M-H]- | Flavonoids | 1379 | Isoascorbic acid 2-O-glucoside | C12H18O11 | [M-H]- | Others |
| 411 | Naringenin-7-O-Neohesperidoside(Naringin)* | C27H32O14 | [M-H]- | Flavonoids | 1380 | Riboflavin (Vitamin B2) | C17H20N4O6 | [M+H]+ | Others |
| 412 | Pinocembrin-7-O-glucoside (Pinocembroside) | C21H22O9 | [M+H]+ | Flavonoids | 1381 | 4-Pyridoxic acid | C8H9NO4 | [M+H]+ | Others |
| 413 | Liquiritigenin-7-O-apioside-4'-O-glucoside | C26H30O13 | [M+H]+ | Flavonoids | 1382 | D-Pantothenic Acid* | C9H17NO5 | [M-H]- | Others |
| 414 | Isosakuranetin (5,7-Dihydroxy-4'-methoxyflavanone) | C16H14O5 | [M+H]+ | Flavonoids | 1383 | Pyridoxine-5'-O-glucoside | C14H21NO8 | [M+H]+ | Others |
| 415 | Bavachinin | C21H22O4 | [M+H]+ | Flavonoids | 1384 | Nicotinamide | C6H6N2O | [M+H]+ | Others |
| 416 | Methylhesperidin | C29H36O15 | [M+H]+ | Flavonoids | 1385 | Nicotinate D-ribonucleoside | C11H14NO6+ | [M]+ | Others |
| 417 | Eriodictyol-7-O-(6''-O-galloyl)glucoside | C28H26O15 | [M-H]- | Flavonoids | 1386 | Erythorbic Acid; Isoascorbic Acid | C6H8O6 | [M-H]- | Others |
| 418 | Naringenin-7-O-Rutinoside-4'-O-glucoside | C33H42O19 | [M-H]- | Flavonoids | 1387 | Pyridoxine | C8H11NO3 | [M+H]+ | Others |
| 419 | 7-O-Methylnaringenin | C16H14O5 | [M+H]+ | Flavonoids | 1388 | N-(beta-D-Glucosyl)nicotinate | C12H15NO7 | [M+H]+ | Others |
| 420 | Cryptostrobin (8-C-Methyl-5,7-Dihydroxyflavanone) | C16H14O4 | [M-H]- | Flavonoids | 1389 | Orotic acid (Vitamin B13) | C5H4N2O4 | [M-H]- | Others |
| 421 | Didymin (Isosakuranetin-7-O-rutinoside)* | C28H34O14 | [M-H]- | Flavonoids | 1390 | Isonicotinic acid | C6H5NO2 | [M+H]+ | Others |
| 422 | Hesperetin-7-O-rutinoside (Hesperidin)* | C28H34O15 | [M+H]+ | Flavonoids | 1391 | Nicotinic acid (Vitamin B3) | C6H5NO2 | [M+H]+ | Others |
| 423 | Hesperetin-7-O-neohesperidoside(Neohesperidin)* | C28H34O15 | [M+H]+ | Flavonoids | 1392 | 4-Pyridoxic acid-O-glucoside | C14H19NO9 | [M+H]+ | Others |
| 424 | Naringenin-4',7-dimethyl ether | C17H16O5 | [M+H]+ | Flavonoids | 1393 | Dehydroascorbic acid | C6H6O6 | [M-H]- | Others |
| 425 | Naringenin-7-O-Rutinoside(Narirutin)* | C27H32O14 | [M-H]- | Flavonoids | 1394 | Pyridoxal | C8H9NO3 | [M+H]+ | Others |
| 426 | Eriodictyol-5,3'-Di-O-rutinoside | C39H52O24 | [M-H]- | Flavonoids | 1395 | L-Ascorbic acid (Vitamin C) | C6H8O6 | [M-H]- | Others |
| 427 | Bavachin | C20H20O4 | [M+H]+ | Flavonoids | 1396 | 6-Galloylglucosyl Aascorbic acid | C19H22O15 | [M-H]- | Others |
| 428 | Eriodictyol-7-O-glucoronide | C21H20O12 | [M+H]+ | Flavonoids | 1397 | Phylloquinone (Vitamin K1) | C31H46O2 | [M+H]+ | Others |
| 429 | Fustin | C15H12O6 | [M-H]- | Flavonoids | 1398 | Thiamine (Vitamin B1) | C12H17N4OS+ | [M]+ | Others |
| 430 | Taxifolin(Dihydroquercetin) | C15H12O7 | [M-H]- | Flavonoids | 1399 | Acitretin | C21H26O3 | [M+H]+ | Others |
| 431 | Dihydrokaempferide | C16H14O6 | [M+H]+ | Flavonoids | 1400 | 5-O-p-Coumaroylquinic acid | C16H18O8 | [M-H]- | Phenolic acids |
| 432 | Aromadendrin-7-O-glucoside* | C21H22O11 | [M-H]- | Flavonoids | 1401 | 5-(2-Hydroxyethyl)-2-O-glucosylphenol | C14H20O8 | [M-H]- | Phenolic acids |
| 433 | Dihydrokaempferol-3-O-glucoside* | C21H22O11 | [M+H]+ | Flavonoids | 1402 | 3,4-Dihydroxybenzeneacetic acid* | C8H8O4 | [M-H]- | Phenolic acids |
| 434 | Taxifolin-3-O-rhamnoside (Astilbin) | C21H22O11 | [M-H]- | Flavonoids | 1403 | Homogentisic acid* | C8H8O4 | [M-H]- | Phenolic acids |
| 435 | Taxifolin-2-O-glucoside | C21H22O12 | [M-H]- | Flavonoids | 1404 | Mucic acid Dimethyl Ester | C8H14O8 | [M-H]- | Phenolic acids |
| 436 | 3,5,7-Trihydroxyflavanone (Pinobanksin) | C15H12O5 | [M-H]- | Flavonoids | 1405 | 2-((5-carboxy-5-(((E)-3-(3-(((E)-3-(3,4-dihydroxyphenyl)acryloyl)oxy)-4-hydroxyphenyl)acryloyl)oxy)-2,3-dihydroxycyclohexyl)oxy)-1,3,4,5-tetrahydroxycyclohexane-1-carboxylic acid | C32H34O18 | [M-H]- | Phenolic acids |
| 437 | Silibinin | C25H22O10 | [M-H]- | Flavonoids | 1406 | Protocatechuic acid glucosyl xyloside | C18H24O13 | [M-H]- | Phenolic acids |
| 438 | Aromadendrin (Dihydrokaempferol) | C15H12O6 | [M-H]- | Flavonoids | 1407 | Iriflophenone-3-C-(2'',6''-Di-O-Galloyl)Glucoside | C33H28O18 | [M-H]- | Phenolic acids |
| 439 | Phellamurin | C26H30O11 | [M+H]+ | Flavonoids | 1408 | 2-(3,4-dihydroxyphenyl)ethanediol 1-O-β-D-glucopyranoside* | C14H20O9 | [M-H]- | Phenolic acids |
| 440 | Dihydromyricetin-3-O-glucoside | C21H22O13 | [M-H]- | Flavonoids | 1409 | Homosyringic Acid 4'-O-Glucoside | C16H22O10 | [M-H]- | Phenolic acids |
| 441 | Dihydromyricetin (Ampelopsin) | C15H12O8 | [M-H]- | Flavonoids | 1410 | Koaburaside* | C14H20O9 | [M-H]- | Phenolic acids |
| 442 | Engeletin | C21H22O10 | [M-H]- | Flavonoids | 1411 | 1-O-Feruloyl-β-D-glucose | C16H20O9 | [M-H]- | Phenolic acids |
| 443 | Luteolin-7-O-glucoside (Cynaroside)* | C21H20O11 | [M+H]+ | Flavonoids | 1412 | Glucosyringic acid | C15H20O10 | [M-H]- | Phenolic acids |
| 444 | Selagin | C16H12O7 | [M+H]+ | Flavonoids | 1413 | Gentisic acid 2-O-(6''-O-p-coumaroyl)Glucoside* | C22H22O11 | [M-H]- | Phenolic acids |
| 445 | Apigenin-7-O-rutinoside (Isorhoifolin)* | C27H30O14 | [M+H]+ | Flavonoids | 1414 | 1-O-Galloyl-2-O-Cinnamoyl-β-D-glucose* | C22H22O11 | [M-H]- | Phenolic acids |
| 446 | Apigenin-6-C-(2''-glucuronyl)xyloside | C26H26O15 | [M+H]+ | Flavonoids | 1415 | Ferulic acid-4-O-glucoside | C16H20O9 | [M-H]- | Phenolic acids |
| 447 | Apigenin-7-O-(6''-p-Coumaryl)glucoside | C30H26O12 | [M+H]+ | Flavonoids | 1416 | Protocatechuic acid 1-O-Rutinoside | C19H26O13 | [M-H]- | Phenolic acids |
| 448 | Apigenin-7-O-neohesperidoside (Rhoifolin)* | C27H30O14 | [M+H]+ | Flavonoids | 1417 | 6-O-Feruloyl-β-D-glucose | C16H20O9 | [M-H]- | Phenolic acids |
| 449 | Luteolin-3'-O-glucoside* | C21H20O11 | [M+H]+ | Flavonoids | 1418 | Chlorogenic acid (3-O-Caffeoylquinic acid)* | C16H18O9 | [M-H]- | Phenolic acids |
| 450 | Luteolin 7-O-p-coumaroyl rhamnoside* | C30H26O12 | [M-H]- | Flavonoids | 1419 | Vanilloloside | C14H20O8 | [M-H]- | Phenolic acids |
| 451 | Chrysin-7-O-glucoside | C21H20O9 | [M+H]+ | Flavonoids | 1420 | 5-Glucosyloxy-2-Hydroxybenzoic acid methyl ester* | C14H18O9 | [M-H]- | Phenolic acids |
| 452 | Isovitexin-2''-O-rhamnoside | C27H30O14 | [M+H]+ | Flavonoids | 1421 | Doitungbiphenyl A | C18H20O4 | [M-H]- | Phenolic acids |
| 453 | Diosmetin-7-O-rutinoside (Diosmin)* | C28H32O15 | [M+H]+ | Flavonoids | 1422 | 4-Nitrophenol | C6H5NO3 | [M+H]+ | Phenolic acids |
| 454 | Luteolin-7-O-(6''-malonyl)glucoside* | C24H22O14 | [M+H]+ | Flavonoids | 1423 | 1-O-Gentisoyl-β-D-glucoside | C13H16O9 | [M-H]- | Phenolic acids |
| 455 | Luteolin-6-C-glucoside (Isoorientin)* | C21H20O11 | [M+H]+ | Flavonoids | 1424 | Butyl isobutyl phthalate* | C16H22O4 | [M+H]+ | Phenolic acids |
| 456 | 3'-O-Methyltricetin-5-O-glucoside* | C22H22O12 | [M+H]+ | Flavonoids | 1425 | 10-acetylmonomelittoside* | C17H24O11 | [M-H]- | Phenolic acids |
| 457 | Luteolin 5-O-p-coumaroyl rhamnoside* | C30H26O12 | [M-H]- | Flavonoids | 1426 | Isotachioside* | C13H18O8 | [M-H]- | Phenolic acids |
| 458 | 6-C-MethylKaempferol-3-glucoside* | C22H22O11 | [M+H]+ | Flavonoids | 1427 | Neochlorogenic acid (5-O-Caffeoylquinic acid)* | C16H18O9 | [M+H]+ | Phenolic acids |
| 459 | Ladanetin | C16H12O6 | [M+H]+ | Flavonoids | 1428 | 1-O-Vanilloyl-D-Glucose* | C14H18O9 | [M-H]- | Phenolic acids |
| 460 | 5,7,8-Tetrahydroxy-6-methoxyflavone | C16H12O6 | [M+H]+ | Flavonoids | 1429 | Methyl Syringate | C10H12O5 | [M+H]+ | Phenolic acids |
| 461 | Hispidulin-7-O-glucoside(Homoplantaginin)* | C22H22O11 | [M+H]+ | Flavonoids | 1430 | Caffeic acid | C9H8O4 | [M-H]- | Phenolic acids |
| 462 | Pectolinarigenin-7-O-glucoside | C23H24O11 | [M+H]+ | Flavonoids | 1431 | Tachioside* | C13H18O8 | [M-H]- | Phenolic acids |
| 463 | Diosmetin-7-O-galactoside* | C22H22O11 | [M+H]+ | Flavonoids | 1432 | Chlorogenic acid methyl ester | C17H20O9 | [M-H]- | Phenolic acids |
| 464 | Jaceosidin-7-O-Glucoside* | C23H24O12 | [M+H]+ | Flavonoids | 1433 | 3-O-caffeoylshikimic acid | C16H16O8 | [M-H]- | Phenolic acids |
| 465 | Luteolin-4'-O-glucoside* | C21H20O11 | [M+H]+ | Flavonoids | 1434 | 5-Galloyl-6-O-Benzoyl Glucose | C20H20O11 | [M-H]- | Phenolic acids |
| 466 | Ladanetin-6-O-β-D-glucosid* | C22H22O11 | [M+H]+ | Flavonoids | 1435 | 1-O-p-Coumaroyl-β-D-glucose | C15H18O8 | [M-H]- | Phenolic acids |
| 467 | 6-Hydroxyluteolin 5-glucoside* | C21H20O12 | [M-H]- | Flavonoids | 1436 | Phthalic anhydride | C8H4O3 | [M+H]+ | Phenolic acids |
| 468 | Luteolin-8-C-glucoside (Orientin)* | C21H20O11 | [M+H]+ | Flavonoids | 1437 | 4-(3,4,5-Trihydroxybenzoxy)benzoic acid | C14H10O7 | [M-H]- | Phenolic acids |
| 469 | Yuanhuanin* | C22H22O11 | [M+H]+ | Flavonoids | 1438 | 1-O-(3,4-Dihydroxy-5-methoxy-benzoyl)-glucoside | C14H18O10 | [M-H]- | Phenolic acids |
| 470 | 4',5,7-Trihydroxy-3',6-dimethoxyflavone (Jaceosidin)* | C17H14O7 | [M+H]+ | Flavonoids | 1439 | 5-O-Caffeoylshikimic acid | C16H16O8 | [M-H]- | Phenolic acids |
| 471 | Leucocyanidin | C15H14O7 | [M-H]- | Flavonoids | 1440 | 2-O-P-Coumaroylhydroxycitric Acid | C15H14O10 | [M-H]- | Phenolic acids |
| 472 | Hypolaetin* | C15H10O7 | [M-H]- | Flavonoids | 1441 | galloyl xylosyl glucoside* | C18H24O14 | [M-H]- | Phenolic acids |
| 473 | Clitorin | C33H40O19 | [M+H]+ | Flavonoids | 1442 | mudanoside B* | C18H24O14 | [M-H]- | Phenolic acids |
| 474 | Apigenin-7-O-glucoside-4'-O-rutinoside | C33H40O19 | [M-H]- | Flavonoids | 1443 | Vanillic acid-4-O-glucoside | C14H18O9 | [M-H]- | Phenolic acids |
| 475 | Isoetin (5,7,2',4',5'-Pentahydroxyflavone)* | C15H10O7 | [M-H]- | Flavonoids | 1444 | Dunalianoside B | C21H22O11 | [M-H]- | Phenolic acids |
| 476 | 5,7-Dihydroxy-4-methoxyflavone-3-O-xylose-(1-6)-glucose | C27H30O15 | [M+H]+ | Flavonoids | 1445 | 6-O-Galloyl-β-D-glucose* | C13H16O10 | [M-H]- | Phenolic acids |
| 477 | Flavoyadorinin B | C23H24O11 | [M-H]- | Flavonoids | 1446 | 4-O-Glucosyl-sinapate | C17H22O10 | [M-H]- | Phenolic acids |
| 478 | 6-Hydroxyluteolin | C15H10O7 | [M-H]- | Flavonoids | 1447 | Rosmarinic acid-3'-O-glucoside | C24H26O13 | [M-H]- | Phenolic acids |
| 479 | Luteolin-6-C-arabinoside-7-O-glucoside | C26H28O15 | [M+H]+ | Flavonoids | 1448 | (2E)-3-[4-(β-D-glucopyranoside)-phenylacrylic]-acid | C15H18O8 | [M-H]- | Phenolic acids |
| 480 | Apigenin-7-O-(6''-acetyl)glucoside | C23H22O11 | [M+H]+ | Flavonoids | 1449 | Protocatechuic acid 1-O-(Glucosylvanilloyl)* | C21H22O12 | [M-H]- | Phenolic acids |
| 481 | Diosmetin-8-C-(2''-O-rhamnosyl)glucoside | C28H32O15 | [M+H]+ | Flavonoids | 1450 | Vanillic acid methyl ester* | C9H10O4 | [M+H]+ | Phenolic acids |
| 482 | Chrysoeriol-6-C-glucoside-4'-O-glucoside | C28H32O16 | [M+H]+ | Flavonoids | 1451 | 5-O-Galloyl-D-hamamelose* | C13H16O10 | [M-H]- | Phenolic acids |
| 483 | Oroxin A | C21H20O10 | [M+H]+ | Flavonoids | 1452 | 1-O-Salicyloyl-β-D-glucose | C13H16O8 | [M-H]- | Phenolic acids |
| 484 | Luteolin-7,3'-di-O-glucoside* | C27H30O16 | [M+H]+ | Flavonoids | 1453 | Salidroside | C14H20O7 | [M-H]- | Phenolic acids |
| 485 | Scutellarein (5,6,7,4'-Tetrahydroxyflavone) | C15H10O6 | [M+H]+ | Flavonoids | 1454 | 3,4,5-trihydroxy-5-[4-(prop-2-enoyloxymethyl)phenoxy]oxane-2-carboxylic acid | C16H18O9 | [M+H]+ | Phenolic acids |
| 486 | Hispidulin-7-O-(6''-O-p-Coumaroyl)Glucoside | C31H28O13 | [M+H]+ | Flavonoids | 1455 | 3-O-Galloyl-D-glucose* | C13H16O10 | [M-H]- | Phenolic acids |
| 487 | 4',5-Dihydroxy-3,3',7-Trimethoxyflavone; Pachypodol* | C18H16O7 | [M+H]+ | Flavonoids | 1456 | Nordihydrocapsiate | C17H26O4 | [M-H]- | Phenolic acids |
| 488 | Galangin-7-O-glucoside* | C21H20O10 | [M+H]+ | Flavonoids | 1457 | Dunalianoside A* | C21H22O10 | [M-H]- | Phenolic acids |
| 489 | Apigenin-6-C-fucoside | C21H20O9 | [M+H]+ | Flavonoids | 1458 | Phenoxyacetic acid | C8H8O3 | [M-H]- | Phenolic acids |
| 490 | Apigenin-7-O-glucoside(Cosmosiin)* | C21H20O10 | [M+H]+ | Flavonoids | 1459 | Phloroglucinol-1-O-β-D-glucopyranoside | C12H16O8 | [M-H]- | Phenolic acids |
| 491 | Apigenin-6-C-glucoside (Isovitexin)* | C21H20O10 | [M-H]- | Flavonoids | 1460 | 4-O-Glucosyl-3,4-dihydroxybenzyl alcohol | C13H18O8 | [M-H]- | Phenolic acids |
| 492 | Ayanin (3',5-Dihydroxy-3,4',7-Trimethoxyflavone)* | C18H16O7 | [M+H]+ | Flavonoids | 1461 | 2,6-dimethoxy-hydroquinone-4-O-β-D-glucopyranoside | C14H20O9 | [M-H]- | Phenolic acids |
| 493 | Vitexin-2''-O-rhamnoside | C27H30O14 | [M+H]+ | Flavonoids | 1462 | Grevilloside F | C15H18O9 | [M-H]- | Phenolic acids |
| 494 | 3',5',5,7-Tetrahydroxy-4'-methoxyflavanone-3'-O-glucoside | C22H24O12 | [M+H]+ | Flavonoids | 1463 | Protocatechuic acid 4-O-(6''-O-p-Coumaroyl)Glucoside | C22H22O11 | [M-H]- | Phenolic acids |
| 495 | Apigenin-8-C-Glucoside (Vitexin)* | C21H20O10 | [M-H]- | Flavonoids | 1464 | Grevilloside G* | C14H20O8 | [M-H]- | Phenolic acids |
| 496 | Tricin-4'-O-glucoside* | C23H24O12 | [M+H]+ | Flavonoids | 1465 | 2-O-Galloyl-D-glucose* | C13H16O10 | [M-H]- | Phenolic acids |
| 497 | Hispidulin-8-C-glucoside* | C22H22O11 | [M+H]+ | Flavonoids | 1466 | 1-O-(3,4,5-Trimethoxybenzoyl)-B-D-Glucopyranoside | C16H22O10 | [M-H]- | Phenolic acids |
| 498 | 5,2'-Dihydroxy-7,8-dimethoxyflavone glycoside* | C23H24O11 | [M+H]+ | Flavonoids | 1467 | davidioside C* | C15H16O9 | [M-H]- | Phenolic acids |
| 499 | Diosmetin-7-O-Neohesperidoside (Neodiosmin)* | C28H32O15 | [M+H]+ | Flavonoids | 1468 | Cimidahurinine* | C14H20O8 | [M-H]- | Phenolic acids |
| 500 | 3',4',5,7-Tetramethoxyflavone; Luteolin Tetramethyl Ether | C19H18O6 | [M+H]+ | Flavonoids | 1469 | 6-O-Caffeoylarbutin | C21H22O10 | [M-H]- | Phenolic acids |
| 501 | Luteolin-7-O-glucuronide-5-O-rhamnoside | C27H28O16 | [M+H]+ | Flavonoids | 1470 | 3,4-Dihydroxyphenethyl alcohol-8-O-[β-D-apinosyl(1→3)]-β-D-glucoside | C19H28O12 | [M-H]- | Phenolic acids |
| 502 | 3'-Demethylnobiletin | C20H20O8 | [M+H]+ | Flavonoids | 1471 | Dunalianoside C* | C21H22O11 | [M-H]- | Phenolic acids |
| 503 | Acacetin-7-O-galactoside | C22H22O10 | [M+H]+ | Flavonoids | 1472 | 2,3-Dihydroxy-1-(4-hydroxy-3,5-dimethoxyphenyl)propan-1-one | C11H14O6 | [M+H]+ | Phenolic acids |
| 504 | ageconyflavone C | C20H20O8 | [M+H]+ | Flavonoids | 1473 | Caffeoyl-O-mannitol | C15H20O9 | [M-H]- | Phenolic acids |
| 505 | 6-Methylflavone | C16H12O2 | [M+H]+ | Flavonoids | 1474 | Rhamnosyl-gentisic acid-5-O-β-D-glucoside | C19H26O13 | [M-H]- | Phenolic acids |
| 506 | Nepetin (5,7,3',4'-Tetrahydroxy-6-methoxyflavone) | C16H12O7 | [M+H]+ | Flavonoids | 1475 | Beta-asarone | C12H16O3 | [M+H]+ | Phenolic acids |
| 507 | Chrysoeriol-7-O-rutinoside-5-O-glucoside | C34H42O20 | [M+H]+ | Flavonoids | 1476 | Dimethyl phthalate | C10H10O4 | [M+H]+ | Phenolic acids |
| 508 | 5,7,3',4'-Tetrahydroxy-6-methoxyflavone-8-C-[glucosyl-(1-2)]-glucoside | C28H32O17 | [M+H]+ | Flavonoids | 1477 | Grevilloside L | C21H24O11 | [M-H]- | Phenolic acids |
| 509 | Apigenin; 4',5,7-Trihydroxyflavone | C15H10O5 | [M+H]+ | Flavonoids | 1478 | 1-(4-Hydroxybenzoyl)Glucose; 25545-07-7 | C13H16O8 | [M-H]- | Phenolic acids |
| 510 | Apigenin-6-C-arabinoside-8-C-xyloside* | C25H26O13 | [M+H]+ | Flavonoids | 1479 | 2,3-Dihydroxybenzoic Acid* | C7H6O4 | [M-H]- | Phenolic acids |
| 511 | Apigenin-6,8-di-C-arabinoside* | C25H26O13 | [M+H]+ | Flavonoids | 1480 | Coniferin | C16H22O8 | [M-H]- | Phenolic acids |
| 512 | Tetahydroxyflavone-7-O-glucuronide | C21H18O12 | [M-H]- | Flavonoids | 1481 | 3,4-Dihydroxybenzoic acid (Protocatechuic acid)* | C7H6O4 | [M-H]- | Phenolic acids |
| 513 | Orientin-7-O-glucoside | C27H30O16 | [M+H]+ | Flavonoids | 1482 | 1-Caffeoylquinic acid | C16H18O9 | [M-H]- | Phenolic acids |
| 514 | Vitexin-7-O-(6''-p-coumaroyl)glucoside | C36H36O17 | [M+H]+ | Flavonoids | 1483 | 1-O-p-Coumaroylquinic acid | C16H18O8 | [M-H]- | Phenolic acids |
| 515 | Meratin* | C27H30O17 | [M+H]+ | Flavonoids | 1484 | 2,4-Dihydroxybenzoic acid* | C7H6O4 | [M-H]- | Phenolic acids |
| 516 | Swertisin | C22H22O10 | [M+H]+ | Flavonoids | 1485 | 1-O-Caffeoyl-β-D-glucose | C15H18O9 | [M-H]- | Phenolic acids |
| 517 | Nobiletin (5,6,7,8,3',4'-Hexamethoxyflavone) | C21H22O8 | [M+H]+ | Flavonoids | 1486 | 2-Phenylethanol | C8H10O | [M-H2O+H]+ | Phenolic acids |
| 518 | Isovitexin-2''-O-xyloside | C26H28O14 | [M+H]+ | Flavonoids | 1487 | 4-Hydroxyphenyl 6-O-(2-Methyl-3-Hydroxypropionyl)-Beta-D-Galactopyranoside | C16H22O9 | [M-H]- | Phenolic acids |
| 519 | Apigenin-8-C-glucoside-7-O-Sophoroside | C33H40O20 | [M+H]+ | Flavonoids | 1488 | ((2R,3R,5R,6R)-6-(((E)-3-(3,4-dihydroxyphenyl)acryloyl)oxy)-3,4,5-trihydroxytetrahydro-2H-pyran-2-yl)methyl (Z)-3-(3,4-dihydroxyphenyl)acrylate | C24H24O12 | [M-H]- | Phenolic acids |
| 520 | 6,7,8-Tetrahydroxy-5-methoxyflavone* | C16H12O6 | [M+H]+ | Flavonoids | 1489 | 5'-Glucosyloxyjasmanic acid* | C18H28O9 | [M-H]- | Phenolic acids |
| 521 | Galangin (3,5,7-Trihydroxyflavone) | C15H10O5 | [M-H]- | Flavonoids | 1490 | Disinapoyl glucoside | C28H32O14 | [M-H]- | Phenolic acids |
| 522 | Chrysoeriol-7,4'-di-O-glucoside | C28H32O16 | [M+H]+ | Flavonoids | 1491 | 4-O-(3'-O-alpha-D-Glucopyranosyl)caffeoylquinic acid | C22H28O14 | [M-H]- | Phenolic acids |
| 523 | Tangeretin (4',5,6,7,8-Pentamethoxyflavone) | C20H20O7 | [M+H]+ | Flavonoids | 1492 | 3-(3-Hydroxyphenyl)-propionic acid | C9H10O3 | [M+H]+ | Phenolic acids |
| 524 | Acacetin-7-O-glucuronide | C26H20O8 | [M+H]+ | Flavonoids | 1493 | 6-O-Glucosyl-caffeoylbenzoic acid | C22H22O10 | [M-H]- | Phenolic acids |
| 525 | Luteolin-6,8-di-C-arabinoside | C25H26O14 | [M+H]+ | Flavonoids | 1494 | Cryptochlorogenic acid (4-O-Caffeoylquinic acid)* | C16H18O9 | [M-H]- | Phenolic acids |
| 526 | 5,7,2'-Trihydroxy-8-methoxyflavone* | C16H12O6 | [M-H]- | Flavonoids | 1495 | Benzyl β-primeveroside* | C18H26O10 | [M-H]- | Phenolic acids |
| 527 | Wogonin-7-O-Glucuronide (Wogonoside) | C22H20O11 | [M+H]+ | Flavonoids | 1496 | 1-O-Galloyl-β-D-glucose* | C13H16O10 | [M+H]+ | Phenolic acids |
| 528 | Dihydroxy-dimethoxyflavone-7-O-glucoside* | C23H24O11 | [M+H]+ | Flavonoids | 1497 | Benzyl B-Primeveroside* | C18H26O10 | [M-H]- | Phenolic acids |
| 529 | Diosmetin (5,7,3'-Trihydroxy-4'-methoxyflavone)* | C16H12O6 | [M-H]- | Flavonoids | 1498 | Benzyl-(2''-O-xylosyl)glucoside* | C18H26O10 | [M-H]- | Phenolic acids |
| 530 | Cirsiliol-8-C-(2''-glucosyl)glucoside | C29H34O17 | [M+H]+ | Flavonoids | 1499 | 3-O-(p-coumaroyl) 3-Hydroxy-3-methylglutaric acid* | C15H16O7 | [M+H]+ | Phenolic acids |
| 531 | Hydroxygenkwanin | C16H12O6 | [M+H]+ | Flavonoids | 1500 | Arbutin* | C12H16O7 | [M-H]- | Phenolic acids |
| 532 | Icariin | C33H40O15 | [M+H]+ | Flavonoids | 1501 | Anthranilate-1-O-Sophoroside | C19H27NO12 | [M-H]- | Phenolic acids |
| 533 | Apigenin-6-C-(2''-xylosyl)glucoside | C26H28O14 | [M+H]+ | Flavonoids | 1502 | Rosmarinic acid | C18H16O8 | [M+H]+ | Phenolic acids |
| 534 | Chrysoeriol-8-C-glucoside (Scoparin)* | C22H22O11 | [M+H]+ | Flavonoids | 1503 | 4-Hydroxybenzoic acid | C7H6O3 | [M+H]+ | Phenolic acids |
| 535 | 5,7,4'-Trihydroxy-3,6,3',5'-Tetramethoxyflavone | C19H18O9 | [M+H]+ | Flavonoids | 1504 | p-Hydroxypheny-β-D-allopyranoside* | C12H16O7 | [M-H]- | Phenolic acids |
| 536 | 5-Hydroxy-3,7,3',4'-tetramethoxyflavone (Retusin) | C19H18O7 | [M+H]+ | Flavonoids | 1505 | 3-Nitrophenol | C6H5NO3 | [M-H]- | Phenolic acids |
| 537 | Chrysoeriol-7-O-gentiobioside | C28H32O16 | [M+H]+ | Flavonoids | 1506 | 3-Hydroxy-5-Methylphenol-1-O-Glucoside | C13H18O7 | [M-H]- | Phenolic acids |
| 538 | Apigenin-6-C-glucoside-7-O-Sophoroside | C33H40O20 | [M+H]+ | Flavonoids | 1507 | Scroside D* | C20H30O13 | [M-H]- | Phenolic acids |
| 539 | Isoscutellarein | C15H10O6 | [M+H]+ | Flavonoids | 1508 | Syringin | C17H24O9 | [M-H]- | Phenolic acids |
| 540 | Chrysoeriol-8-C-arabinoside-7-O-Sophoroside | C33H40O20 | [M-H]- | Flavonoids | 1509 | Dihydroferulic acid glucoside | C16H22O9 | [M-H]- | Phenolic acids |
| 541 | Isovitexin-2''-O-(6'''-p-coumaroyl)glucoside | C36H36O17 | [M+H]+ | Flavonoids | 1510 | Phloroglucinol; 1,3,5-Benzenetriol | C6H6O3 | [M+H]+ | Phenolic acids |
| 542 | Luteolin-7-O-(6''-eudesmyl)glucoside | C31H30O15 | [M-H]- | Flavonoids | 1511 | 1-O-(p-coumaroyl) 3-Hydroxy-3-methylglutaric acid* | C15H16O7 | [M+H]+ | Phenolic acids |
| 543 | Apigenin-7-O-rutinoside-4'-O-rhamnoside | C33H40O18 | [M+H]+ | Flavonoids | 1512 | 2-Hydroxycinnamic acid* | C9H8O3 | [M-H]- | Phenolic acids |
| 544 | Cirsiliol-8-C-glucoside | C23H24O12 | [M+H]+ | Flavonoids | 1513 | Elemicin* | C12H16O3 | [M+H]+ | Phenolic acids |
| 545 | Chrysoeriol-7-O-[2''-O-(2'''-Sinapoyl)glucuronyl]glucuronide | C39H38O22 | [M+H]+ | Flavonoids | 1514 | 4-O-p-Coumaroylquinic acid | C16H18O8 | [M-H]- | Phenolic acids |
| 546 | Isosaponarin(Isovitexin-4'-O-glucoside)* | C27H30O15 | [M+H]+ | Flavonoids | 1515 | 6-O-Caffeoyl-D-glucose | C15H18O9 | [M-H]- | Phenolic acids |
| 547 | Vitexin-2''-O-glucoside* | C27H30O15 | [M+H]+ | Flavonoids | 1516 | Raspberryketone glucoside | C16H22O7 | [M-H]- | Phenolic acids |
| 548 | Luteolin (5,7,3',4'-Tetrahydroxyflavone) | C15H10O6 | [M-H]- | Flavonoids | 1517 | Dihydrocaffeoylglucose* | C15H20O9 | [M-H]- | Phenolic acids |
| 549 | Luteolin-5,7-di-O-rutinoside* | C39H50O24 | [M+H]+ | Flavonoids | 1518 | 3-O-galloyl-beta-d-glucose* | C13H16O10 | [M+H]+ | Phenolic acids |
| 550 | Chrysoeriol-5,7-di-O-rutinoside | C40H52O24 | [M+H]+ | Flavonoids | 1519 | Dicaffeoylquinic acid-O-glucoside | C31H34O17 | [M-H]- | Phenolic acids |
| 551 | Luteolin-7-O-(2''''-O-rhamnosyl)sophorotrioside | C42H46O23 | [M+H]+ | Flavonoids | 1520 | 2-β-D-Glucopyranosyloxy-5-hydroxy-phenylacetic acid | C14H18O9 | [M-H]- | Phenolic acids |
| 552 | Chrysoeriol-5,7-di-O-glucoside | C28H32O16 | [M+H]+ | Flavonoids | 1521 | 2,4-Dinitrophenol | C6H4N2O5 | [M-H]- | Phenolic acids |
| 553 | Chrysoeriol-7-O-(6''-acetyl)glucoside* | C24H24O12 | [M-H]- | Flavonoids | 1522 | 3'-O-Beta-D-Glucopyranosyl plumbagic acid | C17H22O10 | [M-H]- | Phenolic acids |
| 554 | Apigenin-6-C-(2''-glucosyl)arabinoside | C26H28O14 | [M+H]+ | Flavonoids | 1523 | 1-O-Feruloyl-3-O-caffeoylglycerol | C22H22O9 | [M-H]- | Phenolic acids |
| 555 | Apigenin-7-O-(6''-malonyl)glucoside | C24H22O13 | [M+H]+ | Flavonoids | 1524 | 4-hydroxy-3-(2-hydroxy-3-methylbut-3-en-1-yl)benzoic acid | C12H14O4 | [M+H]+ | Phenolic acids |
| 556 | Apigenin-7-O-Gentiobioside | C27H30O15 | [M+H]+ | Flavonoids | 1525 | 1-(4-Methoxyphenyl)-1-propanol | C10H14O2 | [M-H]- | Phenolic acids |
| 557 | Apigenin-7-O-rutinoside-4'-O-rutinoside | C39H50O23 | [M+H]+ | Flavonoids | 1526 | Populoside | C22H24O10 | [M-H]- | Phenolic acids |
| 558 | Isovitexin-8-O-xyloside | C26H28O14 | [M+H]+ | Flavonoids | 1527 | 2,4-Di-Tert-Butylphenol | C14H22O | [M-H]- | Phenolic acids |
| 559 | Isovitexin-7-O-glucoside(Saponarin)* | C27H30O15 | [M+H]+ | Flavonoids | 1528 | Benzyl 3,6-dimethoxy-2-{[3,4,5-trihydroxy-6-(hydroxymethyl)oxan-2-yl]oxy}benzoate | C22H26O10 | [M-H]- | Phenolic acids |
| 560 | 5,7-Dihydroxy-3',4',5'-trimethoxyflavone | C18H16O7 | [M+H]+ | Flavonoids | 1529 | 3-Hydroxycinnamic Acid* | C9H8O3 | [M-H]- | Phenolic acids |
| 561 | Luteolin-6-C-(5''-glucuronyl)xyloside | C26H26O16 | [M+H]+ | Flavonoids | 1530 | 2,6-Dihydroxy-4-isopropylphenyl-1-O-β-D-glucoside | C15H22O8 | [M+H]+ | Phenolic acids |
| 562 | Typhaneoside | C34H42O20 | [M+H]+ | Flavonoids | 1531 | 1'-O-(3,4-Dihydroxyphenethyl)-O-caffeoyl-glucoside | C23H26O11 | [M-H]- | Phenolic acids |
| 563 | Sudachitin | C18H16O8 | [M-H]- | Flavonoids | 1532 | decaffeoylverbascoside | C20H30O12 | [M-H]- | Phenolic acids |
| 564 | Chrysoeriol-7-O-(6''-malonyl)glucoside | C25H24O14 | [M+H]+ | Flavonoids | 1533 | Methyleugenol | C11H14O2 | [M+H]+ | Phenolic acids |
| 565 | 4'-O-Glucosylvitexin | C27H30O15 | [M+H]+ | Flavonoids | 1534 | Salirepin | C13H18O8 | [M-H]- | Phenolic acids |
| 566 | Vitexin-7-O-(6''-feruloyl)glucoside | C37H38O18 | [M+H]+ | Flavonoids | 1535 | Grevilloside C | C17H24O9 | [M+H]+ | Phenolic acids |
| 567 | Apigenin-6,8-di-C-glucoside-4'-O-glucoside | C33H40O20 | [M+H]+ | Flavonoids | 1536 | Sanguisorbic acid dilactone | C21H10O13 | [M+H]+ | Phenolic acids |
| 568 | Tricin-7-O-Glucuronide | C23H22O13 | [M-H]- | Flavonoids | 1537 | Androsin | C15H20O8 | [M-H]- | Phenolic acids |
| 569 | Luteolin-7-O-(6''-malonyl)glucoside-5-O-arabinoside | C29H30O18 | [M+H]+ | Flavonoids | 1538 | gentisic acid 5-O-β-D-(6'-O-galloyl)-gluco-pyranoside | C20H20O13 | [M-H]- | Phenolic acids |
| 570 | Apigenin-8-C-glucoside-7-O-(6''-sinapoyl)glucoside | C38H40O19 | [M+H]+ | Flavonoids | 1539 | (S)-2-Phenyloxirane | C8H8O | [M+H]+ | Phenolic acids |
| 571 | Luteolin-7-O-(6'''-malonyl)sophoroside-5-O-arabinoside | C35H40O23 | [M+H]+ | Flavonoids | 1540 | Syringaldehyde-4-O-glucoside* | C15H20O9 | [M-H]- | Phenolic acids |
| 572 | Hispidulin-8-C-(2''-O-xylosyl)xyloside | C26H28O14 | [M+H]+ | Flavonoids | 1541 | 4,6-dimethoxy-2-methoxyphenyl-1-O-beta-D-glucopyranoside | C13H18O9 | [M+H]+ | Phenolic acids |
| 573 | Apigenin-7-O-rutinoside-4'-O-Sophoroside* | C39H50O24 | [M+H]+ | Flavonoids | 1542 | Protocatechuic acid 4-O-(6''-O-Galloy)Glucoside | C20H20O13 | [M-H]- | Phenolic acids |
| 574 | Mearnsetin-3-O-glucuronide | C22H20O14 | [M-H]- | Flavonoids | 1543 | 2-Hydroxy-3,5-dinitrobenzoic acid | C7H4N2O7 | [M-H]- | Phenolic acids |
| 575 | Apigenin-6-C-(2''-rhamnosyl)glucoside | C27H30O14 | [M+H]+ | Flavonoids | 1544 | β-Hydroxy-(3,4-dihydroxyphenylethanolyl)-glucoside | C14H20O9 | [M-H]- | Phenolic acids |
| 576 | Luteolin-6-C-(2''-glucuronyl)glucoside | C27H28O17 | [M+H]+ | Flavonoids | 1545 | Ethyl phenylacetate | C10H12O2 | [M+H]+ | Phenolic acids |
| 577 | Chrysoeriol-7-O-(6''-sinapoyl)glucoside | C33H32O15 | [M+H]+ | Flavonoids | 1546 | 5-Acetylsalicylic acid | C9H8O4 | [M+H]+ | Phenolic acids |
| 578 | Vitexin-2''-O-galactoside* | C27H30O15 | [M+H]+ | Flavonoids | 1547 | 2-O-Salicyl-6-O-Galloyl-D-Glucose | C20H20O12 | [M-H]- | Phenolic acids |
| 579 | Apigenin-8-C-Arabinoside | C20H18O9 | [M+H]+ | Flavonoids | 1548 | 2'-O-β-D-Glucopyranosylsalicin | C19H28O12 | [M-H]- | Phenolic acids |
| 580 | Baicalin* | C21H18O11 | [M+H]+ | Flavonoids | 1549 | Curculigine | C23H28O12 | [M+H]+ | Phenolic acids |
| 581 | Cirsimaritin-8-C-glucoside | C23H24O11 | [M+H]+ | Flavonoids | 1550 | 1-O-Eudesmoylquinic acid | C17H22O10 | [M-H]- | Phenolic acids |
| 582 | Apigenin-6-C-(2''-glucuronyl)glucoside | C27H28O16 | [M+H]+ | Flavonoids | 1551 | Trollioside | C19H26O9 | [M+H]+ | Phenolic acids |
| 583 | Tricin-4'-O-rutinoside-7-O-rutinoside | C41H54O25 | [M+H]+ | Flavonoids | 1552 | 1-O-Caffeoyl-(6-O-glucosyl)-β-D-glucose | C21H28O14 | [M-H]- | Phenolic acids |
| 584 | Acerosin | C18H16O8 | [M-H]- | Flavonoids | 1553 | Methyl 3-(3-hydroxy-4-methoxyphenyl)propanoate | C11H14O4 | [M-H]- | Phenolic acids |
| 585 | Chrysoeriol-7-O-Sophoroside-5-O-glucuronide | C34H40O22 | [M-H]- | Flavonoids | 1554 | Demethyl coniferin | C15H20O8 | [M-H]- | Phenolic acids |
| 586 | Tricin-7-O-neohesperidoside | C29H34O16 | [M+H]+ | Flavonoids | 1555 | 2-Nitrophenol | C6H5NO3 | [M-H]- | Phenolic acids |
| 587 | Isoluteolin-6,8-di-C-glucoside* | C27H30O16 | [M+H]+ | Flavonoids | 1556 | Caffeoyl(p-Hydroxybenzoyl)tartaric acid | C20H16O11 | [M-H]- | Phenolic acids |
| 588 | Chrysoeriol-8-C-glucoside-7-O-(6''-feruloyl)glucoside | C38H40O19 | [M+H]+ | Flavonoids | 1557 | 2,4,6-Trihydroxybenzoic acid | C7H6O5 | [M+H]+ | Phenolic acids |
| 589 | Lysionotin-C-Xylosyl-glucoside | C29H34O16 | [M+H]+ | Flavonoids | 1558 | Methyl caffeate | C10H10O4 | [M-H]- | Phenolic acids |
| 590 | Chrysoeriol-6-C-glucoside-4'-O-(6''-sinapoyl)glucoside | C39H42O20 | [M+H]+ | Flavonoids | 1559 | Benzyl-β-gentiobioside* | C19H28O11 | [M-H]- | Phenolic acids |
| 591 | Isovitexin-2''-O-(6'''-feruloyl)glucoside | C37H38O18 | [M+H]+ | Flavonoids | 1560 | p-Coumaric acid | C9H8O3 | [M+H]+ | Phenolic acids |
| 592 | Tricin-7-O-(2''-O-rhamnosyl)galacturonide | C29H32O17 | [M+H]+ | Flavonoids | 1561 | Rhododendrol | C10H14O2 | [M+H]+ | Phenolic acids |
| 593 | Luteolin-7-O-(6''-malonyl)glucuronide-5-O-rhamnoside | C30H30O19 | [M+H]+ | Flavonoids | 1562 | 3-Hydroxy-4-isopropylbenzylalcohol-3-O-glucoside | C16H24O7 | [M-H]- | Phenolic acids |
| 594 | Apigenin-7-O-(2''-glucurosyl)glucuronide | C27H26O17 | [M+H]+ | Flavonoids | 1563 | 2,5-Dihydroxyphenylacetate ethyl triglucoside | C28H42O18 | [M-H]- | Phenolic acids |
| 595 | Apigenin-7-O-glucuronide* | C21H18O11 | [M+H]+ | Flavonoids | 1564 | Quinacyl syringic acid | C16H20O10 | [M-H]- | Phenolic acids |
| 596 | Luteolin-7-O-gentiobioside* | C27H30O16 | [M+H]+ | Flavonoids | 1565 | 3,5-Dihydroxytoluene | C7H8O2 | [M+H]+ | Phenolic acids |
| 597 | Kaempferol-3,7-O-dirhamnoside (Kaempferitrin) | C27H30O14 | [M+H]+ | Flavonoids | 1566 | 3-Prenyl-4-O-glucosyloxy-4-hydroxybenzoic acid | C18H24O8 | [M-H]- | Phenolic acids |
| 598 | Quercetin-3-O-robinobioside | C27H30O16 | [M-H]- | Flavonoids | 1567 | Caffeoyl-p-coumaroyltartaric acid | C22H18O11 | [M-H]- | Phenolic acids |
| 599 | Rhamnetin-3-O-Glucoside* | C22H22O12 | [M+H]+ | Flavonoids | 1568 | 2-Methoxy-4-ethenylphenol | C9H10O2 | [M+H]+ | Phenolic acids |
| 600 | 8-Methoxykaempferol-7-O-rhamnoside* | C22H22O11 | [M-H]- | Flavonoids | 1569 | 4-hydroxy-3-methoxyphenyl 1-O-β-D-(6'-O-galloyol)-glucopyranoside* | C20H22O12 | [M-H]- | Phenolic acids |
| 601 | Isorhamnetin-3-O-Glucoside* | C22H22O12 | [M+H]+ | Flavonoids | 1570 | Mucic acid-1,4-lactone-3,5-di-O-gallate | C20H16O15 | [M-H]- | Phenolic acids |
| 602 | Morin-3-O-lyxoside | C20H18O11 | [M-H]- | Flavonoids | 1571 | 3,4-dihydroxy-allylbenzene4-O-p-D-xylopyranosyI-(1-→6)-β-D-glucopyranoside* | C20H28O11 | [M-H]- | Phenolic acids |
| 603 | Isorhamnetin-3-O-(6''-malonyl)glucoside* | C25H24O15 | [M+H]+ | Flavonoids | 1572 | 3,4'-Dihydroxypropiophenone glucoside* | C15H20O8 | [M+H]+ | Phenolic acids |
| 604 | Quercetin-3-O-(6''-O-acetyl)galactoside | C23H22O13 | [M-H]- | Flavonoids | 1573 | 4,6-(S)-Hexahydroxydiphenoyl-β-D-glucose | C20H18O14 | [M-H]- | Phenolic acids |
| 605 | Quercetin-5-O-β-D-glucoside | C21H20O12 | [M+H]+ | Flavonoids | 1574 | Vnilloylcaffeoyltartaric acid | C21H18O12 | [M-H]- | Phenolic acids |
| 606 | Kaempferol-7-O-glucoside* | C21H20O11 | [M-H]- | Flavonoids | 1575 | 1-O-Galloyl-rhamnose | C13H16O9 | [M+H]+ | Phenolic acids |
| 607 | Tamarixetin-3-O-(6''-malonyl)glucoside* | C25H24O15 | [M+H]+ | Flavonoids | 1576 | 3,5-bis(2-hydroxy-3-methylbut-3-en-1-yl)-4-((3,4,5-trihydroxy-6-(hydroxymethyl)tetrahydro-2H-pyran-2-yl)oxy)benzoic acid | C23H32O10 | [M-H]- | Phenolic acids |
| 608 | 6-Hydroxykaempferol-7-O-glucoside | C21H20O12 | [M+H]+ | Flavonoids | 1577 | Phenylmethyl β-L-glucopyranoside | C13H18O6 | [M-H]- | Phenolic acids |
| 609 | Kaempferol-3-O-galactoside (Trifolin)* | C21H20O11 | [M-H]- | Flavonoids | 1578 | 2,3,4-Trihydroxybenzoic acid | C7H6O5 | [M-H]- | Phenolic acids |
| 610 | Robinetin | C15H10O7 | [M+H]+ | Flavonoids | 1579 | 1,7-bis(4-hydroxy-3-methoxyphenyl)hept-1-ene-3-ol | C21H26O5 | [M+H]+ | Phenolic acids |
| 611 | Tamarixetin-3-O-glucoside (Tamarixin)* | C22H22O12 | [M+H]+ | Flavonoids | 1580 | 1-(2,4,5-Trimethoxyphenyl)-1,2-propanedione | C12H14O5 | [M+H]+ | Phenolic acids |
| 612 | Galloylisorhamnetin | C23H16O11 | [M+H]+ | Flavonoids | 1581 | 1,3-O-Dicaffeoylglycerol | C21H20O9 | [M+H]+ | Phenolic acids |
| 613 | Tamarixetin-3-O-rutinoside | C28H32O16 | [M-H]- | Flavonoids | 1582 | Salvianolic acid N | C26H22O10 | [M-H]- | Phenolic acids |
| 614 | 6-C-Methylquercetin-3-O-glucoside* | C22H22O12 | [M+H]+ | Flavonoids | 1583 | 2-Amino-3-methoxybenzoic acid | C8H9NO3 | [M+H]+ | Phenolic acids |
| 615 | Quercetin-3-O-rutinoside (Rutin)* | C27H30O16 | [M+H]+ | Flavonoids | 1584 | Manglieside B* | C20H28O11 | [M-H]- | Phenolic acids |
| 616 | Quercetin-3-O-(4''-O-glucosyl)rhamnoside* | C27H30O16 | [M+H]+ | Flavonoids | 1585 | 1,6-Di-O-caffeoyl-β-D-glucose | C24H24O12 | [M-H]- | Phenolic acids |
| 617 | Kaempferol-3-O-(4''-p-coumaroyl)rhamnoside* | C30H26O12 | [M-H]- | Flavonoids | 1586 | 2,3,4-Trihydroxybutyl 6-O-(E)-caffeoyl-β-D-glucopyranoside | C19H26O12 | [M+H]+ | Phenolic acids |
| 618 | Kaempferol-3-O-robinobioside(Biorobin)* | C27H30O15 | [M-H]- | Flavonoids | 1587 | 3,5-Dihydroxyacetophenone | C8H8O3 | [M-H]- | Phenolic acids |
| 619 | Kaempferol-3-O-(6''-malonyl)galactoside* | C24H22O14 | [M+H]+ | Flavonoids | 1588 | Sibiricose A3 | C19H26O13 | [M-H]- | Phenolic acids |
| 620 | Sexangularetin-3-O-glucoside-7-O-rhamnoside | C28H32O16 | [M+H]+ | Flavonoids | 1589 | 2-(Formylamino)benzoic acid | C8H7NO3 | [M-H]- | Phenolic acids |
| 621 | Quercetin-3-O-glucoside-7-O-rhamnoside* | C27H30O16 | [M+H]+ | Flavonoids | 1590 | 1-O-(6'-O-feruloyl)glucoside-3-O-Caffeoyl Quinic Acid | C32H36O17 | [M-H]- | Phenolic acids |
| 622 | Quercetin-7-O-rutinoside* | C27H30O16 | [M+H]+ | Flavonoids | 1591 | Methyl 4-hydroxybenzoate* | C8H8O3 | [M-H]- | Phenolic acids |
| 623 | Amoenin | C21H20O11 | [M+H]+ | Flavonoids | 1592 | 3,5-Dicaffeoylquinic acid | C25H24O12 | [M-H]- | Phenolic acids |
| 624 | Morin-3-O-xyloside | C20H18O11 | [M+H]+ | Flavonoids | 1593 | Robustaside A;[6'-p-Coumarylarbutin] | C21H22O9 | [M-H]- | Phenolic acids |
| 625 | Quercetin 7-O-p-coumaroyl rhamnoside* | C30H26O13 | [M-H]- | Flavonoids | 1594 | (S)-2-Hydroxy-3-(4-Hydroxyphenyl)Propanoic Acid* | C9H10O4 | [M-H]- | Phenolic acids |
| 626 | Isorhamnetin 3-galactoside | C22H22O12 | [M-H]- | Flavonoids | 1595 | 4-O-(6'-O-Glucosylcaffeoyl)-4-hydroxybenzoic acid | C22H22O11 | [M-H]- | Phenolic acids |
| 627 | Quercetin-3-O-(2''-O-rhamnosyl)galactoside | C27H30O16 | [M+H]+ | Flavonoids | 1596 | 3,4-Divanillyltetrahydrofuran | C20H24O5 | [M+H]+ | Phenolic acids |
| 628 | Quercetin 5-O-p-coumaroyl rhamnoside* | C30H26O13 | [M-H]- | Flavonoids | 1597 | Cimicifugic acid E-glucose-rhamnose* | C33H40O19 | [M+H]+ | Phenolic acids |
| 629 | Kaempferol-3-O-rhamnosyl(1→2)glucoside | C27H30O15 | [M+H]+ | Flavonoids | 1598 | 2-Methoxy-4-methylphenol | C8H10O2 | [M+H]+ | Phenolic acids |
| 630 | Kaempferol-3-O-(6''-malonyl)glucoside* | C24H22O14 | [M+H]+ | Flavonoids | 1599 | 3,4,5-Trihydroxy-6-(hydroxymethyl)oxan-2-yl2-hydroxy-5-methylbenzoate | C14H18O8 | [M-H]- | Phenolic acids |
| 631 | Kaempferol-3-O-(6''-O-acetyl)glucoside | C23H22O12 | [M-H]- | Flavonoids | 1600 | 2-Feruloyl-sn-glycerol* | C13H16O6 | [M+H]+ | Phenolic acids |
| 632 | Quercetin-4'-O-glucoside (Spiraeoside)* | C21H20O12 | [M-H]- | Flavonoids | 1601 | Sinapinaldehyde | C11H12O4 | [M+H]+ | Phenolic acids |
| 633 | Quercetin-7-O-glucoside* | C21H20O12 | [M-H]- | Flavonoids | 1602 | 2-O-(4-carboxylic acid phenethyl)-6-O-caffeoyl glucoside | C23H24O11 | [M-H]- | Phenolic acids |
| 634 | Kaempferol-3-O-rutinoside(Nicotiflorin)* | C27H30O15 | [M-H]- | Flavonoids | 1603 | (E)-caffeyl alcohol 4-O-β-D-glucopyranoside | C15H20O8 | [M-H]- | Phenolic acids |
| 635 | Juglanin* | C20H18O10 | [M+H]+ | Flavonoids | 1604 | 6'-O-Feruloyl-D-sucrose | C22H30O14 | [M-H]- | Phenolic acids |
| 636 | Quercetin-3-O-galactoside (Hyperin) | C21H20O12 | [M-H]- | Flavonoids | 1605 | 2-O-(4-Hydroxybenzoyl)-6-O-(Galloyl)-Beta-D-Glucopyranose | C20H20O12 | [M-H]- | Phenolic acids |
| 637 | Kaempferol-3-O-(6''-galloyl)galactoside* | C28H24O15 | [M+H]+ | Flavonoids | 1606 | galloyl acetyl glucoside | C15H18O11 | [M-H]- | Phenolic acids |
| 638 | Kaempferol-3-O-arabinoside* | C20H18O10 | [M+H]+ | Flavonoids | 1607 | 3-O-p-Coumaroylshikimic acid-O-glucoside | C22H26O12 | [M-H]- | Phenolic acids |
| 639 | Quercetin-3-O-glucoside (Isoquercitrin) | C21H20O12 | [M-H]- | Flavonoids | 1608 | 3-(4-Hydroxy-3-methoxyphenyl)-1,2-propanediol | C10H14O4 | [M+H]+ | Phenolic acids |
| 640 | Azaleatin (5-O-Methylquercetin) | C16H12O7 | [M+H]+ | Flavonoids | 1609 | Methyl 4,6-di-O-galloyl-D-glucoside | C21H22O14 | [M-H]- | Phenolic acids |
| 641 | Rehderianin I* | C17H14O7 | [M+H]+ | Flavonoids | 1610 | Ferulic acid | C10H10O4 | [M-H]- | Phenolic acids |
| 642 | Kaempferol-3-O-(2''-galloyl)galactoside* | C28H24O15 | [M+H]+ | Flavonoids | 1611 | Malonyl Tachioside | C16H20O11 | [M-H]- | Phenolic acids |
| 643 | Isorhamnetin-3-O-arabinoside | C21H20O11 | [M+H]+ | Flavonoids | 1612 | Hydroxytyrosol | C8H10O3 | [M-H]- | Phenolic acids |
| 644 | Kaempferol-3-O-(6''-galloyl)glucoside* | C28H24O15 | [M+H]+ | Flavonoids | 1613 | 3-Hydroxy-4-methoxybenzoic acid; Isovanillic Acid | C8H8O4 | [M+H]+ | Phenolic acids |
| 645 | Quercetin-3-O-(2''-O-acetyl)glucuronide | C23H20O14 | [M-H]- | Flavonoids | 1614 | Benzyl-(2''-O-glucosyl)glucoside* | C19H28O11 | [M-H]- | Phenolic acids |
| 646 | Kaempferol-3-O-sophoroside-7-O-rhamnoside* | C33H40O20 | [M+H]+ | Flavonoids | 1615 | 2-Hydroxybenzaldehyde (Salicylaldehyde) | C7H6O2 | [M-H]- | Phenolic acids |
| 647 | Kaempferol-3-O-neohesperidoside-7-O-glucoside* | C33H40O20 | [M+H]+ | Flavonoids | 1616 | 3,4,5-Trimethoxycinnamic acid | C12H14O5 | [M-H]- | Phenolic acids |
| 648 | Gossypetin-3-O-glucoside* | C21H20O13 | [M+H]+ | Flavonoids | 1617 | 4-O-(6'-O-Glucosylcaffeoylglucosyl)-4-hydroxybenzyl alcohol | C28H34O15 | [M-H]- | Phenolic acids |
| 649 | Myricetin-3-O-galactoside* | C21H20O13 | [M+H]+ | Flavonoids | 1618 | Specnuezhenide | C31H42O17 | [M-H]- | Phenolic acids |
| 650 | Kaempferol-3-O-arabinoside (Juglanin) | C20H18O10 | [M-H]- | Flavonoids | 1619 | Hematoxylin | C16H14O6 | [M+H]+ | Phenolic acids |
| 651 | Maohuoside A | C27H32O12 | [M+H]+ | Flavonoids | 1620 | Brevifolin carboxylic acid | C13H8O8 | [M-H]- | Phenolic acids |
| 652 | Fisetin | C15H10O6 | [M+H]+ | Flavonoids | 1621 | Cis-Coutaric acid | C13H12O8 | [M-H]- | Phenolic acids |
| 653 | Kaempferol-3-O-rhamnoside (Afzelin)(Kaempferin)* | C21H20O10 | [M-H]- | Flavonoids | 1622 | Pyrogallol | C6H6O3 | [M-H]- | Phenolic acids |
| 654 | Kaempferol-3-(2'',6''-di-O-rhamnosyl)-glucoside | C33H40O19 | [M+H]+ | Flavonoids | 1623 | Calceolarioside D* | C23H26O11 | [M+H]+ | Phenolic acids |
| 655 | Malonyl Amurensin | C29H30O14 | [M-H]- | Flavonoids | 1624 | Maplexin C (2,3-Di-O-Galloyl-1,5-Anhydro-D-Glucitol)* | C20H20O13 | [M-H]- | Phenolic acids |
| 656 | Quercetin-3-O-(6''-O-galloyl)galactoside | C28H24O16 | [M+H]+ | Flavonoids | 1625 | Sinapic acid | C11H12O5 | [M-H]- | Phenolic acids |
| 657 | Kaempferol-7-O-rhamnoside* | C21H20O10 | [M-H]- | Flavonoids | 1626 | Cinnamaldehyde | C9H8O | [M+H]+ | Phenolic acids |
| 658 | Myricetin | C15H10O8 | [M+H]+ | Flavonoids | 1627 | Desrhamnosylacteoside* | C23H26O11 | [M+H]+ | Phenolic acids |
| 659 | Kaempferol-3-O-(2''-O-xylosyl-6''-O-rhamnosyl)glucoside | C32H38O19 | [M+H]+ | Flavonoids | 1628 | Arenarioside | C34H44O19 | [M-H]- | Phenolic acids |
| 660 | Isorhamnetin-3-O-sophoroside | C28H32O17 | [M+H]+ | Flavonoids | 1629 | 4-Hydroxy-3,5-diisopropylbenzaldehyde | C13H18O2 | [M+H]+ | Phenolic acids |
| 661 | Laricitrin-3-O-glucoside* | C22H22O13 | [M+H]+ | Flavonoids | 1630 | Protocatechuic Acid Methyl Ester | C8H8O4 | [M-H]- | Phenolic acids |
| 662 | Kaempferol-3,7-O-diglucoside* | C27H30O16 | [M+H]+ | Flavonoids | 1631 | Feruloylcaffeoyltartaric acid | C23H20O12 | [M-H]- | Phenolic acids |
| 663 | Quercetagetin-7-O-glucoside(Quercetagitrin)* | C21H20O13 | [M+H]+ | Flavonoids | 1632 | Protocatechuic acid 4-O-(2''-O-Vanilloyl)Glucoside | C21H22O12 | [M-H]- | Phenolic acids |
| 664 | 6-C-Methylquercetin-3-O-rhamnoside | C22H22O11 | [M+H]+ | Flavonoids | 1633 | Maplexin D (2,4-Di-O-Galloyl-1,5-Anhydro-D-Glucitol)* | C20H20O13 | [M-H]- | Phenolic acids |
| 665 | Quercetagetin; 3,3',4',5,6,7-Hexahydroxyflavone | C15H10O8 | [M+H]+ | Flavonoids | 1634 | 6-O-Galloyl-1-O-vanilloyl-β-D-glucose | C21H22O13 | [M-H]- | Phenolic acids |
| 666 | 6-Hydroxykaempferol-3,6-O-Diglucoside* | C27H30O17 | [M+H]+ | Flavonoids | 1635 | 3,4,5-Trimethoxyphenol-1-O-β-D-glucoside | C15H22O9 | [M+H]+ | Phenolic acids |
| 667 | Hesperetin-6-C-glucoside-7-O-glucoside | C28H34O16 | [M+H]+ | Flavonoids | 1636 | 3-Methylsalicylic Acid | C8H8O3 | [M-H]- | Phenolic acids |
| 668 | Quercetin-3-O-(2''-O-galloyl)galactoside | C28H24O16 | [M+H]+ | Flavonoids | 1637 | 4-Hydroxyphenyllactic Acid* | C9H10O4 | [M-H]- | Phenolic acids |
| 669 | Morin | C15H10O7 | [M-H]- | Flavonoids | 1638 | Methyl 2,4-dihydroxyphenylacetate* | C9H10O4 | [M-H]- | Phenolic acids |
| 670 | 3,5,4'-Trihydroxy-7-methoxyflavone (Rhamnocitrin)* | C16H12O6 | [M+H]+ | Flavonoids | 1639 | Sodium ferulate | C10H9NaO4 | [M-Na]- | Phenolic acids |
| 671 | Quercetin-7-O-(2''-malonyl)glucosyl-5-O-glucoside | C30H32O20 | [M+H]+ | Flavonoids | 1640 | 4-O-(6'-O-Glucosylcaffeoyl)-3,4-dihydroxybenzyl alcohol | C22H24O11 | [M-H]- | Phenolic acids |
| 672 | Kaempferol-3-O-glucuronide | C21H18O12 | [M-H]- | Flavonoids | 1641 | 3-(3,4,5-Trimethoxyphenyl)propan-1-ol | C12H18O4 | [M+H]+ | Phenolic acids |
| 673 | Kaempferol-3-O-(6''-p-Coumaroyl)glucoside (Tiliroside) | C30H26O13 | [M-H]- | Flavonoids | 1642 | Salicin | C13H18O7 | [M-H]- | Phenolic acids |
| 674 | Kaempferol-3-O-robinoside-7-O-rhamnoside (Robinin)* | C33H40O19 | [M+H]+ | Flavonoids | 1643 | Calceolarioside B* | C23H26O11 | [M+H]+ | Phenolic acids |
| 675 | Isorhamnetin-3,7-O-diglucoside | C28H32O17 | [M+H]+ | Flavonoids | 1644 | 3-O-Feruloylquinic acid | C17H20O9 | [M+H]+ | Phenolic acids |
| 676 | Ikarisoside D | C28H30O11 | [M+H]+ | Flavonoids | 1645 | P-Methoyxcinnamate glucoside | C16H20O8 | [M-H]- | Phenolic acids |
| 677 | Tamarixetin (3,3',5,7-Tetrahydroxy-4'-Methoxyflavone) | C16H12O7 | [M+H]+ | Flavonoids | 1646 | Protosappanin B | C16H16O6 | [M-H]- | Phenolic acids |
| 678 | 6-Hydroxykaempferol-7,6-O-Diglucoside | C27H30O17 | [M+H]+ | Flavonoids | 1647 | 3-(4-hydroxyphenyl)-3-oxopropyl beta-D-glucopyranoside* | C15H20O8 | [M+H]+ | Phenolic acids |
| 679 | Kaempferol-3-O-(2''-p-Coumaroyl)glucoside | C30H26O13 | [M-H]- | Flavonoids | 1648 | Phenyl acetate* | C8H8O2 | [M-H]- | Phenolic acids |
| 680 | Syringetin-7-O-glucoside* | C23H24O13 | [M+H]+ | Flavonoids | 1649 | 4-Methylphenol | C7H8O | [M-H]- | Phenolic acids |
| 681 | Quercetin-3-O-(2''-O-galloyl)Arabinoside | C27H22O15 | [M+H]+ | Flavonoids | 1650 | (E)-3-[(2S,3S)-2-(4-hydroxy-3-methoxyphenyl)-7-methoxy-3-methyl-2,3-dihydro-1-benzofuran-5-yl]prop-2-enal* | C20H20O5 | [M-H]- | Phenolic acids |
| 682 | Quercetin-3,7-Di-O-glucoside* | C27H30O17 | [M+H]+ | Flavonoids | 1651 | 4-O-(6'-O-Glucosylferuloyl)-3,4-dihydroxybenzyl alcohol | C23H26O11 | [M-H]- | Phenolic acids |
| 683 | Kaempferol-3-O-(6''-Malonyl)glucoside-7-O-Glucoside | C30H32O19 | [M+H]+ | Flavonoids | 1652 | 2-(3-β-D-glucopyranosyloxy-4-hydroxyphenyl)ethanol-1-O-β-D-glucopyranoside* | C20H30O13 | [M-H]- | Phenolic acids |
| 684 | Kaempferol-3-O-(6''-Rhamnosyl-2''-Glucosyl)Glucoside (Camelliaside A) | C33H40O20 | [M+H]+ | Flavonoids | 1653 | Benzoylmalic acid | C11H10O6 | [M-H]- | Phenolic acids |
| 685 | Limocitrin-3-O-galactoside* | C23H24O13 | [M+H]+ | Flavonoids | 1654 | 4-O-(6'-O-Glucosylcaffeoyl)-3,4-dihydroxybenzoic acid | C22H22O12 | [M-H]- | Phenolic acids |
| 686 | Kaempferol-3-O-(6''''-malonyl)sophorotrioside | C36H42O24 | [M+H]+ | Flavonoids | 1655 | Digallic Acid | C14H10O9 | [M-H]- | Phenolic acids |
| 687 | 3-Methylkaempferol | C16H12O6 | [M+H]+ | Flavonoids | 1656 | 4-Aminobenzoic acid | C7H7NO2 | [M+H]+ | Phenolic acids |
| 688 | Quercetin-5,4'-di-O-glucoside | C27H30O17 | [M+H]+ | Flavonoids | 1657 | 1-O-Caffeoylglycerol | C12H14O6 | [M+H]+ | Phenolic acids |
| 689 | Myricetin-3-O-galactoside-3'-O-rhamnoside | C27H30O17 | [M+H]+ | Flavonoids | 1658 | Trans-5-O-(p-Coumaroyl)shikimate | C16H16O7 | [M+H]+ | Phenolic acids |
| 690 | Kaempferol-3-O-(2''-apiosyl-4''-glucosyl-6''-malonyl)Glucoside | C35H40O23 | [M+H]+ | Flavonoids | 1659 | 2-Phenylethy-1-O-β-D-glucoside | C14H20O6 | [M+H]+ | Phenolic acids |
| 691 | Complanatuside | C28H32O16 | [M+H]+ | Flavonoids | 1660 | 5-Methoxysalicylic acid | C8H8O4 | [M+H]+ | Phenolic acids |
| 692 | Quercetin-3-O-(6''-O-galloyl)glucoside | C28H24O16 | [M+H]+ | Flavonoids | 1661 | 1-(3,4-dihydroxyphenyl)-7-(4-hydroxy-3-methoxyphenyl)hepta-1,6-diene-3,5-dione | C20H18O6 | [M+H]+ | Phenolic acids |
| 693 | Rhamnetin-3-O-rhamnoside | C22H22O11 | [M-H]- | Flavonoids | 1662 | Tyrosol; 4-Hydroxyphenylethanol | C8H10O2 | [M-H]- | Phenolic acids |
| 694 | Quercetin-3-O-sophoroside (Baimaside)* | C27H30O17 | [M+H]+ | Flavonoids | 1663 | 3,4,5-trihydroxy-6-(4-octylphenoxy)oxane-2-carboxylic acid | C20H30O7 | [M+H]+ | Phenolic acids |
| 695 | kaempferol-3-methoxycaffeoyldiglucoside | C37H38O20 | [M+H]+ | Flavonoids | 1664 | Eugenol | C10H12O2 | [M+H]+ | Phenolic acids |
| 696 | Isorhamnetin-3-O-glucuronide-7-O-rhamnoside | C28H30O17 | [M+H]+ | Flavonoids | 1665 | 4-Hydroxybenzoylmalic acid | C11H10O7 | [M-H]- | Phenolic acids |
| 697 | Patuletin-3-O-rutinoside | C28H32O17 | [M+H]+ | Flavonoids | 1666 | Gallic acid-4-O-(6'''-feruloyl)sophoroside | C29H34O18 | [M+H]+ | Phenolic acids |
| 698 | Limocitrin-3-O-glucoside* | C23H24O13 | [M+H]+ | Flavonoids | 1667 | Coniferyl alcohol* | C10H12O3 | [M-H]- | Phenolic acids |
| 699 | Kaempferol-3-O-(3''-O-p-Coumaroyl)rhamnoside | C30H26O12 | [M+H]+ | Flavonoids | 1668 | Gastrodioside | C20H24O8 | [M-H]- | Phenolic acids |
| 700 | Phellatin | C26H30O12 | [M-H]- | Flavonoids | 1669 | 3-O-Galloyl-Glucose | C13H16O10 | [M-H]- | Phenolic acids |
| 701 | Quercetin-3-O-α-rhamnosyl (1→2)-[α-rhamnosyl (1→6)]-β-glucoside | C33H40O20 | [M+H]+ | Flavonoids | 1670 | Gallic acid | C7H6O5 | [M+H]+ | Phenolic acids |
| 702 | kaempferol-3-sinapoyldiglucoside | C38H40O20 | [M+H]+ | Flavonoids | 1671 | Furanofructosyl-α-D-(6-mustard acyl)glucoside* | C23H32O15 | [M-H]- | Phenolic acids |
| 703 | Kaempferol-3-O-(2''-p-Coumaroyl)galactoside* | C30H26O13 | [M+H]+ | Flavonoids | 1672 | Isochlorogenic acid B* | C25H24O12 | [M-H]- | Phenolic acids |
| 704 | Myricetin-3-O-arabinoside | C20H18O12 | [M-H]- | Flavonoids | 1673 | Dihydrocaffeic acid | C9H10O4 | [M+H]+ | Phenolic acids |
| 705 | Kaempferol-3-O-galactoside-4'-O-glucoside* | C27H30O16 | [M+H]+ | Flavonoids | 1674 | Vanillic acid | C8H8O4 | [M-H]- | Phenolic acids |
| 706 | Isorhamnetin-3-O-(6''-acetylglucosyl)(1→3)-glucoside | C30H34O18 | [M+H]+ | Flavonoids | 1675 | Iriflophenone-3-C-glucoside | C19H20O10 | [M-H]- | Phenolic acids |
| 707 | Quercetin-3-O-xylosyl(1→2)glucosyl(1→2)glucoside | C32H38O21 | [M+H]+ | Flavonoids | 1676 | 3-hydroxyphenylacetic acid | C8H8O3 | [M-H]- | Phenolic acids |
| 708 | Quercetin-3-O-sambubioside | C26H28O16 | [M+H]+ | Flavonoids | 1677 | Methyl 3-O-Methyl Gallate | C9H10O5 | [M-H]- | Phenolic acids |
| 709 | Myricetin-3-O-rhamnoside (Myricitrin) | C21H20O12 | [M-H]- | Flavonoids | 1678 | Lavandulifolioside | C34H44O19 | [M-H]- | Phenolic acids |
| 710 | Kaempferide-3-O-（6'-O-acetyl）glucoside* | C24H24O12 | [M-H]- | Flavonoids | 1679 | 1-(2,4,5-Trimethoxyphenyl)propan-1-one | C12H16O4 | [M+H]+ | Phenolic acids |
| 711 | Quercetin-4'-O-glucuronide | C21H18O13 | [M-H]- | Flavonoids | 1680 | 5-O-p-Coumaroylshikimic acid O-glucoside | C22H26O12 | [M-H]- | Phenolic acids |
| 712 | Quercetin-3-O-(2''-O-malonyl)sophoroside-7-O-arabinoside | C35H40O24 | [M+H]+ | Flavonoids | 1681 | 2-Naphthol* | C10H8O | [M-H]- | Phenolic acids |
| 713 | kaempferol-3-sinapoyldiglucoside-7-glucoside | C44H50O25 | [M+H]+ | Flavonoids | 1682 | Feruloylferuloyltartaric acid | C24H22O12 | [M-H]- | Phenolic acids |
| 714 | 6-Methoxyquercetin-3-O-rhamnoside | C22H22O12 | [M+H]+ | Flavonoids | 1683 | Xanthoxylin | C10H12O4 | [M+H]+ | Phenolic acids |
| 715 | Isorhamnetin-3-O-rutinoside-7-O-(2''-O-glucosyl)glucuronate | C40H50O27 | [M+H]+ | Flavonoids | 1684 | 3,4-dihydroxybenzaldehyde-xylose-glucoside* | C18H24O12 | [M-H]- | Phenolic acids |
| 716 | Kaempferol-3-O-(6''-Feruloyl)glucosyl-(1→4)-galactoside | C37H38O19 | [M+H]+ | Flavonoids | 1685 | 3,4,5-Tricaffeoylquinic acid | C34H30O15 | [M-H]- | Phenolic acids |
| 717 | 3,7-Di-O-methylquercetin | C17H14O7 | [M-H]- | Flavonoids | 1686 | Desrhamnosylisoacteoside* | C23H26O11 | [M+H]+ | Phenolic acids |
| 718 | Quercetin-3-O-xylosyl(1→2)arabinoside | C25H26O15 | [M+H]+ | Flavonoids | 1687 | 2-hydroxymethyl benzoic acid | C8H8O3 | [M-H]- | Phenolic acids |
| 719 | Quercetin-3-O-Sambubioside-5-O-Glucoside | C32H38O21 | [M+H]+ | Flavonoids | 1688 | Caffeoylbenzoyltartaric acid | C20H16O10 | [M-H]- | Phenolic acids |
| 720 | Kaempferol (3,5,7,4'-Tetrahydroxyflavone) | C15H10O6 | [M+H]+ | Flavonoids | 1689 | 4-Hydroxybenzoic acid glucosyl xyloside* | C18H24O12 | [M-H]- | Phenolic acids |
| 721 | Quercetin-5-O-glucuronide | C21H18O13 | [M+H]+ | Flavonoids | 1690 | 3,5-Digalloylshikimic acid | C23H26O11 | [M-H]- | Phenolic acids |
| 722 | Quercetin-3-O-(2''-O-rhamnosyl)rutinoside-7-O-glucoside | C39H50O25 | [M+H]+ | Flavonoids | 1691 | 1-O-Galloyl-6-O-p-Coumaroyl-β-D-glucose* | C22H22O12 | [M-H]- | Phenolic acids |
| 723 | Quercetin-3,3'-dimethyl ether | C17H14O7 | [M-H]- | Flavonoids | 1692 | Osmanthuside H[2-(4-Hydroxyphenyl)ethyl-β-D-apiosyl-(1→6)-β-D-glucoside] | C19H28O11 | [M-H]- | Phenolic acids |
| 724 | Myricetin-3-O-(6''-malony)glucoside | C24H22O16 | [M+H]+ | Flavonoids | 1693 | Calceolarioside A* | C23H26O11 | [M+H]+ | Phenolic acids |
| 725 | Kaempferol-3-O-(2'''-p-Coumaroyl)sophoroside-7-O-Glucoside | C42H46O23 | [M+H]+ | Flavonoids | 1694 | 1-Naphthol* | C10H8O | [M-H]- | Phenolic acids |
| 726 | Myricetin-3-O-(6''-acetyl)glucoside | C23H22O14 | [M+H]+ | Flavonoids | 1695 | Maleoyl-caffeoylquinic acid | C20H20O12 | [M-H]- | Phenolic acids |
| 727 | 6-Methoxyquercetin-3-O-Xyloside | C21H20O12 | [M+H]+ | Flavonoids | 1696 | 2-O-Caffeoylmalic acid | C13H12O8 | [M-H]- | Phenolic acids |
| 728 | Limocitrin-3-O-arabinoside | C22H22O12 | [M+H]+ | Flavonoids | 1697 | Yunnaneic acid D | C27H24O12 | [M-H]- | Phenolic acids |
| 729 | Kaempferol-6,8-di-C-glucoside* | C27H30O16 | [M+H]+ | Flavonoids | 1698 | Sibiricose A6 | C23H32O15 | [M-H]- | Phenolic acids |
| 730 | Isorhamnetin-3-O-(6''-malonyl)glucoside-7-O-rhamnoside | C31H34O19 | [M+H]+ | Flavonoids | 1699 | 2-Acetyl-3-hydroxyphenyl-1-O-glucoside | C15H20O7 | [M-H]- | Phenolic acids |
| 731 | Quercetin-3-O-sophorotrioside-7-O-arabinoside | C38H48O26 | [M+H]+ | Flavonoids | 1700 | 4-Hydroxyphenethoxy-8-O-β-D-[6-O-(4-O-β-D-glucopyranosyl)-sinapoyl]glucopyranoside | C31H40O16 | [M-H]- | Phenolic acids |
| 732 | Quercetin-3-O-(2''-O-glucosyl)glucuronide | C27H28O18 | [M+H]+ | Flavonoids | 1701 | 3-Hydroxy-5-Methylphenol-1-O-(6'-Digalloyl)Glucoside | C27H26O15 | [M-H]- | Phenolic acids |
| 733 | Patuletin-3-O-glucoside* | C22H22O13 | [M+H]+ | Flavonoids | 1702 | 1-O-Galloyl-3-O-p-Coumaroyl-β-D-glucose* | C22H22O12 | [M-H]- | Phenolic acids |
| 734 | Kaempferol-3-O-(6''-p-Coumaroyl)galactoside* | C30H26O13 | [M+H]+ | Flavonoids | 1703 | 4-Methylcatechol | C7H8O2 | [M-H]- | Phenolic acids |
| 735 | Kaempferol-3-O-arabinoside-7-O-rhamnoside | C26H28O14 | [M+H]+ | Flavonoids | 1704 | Vanillin acetate | C10H10O4 | [M+H]+ | Phenolic acids |
| 736 | Kaempferol-3-O-(6''-Sinapyl)glucosyl-(1→2)-Galactoside | C38H40O20 | [M+H]+ | Flavonoids | 1705 | 6'-O-Sinapoylsucrose* | C23H32O15 | [M-H]- | Phenolic acids |
| 737 | Prunetin-4'-O-glucoside(Prunitrin) | C22H22O10 | [M-H]- | Flavonoids | 1706 | Cimicifugic acid C | C20H18O10 | [M-H]- | Phenolic acids |
| 738 | Genistein-7-O-galactoside* | C21H20O10 | [M+H]+ | Flavonoids | 1707 | 2,6-Dimethoxybenzoic acid | C9H10O4 | [M+H]+ | Phenolic acids |
| 739 | Sophoricoside* | C21H20O10 | [M+H]+ | Flavonoids | 1708 | 3,4-di-hydroxyphenethyol alcohol 4-O-β-D-(6'-O-galloyol)-glucopyranoside | C21H24O12 | [M-H]- | Phenolic acids |
| 740 | Genistein-7-O-Glucoside (Genistin) | C21H20O10 | [M-H]- | Flavonoids | 1709 | p-Coumaroylcaffeoyltartaric acid | C22H18O11 | [M-H]- | Phenolic acids |
| 741 | 1,2,4,5-tetrahydroxy-7-(hydroxymethyl)anthracene-9,10-dione | C15H10O7 | [M+H]+ | Flavonoids | 1710 | 3-(4-Hydroxyphenyl)-1-propanol | C9H12O2 | [M-H]- | Phenolic acids |
| 742 | 1,2,3,7,8-pentahydroxy-6-methylanthracene-9,10-dione | C15H10O7 | [M+H]+ | Flavonoids | 1711 | D-Threo-guaiacylglycerol-7-O-β-D-glucoside | C16H24O10 | [M-H]- | Phenolic acids |
| 743 | tenuiflorin C glucoside* | C22H22O12 | [M+H]+ | Flavonoids | 1712 | 1,4-Di-O-Galloyl-D-glucose | C20H20O14 | [M-H]- | Phenolic acids |
| 744 | 1,8-dihydroxy-4,5-dimethoxy-3-{[(2s,3r,4s,5s,6r)-3,4,5-trihydroxy-6-(hydroxymethyl)oxan-2-yl]oxy}xanthen-9-one* | C21H22O12 | [M-H]- | Flavonoids | 1713 | 2',6'-Dihydroxyacetophenone | C8H8O3 | [M+H]+ | Phenolic acids |
| 745 | Lancerin; 4-C-Glucosyl-1,3,7-Trihydroxyxanthone | C19H18O10 | [M-H]- | Flavonoids | 1714 | 2-Hydroxyphenol-1-O-glucosyl(6→1)rhamnoside | C18H26O11 | [M-H]- | Phenolic acids |
| 746 | 2,5-dihydroxy-1,6-dimethoxy-8-{[(2s,3r,4s,5s,6r)-3,4,5-trihydroxy-6-(hydroxymethyl)oxan-2-yl]oxy}xanthen-9-one | C21H22O12 | [M-H]- | Flavonoids | 1715 | Syringalide A | C23H26O10 | [M-H]- | Phenolic acids |
| 747 | 1,3,6,7-tetrahydroxy-2-(3,4,5-trihydroxyoxan-2-yl)xanthen-9-one | C18H16O10 | [M-H]- | Flavonoids | 1716 | Isosalicin | C13H18O7 | [M-H]- | Phenolic acids |
| 748 | 1,8-dihydroxy-2,6-dimethoxy-5-{[(2s,3r,4s,5s,6r)-3,4,5-trihydroxy-6-(hydroxymethyl)oxan-2-yl]oxy}xanthen-9-one | C21H22O12 | [M-H]- | Flavonoids | 1717 | 3,4'-Dihydroxy-3'-methoxybenzenepentanoic acid | C12H16O5 | [M-H]- | Phenolic acids |
| 749 | 2,3,5-trimethoxy-1-{[(2s,3r,4s,5s,6r)-3,4,5-trihydroxy-6-(hydroxymethyl)oxan-2-yl]oxy}xanthen-9-one* | C22H24O11 | [M+H]+ | Flavonoids | 1718 | 1-Feruloyl-sn-glycerol* | C13H16O6 | [M+H]+ | Phenolic acids |
| 750 | 5,7,3',4',5'-Pentahydroxyflavan-7-gallate | C22H18O10 | [M+H]+ | Flavonoids | 1719 | 3-O-p-Coumaroylshikimic acid | C16H16O7 | [M-H]- | Phenolic acids |
| 751 | Homomangiferin | C20H20O11 | [M-H]- | Flavonoids | 1720 | Monogalloyl-diglucose | C19H26O15 | [M-H]- | Phenolic acids |
| 752 | 1,6-dihydroxy-3,7-dimethoxy-8-(3-methoxy-3-methylbutyl)-2-(3-methylbut-2-en-1-yl)xanthen-9-one | C26H32O7 | [M-H]- | Flavonoids | 1721 | 3'-Gallic acyl sucrose | C19H26O14 | [M-H]- | Phenolic acids |
| 753 | 2,6-Dimethoxypydroquinone-1-O-glucoside | C14H20O9 | [M+H]+ | Flavonoids | 1722 | Trigallic acid | C21H14O13 | [M-H]- | Phenolic acids |
| 754 | Isoscopoletin-β-D-glucoside* | C16H18O9 | [M+H]+ | Lignans and Coumarins | 1723 | 1-O-Galloyl-2-O-p-Coumaroyl-β-D-glucose* | C22H22O12 | [M-H]- | Phenolic acids |
| 755 | 8-Hydroxycoumarin | C9H6O3 | [M+H]+ | Lignans and Coumarins | 1724 | 6-(5-Carboxy-2,3-Dihydroxyphenoxy)-3,4,5-Trihydroxyoxane-2-Carboxylic Acid | C13H14O11 | [M-H]- | Phenolic acids |
| 756 | Esculetin (6,7-Dihydroxycoumarin) | C9H6O4 | [M-H]- | Lignans and Coumarins | 1725 | 1-O-Caffeoyl-4-O-galloyl-β-D-glucose | C22H22O13 | [M-H]- | Phenolic acids |
| 757 | Fraxetin-8-O-glucoside (Fraxin) | C16H18O10 | [M-H]- | Lignans and Coumarins | 1726 | 4-O-(6'-O-Glucosyl-4''-hydroxybenzoyl)-4-hydroxybenzyl alcohol | C20H22O9 | [M-H]- | Phenolic acids |
| 758 | Daphnin* | C15H16O9 | [M-H]- | Lignans and Coumarins | 1727 | 3-[(1-Carboxyvinyl)oxy]benzoic acid | C10H8O5 | [M-H]- | Phenolic acids |
| 759 | Cichoriin* | C15H16O9 | [M-H]- | Lignans and Coumarins | 1728 | Verbasoside | C20H30O12 | [M-H]- | Phenolic acids |
| 760 | Esculetin-7-O-glucoside* | C15H16O9 | [M-H]- | Lignans and Coumarins | 1729 | 3-(4-Hydroxyphenyl)-propionic acid* | C9H10O3 | [M-H]- | Phenolic acids |
| 761 | Isofraxidin-7-O-glucoside | C17H20O10 | [M+H]+ | Lignans and Coumarins | 1730 | 3,4-Digalloylshikimic acid | C21H18O13 | [M-H]- | Phenolic acids |
| 762 | 7-hydroxy-2H-1-benzopyran-2-one* | C9H6O3 | [M+H]+ | Lignans and Coumarins | 1731 | 1,3-O-Dicaffeoylquinic Acid (Cynarin) | C25H24O12 | [M+H]+ | Phenolic acids |
| 763 | 6-Hydroxycoumarin* | C9H6O3 | [M+H]+ | Lignans and Coumarins | 1732 | 3-O-Cinnamoyl-4,6-(S)-HHDP-β-D-glucose* | C29H24O15 | [M+H]+ | Phenolic acids |
| 764 | Xanthotoxol; 8-Hydroxypsoralen | C11H6O4 | [M+H]+ | Lignans and Coumarins | 1733 | 2-Caffeoyl-L-tartaric acid (Caftaric acid) | C13H12O9 | [M-H]- | Phenolic acids |
| 765 | Fraxetin-7,8-di-O-glucoside | C22H28O15 | [M-H]- | Lignans and Coumarins | 1734 | methyl-4,5-diacetyloxy-3-hydroxy-6-[4-(hydroxymethyl)phenoxy]oxane-2-carboxylate | C18H22O10 | [M+H]+ | Phenolic acids |
| 766 | Scopoletin (7-Hydroxy-6-methoxycoumarin) | C10H8O4 | [M+H]+ | Lignans and Coumarins | 1735 | 3,4-dihydroxyallyl benzene-4-O-[α-L-rhamnosyl-(1→6)]-β-D-glucopyranoside | C21H30O11 | [M-H]- | Phenolic acids |
| 767 | Scopoletin-7-O-glucoside (Scopolin) | C16H18O9 | [M+H]+ | Lignans and Coumarins | 1736 | 2,3,4,5-Trihydroxybutyl 6-O-(E)-caffeoyl-β-D-glucopyranoside | C21H30O13 | [M-H]- | Phenolic acids |
| 768 | 5,7-Dihydroxy-4-methylcoumarin | C10H8O4 | [M+H]+ | Lignans and Coumarins | 1737 | 3,5-Dihydroxyphenyl1-O-(6-O-Galloyl-β-D-Glucopyranoside) | C19H20O12 | [M+H]+ | Phenolic acids |
| 769 | Ditartaroyl-hydroxycoumarin | C17H14O13 | [M+H]+ | Lignans and Coumarins | 1738 | Syringic acid | C9H10O5 | [M-H]- | Phenolic acids |
| 770 | 3-Methyl-4,8-dihydroxy-3,4-dihydroisocoumarin | C10H10O4 | [M-H]- | Lignans and Coumarins | 1739 | 1-O-Caffeoyl-3-O-galloyl-β-D-glucose | C22H22O13 | [M-H]- | Phenolic acids |
| 771 | 7-C-Glucosylcoumarin* | C15H16O7 | [M+H]+ | Lignans and Coumarins | 1740 | Antiarol; 3,4,5-Trimethoxyphenol | C9H12O4 | [M+H]+ | Phenolic acids |
| 772 | Daphnetin | C9H6O4 | [M-H]- | Lignans and Coumarins | 1741 | Bis(p-Coumaroyl)tartaric acid | C22H18O10 | [M-H]- | Phenolic acids |
| 773 | 6,7-Dihydroxycoumarin-7-O-(6'-acetyl)glucoside | C16H16O10 | [M-H]- | Lignans and Coumarins | 1742 | Propyl 4-hydroxybenzoate | C10H12O3 | [M-H]- | Phenolic acids |
| 774 | 3,4-Dihydro-4-(4'-hydroxyphenyl)-5,7-dihydroxycoumarin glucoside* | C21H22O10 | [M+H]+ | Lignans and Coumarins | 1743 | 1-O-Galloyl-4-O-p-Coumaroyl-β-D-glucose* | C22H22O12 | [M-H]- | Phenolic acids |
| 775 | Fraxidin-8-O-glucoside | C17H20O10 | [M+H]+ | Lignans and Coumarins | 1744 | 3-O-Methylgallic acid | C8H8O5 | [M-H]- | Phenolic acids |
| 776 | Angelicin* | C11H6O3 | [M+H]+ | Lignans and Coumarins | 1745 | Homovanillic alcohol; 4-Hydroxy-3-methoxyphenethanol | C9H12O3 | [M-H]- | Phenolic acids |
| 777 | Coumarin-3-carboxylic Acid | C10H6O4 | [M+H]+ | Lignans and Coumarins | 1746 | Isoacteoside | C29H36O15 | [M+H]+ | Phenolic acids |
| 778 | Psoralen* | C11H6O3 | [M+H]+ | Lignans and Coumarins | 1747 | 1,6-Di-O-Galloyl-D-Glucose* | C20H20O14 | [M-H]- | Phenolic acids |
| 779 | 6,7-Dihydroxy-4-methylcoumarin | C10H8O4 | [M+H]+ | Lignans and Coumarins | 1748 | Arillatose A | C34H40O19 | [M-H]- | Phenolic acids |
| 780 | Scopoletin-7-O-xylosyl(1→6)glucoside | C21H26O13 | [M+H]+ | Lignans and Coumarins | 1749 | o-Anisic acid | C8H8O3 | [M+H]+ | Phenolic acids |
| 781 | Suberosin | C15H16O3 | [M+H]+ | Lignans and Coumarins | 1750 | 1-O-Cinnamoyl-4,6-(S)-HHDP-β-D-glucose* | C29H24O15 | [M+H]+ | Phenolic acids |
| 782 | Sideretin (5,7,8-Trihydroxy-6-methoxycoumarin) | C10H8O6 | [M-H]- | Lignans and Coumarins | 1751 | Vanillyl alcohol | C8H10O3 | [M-H]- | Phenolic acids |
| 783 | 6,8-Dihydroxy-3-methylisocoumarin | C10H8O4 | [M-H]- | Lignans and Coumarins | 1752 | 1-O-Galloyl-3-O-Feruloyl-β-D-glucose* | C23H24O13 | [M-H]- | Phenolic acids |
| 784 | Fraxidin (8-Hydroxy-6,7-dimethoxycoumarin) | C11H10O5 | [M+H]+ | Lignans and Coumarins | 1753 | 1-O-Galloyl-2-O-Feruloyl-β-D-glucose* | C23H24O13 | [M-H]- | Phenolic acids |
| 785 | Meranzin | C15H16O4 | [M+H]+ | Lignans and Coumarins | 1754 | Furanofructosyl-α-D-(3-mustard acyl)glucoside* | C23H32O15 | [M-H]- | Phenolic acids |
| 786 | (S)-Peucedanol | C14H16O5 | [M+H]+ | Lignans and Coumarins | 1755 | methyl 4-O-galloylchlorogenate | C24H24O13 | [M-H]- | Phenolic acids |
| 787 | Edgeworin rhamnoside | C24H20O10 | [M+H]+ | Lignans and Coumarins | 1756 | 6-O-Glucosyl-feruloylbenzoic acid | C23H24O10 | [M-H]- | Phenolic acids |
| 788 | Coumarin | C9H6O2 | [M+H]+ | Lignans and Coumarins | 1757 | 2'-Acetylacteoside | C31H38O16 | [M-H]- | Phenolic acids |
| 789 | 4-Hydroxycoumarin di-glucoside | C21H26O13 | [M-H]- | Lignans and Coumarins | 1758 | Methyl Brevifolincarboxylate | C14H10O8 | [M-H]- | Phenolic acids |
| 790 | Epoxybergamottin | C21H22O5 | [M+H]+ | Lignans and Coumarins | 1759 | Methyl gallate | C8H8O5 | [M-H]- | Phenolic acids |
| 791 | 7-Hydroxy-4-methylcoumarin | C10H8O3 | [M-H]- | Lignans and Coumarins | 1760 | 4-p-Cumaroyl-rhamnosyl-(1→6)-D-glucose | C21H28O12 | [M-H]- | Phenolic acids |
| 792 | 7-Methoxy-5-Prenyloxycoumarin | C15H16O4 | [M+H]+ | Lignans and Coumarins | 1761 | 2,3-di-O-galloyl--d-glucose* | C20H20O14 | [M-H]- | Phenolic acids |
| 793 | Fraxetin (7,8-Dihydroxy-6-methoxycoumarin) | C10H8O5 | [M+H]+ | Lignans and Coumarins | 1762 | Sinapoylcaffeoyltartaric acid | C24H22O13 | [M-H]- | Phenolic acids |
| 794 | Secoisolariciresinol | C20H26O6 | [M-H]- | Lignans and Coumarins | 1763 | 1-O-p-Cumaroylglycerol | C12H14O5 | [M-H]- | Phenolic acids |
| 795 | Dehydrodiconiferyl alcohol | C20H22O6 | [M+H]+ | Lignans and Coumarins | 1764 | 1-O-Galloyl-6-O-Cinnamoyl-β-D-glucose* | C22H22O11 | [M-H]- | Phenolic acids |
| 796 | Secoisolariciresinol-9'-O-xyloside | C25H34O10 | [M-H]- | Lignans and Coumarins | 1765 | 1-O-Galloyl-6-O-Feruloyl-β-D-glucose* | C23H24O13 | [M-H]- | Phenolic acids |
| 797 | Schisandrin; Schizandrin; Schizandrol A | C24H32O7 | [M+H]+ | Lignans and Coumarins | 1766 | Crenatoside | C29H34O15 | [M+H]+ | Phenolic acids |
| 798 | 1-Hydroxypinoresinol-1-O-Glucoside* | C26H32O12 | [M-H]- | Lignans and Coumarins | 1767 | Methyl 2-O-(4-hydroxybenzoyl)-2,4,6-trihydroxyphenylacetate | C16H14O7 | [M-H]- | Phenolic acids |
| 799 | 6-((4-(3-hydroxy-2-(4-(3-hydroxypropyl)-2-methylphenoxy)propoxy)-2-methoxyphenoxy)methyl)tetrahydro-2H-pyran-2,3,4,5-tetraol | C26H36O11 | [M-H]- | Lignans and Coumarins | 1768 | p-Coumaraldehyde | C9H8O2 | [M-H]- | Phenolic acids |
| 800 | Schizandriside* | C25H32O10 | [M-H]- | Lignans and Coumarins | 1769 | Acteoside; Verbascoside | C29H36O15 | [M-H]- | Phenolic acids |
| 801 | Isolariciresinol-9'-O-xyloside* | C25H32O10 | [M-H]- | Lignans and Coumarins | 1770 | Chicoric Acid | C22H18O12 | [M-H]- | Phenolic acids |
| 802 | (2r,3r,4s)-6-hydroxy-4-(4-hydroxy-3-methoxyphenyl)-7-methoxy-2,3-dimethyl-3,4-dihydro-2h-naphthalen-1-one | C20H22O5 | [M-H]- | Lignans and Coumarins | 1771 | 3-Hydroxy-5-Methylphenol-1-O-(6'-Galloyl)Glucoside | C20H22O11 | [M-H]- | Phenolic acids |
| 803 | epieudesmin | C22H26O6 | [M+H]+ | Lignans and Coumarins | 1772 | 1-O-Galloyl-4-O-Feruloyl-β-D-glucose* | C23H24O13 | [M-H]- | Phenolic acids |
| 804 | Nortrachelogenin-4-O-glucoside* | C26H32O12 | [M-H]- | Lignans and Coumarins | 1773 | 3,4-Dimethoxyphenyl acetic acid | C10H12O4 | [M-H]- | Phenolic acids |
| 805 | Lyoniresinol-9'-O-xyloside (Lyoniside) | C27H36O12 | [M-H]- | Lignans and Coumarins | 1774 | Salvianolic acid B* | C36H30O16 | [M-H]- | Phenolic acids |
| 806 | Dihydrodehydrodiconiferyl alcohol-4-O-glucoside* | C26H34O11 | [M-H]- | Lignans and Coumarins | 1775 | 4-Methoxyphenylpropionic acid* | C10H12O3 | [M-H]- | Phenolic acids |
| 807 | Pinoresinol-4-O-glucoside* | C26H32O11 | [M-H]- | Lignans and Coumarins | 1776 | Cistanoside A | C36H48O20 | [M-H]- | Phenolic acids |
| 808 | Lyoniresinol | C22H28O8 | [M-H]- | Lignans and Coumarins | 1777 | 3-Methylcatechol | C7H8O2 | [M-H]- | Phenolic acids |
| 809 | Dihydrodehydrodiconiferyl alcohol | C20H24O6 | [M+H]+ | Lignans and Coumarins | 1778 | 4-O-Galloyl-1-O-Cinnamoyl-β-D-glucose* | C22H22O11 | [M-H]- | Phenolic acids |
| 810 | Lariciresinol-4'-O-glucoside* | C26H34O11 | [M-H]- | Lignans and Coumarins | 1779 | 2-O-Trigalloyl-glucose-glucose | C33H34O23 | [M-H]- | Phenolic acids |
| 811 | (7'R,8'R)-7'8'-dihydro-7'-(5'-hydroxy-3'-methoxphenyl)-3-methoxy-8'-methyl-1-(E)-propenylbenzof-uran | C20H22O4 | [M+H]+ | Lignans and Coumarins | 1780 | p-Dimeric galloyl methyl ester | C15H12O9 | [M-H]- | Phenolic acids |
| 812 | Machilin H | C21H26O6 | [M+H]+ | Lignans and Coumarins | 1781 | Digalloylglucose | C20H20O14 | [M-H]- | Phenolic acids |
| 813 | Isolariciresinol-9'-O-glucoside* | C26H34O11 | [M-H]- | Lignans and Coumarins | 1782 | 6'-O-Galloylsalicin | C20H22O11 | [M-H]- | Phenolic acids |
| 814 | Fraxiresinol | C21H24O8 | [M+H]+ | Lignans and Coumarins | 1783 | 4-Hydroxy-3-methoxymandelate | C9H10O5 | [M-H]- | Phenolic acids |
| 815 | Lirioresinol A | C22H26O8 | [M-H]- | Lignans and Coumarins | 1784 | Ethyl maltol | C7H8O3 | [M+H]+ | Phenolic acids |
| 816 | Matairesinol-4'-O-glucoside (Matairesinoside) | C26H32O11 | [M+NH4]+ | Lignans and Coumarins | 1785 | 3,4,5-Tri-O-Galloylshikimic acid | C28H22O17 | [M-H]- | Phenolic acids |
| 817 | 2-[(1s,2r,3r)-1-hydroxy-4-(3-hydroxy-4,5-dimethoxyphenyl)-2,3-dimethylbutyl]-4,5-dimethoxyphenol | C22H30O7 | [M+H]+ | Lignans and Coumarins | 1786 | Tetragallic Acid(3''-O-galloyl-4''-O-galloyl-4-O-galloyl-galloyl acid) | C28H18O17 | [M-H]- | Phenolic acids |
| 818 | 5-[(2r,3s,4s,5s)-5-(2h-1,3-benzodioxol-5-yl)-3,4-dimethyloxolan-2-yl]-2-methoxyphenol | C20H22O5 | [M+H]+ | Lignans and Coumarins | 1787 | 1,3,4,6-Tetra-O-Galloyl-D-Glucose | C34H28O22 | [M-H]- | Phenolic acids |
| 819 | 5'-Methoxyisolariciresinol-9'-O-glucoside | C27H36O12 | [M-H]- | Lignans and Coumarins | 1788 | Proglobeflowery acid | C13H16O4 | [M-H]- | Phenolic acids |
| 820 | Sesamol | C7H6O3 | [M+H]+ | Lignans and Coumarins | 1789 | 1,6-Di-O-galloyl-β-D-glucose | C20H20O14 | [M+H]+ | Phenolic acids |
| 821 | Balanophonin | C20H20O6 | [M+H]+ | Lignans and Coumarins | 1790 | Lithospermic acid B* | C36H30O16 | [M-H]- | Phenolic acids |
| 822 | Schisantherin E | C30H34O9 | [M+H]+ | Lignans and Coumarins | 1791 | Salvianolic acid L* | C36H30O16 | [M-H]- | Phenolic acids |
| 823 | Nortrachelogenin-4'-O-gentiobioside* | C32H42O17 | [M-H]- | Lignans and Coumarins | 1792 | Oleoacteoside | C46H58O25 | [M-H]- | Phenolic acids |
| 824 | [(2S,3S)-2-(3,4-dimethoxyphenyl)-7-methoxy-5-prop-2-enyl-2,3-dihydro-1-benzofuran-3-yl]methanol | C21H24O5 | [M+H]+ | Lignans and Coumarins | 1793 | 4-O-(6'-O-Glucosylcaffeoylglucosylferuloyl)-4-hydroxybenzyl alcohol | C38H42O18 | [M-H]- | Phenolic acids |
| 825 | Matairesinol | C20H22O6 | [M+H]+ | Lignans and Coumarins | 1794 | 3,5-Dihydroxy-4-methoxybenzoic acid; 4-O-Methylgallic Acid | C8H8O5 | [M-H]- | Phenolic acids |
| 826 | 1-Hydroxypineolin Diglucoside* | C32H42O17 | [M-H]- | Lignans and Coumarins | 1795 | 1,4-Di-O-galloyl-2-O-p-coumaroyl-β-D-glucose* | C29H26O16 | [M-H]- | Phenolic acids |
| 827 | wulignan A2 | C20H22O5 | [M-H]- | Lignans and Coumarins | 1796 | 1,2,3-Tri-O-galloyl-β-D-glucose* | C27H24O18 | [M-H]- | Phenolic acids |
| 828 | Erythro-Guaiacylglycerol-β-dihydroconiferyl Ether | C20H26O7 | [M-H]- | Lignans and Coumarins | 1797 | Gallic Acid Ethyl Ester; Ethyl gallate | C9H10O5 | [M-H]- | Phenolic acids |
| 829 | 5'-Methoxyisolariciresinol-9'-O-xyloside | C26H34O11 | [M-H]- | Lignans and Coumarins | 1798 | 1,6-Di-O-galloyl-2-O-p-coumaroyl-β-D-glucose* | C29H26O16 | [M-H]- | Phenolic acids |
| 830 | Nortrachelogenin | C20H22O7 | [M+H]+ | Lignans and Coumarins | 1799 | 1,2,6-Tri-O-galloyl-β-D-glucose* | C27H24O18 | [M-H]- | Phenolic acids |
| 831 | Tracheloside | C27H34O12 | [M-H]- | Lignans and Coumarins | 1800 | 2,4,6-Tri-O-galloyl-D-glucose* | C27H24O18 | [M-H]- | Phenolic acids |
| 832 | 4-HydroxyseSamin | C20H18O7 | [M+H]+ | Lignans and Coumarins | 1801 | 1,4,6-Tri-O-galloyl-β-D-glucose* | C27H24O18 | [M-H]- | Phenolic acids |
| 833 | Syringaresinol | C22H26O8 | [M-H]- | Lignans and Coumarins | 1802 | 2,3-O-Digalloyl-1,4,6-tri-O-galloyl-glucose | C55H40O34 | [M-H]- | Phenolic acids |
| 834 | Syringaresinol-4'-O-glucoside; Acanthoside B | C28H36O13 | [M-H]- | Lignans and Coumarins | 1803 | Leonuriside A | C14H20O9 | [M-H]- | Phenolic acids |
| 835 | Hinokinin | C20H18O6 | [M+H]+ | Lignans and Coumarins | 1804 | 1,3,6-Tri-O-galloyl-β-D-glucose* | C27H24O18 | [M-H]- | Phenolic acids |
| 836 | trans-1,2-dihydrodehydroguaiaretic acid* | C20H22O4 | [M+H]+ | Lignans and Coumarins | 1805 | Isochlorogenic acid C* | C25H24O12 | [M-H]- | Phenolic acids |
| 837 | (7S,8S-Threo-3',4,7,9-tetrahydroxy-3-methoxy-8-O-4'-neolignan-9'-O-rhamnoside | C25H34O11 | [M-H]- | Lignans and Coumarins | 1806 | Dicaffeoylshikimic acid | C25H22O11 | [M+H]+ | Phenolic acids |
| 838 | (3r,4r)-3-(2h-1,3-benzodioxol-5-ylmethyl)-4-[(4-hydroxy-3-methoxyphenyl)methyl]oxolan-2-one* | C20H20O6 | [M+H]+ | Lignans and Coumarins | 1807 | Feruloylmalic acid | C14H14O8 | [M-H]- | Phenolic acids |
| 839 | Pinoresinol* | C20H22O6 | [M-H]- | Lignans and Coumarins | 1808 | 2-[2-(2-Hydroxyphenyl)ethyl]-4,6-dimethoxyphenol | C16H18O4 | [M+H]+ | Phenolic acids |
| 840 | Erythro-Guaiacylglycerol-β-O-4'-dehydrodisinapyl Ether | C31H36O11 | [M-H]- | Lignans and Coumarins | 1809 | Isochlorogenic acid A* | C25H24O12 | [M-H]- | Phenolic acids |
| 841 | Chicanin | C20H22O5 | [M+H]+ | Lignans and Coumarins | 1810 | 4,5-O-Dicaffeoylquinic Acid Methyl Ester | C26H26O12 | [M-H]- | Phenolic acids |
| 842 | Olivil-4'-O-glucoside | C26H34O12 | [M-H]- | Lignans and Coumarins | 1811 | 3,6'-Diferuloylsucrose | C32H38O17 | [M-H]- | Phenolic acids |
| 843 | Epipinoresinol* | C20H22O6 | [M-H]- | Lignans and Coumarins | 1812 | Procyanidin B4 | C30H26O12 | [M-H]- | Tannins |
| 844 | 5'-Methoxymatairesinoside | C27H34O12 | [M-H]- | Lignans and Coumarins | 1813 | Procyanidin C1 | C45H38O18 | [M-H]- | Tannins |
| 845 | Isolariciresinol-9-O-xyloside | C25H32O10 | [M-H]- | Lignans and Coumarins | 1814 | 2α,3α-Epoxy-5,7,3',4'-tetrahydroxyflavan-(4β-8-catechin)* | C30H24O12 | [M-H]- | Tannins |
| 846 | Eucommin A | C27H34O12 | [M-H]- | Lignans and Coumarins | 1815 | Procyanidin C2 | C45H38O18 | [M-H]- | Tannins |
| 847 | Lyoniresinol-3α-O-glucoside | C28H38O13 | [M-H]- | Lignans and Coumarins | 1816 | Procyanidin A2* | C30H24O12 | [M-H]- | Tannins |
| 848 | Meso-dihydroguaiaretic acid | C20H26O4 | [M+H]+ | Lignans and Coumarins | 1817 | Procyanidin B2 | C30H26O12 | [M+H]+ | Tannins |
| 849 | Lariciresinol | C20H24O6 | [M+H]+ | Lignans and Coumarins | 1818 | Procyanidin B5 | C30H26O12 | [M+H]+ | Tannins |
| 850 | Isolariciresinol | C20H24O6 | [M-H]- | Lignans and Coumarins | 1819 | Procyanidin B3 | C30H26O12 | [M-H]- | Tannins |
| 851 | 9-hydroxysesamin | C20H18O7 | [M-H]- | Lignans and Coumarins | 1820 | Procyanidin A1* | C30H24O12 | [M-H]- | Tannins |
| 852 | Austrobailignan-5* | C20H22O4 | [M+H]+ | Lignans and Coumarins | 1821 | Procyanidin A6 | C31H28O12 | [M-H]- | Tannins |
| 853 | Secoisolariciresinol diglucoside | C32H46O16 | [M-H]- | Lignans and Coumarins | 1822 | Galloylprocyanidin B4 | C37H30O16 | [M-H]- | Tannins |
| 854 | 3,4-Dihydro-4-(4-hydroxy-3-methoxyphenyl)-3-(hydroxymethyl)-6,7-dimethoxy-(3R,4S)-2-naphthalenecarboxaldehyde | C21H22O6 | [M-H]- | Lignans and Coumarins | 1823 | Procyanidin B1 | C30H26O12 | [M-H]- | Tannins |
| 855 | Medioresinol-4,4'-di-O-glucoside | C33H44O17 | [M-H]- | Lignans and Coumarins | 1824 | Proanthocyanidins | C30H26O13 | [M-H]- | Tannins |
| 856 | Piperitol | C20H20O6 | [M-H]- | Lignans and Coumarins | 1825 | Galloylprocyanidin C2 | C52H42O22 | [M+H]+ | Tannins |
| 857 | fragransol D | C21H22O6 | [M+H]+ | Lignans and Coumarins | 1826 | Procyanidin C1 3'-O-gallate | C52H42O22 | [M+H]+ | Tannins |
| 858 | Verrucosin | C20H24O5 | [M+H]+ | Lignans and Coumarins | 1827 | Theaflavin | C29H24O12 | [M+H]+ | Tannins |
| 859 | (5R,6S,7R)-5-(3,4,5-Trimethoxyphenyl)-7-methyl-8-oxo-5,6,7,8-tetrahydronaphtho[2,3-d]-1,3-dioxole-6-carboxylic acidethylester | C24H26O8 | [M+H]+ | Lignans and Coumarins | 1828 | Arecatannin C1 | C45H38O18 | [M+H]+ | Tannins |
| 860 | 4-(5-(3,4-dimethoxyphenyl)-3,4-dimethyltetrahydrofuran-2-yl)benzene-1,2-diol | C20H24O5 | [M+H]+ | Lignans and Coumarins | 1829 | Aesculitannin B | C45H36O18 | [M+H]+ | Tannins |
| 861 | 7',8'-Dihydro-7'-(5'-hydroxy-3'-methoxyphenyl)-3-methoxy-8'-methyl-1-(E)-propenylbenzofuran | C20H22O4 | [M+H]+ | Lignans and Coumarins | 1830 | Arecatannin B1 | C45H38O18 | [M+H]+ | Tannins |
| 862 | saucerneol J | C20H22O5 | [M+H]+ | Lignans and Coumarins | 1831 | Cinnamtannin A2 | C60H50O24 | [M-H]- | Tannins |
| 863 | Olivil | C20H24O7 | [M-H]- | Lignans and Coumarins | 1832 | Gambiriin A1 | C30H28O12 | [M+H]+ | Tannins |
| 864 | Medioresinol-4'-O-(6'''-acetyl)glucoside | C29H36O13 | [M-H]- | Lignans and Coumarins | 1833 | Cinnamtannin B1 | C45H36O18 | [M-H]- | Tannins |
| 865 | (7S,7'R,8S,8'R)-4,4'-Dihydroxy-3,5,3'-trimethoxy-7,7'-epoxylignan | C21H26O6 | [M+H]+ | Lignans and Coumarins | 1834 | Cinnamtannin B2 | C60H50O24 | [M-H]- | Tannins |
| 866 | 2,6-Dimethoxy-4-(7-methoxy-3-methyl-5-prop-1-enyl-2,3-dihydro-1-benzofuran-2-yl)phenol | C21H24O5 | [M+H]+ | Lignans and Coumarins | 1835 | Cinnamtannin D1 | C45H36O18 | [M-H]- | Tannins |
| 867 | tetrahydrofuroguaiacin B* | C20H24O5 | [M+H]+ | Lignans and Coumarins | 1836 | Arecatannin A2 | C60H50O24 | [M+H]+ | Tannins |
| 868 | 4-ketopinoresinol | C20H20O7 | [M-H]- | Lignans and Coumarins | 1837 | 3-O-Methylellagic acid-4'-O-(5''-O-acetyl)arabinoside | C22H18O13 | [M+H]+ | Tannins |
| 869 | 7S,8R-threo-3',9,9'-trihydroxy-3-methoxy-4',7-epoxy-neolignan-4-O-rhamnoside | C25H32O10 | [M-H]- | Lignans and Coumarins | 1838 | 3,3',4-O-Trimethylellagic acid | C17H12O8 | [M+H]+ | Tannins |
| 870 | 4-[3-(hydroxymethyl)-7-methoxy-5-(prop-2-en-1-yl)-2,3-dihydro-1-benzofuran-2-yl]-2-methoxyphenol | C20H22O5 | [M-H]- | Lignans and Coumarins | 1839 | 3-O-Methylellagic acid | C15H8O8 | [M-H]- | Tannins |
| 871 | licarin A* | C20H22O4 | [M+H]+ | Lignans and Coumarins | 1840 | Gambiriin B3 | C30H26O11 | [M+H]+ | Tannins |
| 872 | Machilin D* | C20H24O5 | [M+H]+ | Lignans and Coumarins | 1841 | 3,4,5,3',4',5'-Hexahydroxydiphenoyl Acid-glucose | C20H18O14 | [M-H]- | Tannins |
| 873 | Saururinone | C20H20O5 | [M+H]+ | Lignans and Coumarins | 1842 | 3'-O-Methyl-4-O-(beta-D-xylopyranosyl)ellagic acid* | C20H16O12 | [M-H]- | Tannins |
| 874 | taiwanin A | C20H14O6 | [M+H]+ | Lignans and Coumarins | 1843 | Ellagic acid-4-O-rhamnoside* | C20H16O12 | [M-H]- | Tannins |
| 875 | Machilin A* | C20H22O4 | [M+H]+ | Lignans and Coumarins | 1844 | Flavogallonic Acid Dilactone | C21H10O12 | [M-H]- | Tannins |
| 876 | (7R,8R)-7,8-dihydro-7-(3,4-dihydroxyphenyl)-3'-methoxy-8-methyl-1'-(E-propenyl)benzofuran | C19H20O4 | [M-H]- | Lignans and Coumarins | 1845 | Eschweilenol C* | C20H16O12 | [M-H]- | Tannins |
| 877 | Dehydrodiconiferyl alcohol-gamma'-O-glucoside | C26H32O11 | [M+H]+ | Lignans and Coumarins | 1846 | Gallic acid-1-O-xyloside | C12H14O9 | [M-H]- | Tannins |
| 878 | Licarin B* | C20H20O4 | [M+H]+ | Lignans and Coumarins | 1847 | Isocorilagin* | C27H22O18 | [M-H]- | Tannins |
| 879 | otobain* | C20H20O4 | [M+H]+ | Lignans and Coumarins | 1848 | Phyllanemblinin B* | C27H22O18 | [M-H]- | Tannins |
| 880 | 4-[4-(4-hydroxy-3-methoxyphenyl)-2,3-dimethylbutyl]-2-methoxyphenol | C20H26O4 | [M-H]- | Lignans and Coumarins | 1849 | Strictinin* | C27H22O18 | [M-H]- | Tannins |
| 881 | Otobaphenol* | C20H22O4 | [M+H]+ | Lignans and Coumarins | 1850 | Corilagin* | C27H22O18 | [M-H]- | Tannins |
| 882 | Dehydrodiisoeugenol* | C20H22O4 | [M+H]+ | Lignans and Coumarins | 1851 | Gemin D | C27H22O18 | [M-H]- | Tannins |
| 883 | Palmitaldehyde | C16H32O | [M-H]- | Lipids | 1852 | Sanguiin H4* | C27H22O18 | [M-H]- | Tannins |
| 884 | E,E,Z-1,3,12-Nonadecatriene-5,14-diol | C19H34O2 | [M-H]- | Lipids | 1853 | (1R,3E,7E,11S,12R)-Dolabella-3,7-dien-18-ol | C20H34O | [M+H]+ | Terpenoids |
| 885 | Elaidic Acid* | C18H34O2 | [M-H]- | Lipids | 1854 | (1S,3R,3a1S,8R,10S,11aS)-1,5,10-trihydroxy-3,3a1,8,11a-tetramethyl-2,11-dioxohexadecahydrodibenzo[de,g]chromen-4-yl acetate | C22H32O8 | [M+H]+ | Terpenoids |
| 886 | Octadeca-11E,13E,15Z-trienoic acid | C18H30O2 | [M+H]+ | Lipids | 1855 | [(1S,2S,3S,7R,9R,13S,14R,15R,17S)-3,15-diacetyloxy-7-hydroxy-2,6,14-trimethyl-4,11,16-trioxo-10-oxatetracyclo[7.7.1.02,7.013,17]heptadec-5-en-17-yl]methylacetate* | C26H32O11 | [M-H]- | Terpenoids |
| 887 | Undecylic Acid | C11H22O2 | [M-H]- | Lipids | 1856 | Isopimaric acid* | C20H30O2 | [M-H]- | Terpenoids |
| 888 | Methyl palmitate | C17H34O2 | [M-H]- | Lipids | 1857 | Pimaric acid* | C20H30O2 | [M-H]- | Terpenoids |
| 889 | 12-Hydroxy-16-heptadecynoic acid | C17H30O3 | [M-H]- | Lipids | 1858 | cis-Abienol | C20H34O | [M-H]- | Terpenoids |
| 890 | 3-Hydroxyoctadecanoic Acid | C18H36O3 | [M-H]- | Lipids | 1859 | 5-[2-(furan-3-yl)ethyl]-5,6,8a-trimethyl-3,4,4a,6,7,8-hexahydronaphthalene-1-carboxylic acid | C20H28O3 | [M-H]- | Terpenoids |
| 891 | Heptadecanoic acid | C17H34O2 | [M-H]- | Lipids | 1860 | (-)-Oleoside 11-methyl ester* | C17H24O11 | [M-H]- | Terpenoids |
| 892 | Tridecanedioic acid | C13H24O4 | [M-H]- | Lipids | 1861 | (1S,4aS,7aR)-7-(3-hydroxybutyl)-4-((((2R,3R,4S,5S,6R)-3,4,5-trihydroxy-6-(hydroxymethyl)tetrahydro-2H-pyran-2-yl)oxy)methyl)-1,4a,5,6,7,7a-hexahydrocyclopenta[c]pyran-1-yl acetate | C21H34O10 | [M-H]- | Terpenoids |
| 893 | α-Linolenic acid* | C18H30O2 | [M+H]+ | Lipids | 1862 | Bartsioside | C15H22O8 | [M+H]+ | Terpenoids |
| 894 | methyl-8,11,14-heptadecatrienoate* | C18H30O2 | [M+H]+ | Lipids | 1863 | Nuezhengalaside* | C18H28O9 | [M-H]- | Terpenoids |
| 895 | 11-Octadecanoic acid(Vaccenic acid)* | C18H34O2 | [M-H]- | Lipids | 1864 | 6-DeoxyCatalpol | C15H22O9 | [M-H]- | Terpenoids |
| 896 | Petroselinic acid* | C18H34O2 | [M-H]- | Lipids | 1865 | Genipin | C11H14O5 | [M+H]+ | Terpenoids |
| 897 | 13(S)-HODE;13(S)-Hydroxyoctadeca-9Z,11E-dienoic acid* | C18H32O3 | [M-H]- | Lipids | 1866 | cis-Citral | C10H16O | [M+H]+ | Terpenoids |
| 898 | 9S-Hydroxy-10E,12Z-octadecadienoic acid* | C18H32O3 | [M-H]- | Lipids | 1867 | Gardenoside | C17H24O11 | [M+NH4]+ | Terpenoids |
| 899 | 7S,8S-DiHODE; (9Z,12Z)-(7S,8S)-Dihydroxyoctadeca-9,12-dienoic acid* | C18H32O4 | [M-H]- | Lipids | 1868 | 8-Epiloganic acid | C16H24O10 | [M-H]- | Terpenoids |
| 900 | 13-Hydroperoxy-9Z,11E-octadecadienoic acid* | C18H32O4 | [M-H]- | Lipids | 1869 | Blumenol A | C13H20O3 | [M+H]+ | Terpenoids |
| 901 | Hydroxypentadecenoic acid glucoside | C21H38O8 | [M+CH3COOH-H]- | Lipids | 1870 | Geniposidic acid | C16H22O10 | [M-H]- | Terpenoids |
| 902 | 12(13)Ep-9-KODE | C18H30O4 | [M-H]- | Lipids | 1871 | 6-O-p-Coumaroyl-3-methoxy-7-deoxyrehmaglutin A | C19H22O7 | [M+H]+ | Terpenoids |
| 903 | (+/-)5,6-EET Methyl Ester | C21H34O3 | [M+H]+ | Lipids | 1872 | 6-O-Trans-Caffeoyl Ajugol | C24H30O12 | [M-H]- | Terpenoids |
| 904 | Palmitic acid | C16H32O2 | [M-H]- | Lipids | 1873 | Ajugoside | C17H26O10 | [M-H]- | Terpenoids |
| 905 | 9,16-Dihydroxypalmitic acid | C16H32O4 | [M-H]- | Lipids | 1874 | Frehmaglutoside G | C21H36O9 | [M-H]- | Terpenoids |
| 906 | 15(R)-Hydroxylinoleic Acid | C18H32O3 | [M+H]+ | Lipids | 1875 | 2'-O-(3''-hydroxybenzoyl)-kingiside | C24H28O13 | [M-H]- | Terpenoids |
| 907 | 2-[(1R,2R)-3-oxo-2-[(Z)-5-[3,4,5-trihydroxy-6-(hydroxymethyl)oxan-2-yl]oxypent-2-enyl]cyclopentyl]acetic acid | C18H28O9 | [M-H]- | Lipids | 1876 | Blumenol C | C13H22O2 | [M+H]+ | Terpenoids |
| 908 | 13(s)-hydroperoxy-(9z,11e,15z)-octadecatrienoic acid | C18H30O4 | [M-H]- | Lipids | 1877 | 2,6-Dimethyl-6-hydroxy-2,7-octadienyl-β-D-glucoside (Betulalbuside A; Betulabuside A) | C16H28O7 | [M+H]+ | Terpenoids |
| 909 | Eicosenoic acid | C20H38O2 | [M-H]- | Lipids | 1878 | Penstemonoside | C17H26O10 | [M+NH4]+ | Terpenoids |
| 910 | 9,10,13-Trihydroxy-11-Octadecenoic Acid | C18H34O5 | [M-H]- | Lipids | 1879 | Germacra-1(10),4,7(11)-trien-9α-ol | C15H24O | [M+H]+ | Terpenoids |
| 911 | Arachidic acid | C20H40O2 | [M-H]- | Lipids | 1880 | 10-Deoxygeniposidic acid | C16H22O9 | [M+H]+ | Terpenoids |
| 912 | 1-Octadecanol | C18H38O | [M-H]- | Lipids | 1881 | 6-O-p-Methoxycinnamoylcatalpol | C25H30O12 | [M+H]+ | Terpenoids |
| 913 | 13S-Hydroxy-9Z,11E,15Z-octadecatrienoic acid | C18H30O3 | [M-H]- | Lipids | 1882 | p-Menth-4(5)-ene-1,2-diol-1-O-β-D-(2-O-acetyl)-glucoside | C18H30O8 | [M-H]- | Terpenoids |
| 914 | 1,14-Tetradecanedioic Acid | C14H26O4 | [M-H]- | Lipids | 1883 | 6-O-Vanilloylajugol | C23H30O12 | [M-H]- | Terpenoids |
| 915 | (R)-Beta-Hydroxypalmitic Acid* | C16H32O3 | [M-H]- | Lipids | 1884 | 3β,11-Dihydroxy-4,14-oxideenantiocudesmane | C15H26O3 | [M+H]+ | Terpenoids |
| 916 | Methyl linolenate | C19H32O2 | [M+H]+ | Lipids | 1885 | 6β-Hydroxy-8α-methoxyeremophila-1(10),7(11)-dien-12,8β-olide* | C16H22O4 | [M+H]+ | Terpenoids |
| 917 | Hexadecanedioic acid | C16H30O4 | [M-H]- | Lipids | 1886 | 1-O-Acetyl britannilactone | C17H24O5 | [M-H]- | Terpenoids |
| 918 | 2-Hydroxy-4-methyl-3-undecanoyloxypentanoic acid methyl ester | C18H34O5 | [M-H]- | Lipids | 1887 | 30-Hydroxyfraxinellone | C14H16O4 | [M+H]+ | Terpenoids |
| 919 | Dodecanoic acid (Lauric acid) | C12H24O2 | [M-H]- | Lipids | 1888 | 5-hydroxyindene-1,3-dione | C9H6O3 | [M+H]+ | Terpenoids |
| 920 | 4-Oxo-9Z,11Z,13E,15E-Octadecatetraenoic Acid | C18H26O3 | [M+H]+ | Lipids | 1889 | Aucubin | C15H22O9 | [M-H]- | Terpenoids |
| 921 | Monogalactosyldiacylglycerol | C45H70O10 | [M+H]+ | Lipids | 1890 | 7-Hydroxy-costol-glucoside | C21H34O7 | [M+H]+ | Terpenoids |
| 922 | 12,13-DHOME; (9Z)-12,13-Dihydroxyoctadec-9-enoic acid | C18H34O4 | [M-H]- | Lipids | 1891 | Acetylvalerenolic acid | C17H24O4 | [M-H]- | Terpenoids |
| 923 | Undecanedioic acid | C11H20O4 | [M-H]- | Lipids | 1892 | Spathulenol | C15H24O | [M+H]+ | Terpenoids |
| 924 | Docosatetraenoic acid | C22H36O2 | [M-H]- | Lipids | 1893 | Procurcumenol | C15H22O2 | [M+H]+ | Terpenoids |
| 925 | Erucic acid | C22H42O2 | [M-H]- | Lipids | 1894 | 9-Hydroxyselina-4,11-dien-14-al | C15H22O2 | [M+H]+ | Terpenoids |
| 926 | 9-Hydroxyoctadeca-6,10,12,15-Tetraenoic Acid | C18H28O3 | [M-H]- | Lipids | 1895 | (4β,10E)-6α,15-Dihydroxy-8β-(angeloyloxy)-14-oxogermacra-1(10),11(13)-diene-12-oic acid-12,6-lactone | C20H26O6 | [M-H]- | Terpenoids |
| 927 | 5S,8R-DiHODE; (5S,8R,9Z,12Z)-5,8-Dihydroxyoctadeca-9,12-dienoate | C18H32O4 | [M-H]- | Lipids | 1896 | Glutinosone | C14H20O2 | [M+H]+ | Terpenoids |
| 928 | 12S-HHT | C17H28O3 | [M+CH3COO]- | Lipids | 1897 | 6,9-Dihydroxy-7-megastigmen-3-one | C13H22O3 | [M+H]+ | Terpenoids |
| 929 | Ricinoleic acid | C18H34O3 | [M-H]- | Lipids | 1898 | 1β-Hydroxy-α-cyperone | C15H24O2 | [M+H]+ | Terpenoids |
| 930 | 1-Eicosanol | C20H42O | [M-H]- | Lipids | 1899 | (R)-3-(3'-Hydroxybutyl)-2,4,4-trimethylcyclohexa-2,5-dienone | C13H20O2 | [M+H]+ | Terpenoids |
| 931 | 9-Hydroperoxy-10E,12,15Z-octadecatrienoic acid | C18H30O4 | [M-H]- | Lipids | 1900 | Nootkatone | C15H22O | [M+H]+ | Terpenoids |
| 932 | Cis-10-Pentadecenoic Acid(C15:1) | C15H28O2 | [M-H]- | Lipids | 1901 | Reynosin | C15H20O3 | [M+H]+ | Terpenoids |
| 933 | 10-Undecenoic acid | C11H20O2 | [M-H]- | Lipids | 1902 | 10-Dehydrogeniposide | C17H22O10 | [M+H]+ | Terpenoids |
| 934 | 13-methylmyristic acid* | C15H30O2 | [M-H]- | Lipids | 1903 | 10α-Hydroperoxy-guaia-1,11-diene* | C15H24O2 | [M+H]+ | Terpenoids |
| 935 | 10,16-Dihydroxypalmitic acid | C16H32O4 | [M-H]- | Lipids | 1904 | Enantio-7(11)eudesmen-4-ol | C15H26O | [M+H]+ | Terpenoids |
| 936 | Pentadecanoic Acid* | C15H30O2 | [M-H]- | Lipids | 1905 | 1,4-Peroxyaurol-ene* | C15H24O2 | [M+H]+ | Terpenoids |
| 937 | 9,10-DHOME; (12Z)-9,10-Dihydroxyoctadec-12-enoic acid | C18H34O4 | [M-H]- | Lipids | 1906 | 9-O-β-D-Glucoside of 3-hydroxy-7,8-didehydro-β-ionol | C19H30O7 | [M+H]+ | Terpenoids |
| 938 | Stearidonic acid | C18H28O2 | [M+H]+ | Lipids | 1907 | 10α-Hydroxycadin-4-en-15-al | C15H24O2 | [M+H]+ | Terpenoids |
| 939 | Crepenynic acid | C18H30O2 | [M-H]- | Lipids | 1908 | 7-Hydroxy-costol-malonyl glucoside | C24H36O10 | [M+H]+ | Terpenoids |
| 940 | 13S-Hydroperoxy-6Z,9Z,11E-octadecatrienoic acid | C18H30O4 | [M-H]- | Lipids | 1909 | (1R,4aR,7R,8aR)-1,4a,8a-trimethyl-7-(prop-1-en-2-yl)-deca-hydronaphthalen-1-ol | C15H24O | [M+H]+ | Terpenoids |
| 941 | 5,6-DiHETrE[(±)5,6-dihydroxy-8Z,11Z,14Z-eicosatrienoic acid] | C20H34O4 | [M+H]+ | Lipids | 1910 | 2β-Methoxy-14-nor-β-patchoulane-1(5)-ene-4-one | C15H22O2 | [M+H]+ | Terpenoids |
| 942 | DL-2-hydroxystearic acid | C18H36O3 | [M-H]- | Lipids | 1911 | Cinncassiol A | C20H30O7 | [M-H]- | Terpenoids |
| 943 | 11,14,17-Eicosatrienoic acid | C20H34O2 | [M-H]- | Lipids | 1912 | Dihydroisoalantolactone | C15H22O2 | [M+H]+ | Terpenoids |
| 944 | Cis-4,7,10,13,16,19-Docosahexaenoic Acid | C22H32O2 | [M-H]- | Lipids | 1913 | 5-Hydroxy-2-Carbonylcadinane-12,7-lactone | C15H20O4 | [M+H]+ | Terpenoids |
| 945 | Dodecanedioic aicd | C12H22O4 | [M-H]- | Lipids | 1914 | Santalol | C15H24O | [M+H]+ | Terpenoids |
| 946 | 2R-hydroxy-9Z,12Z,15Z-octadecatrienoic acid | C18H30O3 | [M-H]- | Lipids | 1915 | Cinncassiol C1 | C20H28O7 | [M-H]- | Terpenoids |
| 947 | 9,10,11-Trihydroxy-12-octadecenoic acid | C18H34O5 | [M-H]- | Lipids | 1916 | Dehydrololiolide | C11H14O3 | [M-H]- | Terpenoids |
| 948 | 9(10)-EpOME;(9R,10S)-(12Z)-9,10-Epoxyoctadecenoic acid | C18H32O3 | [M-H]- | Lipids | 1917 | 3α,12β,15α,21β,24-pentahydroxyserratane | C30H50O8 | [M+H]+ | Terpenoids |
| 949 | Oleamide (9-Octadecenamide) | C18H35NO | [M+H]+ | Lipids | 1918 | 8-Epiloganin | C17H26O10 | [M-H]- | Terpenoids |
| 950 | 1-Linoleoyl-2-Lysophosphatidic Acid Monobutylamine Ester | C25H48NO7P | [M-H]- | Lipids | 1919 | 6''-O-Acetylharpagide | C17H26O11 | [M-H]- | Terpenoids |
| 951 | Gingerglycolipid A | C33H56O14 | [M-H]- | Lipids | 1920 | Methyl geranate | C11H18O2 | [M+Na]+ | Terpenoids |
| 952 | LysoPG 16:0 | C22H45O9P | [M-H]- | Lipids | 1921 | 23-Hydroxytoonacilide | C31H38O11 | [M-H]- | Terpenoids |
| 953 | 1-α-Linolenoyl-glycerol* | C21H36O4 | [M+H]+ | Lipids | 1922 | 3-Oxolup-20(29)-en-28-oic acid (Betulonic acid) | C30H46O3 | [M-H]- | Terpenoids |
| 954 | Monolinolenin* | C21H36O4 | [M+H]+ | Lipids | 1923 | 3-Oxours-12-en-28-oic acid (Ursonic acid) | C30H46O3 | [M-H]- | Terpenoids |
| 955 | Glycerol 9(E),11(Z),13(E)-octadecatrienoyl ester* | C21H36O4 | [M+H]+ | Lipids | 1924 | 3,19-Epoxy-3,22-dihydroxydammara-20,24-dien-26-oic acid δ-lactone (Semialactone) | C30H44O4 | [M-H]- | Terpenoids |
| 956 | 2-α-Linolenoyl-glycerol* | C21H36O4 | [M+H]+ | Lipids | 1925 | orthosphenic acid | C30H48O5 | [M-H]- | Terpenoids |
| 957 | LysoPG 16:1 | C22H43O9P | [M-H]- | Lipids | 1926 | 3,13,15-Trihydroxyoleanane-12-one | C30H50O4 | [M-H]- | Terpenoids |
| 958 | Gingerglycolipid B | C33H58O14 | [M-H]- | Lipids | 1927 | 2-((3,16-dihydroxy-17-(1-hydroxyethyl)-10,13-dimethyl-2,3,4,5,8,9,10,11,12,13,14,15,16,17-tetradecahydro-1H-cyclopenta[a]phenanthren-2-yl)oxy)-6-(((3,4,5-trihydroxy-6-methyltetrahydro-2H-pyran-2-yl)oxy)methyl)tetrahydro-2H-pyran-3,4,5-triol | C33H54O13 | [M+H]+ | Terpenoids |
| 959 | 1-Oleoyl-Sn-Glycerol | C21H40O4 | [M+H]+ | Lipids | 1928 | celastolide | C30H46O5 | [M-H]- | Terpenoids |
| 960 | 2-Palmitoyl-Sn-Glycerol 3-O-Diglucoside | C31H58O14 | [M-H]- | Lipids | 1929 | 3-Hydroxyurs-12-en-28-oic acid (3-Epiursolic acid) | C30H48O3 | [M-H]- | Terpenoids |
| 961 | 1-O-Linoleoyl-3-O-galactopyranosyl-L-glycerol | C27H48O9 | [M-H]- | Lipids | 1930 | 3-O-methyl furanovibsanin | C26H36O5 | [M+H]+ | Terpenoids |
| 962 | Gingerglycolipid C | C33H60O14 | [M-H]- | Lipids | 1931 | 27,28-Dicarboxyl ursolic acid | C30H46O5 | [M+H]+ | Terpenoids |
| 963 | 1-Palmitoyl-Sn-Glycerol 3-O-Diglucoside | C31H58O14 | [M-H]- | Lipids | 1932 | 26,27-Dihydroxylanosta-7,9(11),24-trien-3-one (Ganoderiol F) | C30H46O3 | [M+H]+ | Terpenoids |
| 964 | PA(18:2/0:0) | C21H39O7P | [M-H]- | Lipids | 1933 | Viburnenone B1 methyl ester | C32H50O6 | [M-H]- | Terpenoids |
| 965 | LysoPC 18:3(2n isomer)* | C26H48NO7P | [M+H]+ | Lipids | 1934 | 2,3,16,21-Tetrahydroxyolean-12-en-28-oic acid (Platycogenic acid C) | C30H48O6 | [M-H]- | Terpenoids |
| 966 | LysoPC 16:0* | C24H50NO7P | [M+H]+ | Lipids | 1935 | 2,3-Dihydroxyurs-12-en-28-oic acid (Corosolic acid) | C30H48O4 | [M+H]+ | Terpenoids |
| 967 | LysoPC 16:0(2n isomer)* | C24H50NO7P | [M+H]+ | Lipids | 1936 | Clavatol | C10H12O3 | [M+H]+ | Terpenoids |
| 968 | LysoPC 18:3* | C26H48NO7P | [M+H]+ | Lipids | 1937 | 4-[5-(3-Hydroxypropyl)-7-methoxy-3-methyl-2,3-dihydro-1-benzofuran-2-yl]-2-methoxyphenol | C20H24O5 | [M+H]+ | Terpenoids |
| 969 | LysoPC 18:1(2n isomer) | C26H52NO7P | [M+H]+ | Lipids |  |  |  |  |  |

**Supplementary Table S3.** Total of 1364 DAMs in different tissues of *Machilus nanmu*.

| NO. | Compounds | Sub class | Formula | Class I | NO. | Compounds | Sub class | Formula | Class I |
| --- | --- | --- | --- | --- | --- | --- | --- | --- | --- |
| 1 | Agmatine | 1 | C5H14N4 | Alkaloids | 683 | Sexangularetin-3-O-glucoside-7-O-rhamnoside | 7 | C28H32O16 | Flavonoids |
| 2 | 2-propenamide* | 1 | C19H21NO5 | Alkaloids | 684 | 6-Hydroxykaempferol-3,6-O-Diglucoside* | 7 | C27H30O17 | Flavonoids |
| 3 | 4-Methylazetidine-2-Carboxylic acid* | 1 | C5H9NO2 | Alkaloids | 685 | Quercetin-3-O-Sambubioside-5-O-Glucoside | 7 | C32H38O21 | Flavonoids |
| 4 | Demethylcoclaurine* | 1 | C16H17NO3 | Alkaloids | 686 | 2,5-dihydroxy-1,6-dimethoxy-8-{[(2s,3r,4s,5s,6r)-3,4,5-trihydroxy-6-(hydroxymethyl)oxan-2-yl]oxy}xanthen-9-one | 7 | C21H22O12 | Flavonoids |
| 5 | Hercynine | 1 | C9H15N3O2 | Alkaloids | 687 | 2,3,5-trimethoxy-1-{[(2s,3r,4s,5s,6r)-3,4,5-trihydroxy-6-(hydroxymethyl)oxan-2-yl]oxy}xanthen-9-one* | 7 | C22H24O11 | Flavonoids |
| 6 | Pantetheine | 1 | C11H22N2O4S | Alkaloids | 688 | 1,2,3,7,8-pentahydroxy-6-methylanthracene-9,10-dione | 7 | C15H10O7 | Flavonoids |
| 7 | 1-methoxy-6-methyl-10-propan-2-yloxy-5,6,6a,7-tetrahydro-4H-dibenzo[de,g]quinoline-2,9-diol* | 1 | C21H25NO4 | Alkaloids | 689 | 1,2,4,5-tetrahydroxy-7-(hydroxymethyl)anthracene-9,10-dione | 7 | C15H10O7 | Flavonoids |
| 8 | N-Acetylputrescine | 1 | C6H14N2O | Alkaloids | 690 | Angelicin* | 7 | C11H6O3 | Lignans and Coumarins |
| 9 | Isorescinnamine, dihydro- | 1 | C35H44N2O9 | Alkaloids | 691 | Psoralen* | 7 | C11H6O3 | Lignans and Coumarins |
| 10 | 2-(4-((6,7-dihydroxy-5-methoxy-1,2,3,4-tetrahydroisoquinolin-1-yl)methyl)phenoxy)-6-(hydroxymethyl)tetrahydro-2H-pyran-3,4,5-triol | 1 | C23H29NO9 | Alkaloids | 692 | Sideretin (5,7,8-Trihydroxy-6-methoxycoumarin) | 7 | C10H8O6 | Lignans and Coumarins |
| 11 | L-Carnitine | 1 | C7H15NO3 | Alkaloids | 693 | Daphnin* | 7 | C15H16O9 | Lignans and Coumarins |
| 12 | (E)-1-(3,4-dimethoxybenzylidene)-6,7-dimethoxy-2-methyl-1,2,3,4-tetrahydroisoquinoline* | 1 | C21H25NO4 | Alkaloids | 694 | Cichoriin* | 7 | C15H16O9 | Lignans and Coumarins |
| 13 | 3-(1H-imidazol-5-yl)-2-(trimethylammonio)propanoate | 1 | C9H15N3O2 | Alkaloids | 695 | Esculetin-7-O-glucoside* | 7 | C15H16O9 | Lignans and Coumarins |
| 14 | Hetidine | 1 | C21H27NO4 | Alkaloids | 696 | Scopoletin-7-O-xylosyl(1→6)glucoside | 7 | C21H26O13 | Lignans and Coumarins |
| 15 | N-Methyl-coclaurine | 1 | C18H21NO3 | Alkaloids | 697 | 3,4-Dihydro-4-(4'-hydroxyphenyl)-5,7-dihydroxycoumarin glucoside* | 7 | C21H22O10 | Lignans and Coumarins |
| 16 | Caseadine* | 1 | C20H23NO4 | Alkaloids | 698 | 6,8-Dihydroxy-3-methylisocoumarin | 7 | C10H8O4 | Lignans and Coumarins |
| 17 | Anthriscifoldine B | 1 | C25H39NO7 | Alkaloids | 699 | Fraxidin-8-O-glucoside | 7 | C17H20O10 | Lignans and Coumarins |
| 18 | Huangjinjian | 1 | C19H21NO4 | Alkaloids | 700 | Dihydrodehydrodiconiferyl alcohol | 7 | C20H24O6 | Lignans and Coumarins |
| 19 | laudanine* | 1 | C20H25NO4 | Alkaloids | 701 | Lariciresinol-4'-O-glucoside* | 7 | C26H34O11 | Lignans and Coumarins |
| 20 | (R)-1,10-Dimethoxy-6-methyl-5,6,6a,7-tetrahydro-4H-dibenzo[de,g]quinoline-2,9-diol | 1 | C19H21NO4 | Alkaloids | 702 | Dehydrodiconiferyl alcohol | 7 | C20H22O6 | Lignans and Coumarins |
| 21 | N-[7'-(4'-Methoxyphenyl)ethyl]-2-methoxybenzamide | 1 | C17H19NO3 | Alkaloids | 703 | Pinoresinol-4-O-glucoside* | 7 | C26H32O11 | Lignans and Coumarins |
| 22 | 6-ethyl-1,10-dimethoxy-5,6,6a,7-tetrahydro-4H-dibenzo[de,g]quinoline-2,9-diol | 1 | C20H23NO4 | Alkaloids | 704 | Tracheloside | 7 | C27H34O12 | Lignans and Coumarins |
| 23 | 3-amino-2-naphthoic acid* | 1 | C11H9NO2 | Alkaloids | 705 | Lyoniresinol | 7 | C22H28O8 | Lignans and Coumarins |
| 24 | (4,13-Dimethoxy-17-methyl-12-oxo-5-phenylmethoxy-17-azatetracyclo[7.5.3.01,10.02,7]heptadeca-2,4,6,10,13-pentaen-3-yl)acetate | 1 | C28H29NO6 | Alkaloids | 706 | Matairesinol-4'-O-glucoside (Matairesinoside) | 7 | C26H32O11 | Lignans and Coumarins |
| 25 | Laurotetanine | 1 | C19H21NO4 | Alkaloids | 707 | epieudesmin | 7 | C22H26O6 | Lignans and Coumarins |
| 26 | Anaxagoreine | 1 | C17H17NO3 | Alkaloids | 708 | 5'-Methoxymatairesinoside | 7 | C27H34O12 | Lignans and Coumarins |
| 27 | Lauroscholtzine; N-Methyllaurotetanine | 1 | C20H23NO4 | Alkaloids | 709 | 1,14-Tetradecanedioic Acid | 7 | C14H26O4 | Lipids |
| 28 | 1,2,10-trimethoxy-6-methyl-5,6,6a,7-tetrahydro-4H-dibenzo[de,g]quinolin-11-ol | 1 | C20H23NO4 | Alkaloids | 710 | 12,13-DHOME; (9Z)-12,13-Dihydroxyoctadec-9-enoic acid | 7 | C18H34O4 | Lipids |
| 29 | Norisoboldine | 1 | C18H19NO4 | Alkaloids | 711 | Crepenynic acid | 7 | C18H30O2 | Lipids |
| 30 | Higenamine* | 1 | C16H17NO3 | Alkaloids | 712 | 12S-HHT | 7 | C17H28O3 | Lipids |
| 31 | 2-methyl-1betah-coclaurine | 1 | C18H21NO3 | Alkaloids | 713 | 2-Hydroxy-4-methyl-3-undecanoyloxypentanoic acid methyl ester | 7 | C18H34O5 | Lipids |
| 32 | (1s)-7-methoxy-1-[(4-methoxyphenyl)methyl]-2-methyl-3,4-dihydro-1h-isoquinolin-6-ol | 1 | C19H23NO3 | Alkaloids | 714 | Gingerglycolipid A | 7 | C33H56O14 | Lipids |
| 33 | 1-(4-hydroxyphenyl)-7-methoxy-1,2,3,4-tetrahydroisoquinolin-8-ol* | 1 | C16H17NO3 | Alkaloids | 715 | 1-Palmitoyl-Sn-Glycerol 3-O-Diglucoside | 7 | C31H58O14 | Lipids |
| 34 | 3',6-Dihydroxy-4',7-dimethoxyl-N,N-dimethyltetrahydroisoquinoline* | 1 | C20H25NO4 | Alkaloids | 716 | LysoPE 18:3* | 7 | C23H42NO7P | Lipids |
| 35 | 6-Acetylmorphine | 1 | C19H21NO4 | Alkaloids | 717 | Choline Alfoscerate | 7 | C8H20NO6P | Lipids |
| 36 | 6'-hydroxy-3,5'-dimethoxy-1'-methyl-2',3',8',8a'-tetrahydro-1'H-spiro[cyclohexane-1,7'-cyclopenta[ij]isoquinolin]-2-en-4-one | 1 | C19H23NO4 | Alkaloids | 718 | 2-Aminopurine | 7 | C5H5N5 | Nucleotides and derivatives |
| 37 | (S)-2,10,11-trimethoxy-6-methyl-5,6,6a,7-tetrahydro-4H-dibenzo[de,g]quinolin-1-ol* | 1 | C20H23NO4 | Alkaloids | 719 | 9-Alpha-Ribofuranosyladenine* | 7 | C10H13N5O4 | Nucleotides and derivatives |
| 38 | 1-[(4-hydroxyphenyl)methyl]-7-methoxy-1,2,3,4-tetrahydroisoquinolin-8-ol | 1 | C17H19NO3 | Alkaloids | 720 | Vidarabine* | 7 | C10H13N5O4 | Nucleotides and derivatives |
| 39 | 1-[(4-methoxyphenyl)methyl]-1,2,3,4-tetrahydroisoquinoline-6,7-diol* | 1 | C17H19NO3 | Alkaloids | 721 | Adenosine* | 7 | C10H13N5O4 | Nucleotides and derivatives |
| 40 | Coclaurine* | 1 | C17H19NO3 | Alkaloids | 722 | 2-Deoxyribose-1-phosphate* | 7 | C5H11O7P | Nucleotides and derivatives |
| 41 | 1-[(4-hydroxyphenyl)methyl]-6-methoxy-2-methyl-3,4-dihydro-1h-isoquinolin-7-ol | 1 | C18H21NO3 | Alkaloids | 723 | 2-Deoxyribose-5'-phosphate* | 7 | C5H11O7P | Nucleotides and derivatives |
| 42 | N-Trans-Feruloyl-3'-O-methyldopamine* | 1 | C19H21NO5 | Alkaloids | 724 | 2'-Deoxyadenosine-5'-monophosphate | 7 | C10H14N5O6P | Nucleotides and derivatives |
| 43 | N-Feruloyltyramine; Moupinamide | 1 | C18H19NO4 | Alkaloids | 725 | 2,6-Diaminooimelic acid | 7 | C7H14N2O4 | Organic acids |
| 44 | N-Feruloyl-3-methoxytyramine* | 1 | C19H21NO5 | Alkaloids | 726 | DL-Glyceraldehyde-3-phosphate | 7 | C3H7O6P | Organic acids |
| 45 | N-Trans-Sinapoyltyramine | 1 | C19H21NO5 | Alkaloids | 727 | 2-Hydroxyethylphosphonic acid | 7 | C2H7O4P | Organic acids |
| 46 | Feruloylhistamine | 1 | C15H17N3O3 | Alkaloids | 728 | Oroxylin A-7-o-beta-D-glucuronide | 7 | C22H20O11 | Organic acids |
| 47 | Grossamide | 1 | C36H36N2O8 | Alkaloids | 729 | Mevalonic acid | 7 | C6H12O4 | Organic acids |
| 48 | 4-Hydroxymandelonitrile | 1 | C8H7NO2 | Alkaloids | 730 | Quinic Acid | 7 | C7H12O6 | Organic acids |
| 49 | Hordenine | 1 | C10H15NO | Alkaloids | 731 | 2-Oxoadipic acid | 7 | C6H8O5 | Organic acids |
| 50 | 1-(Dihydroxyphenyl)-N2,N3-bis(4-hydroxyphenethyl)-(5-8)-dimethoxy-1,2dihydronaphthalene-2,3-dicarboxamide | 1 | C36H36N2O8 | Alkaloids | 732 | 2-Hydroxyphenylacetic acid | 7 | C8H8O3 | Organic acids |
| 51 | Dobutamine | 1 | C18H23NO3 | Alkaloids | 733 | 1-Naphthylacetic acid | 7 | C12H10O2 | Organic acids |
| 52 | N1-Dihydrocaffeoyl-N10-coumaroylspermidine | 1 | C25H33N3O5 | Alkaloids | 734 | 3-Methylbenzaldehyde | 7 | C8H8O | Others |
| 53 | Vanillylamine | 1 | C8H11NO2 | Alkaloids | 735 | 3,5,7,4'-Tetrahydroxy-Coumaronochromone | 7 | C15H10O7 | Others |
| 54 | Dopamine | 1 | C8H11NO2 | Alkaloids | 736 | 3,7-Dihydroxychromen-4-one | 7 | C9H6O4 | Others |
| 55 | N-Feruloylputrescine | 1 | C14H20N2O3 | Alkaloids | 737 | 9-hydroxy-2-(2-hydroxy-3-methoxyphenyl)-3-(hydroxymethyl)-12-methoxy-2,3-dihydro-1,4,5-trioxatetraphen-10-one* | 7 | C24H20O9 | Others |
| 56 | p-Coumaroylputrescine | 1 | C13H18N2O2 | Alkaloids | 738 | Koparin | 7 | C16H12O6 | Others |
| 57 | 4-Hydroxy-3-methoxy-β-phenethylamine | 1 | C9H13NO2 | Alkaloids | 739 | 9-hydroxy-2-(3-hydroxy-4-methoxyphenyl)-3-(hydroxymethyl)-12-methoxy-2,3-dihydro-1,4,5-trioxatetraphen-10-one* | 7 | C24H20O9 | Others |
| 58 | 5-Hydroxyindole-3-acetic acid | 1 | C10H9NO3 | Alkaloids | 740 | Quercetin 3-O-alpha-L-rhamnoside | 7 | C21H20O11 | Others |
| 59 | 3-Indole acetamide | 1 | C10H10N2O | Alkaloids | 741 | Icariside E5* | 7 | C26H34O11 | Others |
| 60 | 1-Methoxy-indole-3-acetamide | 1 | C11H12N2O2 | Alkaloids | 742 | Dihydroactinidiolide | 7 | C11H16O2 | Others |
| 61 | Methoxyindoleacetic acid | 1 | C11H11NO3 | Alkaloids | 743 | Naringenin 7-0-glucoside* | 7 | C21H22O10 | Others |
| 62 | 3-Indoleacrylic acid* | 1 | C11H9NO2 | Alkaloids | 744 | Melilotoside | 7 | C15H18O8 | Others |
| 63 | 3-hydroxy-1-methylpyrrolidin-2-one* | 1 | C5H9NO2 | Alkaloids | 745 | 4-Methylumbelliferyl glucuronide | 7 | C16H16O9 | Others |
| 64 | Pterolactam* | 1 | C5H9NO2 | Alkaloids | 746 | Glucosyl 3,7-dimethyloct-2-ene-1,6,7-triol | 7 | C16H30O8 | Others |
| 65 | 3,5-Dihydro-2H-Furo[3,2-C]Quinolin-4-One* | 1 | C11H9NO2 | Alkaloids | 747 | 3ξ-(1ξ-hydroxyethyl)-7-hydroxy-1-isobenzofuranone | 7 | C10H10O4 | Others |
| 66 | N-Monomethyl-L-arginine* | 1 | C7H16N4O2 | Amino acids and derivatives | 748 | Glucosyl 3,7-dimethylocta-2,6-diene-1,4,8-trio | 7 | C16H28O8 | Others |
| 67 | Arginine methyl ester* | 1 | C7H16N4O2 | Amino acids and derivatives | 749 | 2-(hydroxymethyl)-6-[4-(3-hydroxypropyl)-2,6-dimethoxyphenoxy]oxane-3,4,5-triol | 7 | C17H26O9 | Others |
| 68 | L-Arginine | 1 | C6H14N4O2 | Amino acids and derivatives | 750 | D-Threose | 7 | C4H8O4 | Others |
| 69 | L-Cystathionine | 1 | C7H14N2O4S | Amino acids and derivatives | 751 | Allitol | 7 | C6H14O6 | Others |
| 70 | D-Ornithine | 1 | C5H12N2O2 | Amino acids and derivatives | 752 | D-Sorbitol | 7 | C6H14O6 | Others |
| 71 | N(6),N(6)-Dimethyl-L-lysine | 1 | C8H18N2O2 | Amino acids and derivatives | 753 | D-Mannitol | 7 | C6H14O6 | Others |
| 72 | O-Phospho-L-serine | 1 | C3H8NO6P | Amino acids and derivatives | 754 | Butyl Beta-D-Fructopyranoside | 7 | C10H20O6 | Others |
| 73 | Homoarginine | 1 | C7H16N4O2 | Amino acids and derivatives | 755 | Pyridoxine-5'-O-glucoside | 7 | C14H21NO8 | Others |
| 74 | L-Citrulline | 1 | C6H13N3O3 | Amino acids and derivatives | 756 | Methyl Syringate | 7 | C10H12O5 | Phenolic acids |
| 75 | N-α-Acetyl-L-ornithine | 1 | C7H14N2O3 | Amino acids and derivatives | 757 | 4-O-(3'-O-alpha-D-Glucopyranosyl)caffeoylquinic acid | 7 | C22H28O14 | Phenolic acids |
| 76 | L-Tryptophan | 1 | C11H12N2O2 | Amino acids and derivatives | 758 | 2,4-Dinitrophenol | 7 | C6H4N2O5 | Phenolic acids |
| 77 | His-Val-Ser | 1 | C14H23N5O5 | Amino acids and derivatives | 759 | Ethyl phenylacetate | 7 | C10H12O2 | Phenolic acids |
| 78 | L-γ-Glutamyl-L-leucine | 1 | C11H20N2O5 | Amino acids and derivatives | 760 | 3-O-caffeoylshikimic acid | 7 | C16H16O8 | Phenolic acids |
| 79 | N-Acetyl-L-Arginine | 1 | C8H16N4O3 | Amino acids and derivatives | 761 | Doitungbiphenyl A | 7 | C18H20O4 | Phenolic acids |
| 80 | Asp-Met | 1 | C9H16N2O5S | Amino acids and derivatives | 762 | Calceolarioside A* | 7 | C23H26O11 | Phenolic acids |
| 81 | Val-Abu-OH | 1 | C14H18N2O6 | Amino acids and derivatives | 763 | 4-O-(6'-O-Glucosylcaffeoyl)-4-hydroxybenzoic acid | 7 | C22H22O11 | Phenolic acids |
| 82 | cyclo-(Gly-Phe) | 1 | C11H12N2O2 | Amino acids and derivatives | 764 | 5-O-Caffeoylshikimic acid | 7 | C16H16O8 | Phenolic acids |
| 83 | Gly-Pro-Arg | 1 | C13H24N6O4 | Amino acids and derivatives | 765 | 4-Hydroxyphenyllactic Acid* | 7 | C9H10O4 | Phenolic acids |
| 84 | Epicatechin-(2β→O→7,4β→8)-epiafzelechin-(4α→8)-epicatechin | 1 | C45H36O17 | Flavonoids | 766 | Methyl 2,4-dihydroxyphenylacetate* | 7 | C9H10O4 | Phenolic acids |
| 85 | Tetrahydroxyflavan-(4α-8-epicatechin)* | 1 | C30H24O12 | Flavonoids | 767 | 2,3,4-Trihydroxybutyl 6-O-(E)-caffeoyl-β-D-glucopyranoside | 7 | C19H26O12 | Phenolic acids |
| 86 | Cryptostrobin (8-C-Methyl-5,7-Dihydroxyflavanone) | 1 | C16H14O4 | Flavonoids | 768 | 2-O-P-Coumaroylhydroxycitric Acid | 7 | C15H14O10 | Phenolic acids |
| 87 | Cirsilineol (4',5-Dihydroxy-3',6,7-trimethoxyflavone) | 1 | C18H16O7 | Flavonoids | 769 | Populoside | 7 | C22H24O10 | Phenolic acids |
| 88 | Eriodictyol-8-C-glucoside-4'-O-glucoside | 1 | C27H32O16 | Flavonoids | 770 | Homosyringic Acid 4'-O-Glucoside | 7 | C16H22O10 | Phenolic acids |
| 89 | Bavachin | 1 | C20H20O4 | Flavonoids | 771 | Cis-Coutaric acid | 7 | C13H12O8 | Phenolic acids |
| 90 | 5,7-Dihydroxy-3',4',5'-trimethoxyflavone | 1 | C18H16O7 | Flavonoids | 772 | 3,5-Dihydroxytoluene | 7 | C7H8O2 | Phenolic acids |
| 91 | Tricin-7-O-(2''-O-rhamnosyl)galacturonide | 1 | C29H32O17 | Flavonoids | 773 | 1-O-Eudesmoylquinic acid | 7 | C17H22O10 | Phenolic acids |
| 92 | 5,7,4'-Trihydroxy-3,6,3',5'-Tetramethoxyflavone | 1 | C19H18O9 | Flavonoids | 774 | 3-Nitrophenol | 7 | C6H5NO3 | Phenolic acids |
| 93 | Apigenin-7-O-(2''-glucurosyl)glucuronide | 1 | C27H26O17 | Flavonoids | 775 | davidioside C* | 7 | C15H16O9 | Phenolic acids |
| 94 | 3,7-Di-O-methylquercetin | 1 | C17H14O7 | Flavonoids | 776 | Phloroglucinol; 1,3,5-Benzenetriol | 7 | C6H6O3 | Phenolic acids |
| 95 | Kaempferol-3-O-(2''-apiosyl-4''-glucosyl-6''-malonyl)Glucoside | 1 | C35H40O23 | Flavonoids | 777 | Phenylmethyl β-L-glucopyranoside | 7 | C13H18O6 | Phenolic acids |
| 96 | kaempferol-3-sinapoyldiglucoside | 1 | C38H40O20 | Flavonoids | 778 | Protocatechuic acid 1-O-(Glucosylvanilloyl)* | 7 | C21H22O12 | Phenolic acids |
| 97 | kaempferol-3-methoxycaffeoyldiglucoside | 1 | C37H38O20 | Flavonoids | 779 | Dunalianoside A* | 7 | C21H22O10 | Phenolic acids |
| 98 | Quercetin-3-O-sophorotrioside-7-O-arabinoside | 1 | C38H48O26 | Flavonoids | 780 | Dunalianoside B | 7 | C21H22O11 | Phenolic acids |
| 99 | Epoxybergamottin | 1 | C21H22O5 | Lignans and Coumarins | 781 | Iriflophenone-3-C-glucoside | 7 | C19H20O10 | Phenolic acids |
| 100 | Edgeworin rhamnoside | 1 | C24H20O10 | Lignans and Coumarins | 782 | Specnuezhenide | 7 | C31H42O17 | Phenolic acids |
| 101 | 7-Methoxy-5-Prenyloxycoumarin | 1 | C15H16O4 | Lignans and Coumarins | 783 | Digallic Acid | 7 | C14H10O9 | Phenolic acids |
| 102 | 4-[3-(hydroxymethyl)-7-methoxy-5-(prop-2-en-1-yl)-2,3-dihydro-1-benzofuran-2-yl]-2-methoxyphenol | 1 | C20H22O5 | Lignans and Coumarins | 784 | Dicaffeoylquinic acid-O-glucoside | 7 | C31H34O17 | Phenolic acids |
| 103 | Austrobailignan-5* | 1 | C20H22O4 | Lignans and Coumarins | 785 | 1-O-p-Coumaroyl-β-D-glucose | 7 | C15H18O8 | Phenolic acids |
| 104 | 7',8'-Dihydro-7'-(5'-hydroxy-3'-methoxyphenyl)-3-methoxy-8'-methyl-1-(E)-propenylbenzofuran | 1 | C20H22O4 | Lignans and Coumarins | 786 | 3-O-p-Coumaroylshikimic acid | 7 | C16H16O7 | Phenolic acids |
| 105 | (7S,7'R,8S,8'R)-4,4'-Dihydroxy-3,5,3'-trimethoxy-7,7'-epoxylignan | 1 | C21H26O6 | Lignans and Coumarins | 787 | 6-O-Feruloyl-β-D-glucose | 7 | C16H20O9 | Phenolic acids |
| 106 | Verrucosin | 1 | C20H24O5 | Lignans and Coumarins | 788 | Furanofructosyl-α-D-(3-mustard acyl)glucoside* | 7 | C23H32O15 | Phenolic acids |
| 107 | Chicanin | 1 | C20H22O5 | Lignans and Coumarins | 789 | 3,4,5-Trihydroxy-6-(hydroxymethyl)oxan-2-yl2-hydroxy-5-methylbenzoate | 7 | C14H18O8 | Phenolic acids |
| 108 | Saururinone | 1 | C20H20O5 | Lignans and Coumarins | 790 | 4-Methylcatechol | 7 | C7H8O2 | Phenolic acids |
| 109 | (7R,8R)-7,8-dihydro-7-(3,4-dihydroxyphenyl)-3'-methoxy-8-methyl-1'-(E-propenyl)benzofuran | 1 | C19H20O4 | Lignans and Coumarins | 791 | 2-((5-carboxy-5-(((E)-3-(3-(((E)-3-(3,4-dihydroxyphenyl)acryloyl)oxy)-4-hydroxyphenyl)acryloyl)oxy)-2,3-dihydroxycyclohexyl)oxy)-1,3,4,5-tetrahydroxycyclohexane-1-carboxylic acid | 7 | C32H34O18 | Phenolic acids |
| 110 | saucerneol J | 1 | C20H22O5 | Lignans and Coumarins | 792 | Calceolarioside D* | 7 | C23H26O11 | Phenolic acids |
| 111 | [(2S,3S)-2-(3,4-dimethoxyphenyl)-7-methoxy-5-prop-2-enyl-2,3-dihydro-1-benzofuran-3-yl]methanol | 1 | C21H24O5 | Lignans and Coumarins | 793 | 4-O-(6'-O-Glucosylcaffeoylglucosyl)-4-hydroxybenzyl alcohol | 7 | C28H34O15 | Phenolic acids |
| 112 | Licarin B* | 1 | C20H20O4 | Lignans and Coumarins | 794 | Gallic acid-4-O-(6'''-feruloyl)sophoroside | 7 | C29H34O18 | Phenolic acids |
| 113 | otobain* | 1 | C20H20O4 | Lignans and Coumarins | 795 | 1-Caffeoylquinic acid | 7 | C16H18O9 | Phenolic acids |
| 114 | 4-[4-(4-hydroxy-3-methoxyphenyl)-2,3-dimethylbutyl]-2-methoxyphenol | 1 | C20H26O4 | Lignans and Coumarins | 796 | 1,3-O-Dicaffeoylglycerol | 7 | C21H20O9 | Phenolic acids |
| 115 | wulignan A2 | 1 | C20H22O5 | Lignans and Coumarins | 797 | Rosmarinic acid | 7 | C18H16O8 | Phenolic acids |
| 116 | Hinokinin | 1 | C20H18O6 | Lignans and Coumarins | 798 | Grevilloside C | 7 | C17H24O9 | Phenolic acids |
| 117 | Dehydrodiisoeugenol* | 1 | C20H22O4 | Lignans and Coumarins | 799 | Rosmarinic acid-3'-O-glucoside | 7 | C24H26O13 | Phenolic acids |
| 118 | Machilin A* | 1 | C20H22O4 | Lignans and Coumarins | 800 | 3-O-p-Coumaroylshikimic acid-O-glucoside | 7 | C22H26O12 | Phenolic acids |
| 119 | 5'-Methoxyisolariciresinol-9'-O-xyloside | 1 | C26H34O11 | Lignans and Coumarins | 801 | Benzyl 3,6-dimethoxy-2-{[3,4,5-trihydroxy-6-(hydroxymethyl)oxan-2-yl]oxy}benzoate | 7 | C22H26O10 | Phenolic acids |
| 120 | licarin A* | 1 | C20H22O4 | Lignans and Coumarins | 802 | Hematoxylin | 7 | C16H14O6 | Phenolic acids |
| 121 | 4-(5-(3,4-dimethoxyphenyl)-3,4-dimethyltetrahydrofuran-2-yl)benzene-1,2-diol | 1 | C20H24O5 | Lignans and Coumarins | 803 | Desrhamnosylisoacteoside* | 7 | C23H26O11 | Phenolic acids |
| 122 | Otobaphenol* | 1 | C20H22O4 | Lignans and Coumarins | 804 | 1'-O-(3,4-Dihydroxyphenethyl)-O-caffeoyl-glucoside | 7 | C23H26O11 | Phenolic acids |
| 123 | tetrahydrofuroguaiacin B* | 1 | C20H24O5 | Lignans and Coumarins | 805 | Calceolarioside B* | 7 | C23H26O11 | Phenolic acids |
| 124 | Lariciresinol | 1 | C20H24O6 | Lignans and Coumarins | 806 | Cimicifugic acid C | 7 | C20H18O10 | Phenolic acids |
| 125 | Machilin D* | 1 | C20H24O5 | Lignans and Coumarins | 807 | Feruloylferuloyltartaric acid | 7 | C24H22O12 | Phenolic acids |
| 126 | trans-1,2-dihydrodehydroguaiaretic acid* | 1 | C20H22O4 | Lignans and Coumarins | 808 | 1-O-Vanilloyl-D-Glucose* | 7 | C14H18O9 | Phenolic acids |
| 127 | 2,6-Dimethoxy-4-(7-methoxy-3-methyl-5-prop-1-enyl-2,3-dihydro-1-benzofuran-2-yl)phenol | 1 | C21H24O5 | Lignans and Coumarins | 809 | Procyanidin A6 | 7 | C31H28O12 | Tannins |
| 128 | (+/-)5,6-EET Methyl Ester | 1 | C21H34O3 | Lipids | 810 | 3'-O-Methyl-4-O-(beta-D-xylopyranosyl)ellagic acid* | 7 | C20H16O12 | Tannins |
| 129 | 9(10)-EpOME;(9R,10S)-(12Z)-9,10-Epoxyoctadecenoic acid | 1 | C18H32O3 | Lipids | 811 | Flavogallonic Acid Dilactone | 7 | C21H10O12 | Tannins |
| 130 | 5,6-DiHETrE[(±)5,6-dihydroxy-8Z,11Z,14Z-eicosatrienoic acid] | 1 | C20H34O4 | Lipids | 812 | [(1S,2S,3S,7R,9R,13S,14R,15R,17S)-3,15-diacetyloxy-7-hydroxy-2,6,14-trimethyl-4,11,16-trioxo-10-oxatetracyclo[7.7.1.02,7.013,17]heptadec-5-en-17-yl]methylacetate* | 7 | C26H32O11 | Terpenoids |
| 131 | 9,10,11-Trihydroxy-12-octadecenoic acid | 1 | C18H34O5 | Lipids | 813 | Blumenol A | 7 | C13H20O3 | Terpenoids |
| 132 | Erucic acid | 1 | C22H42O2 | Lipids | 814 | 8-Epiloganic acid | 7 | C16H24O10 | Terpenoids |
| 133 | Hydroxypentadecenoic acid glucoside | 1 | C21H38O8 | Lipids | 815 | Ajugoside | 7 | C17H26O10 | Terpenoids |
| 134 | PA(18:2/0:0) | 1 | C21H39O7P | Lipids | 816 | 6''-O-Acetylharpagide | 7 | C17H26O11 | Terpenoids |
| 135 | LysoPC 18:1(2n isomer) | 1 | C26H52NO7P | Lipids | 817 | 23-Hydroxytoonacilide | 7 | C31H38O11 | Terpenoids |
| 136 | LysoPC 18:4 | 1 | C26H46NO7P | Lipids | 818 | N-Methylcolchamine | 8 | C22H27NO5 | Alkaloids |
| 137 | LysoPE 17:1(2n isomer) | 1 | C22H44NO7P | Lipids | 819 | (3S,3'S)-N,N'-((1R,2R)-5-((3,4,5-trihydroxy-6-methyltetrahydro-2H-pyran-2-yl)oxy)cyclohex-3-ene-1,2-diyl)bis(2-methyl-2,3,4,9-tetrahydro-1H-pyrido[3,4-b]indole-3-carboxamide) | 8 | C38H46N6O7 | Alkaloids |
| 138 | LysoPE 18:1(2n isomer)* | 1 | C23H46NO7P | Lipids | 820 | Caffeine | 8 | C8H10N4O2 | Alkaloids |
| 139 | LysoPE 18:2(2n isomer)* | 1 | C23H44NO7P | Lipids | 821 | (12S)-7,15,16,17-tetramethoxy-3,5-dioxa-11-azapentacyclo[10.7.1.02,6.08,20.014,19]icosa-1,6,8(20),14,16,18-hexaene | 8 | C21H23NO6 | Alkaloids |
| 140 | LysoPE 16:1* | 1 | C21H42NO7P | Lipids | 822 | N-Benzylmethylene isomethylamine | 8 | C8H9N | Alkaloids |
| 141 | LysoPE 16:1(2n isomer)* | 1 | C21H42NO7P | Lipids | 823 | 4,5,6-Trihydroxy-2-cyclohexen-1-ylideneacetonitrile | 8 | C8H9NO3 | Alkaloids |
| 142 | Riboprine | 1 | C15H21N5O4 | Nucleotides and derivatives | 824 | 11-[3,4,5-Trihydroxy-6-(hydroxymethyl)oxan-2-yl]oxy-1,6-diazatetracyclo[7.6.1.05,16.010,15]hexadeca-3,5(16),6,8,10,12,14-heptaen-2-one | 8 | C20H18N2O7 | Alkaloids |
| 143 | Uridine 5'-diphospho-D-glucose | 1 | C15H24N2O17P2 | Nucleotides and derivatives | 825 | N-(4-Aminobutyl)benzamide | 8 | C11H16N2O | Alkaloids |
| 144 | Hypoxanthine | 1 | C5H4N4O | Nucleotides and derivatives | 826 | Caffeoylcholine | 8 | C14H20NO4+ | Alkaloids |
| 145 | 4-Guanidinobutyric acid | 1 | C5H11N3O2 | Organic acids | 827 | Sinapine | 8 | C16H24NO5+ | Alkaloids |
| 146 | Glucosyl 2-Hydroxy-4-Methylpentanoic Acid | 1 | C12H22O8 | Organic acids | 828 | N1,N10-Bis(p-coumaroyl)spermidine | 8 | C25H31N3O4 | Alkaloids |
| 147 | Methyl dihydrojasmonate | 1 | C13H22O3 | Organic acids | 829 | Dioxindole-3-acetyl-3-O-glucoside | 8 | C16H19NO9 | Alkaloids |
| 148 | 4-Ethylbenzaldehyde | 1 | C9H10O | Others | 830 | N-Acetylisatin | 8 | C10H7NO3 | Alkaloids |
| 149 | Isovanillin | 1 | C8H8O3 | Others | 831 | Methyl dioxindole-3-acetate | 8 | C11H11NO4 | Alkaloids |
| 150 | Mesitaldehyde | 1 | C10H12O | Others | 832 | 6-Hydroxynicotinic acid | 8 | C6H5NO3 | Alkaloids |
| 151 | 6-hydroxy-2-[2-(3,4-dimethoxyphenyl)ethyl]chromone | 1 | C19H18O5 | Others | 833 | Cinchonidine | 8 | C19H22N2O | Alkaloids |
| 152 | Deoxysappanone B trimethyl ether | 1 | C19H20O5 | Others | 834 | N-Acetyl-L-threonine | 8 | C6H11NO4 | Amino acids and derivatives |
| 153 | 1-(4-hydroxy-3-methoxyphenyl)-7-phenyl-3,5-diheptanone* | 1 | C20H22O4 | Others | 835 | N-Acetyl-L-glutamic acid | 8 | C7H11NO5 | Amino acids and derivatives |
| 154 | Benzylacetone | 1 | C10H12O | Others | 836 | L-Phenylalanine | 8 | C9H11NO2 | Amino acids and derivatives |
| 155 | 1-(4-hydroxyphenyl)-7-(4-hydroxy-3-methoxyphenyl)-4-ene-3-heptanone | 1 | C20H22O4 | Others | 837 | Cyclo(Pro-Glu) | 8 | C10H14N2O4 | Amino acids and derivatives |
| 156 | Lincomolide B | 1 | C17H26O3 | Others | 838 | L-Alanyl-L-leucine | 8 | C9H18N2O3 | Amino acids and derivatives |
| 157 | 9-Oxodemethylmyricanane B | 1 | C20H20O5 | Others | 839 | L-Prolyl-L-Phenylalanine | 8 | C14H18N2O3 | Amino acids and derivatives |
| 158 | acetophenone | 1 | C8H8O | Others | 840 | Oxiglutatione | 8 | C20H32N6O12S2 | Amino acids and derivatives |
| 159 | Hydroxygeranyl 6-O-xylopyranosyl-glucopyranoside | 1 | C21H36O11 | Others | 841 | N-Acetyl-L-leucine | 8 | C8H15NO3 | Amino acids and derivatives |
| 160 | Butyl 1,3,5-trihydroxy-4-{[3-(4-hydroxy-3-methoxyphenyl)prop-2-enoyl]oxy}cyclohexane-1-carboxylate | 1 | C21H28O9 | Others | 842 | L-Phenylalanyl-L-phenylalanine | 8 | C18H20N2O3 | Amino acids and derivatives |
| 161 | Manninotriose | 1 | C18H32O16 | Others | 843 | Cyclo(D-Val-L-Pro) | 8 | C10H16N2O2 | Amino acids and derivatives |
| 162 | Nystose | 1 | C24H42O21 | Others | 844 | Glutathione reduced form | 8 | C10H17N3O6S | Amino acids and derivatives |
| 163 | Verbascose | 1 | C30H52O26 | Others | 845 | Naringenin chalcone; 2',4,4',6'-Tetrahydroxychalcone* | 8 | C15H12O5 | Flavonoids |
| 164 | Glucan | 1 | C18H32O16 | Others | 846 | Okanin-4'-O-gentiobioside* | 8 | C27H32O16 | Flavonoids |
| 165 | D-Maltotetraose | 1 | C24H42O21 | Others | 847 | Okanin-3',4'-di-O-glucoside* | 8 | C27H32O16 | Flavonoids |
| 166 | Stachyose | 1 | C24H42O21 | Others | 848 | Carthamone* | 8 | C21H20O11 | Flavonoids |
| 167 | Laminaran | 1 | C18H32O16 | Others | 849 | Licochalcone B | 8 | C16H14O5 | Flavonoids |
| 168 | D-Glucosamine | 1 | C6H13NO5 | Others | 850 | Didymin (Isosakuranetin-7-O-rutinoside)* | 8 | C28H34O14 | Flavonoids |
| 169 | D-Panose | 1 | C18H32O16 | Others | 851 | Butin; 7,3',4'-Trihydroxyflavanone* | 8 | C15H12O5 | Flavonoids |
| 170 | Pyridoxine | 1 | C8H11NO3 | Others | 852 | Andrographidine A | 8 | C23H26O10 | Flavonoids |
| 171 | Nicotinic acid (Vitamin B3) | 1 | C6H5NO2 | Others | 853 | Naringenin (5,7,4'-Trihydroxyflavanone)* | 8 | C15H12O5 | Flavonoids |
| 172 | Acitretin | 1 | C21H26O3 | Others | 854 | Naringenin-4',7-dimethyl ether | 8 | C17H16O5 | Flavonoids |
| 173 | 3,4-dihydroxy-allylbenzene4-O-p-D-xylopyranosyI-(1-→6)-β-D-glucopyranoside* | 1 | C20H28O11 | Phenolic acids | 855 | Isosakuranetin (5,7-Dihydroxy-4'-methoxyflavanone) | 8 | C16H14O5 | Flavonoids |
| 174 | 3,4-dihydroxybenzaldehyde-xylose-glucoside* | 1 | C18H24O12 | Phenolic acids | 856 | Poncirin (Isosakuranetin-7-O-neohesperidoside)* | 8 | C28H34O14 | Flavonoids |
| 175 | Manglieside B* | 1 | C20H28O11 | Phenolic acids | 857 | Hesperetin-7-O-glucoside* | 8 | C22H24O11 | Flavonoids |
| 176 | 1,7-bis(4-hydroxy-3-methoxyphenyl)hept-1-ene-3-ol | 1 | C21H26O5 | Phenolic acids | 858 | Phellamurin | 8 | C26H30O11 | Flavonoids |
| 177 | Osmanthuside H[2-(4-Hydroxyphenyl)ethyl-β-D-apiosyl-(1→6)-β-D-glucoside] | 1 | C19H28O11 | Phenolic acids | 859 | Engeletin | 8 | C21H22O10 | Flavonoids |
| 178 | 4-Hydroxybenzoic acid glucosyl xyloside* | 1 | C18H24O12 | Phenolic acids | 860 | Taxifolin-3-O-rhamnoside (Astilbin) | 8 | C21H22O11 | Flavonoids |
| 179 | Vnilloylcaffeoyltartaric acid | 1 | C21H18O12 | Phenolic acids | 861 | Luteolin 7-O-p-coumaroyl rhamnoside* | 8 | C30H26O12 | Flavonoids |
| 180 | (S)-2-Phenyloxirane | 1 | C8H8O | Phenolic acids | 862 | Acerosin | 8 | C18H16O8 | Flavonoids |
| 181 | 4,5-O-Dicaffeoylquinic Acid Methyl Ester | 1 | C26H26O12 | Phenolic acids | 863 | Luteolin 5-O-p-coumaroyl rhamnoside* | 8 | C30H26O12 | Flavonoids |
| 182 | 3,4-dihydroxyallyl benzene-4-O-[α-L-rhamnosyl-(1→6)]-β-D-glucopyranoside | 1 | C21H30O11 | Phenolic acids | 864 | Luteolin-6-C-(5''-glucuronyl)xyloside | 8 | C26H26O16 | Flavonoids |
| 183 | methyl 4-O-galloylchlorogenate | 1 | C24H24O13 | Phenolic acids | 865 | Sudachitin | 8 | C18H16O8 | Flavonoids |
| 184 | 2-Hydroxyphenol-1-O-glucosyl(6→1)rhamnoside | 1 | C18H26O11 | Phenolic acids | 866 | 6-C-MethylKaempferol-3-glucoside* | 8 | C22H22O11 | Flavonoids |
| 185 | 2-[2-(2-Hydroxyphenyl)ethyl]-4,6-dimethoxyphenol | 1 | C16H18O4 | Phenolic acids | 867 | 5,2'-Dihydroxy-7,8-dimethoxyflavone glycoside* | 8 | C23H24O11 | Flavonoids |
| 186 | Protocatechuic Acid Methyl Ester | 1 | C8H8O4 | Phenolic acids | 868 | Yuanhuanin* | 8 | C22H22O11 | Flavonoids |
| 187 | Eugenol | 1 | C10H12O2 | Phenolic acids | 869 | Isovitexin-7-O-glucoside(Saponarin)* | 8 | C27H30O15 | Flavonoids |
| 188 | Isochlorogenic acid A* | 1 | C25H24O12 | Phenolic acids | 870 | Apigenin-8-C-Glucoside (Vitexin)* | 8 | C21H20O10 | Flavonoids |
| 189 | 2-Methoxy-4-ethenylphenol | 1 | C9H10O2 | Phenolic acids | 871 | Isoscutellarein | 8 | C15H10O6 | Flavonoids |
| 190 | 2,5-Dihydroxyphenylacetate ethyl triglucoside | 1 | C28H42O18 | Phenolic acids | 872 | Luteolin-7-O-gentiobioside* | 8 | C27H30O16 | Flavonoids |
| 191 | Procyanidin A2* | 1 | C30H24O12 | Tannins | 873 | Apigenin-6-C-glucoside (Isovitexin)* | 8 | C21H20O10 | Flavonoids |
| 192 | (1R,3E,7E,11S,12R)-Dolabella-3,7-dien-18-ol | 1 | C20H34O | Terpenoids | 874 | Isovitexin-8-O-xyloside | 8 | C26H28O14 | Flavonoids |
| 193 | Santalol | 1 | C15H24O | Terpenoids | 875 | Vitexin-2''-O-glucoside* | 8 | C27H30O15 | Flavonoids |
| 194 | Acetylvalerenolic acid | 1 | C17H24O4 | Terpenoids | 876 | Chrysoeriol-7,4'-di-O-glucoside | 8 | C28H32O16 | Flavonoids |
| 195 | Glutinosone | 1 | C14H20O2 | Terpenoids | 877 | Isosaponarin(Isovitexin-4'-O-glucoside)* | 8 | C27H30O15 | Flavonoids |
| 196 | Dihydroisoalantolactone | 1 | C15H22O2 | Terpenoids | 878 | Luteolin-3'-O-glucoside* | 8 | C21H20O11 | Flavonoids |
| 197 | 9-O-β-D-Glucoside of 3-hydroxy-7,8-didehydro-β-ionol | 1 | C19H30O7 | Terpenoids | 879 | Luteolin-7-O-(6''-malonyl)glucoside* | 8 | C24H22O14 | Flavonoids |
| 198 | Methyl geranate | 1 | C11H18O2 | Terpenoids | 880 | Wogonin-7-O-Glucuronide (Wogonoside) | 8 | C22H20O11 | Flavonoids |
| 199 | 2,3,16,21-Tetrahydroxyolean-12-en-28-oic acid (Platycogenic acid C) | 1 | C30H48O6 | Terpenoids | 881 | Apigenin-6,8-di-C-glucoside-4'-O-glucoside | 8 | C33H40O20 | Flavonoids |
| 200 | Prunasin | 2 | C14H17NO6 | Alkaloids | 882 | Vitexin-2''-O-galactoside* | 8 | C27H30O15 | Flavonoids |
| 201 | O-Phosphorylethanolamine | 2 | C2H8NO4P | Alkaloids | 883 | Apigenin-7-O-Gentiobioside | 8 | C27H30O15 | Flavonoids |
| 202 | N,N-cinnamoylbutanediamine* | 2 | C13H18N2O | Alkaloids | 884 | Apigenin-8-C-Arabinoside | 8 | C20H18O9 | Flavonoids |
| 203 | N-Monocinnamoylputrescine* | 2 | C13H18N2O | Alkaloids | 885 | 4'-O-Glucosylvitexin | 8 | C27H30O15 | Flavonoids |
| 204 | 3β-Isodihydrocadambine | 2 | C27H34N2O10 | Alkaloids | 886 | Luteolin-7,3'-di-O-glucoside* | 8 | C27H30O16 | Flavonoids |
| 205 | 6-Methylnicotinamide | 2 | C7H8N2O | Alkaloids | 887 | Luteolin-5,7-di-O-rutinoside* | 8 | C39H50O24 | Flavonoids |
| 206 | 4,6-Dihydroxyquinoline | 2 | C9H7NO2 | Alkaloids | 888 | Apigenin-7-O-glucoside-4'-O-rutinoside | 8 | C33H40O19 | Flavonoids |
| 207 | L-Cyclopentylglycine | 2 | C7H13NO2 | Amino acids and derivatives | 889 | Apigenin-8-C-glucoside-7-O-(6''-sinapoyl)glucoside | 8 | C38H40O19 | Flavonoids |
| 208 | L-Tyrosine methyl ester | 2 | C10H13NO3 | Amino acids and derivatives | 890 | Apigenin-7-O-rutinoside-4'-O-rutinoside | 8 | C39H50O23 | Flavonoids |
| 209 | N-Acetyl-L-tyrosine | 2 | C11H13NO4 | Amino acids and derivatives | 891 | Hispidulin-8-C-(2''-O-xylosyl)xyloside | 8 | C26H28O14 | Flavonoids |
| 210 | Gallocatechin-(4α->8)-Catechin-(4α->8)-Catechin | 2 | C45H38O19 | Flavonoids | 892 | Chrysoeriol-7-O-(6''-sinapoyl)glucoside | 8 | C33H32O15 | Flavonoids |
| 211 | 8,8'-Methylenebiscatechin | 2 | C31H28O12 | Flavonoids | 893 | Apigenin-6-C-(2''-xylosyl)glucoside | 8 | C26H28O14 | Flavonoids |
| 212 | Choerospondin | 2 | C21H22O10 | Flavonoids | 894 | Isovitexin-2''-O-xyloside | 8 | C26H28O14 | Flavonoids |
| 213 | 5,7,3',4',5'-Pentahydroxydihydroflavone | 2 | C15H12O7 | Flavonoids | 895 | Apigenin-7-O-(6''-malonyl)glucoside | 8 | C24H22O13 | Flavonoids |
| 214 | Ladanetin | 2 | C16H12O6 | Flavonoids | 896 | Chrysoeriol-7-O-gentiobioside | 8 | C28H32O16 | Flavonoids |
| 215 | 5,7,8-Tetrahydroxy-6-methoxyflavone | 2 | C16H12O6 | Flavonoids | 897 | Vitexin-7-O-(6''-feruloyl)glucoside | 8 | C37H38O18 | Flavonoids |
| 216 | Hispidulin-7-O-(6''-O-p-Coumaroyl)Glucoside | 2 | C31H28O13 | Flavonoids | 898 | Chrysoeriol-7-O-(6''-malonyl)glucoside | 8 | C25H24O14 | Flavonoids |
| 217 | Jaceosidin-7-O-Glucoside* | 2 | C23H24O12 | Flavonoids | 899 | Galangin-7-O-glucoside* | 8 | C21H20O10 | Flavonoids |
| 218 | Nepetin (5,7,3',4'-Tetrahydroxy-6-methoxyflavone) | 2 | C16H12O7 | Flavonoids | 900 | Chrysoeriol-7-O-rutinoside-5-O-glucoside | 8 | C34H42O20 | Flavonoids |
| 219 | Luteolin-7-O-(6''-eudesmyl)glucoside | 2 | C31H30O15 | Flavonoids | 901 | Chrysoeriol-6-C-glucoside-4'-O-glucoside | 8 | C28H32O16 | Flavonoids |
| 220 | Typhaneoside | 2 | C34H42O20 | Flavonoids | 902 | Luteolin-4'-O-glucoside* | 8 | C21H20O11 | Flavonoids |
| 221 | Isorhamnetin-3-O-arabinoside | 2 | C21H20O11 | Flavonoids | 903 | Ladanetin-6-O-β-D-glucosid* | 8 | C22H22O11 | Flavonoids |
| 222 | Quercetin-3-O-(6''-O-acetyl)galactoside | 2 | C23H22O13 | Flavonoids | 904 | Scutellarein (5,6,7,4'-Tetrahydroxyflavone) | 8 | C15H10O6 | Flavonoids |
| 223 | Kaempferol-3-O-rhamnosyl(1→2)glucoside | 2 | C27H30O15 | Flavonoids | 905 | Meratin* | 8 | C27H30O17 | Flavonoids |
| 224 | Quercetin-3-O-(2''-O-galloyl)Arabinoside | 2 | C27H22O15 | Flavonoids | 906 | Vitexin-2''-O-rhamnoside | 8 | C27H30O14 | Flavonoids |
| 225 | Limocitrin-3-O-galactoside* | 2 | C23H24O13 | Flavonoids | 907 | Apigenin-8-C-glucoside-7-O-Sophoroside | 8 | C33H40O20 | Flavonoids |
| 226 | Kaempferol-3-(2'',6''-di-O-rhamnosyl)-glucoside | 2 | C33H40O19 | Flavonoids | 908 | Apigenin-7-O-glucoside(Cosmosiin)* | 8 | C21H20O10 | Flavonoids |
| 227 | Isorhamnetin-3-O-Glucoside* | 2 | C22H22O12 | Flavonoids | 909 | Isoluteolin-6,8-di-C-glucoside* | 8 | C27H30O16 | Flavonoids |
| 228 | 6-Methoxyquercetin-3-O-rhamnoside | 2 | C22H22O12 | Flavonoids | 910 | Cirsiliol-8-C-(2''-glucosyl)glucoside | 8 | C29H34O17 | Flavonoids |
| 229 | Kaempferol-3-O-(6''-Rhamnosyl-2''-Glucosyl)Glucoside (Camelliaside A) | 2 | C33H40O20 | Flavonoids | 911 | Hispidulin-7-O-glucoside(Homoplantaginin)* | 8 | C22H22O11 | Flavonoids |
| 230 | Kaempferol-3-O-sophoroside-7-O-rhamnoside* | 2 | C33H40O20 | Flavonoids | 912 | Tricin-4'-O-rutinoside-7-O-rutinoside | 8 | C41H54O25 | Flavonoids |
| 231 | Kaempferol-3-O-neohesperidoside-7-O-glucoside* | 2 | C33H40O20 | Flavonoids | 913 | Chrysoeriol-8-C-glucoside-7-O-(6''-feruloyl)glucoside | 8 | C38H40O19 | Flavonoids |
| 232 | Rhamnetin-3-O-Glucoside* | 2 | C22H22O12 | Flavonoids | 914 | Isovitexin-2''-O-(6'''-p-coumaroyl)glucoside | 8 | C36H36O17 | Flavonoids |
| 233 | Prunetin-4'-O-glucoside(Prunitrin) | 2 | C22H22O10 | Flavonoids | 915 | Chrysoeriol-6-C-glucoside-4'-O-(6''-sinapoyl)glucoside | 8 | C39H42O20 | Flavonoids |
| 234 | 1,3,6,7-tetrahydroxy-2-(3,4,5-trihydroxyoxan-2-yl)xanthen-9-one | 2 | C18H16O10 | Flavonoids | 916 | Apigenin-6-C-(2''-rhamnosyl)glucoside | 8 | C27H30O14 | Flavonoids |
| 235 | Coumarin-3-carboxylic Acid | 2 | C10H6O4 | Lignans and Coumarins | 917 | Lysionotin-C-Xylosyl-glucoside | 8 | C29H34O16 | Flavonoids |
| 236 | Esculetin (6,7-Dihydroxycoumarin) | 2 | C9H6O4 | Lignans and Coumarins | 918 | Diosmetin-7-O-galactoside* | 8 | C22H22O11 | Flavonoids |
| 237 | Scopoletin-7-O-glucoside (Scopolin) | 2 | C16H18O9 | Lignans and Coumarins | 919 | Apigenin-6-C-glucoside-7-O-Sophoroside | 8 | C33H40O20 | Flavonoids |
| 238 | Secoisolariciresinol | 2 | C20H26O6 | Lignans and Coumarins | 920 | Tricin-7-O-neohesperidoside | 8 | C29H34O16 | Flavonoids |
| 239 | 6-((4-(3-hydroxy-2-(4-(3-hydroxypropyl)-2-methylphenoxy)propoxy)-2-methoxyphenoxy)methyl)tetrahydro-2H-pyran-2,3,4,5-tetraol | 2 | C26H36O11 | Lignans and Coumarins | 921 | Kaempferol-3-O-(4''-p-coumaroyl)rhamnoside* | 8 | C30H26O12 | Flavonoids |
| 240 | Lyoniresinol-3α-O-glucoside | 2 | C28H38O13 | Lignans and Coumarins | 922 | Morin | 8 | C15H10O7 | Flavonoids |
| 241 | 9,16-Dihydroxypalmitic acid | 2 | C16H32O4 | Lipids | 923 | Kaempferol-3-O-(2''-p-Coumaroyl)glucoside | 8 | C30H26O13 | Flavonoids |
| 242 | DL-2-hydroxystearic acid | 2 | C18H36O3 | Lipids | 924 | Juglanin* | 8 | C20H18O10 | Flavonoids |
| 243 | 2'-O-Methyladenosine | 2 | C11H15N5O4 | Nucleotides and derivatives | 925 | Kaempferol-7-O-glucoside* | 8 | C21H20O11 | Flavonoids |
| 244 | Allopurinol | 2 | C5H4N4O | Nucleotides and derivatives | 926 | Kaempferol-3-O-(6''-p-Coumaroyl)glucoside (Tiliroside) | 8 | C30H26O13 | Flavonoids |
| 245 | Cis-Aconitic acid | 2 | C6H6O6 | Organic acids | 927 | Fisetin | 8 | C15H10O6 | Flavonoids |
| 246 | DL-3-Phenyllactic acid* | 2 | C9H10O3 | Organic acids | 928 | Kaempferol (3,5,7,4'-Tetrahydroxyflavone) | 8 | C15H10O6 | Flavonoids |
| 247 | Tuberonic acid glucoside* | 2 | C18H28O9 | Organic acids | 929 | Kaempferol-3-O-rhamnoside (Afzelin)(Kaempferin)* | 8 | C21H20O10 | Flavonoids |
| 248 | 3,4-methylenedioxy cinnamyl alcohol | 2 | C10H10O3 | Others | 930 | Kaempferol-7-O-rhamnoside* | 8 | C21H20O10 | Flavonoids |
| 249 | 1,4-Benzodioxin-6-propanol | 2 | C11H12O3 | Others | 931 | Kaempferol-6,8-di-C-glucoside* | 8 | C27H30O16 | Flavonoids |
| 250 | 2,6-Dimethoxybenzaldehyde* | 2 | C9H10O3 | Others | 932 | Kaempferol-3-O-(6''-malonyl)galactoside* | 8 | C24H22O14 | Flavonoids |
| 251 | Capillarisin | 2 | C16H12O7 | Others | 933 | Kaempferol-3-O-(2''-p-Coumaroyl)galactoside* | 8 | C30H26O13 | Flavonoids |
| 252 | Hydroxydihydrobovolide* | 2 | C11H18O3 | Others | 934 | Kaempferol-3-O-(6''-malonyl)glucoside* | 8 | C24H22O14 | Flavonoids |
| 253 | 5-methylfurfural | 2 | C6H6O2 | Others | 935 | Kaempferol-3,7-O-diglucoside* | 8 | C27H30O16 | Flavonoids |
| 254 | cis-coumarinic acid-beta-D-glucoside | 2 | C15H18O8 | Others | 936 | Kaempferol-3-O-arabinoside* | 8 | C20H18O10 | Flavonoids |
| 255 | 5-hydroxy-3,4-dimethyl-5-pentylfuran-2(5H)-one* | 2 | C11H18O3 | Others | 937 | Quercetin-3-O-α-rhamnosyl (1→2)-[α-rhamnosyl (1→6)]-β-glucoside | 8 | C33H40O20 | Flavonoids |
| 256 | Eugenyl formate | 2 | C11H12O3 | Others | 938 | Kaempferol-3-O-robinobioside(Biorobin)* | 8 | C27H30O15 | Flavonoids |
| 257 | (R)-3-ethyl-7-hydroxy-6-methoxyphthalide | 2 | C11H12O4 | Others | 939 | Kaempferol-3-O-(6''-Sinapyl)glucosyl-(1→2)-Galactoside | 8 | C38H40O20 | Flavonoids |
| 258 | L-Fucitol | 2 | C6H14O5 | Others | 940 | Kaempferol-3-O-rutinoside(Nicotiflorin)* | 8 | C27H30O15 | Flavonoids |
| 259 | Rutinose | 2 | C12H22O10 | Others | 941 | Kaempferol-3-O-arabinoside-7-O-rhamnoside | 8 | C26H28O14 | Flavonoids |
| 260 | D-Fructose 6-Phosphate* | 2 | C6H13O9P | Others | 942 | Kaempferol-3-O-galactoside-4'-O-glucoside* | 8 | C27H30O16 | Flavonoids |
| 261 | L-Arabitol | 2 | C5H12O5 | Others | 943 | Kaempferol-3-O-arabinoside (Juglanin) | 8 | C20H18O10 | Flavonoids |
| 262 | D-Glucose-1-phosphate* | 2 | C6H13O9P | Others | 944 | Kaempferol-3-O-(6''-p-Coumaroyl)galactoside* | 8 | C30H26O13 | Flavonoids |
| 263 | Isoascorbic acid 2-O-glucoside | 2 | C12H18O11 | Others | 945 | Isorhamnetin-3-O-rutinoside-7-O-(2''-O-glucosyl)glucuronate | 8 | C40H50O27 | Flavonoids |
| 264 | N-(beta-D-Glucosyl)nicotinate | 2 | C12H15NO7 | Others | 946 | Isorhamnetin-3-O-(6''-acetylglucosyl)(1→3)-glucoside | 8 | C30H34O18 | Flavonoids |
| 265 | Orotic acid (Vitamin B13) | 2 | C5H4N2O4 | Others | 947 | Kaempferol-3-O-galactoside (Trifolin)* | 8 | C21H20O11 | Flavonoids |
| 266 | Methyl caffeate | 2 | C10H10O4 | Phenolic acids | 948 | Kaempferol-3-O-(3''-O-p-Coumaroyl)rhamnoside | 8 | C30H26O12 | Flavonoids |
| 267 | Syringalide A | 2 | C23H26O10 | Phenolic acids | 949 | Limocitrin-3-O-arabinoside | 8 | C22H22O12 | Flavonoids |
| 268 | Caffeic acid | 2 | C9H8O4 | Phenolic acids | 950 | Amoenin | 8 | C21H20O11 | Flavonoids |
| 269 | ((2R,3R,5R,6R)-6-(((E)-3-(3,4-dihydroxyphenyl)acryloyl)oxy)-3,4,5-trihydroxytetrahydro-2H-pyran-2-yl)methyl (Z)-3-(3,4-dihydroxyphenyl)acrylate | 2 | C24H24O12 | Phenolic acids | 951 | Genistein-7-O-Glucoside (Genistin) | 8 | C21H20O10 | Flavonoids |
| 270 | 1-O-(3,4-Dihydroxy-5-methoxy-benzoyl)-glucoside | 2 | C14H18O10 | Phenolic acids | 952 | Genistein-7-O-galactoside* | 8 | C21H20O10 | Flavonoids |
| 271 | 5-O-p-Coumaroylshikimic acid O-glucoside | 2 | C22H26O12 | Phenolic acids | 953 | Sophoricoside* | 8 | C21H20O10 | Flavonoids |
| 272 | Sodium ferulate | 2 | C10H9NaO4 | Phenolic acids | 954 | 5,7-Dihydroxy-4-methylcoumarin | 8 | C10H8O4 | Lignans and Coumarins |
| 273 | gentisic acid 5-O-β-D-(6'-O-galloyl)-gluco-pyranoside | 2 | C20H20O13 | Phenolic acids | 955 | Scopoletin (7-Hydroxy-6-methoxycoumarin) | 8 | C10H8O4 | Lignans and Coumarins |
| 274 | 3,5-Dicaffeoylquinic acid | 2 | C25H24O12 | Phenolic acids | 956 | 6,7-Dihydroxycoumarin-7-O-(6'-acetyl)glucoside | 8 | C16H16O10 | Lignans and Coumarins |
| 275 | 6-O-Caffeoylarbutin | 2 | C21H22O10 | Phenolic acids | 957 | Secoisolariciresinol-9'-O-xyloside | 8 | C25H34O10 | Lignans and Coumarins |
| 276 | galloyl xylosyl glucoside* | 2 | C18H24O14 | Phenolic acids | 958 | 1-Hydroxypinoresinol-1-O-Glucoside* | 8 | C26H32O12 | Lignans and Coumarins |
| 277 | mudanoside B* | 2 | C18H24O14 | Phenolic acids | 959 | Isolariciresinol-9'-O-glucoside* | 8 | C26H34O11 | Lignans and Coumarins |
| 278 | Hydroxytyrosol | 2 | C8H10O3 | Phenolic acids | 960 | Eucommin A | 8 | C27H34O12 | Lignans and Coumarins |
| 279 | 4-O-(6'-O-Glucosylcaffeoyl)-3,4-dihydroxybenzyl alcohol | 2 | C22H24O11 | Phenolic acids | 961 | Nortrachelogenin-4'-O-gentiobioside* | 8 | C32H42O17 | Lignans and Coumarins |
| 280 | 3-(4-Hydroxy-3-methoxyphenyl)-1,2-propanediol | 2 | C10H14O4 | Phenolic acids | 962 | Schisandrin; Schizandrin; Schizandrol A | 8 | C24H32O7 | Lignans and Coumarins |
| 281 | Phenoxyacetic acid | 2 | C8H8O3 | Phenolic acids | 963 | Syringaresinol-4'-O-glucoside; Acanthoside B | 8 | C28H36O13 | Lignans and Coumarins |
| 282 | 1-O-Caffeoylglycerol | 2 | C12H14O6 | Phenolic acids | 964 | Nortrachelogenin-4-O-glucoside* | 8 | C26H32O12 | Lignans and Coumarins |
| 283 | 5-Methoxysalicylic acid | 2 | C8H8O4 | Phenolic acids | 965 | Machilin H | 8 | C21H26O6 | Lignans and Coumarins |
| 284 | 3-Hydroxy-4-isopropylbenzylalcohol-3-O-glucoside | 2 | C16H24O7 | Phenolic acids | 966 | Nortrachelogenin | 8 | C20H22O7 | Lignans and Coumarins |
| 285 | 5'-Glucosyloxyjasmanic acid* | 2 | C18H28O9 | Phenolic acids | 967 | Schizandriside* | 8 | C25H32O10 | Lignans and Coumarins |
| 286 | 3,4'-Dihydroxy-3'-methoxybenzenepentanoic acid | 2 | C12H16O5 | Phenolic acids | 968 | Schisantherin E | 8 | C30H34O9 | Lignans and Coumarins |
| 287 | Curculigine | 2 | C23H28O12 | Phenolic acids | 969 | Isolariciresinol-9'-O-xyloside* | 8 | C25H32O10 | Lignans and Coumarins |
| 288 | 3-(4-Hydroxyphenyl)-1-propanol | 2 | C9H12O2 | Phenolic acids | 970 | 1-Hydroxypineolin Diglucoside* | 8 | C32H42O17 | Lignans and Coumarins |
| 289 | Proanthocyanidins | 2 | C30H26O13 | Tannins | 971 | (2r,3r,4s)-6-hydroxy-4-(4-hydroxy-3-methoxyphenyl)-7-methoxy-2,3-dimethyl-3,4-dihydro-2h-naphthalen-1-one | 8 | C20H22O5 | Lignans and Coumarins |
| 290 | Procyanidin C1 | 2 | C45H38O18 | Tannins | 972 | 7S,8R-threo-3',9,9'-trihydroxy-3-methoxy-4',7-epoxy-neolignan-4-O-rhamnoside | 8 | C25H32O10 | Lignans and Coumarins |
| 291 | Genipin | 2 | C11H14O5 | Terpenoids | 973 | E,E,Z-1,3,12-Nonadecatriene-5,14-diol | 8 | C19H34O2 | Lipids |
| 292 | Nuezhengalaside* | 2 | C18H28O9 | Terpenoids | 974 | 13(S)-HODE;13(S)-Hydroxyoctadeca-9Z,11E-dienoic acid* | 8 | C18H32O3 | Lipids |
| 293 | 6-O-p-Coumaroyl-3-methoxy-7-deoxyrehmaglutin A | 2 | C19H22O7 | Terpenoids | 975 | 9S-Hydroxy-10E,12Z-octadecadienoic acid* | 8 | C18H32O3 | Lipids |
| 294 | 3β,11-Dihydroxy-4,14-oxideenantiocudesmane | 2 | C15H26O3 | Terpenoids | 976 | 15(R)-Hydroxylinoleic Acid | 8 | C18H32O3 | Lipids |
| 295 | 4-[5-(3-Hydroxypropyl)-7-methoxy-3-methyl-2,3-dihydro-1-benzofuran-2-yl]-2-methoxyphenol | 2 | C20H24O5 | Terpenoids | 977 | 4-Oxo-9Z,11Z,13E,15E-Octadecatetraenoic Acid | 8 | C18H26O3 | Lipids |
| 296 | Betaine | 3 | C5H11NO2 | Alkaloids | 978 | 10-Undecenoic acid | 8 | C11H20O2 | Lipids |
| 297 | γ-Sanshool | 3 | C18H27NO | Alkaloids | 979 | Palmitaldehyde | 8 | C16H32O | Lipids |
| 298 | N-(gamma-L-glutamyl)tyramine O-glucoside | 3 | C19H28N2O9 | Alkaloids | 980 | Monogalactosyldiacylglycerol | 8 | C45H70O10 | Lipids |
| 299 | p-Coumaroylcadaverine | 3 | C14H20N2O2 | Alkaloids | 981 | 9,10-DHOME; (12Z)-9,10-Dihydroxyoctadec-12-enoic acid | 8 | C18H34O4 | Lipids |
| 300 | L-Methionine Sulfoxide | 3 | C5H11NO3S | Amino acids and derivatives | 982 | LysoPG 16:1 | 8 | C22H43O9P | Lipids |
| 301 | N-(acetyl)phenylalanine | 3 | C11H13NO3 | Amino acids and derivatives | 983 | Monolinolenin* | 8 | C21H36O4 | Lipids |
| 302 | Allysine(6-Oxo DL-Norleucine) | 3 | C6H11NO3 | Amino acids and derivatives | 984 | Glycerol 9(E),11(Z),13(E)-octadecatrienoyl ester* | 8 | C21H36O4 | Lipids |
| 303 | 3-Hydroxyphloretin-4'-O-(2''-O-galloyl)glucoside | 3 | C28H28O15 | Flavonoids | 985 | 2-α-Linolenoyl-glycerol* | 8 | C21H36O4 | Lipids |
| 304 | Phloretin-2'-O-glucoside (Phlorizin) | 3 | C21H24O10 | Flavonoids | 986 | 1-α-Linolenoyl-glycerol* | 8 | C21H36O4 | Lipids |
| 305 | 2,4,2',5'-Tetrahydroxydihydrochalcone | 3 | C15H14O5 | Flavonoids | 987 | LysoPC 18:3(2n isomer)* | 8 | C26H48NO7P | Lipids |
| 306 | Gallocatechin | 3 | C15H14O7 | Flavonoids | 988 | LysoPC 19:2* | 8 | C27H52NO7P | Lipids |
| 307 | catechin-4-β-D-galactopyranoside* | 3 | C21H24O11 | Flavonoids | 989 | LysoPC 18:3* | 8 | C26H48NO7P | Lipids |
| 308 | Epicatechin-3'-O-β-D-glucopyranoside* | 3 | C21H24O11 | Flavonoids | 990 | LysoPC 19:2(2n isomer)* | 8 | C27H52NO7P | Lipids |
| 309 | Epicatechin-4'-O-β-D-glucopyranoside* | 3 | C21H24O11 | Flavonoids | 991 | LysoPC(18:3(9Z,12Z,15Z)) | 8 | C26H48NO7P | Lipids |
| 310 | Isookanin | 3 | C15H12O6 | Flavonoids | 992 | 1-(2,3-dihydroxypropoxy)-3-(((2-(dimethylamino)ethoxy)(hydroxy)phosphoryl)oxy)propan-2-yl (8E,11Z,14Z)-octadeca-8,11,14-trienoate* | 8 | C28H52NO9P | Lipids |
| 311 | Hesperetin-7-O-rutinoside (Hesperidin)* | 3 | C28H34O15 | Flavonoids | 993 | 2-(2,3-dihydroxypropoxy)-3-(((2-(dimethylamino)ethoxy)(hydroxy)phosphoryl)oxy)propyl (8E,11Z,14Z)-octadeca-8,11,14-trienoate* | 8 | C28H52NO9P | Lipids |
| 312 | Hesperetin-7-O-neohesperidoside(Neohesperidin)* | 3 | C28H34O15 | Flavonoids | 994 | LysoPE 18:3(2n isomer)* | 8 | C23H42NO7P | Lipids |
| 313 | Methylhesperidin | 3 | C29H36O15 | Flavonoids | 995 | 4-methyl-1,5,2,3-dioxadiazinan-2-amine | 8 | C3H9N3O2 | Nucleotides and derivatives |
| 314 | Hypolaetin* | 3 | C15H10O7 | Flavonoids | 996 | Inosine 5'-monophosphate | 8 | C10H13N4O8P | Nucleotides and derivatives |
| 315 | Isoetin (5,7,2',4',5'-Pentahydroxyflavone)* | 3 | C15H10O7 | Flavonoids | 997 | Adenosine 5'-monophosphate | 8 | C10H14N5O7P | Nucleotides and derivatives |
| 316 | Chrysin-7-O-glucoside | 3 | C21H20O9 | Flavonoids | 998 | Ribosyladenosine | 8 | C15H21N5O8 | Nucleotides and derivatives |
| 317 | Leucocyanidin | 3 | C15H14O7 | Flavonoids | 999 | Guanine | 8 | C5H5N5O | Nucleotides and derivatives |
| 318 | ageconyflavone C | 3 | C20H20O8 | Flavonoids | 1000 | Barbituric acid;Malonylurea;2,4,6-Pyrimidinetrione | 8 | C4H4N2O3 | Nucleotides and derivatives |
| 319 | Chrysoeriol-7-O-(6''-acetyl)glucoside* | 3 | C24H24O12 | Flavonoids | 1001 | 1-Methyladenine | 8 | C6H7N5 | Nucleotides and derivatives |
| 320 | 3'-O-Methyltricetin-5-O-glucoside* | 3 | C22H22O12 | Flavonoids | 1002 | Flavin Single Nucleotide(FMN) | 8 | C17H21N4O9P | Nucleotides and derivatives |
| 321 | Diosmetin-7-O-Neohesperidoside (Neodiosmin)* | 3 | C28H32O15 | Flavonoids | 1003 | Succinyladenosine | 8 | C14H17N5O8 | Nucleotides and derivatives |
| 322 | Diosmetin-8-C-(2''-O-rhamnosyl)glucoside | 3 | C28H32O15 | Flavonoids | 1004 | β-Pseudouridine | 8 | C9H12N2O6 | Nucleotides and derivatives |
| 323 | Pectolinarigenin-7-O-glucoside | 3 | C23H24O11 | Flavonoids | 1005 | 5-Aminoimidazole ribonucleotide | 8 | C8H14N3O7P | Nucleotides and derivatives |
| 324 | Diosmetin-7-O-rutinoside (Diosmin)* | 3 | C28H32O15 | Flavonoids | 1006 | α-Ketoglutaric acid | 8 | C5H6O5 | Organic acids |
| 325 | Rehderianin I* | 3 | C17H14O7 | Flavonoids | 1007 | Methanesulfonic acid | 8 | CH4O3S | Organic acids |
| 326 | Myricetin | 3 | C15H10O8 | Flavonoids | 1008 | 3-Guanidinopropionic acid | 8 | C4H9N3O2 | Organic acids |
| 327 | Isorhamnetin-3-O-sophoroside | 3 | C28H32O17 | Flavonoids | 1009 | Glucosyl 2,3-Dihydroxy-2-Methylbutanoic Acid | 8 | C11H20O9 | Organic acids |
| 328 | Kaempferide-3-O-（6'-O-acetyl）glucoside* | 3 | C24H24O12 | Flavonoids | 1010 | 3-(Beta-D-Glucopyranosyloxy)-5-Hydroxyhexanoic Acid Methyl Ester | 8 | C13H24O9 | Organic acids |
| 329 | 8-Methoxykaempferol-7-O-rhamnoside* | 3 | C22H22O11 | Flavonoids | 1011 | 4,8-Dihydroxyquinoline-2-carboxylic acid | 8 | C10H7NO4 | Organic acids |
| 330 | Quercetin-3-O-(2''-O-acetyl)glucuronide | 3 | C23H20O14 | Flavonoids | 1012 | 2-Isopropylmalic Acid | 8 | C7H12O5 | Organic acids |
| 331 | Complanatuside | 3 | C28H32O16 | Flavonoids | 1013 | 2-Propylmalic Acid | 8 | C7H12O5 | Organic acids |
| 332 | Malonyl Amurensin | 3 | C29H30O14 | Flavonoids | 1014 | 4-Hydroxy-2-oxoglutaric acid | 8 | C5H6O6 | Organic acids |
| 333 | Kaempferol-3-O-(2''-O-xylosyl-6''-O-rhamnosyl)glucoside | 3 | C32H38O19 | Flavonoids | 1015 | 6-Acetamidohexanoic acid | 8 | C8H15NO3 | Organic acids |
| 334 | Kaempferol-3-O-(2''-galloyl)galactoside* | 3 | C28H24O15 | Flavonoids | 1016 | 5,7-Dihydroxychromone glucoside | 8 | C15H16O9 | Others |
| 335 | Quercetin-3-O-sambubioside | 3 | C26H28O16 | Flavonoids | 1017 | 3,5-Dihydroxy-2-(4-hydroxyphenyl)-7-[(3,4,5-trihydroxy-6-methyltetrahydro-2H-pyran-2-yl)oxy]-4H-1-benzopyran-4-one* | 8 | C21H20O10 | Others |
| 336 | Tamarixetin-3-O-glucoside (Tamarixin)* | 3 | C22H22O12 | Flavonoids | 1018 | 1-18:3-LysoPC | 8 | C26H48NO7P | Others |
| 337 | Galloylisorhamnetin | 3 | C23H16O11 | Flavonoids | 1019 | 2,6-Dimethoxy-4-hydroxyphenol-1-O-ß-D-glucopyranoside | 8 | C14H20O9 | Others |
| 338 | Isorhamnetin-3-O-(6''-malonyl)glucoside* | 3 | C25H24O15 | Flavonoids | 1020 | epiloliolide | 8 | C11H16O3 | Others |
| 339 | Myricetin-3-O-galactoside-3'-O-rhamnoside | 3 | C27H30O17 | Flavonoids | 1021 | 7S-O-methyl morroniside | 8 | C18H28O11 | Others |
| 340 | Quercetagetin; 3,3',4',5,6,7-Hexahydroxyflavone | 3 | C15H10O8 | Flavonoids | 1022 | 1-(4'-Hydroxy-3'-methoxyphenyl)-2-[4''-(3-hydroxypropyl)-2'',6''-dimethoxyphenyl]-propane-1,3-Diol | 8 | C21H28O8 | Others |
| 341 | Quercetin-5,4'-di-O-glucoside | 3 | C27H30O17 | Flavonoids | 1023 | D-Glucurono-6,3-lactone | 8 | C6H8O6 | Others |
| 342 | Quercetin-3-O-(2''-O-rhamnosyl)galactoside | 3 | C27H30O16 | Flavonoids | 1024 | Digalactosylglycerol | 8 | C15H28O13 | Others |
| 343 | Fraxetin-8-O-glucoside (Fraxin) | 3 | C16H18O10 | Lignans and Coumarins | 1025 | D-Sedoheptuiose 7-phosphate | 8 | C7H15O10P | Others |
| 344 | 4-Hydroxycoumarin di-glucoside | 3 | C21H26O13 | Lignans and Coumarins | 1026 | Ribulose-5-phosphate | 8 | C5H11O8P | Others |
| 345 | 8-Hydroxycoumarin | 3 | C9H6O3 | Lignans and Coumarins | 1027 | Glucaric acid-1-Phosphate | 8 | C6H11PO11 | Others |
| 346 | Isolariciresinol-9-O-xyloside | 3 | C25H32O10 | Lignans and Coumarins | 1028 | Trehalose 6-phosphate | 8 | C12H23O14P | Others |
| 347 | Pentadecanoic Acid* | 3 | C15H30O2 | Lipids | 1029 | Glucopyranose 6-Hydroxydecanoate | 8 | C16H30O8 | Others |
| 348 | 12(13)Ep-9-KODE | 3 | C18H30O4 | Lipids | 1030 | 1-O-Acetyl-Glucopyranose 6-Hydroxydecanoate | 8 | C18H32O9 | Others |
| 349 | 11,14,17-Eicosatrienoic acid | 3 | C20H34O2 | Lipids | 1031 | 4-Pyridoxic acid | 8 | C8H9NO4 | Others |
| 350 | 3-Methylxanthine | 3 | C6H6N4O2 | Nucleotides and derivatives | 1032 | 4-Pyridoxic acid-O-glucoside | 8 | C14H19NO9 | Others |
| 351 | N6-(2-Hydroxyethyl)adenosine* | 3 | C12H17N5O5 | Nucleotides and derivatives | 1033 | Riboflavin (Vitamin B2) | 8 | C17H20N4O6 | Others |
| 352 | 5-Methyl-2'-Deoxycytidine | 3 | C10H15N3O4 | Nucleotides and derivatives | 1034 | Syringaldehyde-4-O-glucoside* | 8 | C15H20O9 | Phenolic acids |
| 353 | 7-Methylxanthine | 3 | C6H6N4O2 | Nucleotides and derivatives | 1035 | 1-O-Galloyl-β-D-glucose* | 8 | C13H16O10 | Phenolic acids |
| 354 | 1,7-Dimethylxanthine | 3 | C7H8N4O2 | Nucleotides and derivatives | 1036 | p-Hydroxypheny-β-D-allopyranoside* | 8 | C12H16O7 | Phenolic acids |
| 355 | 1-Methylxanthine | 3 | C6H6N4O2 | Nucleotides and derivatives | 1037 | 2-Nitrophenol | 8 | C6H5NO3 | Phenolic acids |
| 356 | Piperonylic acid | 3 | C8H6O4 | Organic acids | 1038 | Disinapoyl glucoside | 8 | C28H32O14 | Phenolic acids |
| 357 | Oxalic acid | 3 | C2H2O4 | Organic acids | 1039 | Vanilloloside | 8 | C14H20O8 | Phenolic acids |
| 358 | 2-(4-Aminobutanamido)-3-(1-methyl-1H-imidazol-5-yl)propanoic acid | 3 | C10H16N4O3 | Organic acids | 1040 | 1-O-Caffeoyl-β-D-glucose | 8 | C15H18O9 | Phenolic acids |
| 359 | Abscisic acid | 3 | C15H20O4 | Organic acids | 1041 | 4,6-(S)-Hexahydroxydiphenoyl-β-D-glucose | 8 | C20H18O14 | Phenolic acids |
| 360 | L-Lactic Acid | 3 | C3H6O3 | Organic acids | 1042 | 1-O-Gentisoyl-β-D-glucoside | 8 | C13H16O9 | Phenolic acids |
| 361 | 3-(2,5-dimethoxyphenyl)propanoic acid | 3 | C11H14O4 | Organic acids | 1043 | Homogentisic acid* | 8 | C8H8O4 | Phenolic acids |
| 362 | 2,3-Dimethylmaleic anhydride | 3 | C6H6O3 | Organic acids | 1044 | 1-O-(3,4,5-Trimethoxybenzoyl)-B-D-Glucopyranoside | 8 | C16H22O10 | Phenolic acids |
| 363 | 3,4,5-Trihydroxy-6-[5-hydroxy-2-(4-hydroxyphenyl)-4-oxochromen-7-yl]oxyoxane-2-carboxylic acid | 3 | C21H18O11 | Organic acids | 1045 | Mucic acid-1,4-lactone-3,5-di-O-gallate | 8 | C20H16O15 | Phenolic acids |
| 364 | 2-Methylglutaric acid | 3 | C6H10O4 | Organic acids | 1046 | Caffeoyl-O-mannitol | 8 | C15H20O9 | Phenolic acids |
| 365 | Argininosuccinic acid | 3 | C10H18N4O6 | Organic acids | 1047 | 3,4-Dihydroxybenzeneacetic acid* | 8 | C8H8O4 | Phenolic acids |
| 366 | 5-Hydroxymethylfurfural | 3 | C6H6O3 | Others | 1048 | Protocatechuic acid 4-O-(6''-O-p-Coumaroyl)Glucoside | 8 | C22H22O11 | Phenolic acids |
| 367 | Protocatechualdehyde | 3 | C7H6O3 | Others | 1049 | Arbutin* | 8 | C12H16O7 | Phenolic acids |
| 368 | α-Cyperone | 3 | C15H22O | Others | 1050 | Dihydrocaffeoylglucose* | 8 | C15H20O9 | Phenolic acids |
| 369 | Aquilegiolide | 3 | C8H8O3 | Others | 1051 | 1-O-(6'-O-feruloyl)glucoside-3-O-Caffeoyl Quinic Acid | 8 | C32H36O17 | Phenolic acids |
| 370 | 3-Ethyl-7-hydroxyphthalide | 3 | C10H10O3 | Others | 1052 | Quinacyl syringic acid | 8 | C16H20O10 | Phenolic acids |
| 371 | D-Xylonic acid | 3 | C5H10O6 | Others | 1053 | 5-(2-Hydroxyethyl)-2-O-glucosylphenol | 8 | C14H20O8 | Phenolic acids |
| 372 | D-Galacturonic acid* | 3 | C6H10O7 | Others | 1054 | 4-O-(6'-O-Glucosylcaffeoyl)-3,4-dihydroxybenzoic acid | 8 | C22H22O12 | Phenolic acids |
| 373 | (2r,3s,4s,5r)-2,5-bis(hydroxymethyl)-2-methoxyoxolane-3,4-diol | 3 | C7H14O6 | Others | 1055 | 5-Galloyl-6-O-Benzoyl Glucose | 8 | C20H20O11 | Phenolic acids |
| 374 | L-Fucose | 3 | C6H12O5 | Others | 1056 | Robustaside A;[6'-p-Coumarylarbutin] | 8 | C21H22O9 | Phenolic acids |
| 375 | D-Glucoronic acid* | 3 | C6H10O7 | Others | 1057 | 3,4'-Dihydroxypropiophenone glucoside* | 8 | C15H20O8 | Phenolic acids |
| 376 | D-Glucono-1,5-lactone | 3 | C6H10O6 | Others | 1058 | (2E)-3-[4-(β-D-glucopyranoside)-phenylacrylic]-acid | 8 | C15H18O8 | Phenolic acids |
| 377 | Gluconic acid | 3 | C6H12O7 | Others | 1059 | 4-O-Glucosyl-sinapate | 8 | C17H22O10 | Phenolic acids |
| 378 | DMelezitose O-rhamnoside | 3 | C24H42O20 | Others | 1060 | 1-(4-Methoxyphenyl)-1-propanol | 8 | C10H14O2 | Phenolic acids |
| 379 | Nicotinate D-ribonucleoside | 3 | C11H14NO6+ | Others | 1061 | 3-Prenyl-4-O-glucosyloxy-4-hydroxybenzoic acid | 8 | C18H24O8 | Phenolic acids |
| 380 | 6-Galloylglucosyl Aascorbic acid | 3 | C19H22O15 | Others | 1062 | 6-O-Caffeoyl-D-glucose | 8 | C15H18O9 | Phenolic acids |
| 381 | Tachioside* | 3 | C13H18O8 | Phenolic acids | 1063 | Feruloylcaffeoyltartaric acid | 8 | C23H20O12 | Phenolic acids |
| 382 | Isotachioside* | 3 | C13H18O8 | Phenolic acids | 1064 | 2-Feruloyl-sn-glycerol* | 8 | C13H16O6 | Phenolic acids |
| 383 | 2,3-Dihydroxy-1-(4-hydroxy-3,5-dimethoxyphenyl)propan-1-one | 3 | C11H14O6 | Phenolic acids | 1065 | 3,4,5-Trimethoxycinnamic acid | 8 | C12H14O5 | Phenolic acids |
| 384 | 10-acetylmonomelittoside* | 3 | C17H24O11 | Phenolic acids | 1066 | 1-O-Cinnamoyl-4,6-(S)-HHDP-β-D-glucose* | 8 | C29H24O15 | Phenolic acids |
| 385 | 2-(3,4-dihydroxyphenyl)ethanediol 1-O-β-D-glucopyranoside* | 3 | C14H20O9 | Phenolic acids | 1067 | 2'-O-β-D-Glucopyranosylsalicin | 8 | C19H28O12 | Phenolic acids |
| 386 | 3,4,5-trihydroxy-5-[4-(prop-2-enoyloxymethyl)phenoxy]oxane-2-carboxylic acid | 3 | C16H18O9 | Phenolic acids | 1068 | 1-O-Caffeoyl-(6-O-glucosyl)-β-D-glucose | 8 | C21H28O14 | Phenolic acids |
| 387 | 4-O-Galloyl-1-O-Cinnamoyl-β-D-glucose* | 3 | C22H22O11 | Phenolic acids | 1069 | Sanguisorbic acid dilactone | 8 | C21H10O13 | Phenolic acids |
| 388 | Koaburaside* | 3 | C14H20O9 | Phenolic acids | 1070 | 2-Caffeoyl-L-tartaric acid (Caftaric acid) | 8 | C13H12O9 | Phenolic acids |
| 389 | Gentisic acid 2-O-(6''-O-p-coumaroyl)Glucoside* | 3 | C22H22O11 | Phenolic acids | 1071 | Phloroglucinol-1-O-β-D-glucopyranoside | 8 | C12H16O8 | Phenolic acids |
| 390 | 1-O-Galloyl-2-O-Cinnamoyl-β-D-glucose* | 3 | C22H22O11 | Phenolic acids | 1072 | 6-O-Glucosyl-caffeoylbenzoic acid | 8 | C22H22O10 | Phenolic acids |
| 391 | Methyl 2-O-(4-hydroxybenzoyl)-2,4,6-trihydroxyphenylacetate | 3 | C16H14O7 | Phenolic acids | 1073 | Trigallic acid | 8 | C21H14O13 | Phenolic acids |
| 392 | 1-O-Galloyl-3-O-Feruloyl-β-D-glucose* | 3 | C23H24O13 | Phenolic acids | 1074 | 4-Aminobenzoic acid | 8 | C7H7NO2 | Phenolic acids |
| 393 | 2-O-Galloyl-D-glucose* | 3 | C13H16O10 | Phenolic acids | 1075 | 1-(2,4,5-Trimethoxyphenyl)-1,2-propanedione | 8 | C12H14O5 | Phenolic acids |
| 394 | 2,6-dimethoxy-hydroquinone-4-O-β-D-glucopyranoside | 3 | C14H20O9 | Phenolic acids | 1076 | Isochlorogenic acid B* | 8 | C25H24O12 | Phenolic acids |
| 395 | Salvianolic acid N | 3 | C26H22O10 | Phenolic acids | 1077 | 4-Methylphenol | 8 | C7H8O | Phenolic acids |
| 396 | 2',6'-Dihydroxyacetophenone | 3 | C8H8O3 | Phenolic acids | 1078 | Caffeoylbenzoyltartaric acid | 8 | C20H16O10 | Phenolic acids |
| 397 | 3-O-Galloyl-D-glucose* | 3 | C13H16O10 | Phenolic acids | 1079 | 4-O-(6'-O-Glucosylcaffeoylglucosylferuloyl)-4-hydroxybenzyl alcohol | 8 | C38H42O18 | Phenolic acids |
| 398 | 6-(5-Carboxy-2,3-Dihydroxyphenoxy)-3,4,5-Trihydroxyoxane-2-Carboxylic Acid | 3 | C13H14O11 | Phenolic acids | 1080 | Dihydroferulic acid glucoside | 8 | C16H22O9 | Phenolic acids |
| 399 | P-Methoyxcinnamate glucoside | 3 | C16H20O8 | Phenolic acids | 1081 | 3'-O-Beta-D-Glucopyranosyl plumbagic acid | 8 | C17H22O10 | Phenolic acids |
| 400 | Protocatechuic acid glucosyl xyloside | 3 | C18H24O13 | Phenolic acids | 1082 | 3-(3,4,5-Trimethoxyphenyl)propan-1-ol | 8 | C12H18O4 | Phenolic acids |
| 401 | 6-O-Galloyl-β-D-glucose* | 3 | C13H16O10 | Phenolic acids | 1083 | 3-O-Methylellagic acid-4'-O-(5''-O-acetyl)arabinoside | 8 | C22H18O13 | Tannins |
| 402 | 1-O-Galloyl-6-O-Cinnamoyl-β-D-glucose* | 3 | C22H22O11 | Phenolic acids | 1084 | Bartsioside | 8 | C15H22O8 | Terpenoids |
| 403 | 2-O-Caffeoylmalic acid | 3 | C13H12O8 | Phenolic acids | 1085 | Gardenoside | 8 | C17H24O11 | Terpenoids |
| 404 | 3,5-bis(2-hydroxy-3-methylbut-3-en-1-yl)-4-((3,4,5-trihydroxy-6-(hydroxymethyl)tetrahydro-2H-pyran-2-yl)oxy)benzoic acid | 3 | C23H32O10 | Phenolic acids | 1086 | Frehmaglutoside G | 8 | C21H36O9 | Terpenoids |
| 405 | 4-Hydroxybenzoylmalic acid | 3 | C11H10O7 | Phenolic acids | 1087 | 10-Deoxygeniposidic acid | 8 | C16H22O9 | Terpenoids |
| 406 | 5-O-Galloyl-D-hamamelose* | 3 | C13H16O10 | Phenolic acids | 1088 | Spathulenol | 8 | C15H24O | Terpenoids |
| 407 | 1,4-Di-O-galloyl-2-O-p-coumaroyl-β-D-glucose* | 3 | C29H26O16 | Phenolic acids | 1089 | 10-Dehydrogeniposide | 8 | C17H22O10 | Terpenoids |
| 408 | 3'-Gallic acyl sucrose | 3 | C19H26O14 | Phenolic acids | 1090 | 3-Oxolup-20(29)-en-28-oic acid (Betulonic acid) | 8 | C30H46O3 | Terpenoids |
| 409 | Maplexin C (2,3-Di-O-Galloyl-1,5-Anhydro-D-Glucitol)* | 3 | C20H20O13 | Phenolic acids | 1091 | 3,19-Epoxy-3,22-dihydroxydammara-20,24-dien-26-oic acid δ-lactone (Semialactone) | 8 | C30H44O4 | Terpenoids |
| 410 | 2,4,6-Trihydroxybenzoic acid | 3 | C7H6O5 | Phenolic acids | 1092 | 3-Oxours-12-en-28-oic acid (Ursonic acid) | 8 | C30H46O3 | Terpenoids |
| 411 | 1,3-O-Dicaffeoylquinic Acid (Cynarin) | 3 | C25H24O12 | Phenolic acids | 1093 | 2-((3,16-dihydroxy-17-(1-hydroxyethyl)-10,13-dimethyl-2,3,4,5,8,9,10,11,12,13,14,15,16,17-tetradecahydro-1H-cyclopenta[a]phenanthren-2-yl)oxy)-6-(((3,4,5-trihydroxy-6-methyltetrahydro-2H-pyran-2-yl)oxy)methyl)tetrahydro-2H-pyran-3,4,5-triol | 8 | C33H54O13 | Terpenoids |
| 412 | 6'-O-Galloylsalicin | 3 | C20H22O11 | Phenolic acids | 1094 | 4-Hydroxypipecolic acid* | 9 | C6H11NO3 | Alkaloids |
| 413 | 1,6-Di-O-galloyl-2-O-p-coumaroyl-β-D-glucose* | 3 | C29H26O16 | Phenolic acids | 1095 | 6-[(7-hydroxy-2-methyl-6-oxo-3,4-dihydroisoquinolin-1-yl)methyl]-2,3-dimethoxybenzoic acid | 9 | C20H21NO6 | Alkaloids |
| 414 | Tetragallic Acid(3''-O-galloyl-4''-O-galloyl-4-O-galloyl-galloyl acid) | 3 | C28H18O17 | Phenolic acids | 1096 | p-Coumaroylagmatine | 9 | C14H20N4O2 | Alkaloids |
| 415 | 4,6-dimethoxy-2-methoxyphenyl-1-O-beta-D-glucopyranoside | 3 | C13H18O9 | Phenolic acids | 1097 | N,N'-Diferuloylputrescine | 9 | C24H28N2O6 | Alkaloids |
| 416 | 3-Hydroxy-5-Methylphenol-1-O-(6'-Galloyl)Glucoside | 3 | C20H22O11 | Phenolic acids | 1098 | S-Ribosyl-L-homocysteine | 9 | C9H17NO6S | Amino acids and derivatives |
| 417 | Benzoylmalic acid | 3 | C11H10O6 | Phenolic acids | 1099 | S-(Methyl)glutathione | 9 | C11H19N3O6S | Amino acids and derivatives |
| 418 | Gallic acid | 3 | C7H6O5 | Phenolic acids | 1100 | N-Methyl-Trans-4-Hydroxy-L-Proline* | 9 | C6H11NO3 | Amino acids and derivatives |
| 419 | p-Coumaric acid | 3 | C9H8O3 | Phenolic acids | 1101 | L-Cystine | 9 | C6H12N2O4S2 | Amino acids and derivatives |
| 420 | Maplexin D (2,4-Di-O-Galloyl-1,5-Anhydro-D-Glucitol)* | 3 | C20H20O13 | Phenolic acids | 1102 | Teupolioside | 9 | C35H46O20 | Amino acids and derivatives |
| 421 | 3,5-Dihydroxyphenyl1-O-(6-O-Galloyl-β-D-Glucopyranoside) | 3 | C19H20O12 | Phenolic acids | 1103 | Phloretin | 9 | C15H14O5 | Flavonoids |
| 422 | 2-Phenylethy-1-O-β-D-glucoside | 3 | C14H20O6 | Phenolic acids | 1104 | Okanin-4'-(6''-O-acetyl)glucoside* | 9 | C23H24O13 | Flavonoids |
| 423 | 5-Glucosyloxy-2-Hydroxybenzoic acid methyl ester* | 3 | C14H18O9 | Phenolic acids | 1105 | Farrerol-7-O-glucoside | 9 | C23H26O10 | Flavonoids |
| 424 | Scroside D* | 3 | C20H30O13 | Phenolic acids | 1106 | Butin-7-O-glucoside* | 9 | C21H22O10 | Flavonoids |
| 425 | Vanillyl alcohol | 3 | C8H10O3 | Phenolic acids | 1107 | Bavachinin | 9 | C21H22O4 | Flavonoids |
| 426 | Methyl 3-(3-hydroxy-4-methoxyphenyl)propanoate | 3 | C11H14O4 | Phenolic acids | 1108 | 6-C-Glucosyl-2-Hydroxynaringenin | 9 | C21H22O11 | Flavonoids |
| 427 | 3,4-di-hydroxyphenethyol alcohol 4-O-β-D-(6'-O-galloyol)-glucopyranoside | 3 | C21H24O12 | Phenolic acids | 1109 | Eriodictyol-7-O-Rutinoside (Eriocitrin) | 9 | C27H32O15 | Flavonoids |
| 428 | Salvianolic acid B* | 3 | C36H30O16 | Phenolic acids | 1110 | Sakuranin | 9 | C22H24O10 | Flavonoids |
| 429 | 1,2,3-Tri-O-galloyl-β-D-glucose* | 3 | C27H24O18 | Phenolic acids | 1111 | 3,5,7-Trihydroxyflavanone (Pinobanksin) | 9 | C15H12O5 | Flavonoids |
| 430 | Neochlorogenic acid (5-O-Caffeoylquinic acid)* | 3 | C16H18O9 | Phenolic acids | 1112 | Aromadendrin-7-O-glucoside* | 9 | C21H22O11 | Flavonoids |
| 431 | 2-O-Trigalloyl-glucose-glucose | 3 | C33H34O23 | Phenolic acids | 1113 | Flavoyadorinin B | 9 | C23H24O11 | Flavonoids |
| 432 | 2-(3-β-D-glucopyranosyloxy-4-hydroxyphenyl)ethanol-1-O-β-D-glucopyranoside* | 3 | C20H30O13 | Phenolic acids | 1114 | Hispidulin-8-C-glucoside* | 9 | C22H22O11 | Flavonoids |
| 433 | Procyanidin B5 | 3 | C30H26O12 | Tannins | 1115 | Apigenin; 4',5,7-Trihydroxyflavone | 9 | C15H10O5 | Flavonoids |
| 434 | Isocorilagin* | 3 | C27H22O18 | Tannins | 1116 | Luteolin-6,8-di-C-arabinoside | 9 | C25H26O14 | Flavonoids |
| 435 | Phyllanemblinin B* | 3 | C27H22O18 | Tannins | 1117 | Luteolin-7-O-(2''''-O-rhamnosyl)sophorotrioside | 9 | C42H46O23 | Flavonoids |
| 436 | Gallic acid-1-O-xyloside | 3 | C12H14O9 | Tannins | 1118 | Chrysoeriol-8-C-glucoside (Scoparin)* | 9 | C22H22O11 | Flavonoids |
| 437 | Gemin D | 3 | C27H22O18 | Tannins | 1119 | Tricin-7-O-Glucuronide | 9 | C23H22O13 | Flavonoids |
| 438 | Strictinin* | 3 | C27H22O18 | Tannins | 1120 | Selagin | 9 | C16H12O7 | Flavonoids |
| 439 | Corilagin* | 3 | C27H22O18 | Tannins | 1121 | Maohuoside A | 9 | C27H32O12 | Flavonoids |
| 440 | 3-O-Methylellagic acid | 3 | C15H8O8 | Tannins | 1122 | Quercetin-3-O-robinobioside | 9 | C27H30O16 | Flavonoids |
| 441 | (-)-Oleoside 11-methyl ester* | 3 | C17H24O11 | Terpenoids | 1123 | Tamarixetin (3,3',5,7-Tetrahydroxy-4'-Methoxyflavone) | 9 | C16H12O7 | Flavonoids |
| 442 | Germacra-1(10),4,7(11)-trien-9α-ol | 3 | C15H24O | Terpenoids | 1124 | Morin-3-O-lyxoside | 9 | C20H18O11 | Flavonoids |
| 443 | 1,4-Peroxyaurol-ene* | 3 | C15H24O2 | Terpenoids | 1125 | Quercetagetin-7-O-glucoside(Quercetagitrin)* | 9 | C21H20O13 | Flavonoids |
| 444 | 10α-Hydroperoxy-guaia-1,11-diene* | 3 | C15H24O2 | Terpenoids | 1126 | Tamarixetin-3-O-rutinoside | 9 | C28H32O16 | Flavonoids |
| 445 | Procurcumenol | 3 | C15H22O2 | Terpenoids | 1127 | Isorhamnetin-3,7-O-diglucoside | 9 | C28H32O17 | Flavonoids |
| 446 | Aucubin | 3 | C15H22O9 | Terpenoids | 1128 | Morin-3-O-xyloside | 9 | C20H18O11 | Flavonoids |
| 447 | 10α-Hydroxycadin-4-en-15-al | 3 | C15H24O2 | Terpenoids | 1129 | Patuletin-3-O-rutinoside | 9 | C28H32O17 | Flavonoids |
| 448 | 9-Hydroxyselina-4,11-dien-14-al | 3 | C15H22O2 | Terpenoids | 1130 | Quercetin-3-O-rutinoside (Rutin)* | 9 | C27H30O16 | Flavonoids |
| 449 | Nootkatone | 3 | C15H22O | Terpenoids | 1131 | Quercetin-3-O-(4''-O-glucosyl)rhamnoside* | 9 | C27H30O16 | Flavonoids |
| 450 | 5-Hydroxy-2-Carbonylcadinane-12,7-lactone | 3 | C15H20O4 | Terpenoids | 1132 | Quercetin-3-O-sophoroside (Baimaside)* | 9 | C27H30O17 | Flavonoids |
| 451 | (R)-3-(3'-Hydroxybutyl)-2,4,4-trimethylcyclohexa-2,5-dienone | 3 | C13H20O2 | Terpenoids | 1133 | Rhamnetin-3-O-rhamnoside | 9 | C22H22O11 | Flavonoids |
| 452 | Dehydrololiolide | 3 | C11H14O3 | Terpenoids | 1134 | Syringetin-7-O-glucoside* | 9 | C23H24O13 | Flavonoids |
| 453 | 26,27-Dihydroxylanosta-7,9(11),24-trien-3-one (Ganoderiol F) | 3 | C30H46O3 | Terpenoids | 1135 | Myricetin-3-O-galactoside* | 9 | C21H20O13 | Flavonoids |
| 454 | orthosphenic acid | 3 | C30H48O5 | Terpenoids | 1136 | 6-C-Methylquercetin-3-O-glucoside* | 9 | C22H22O12 | Flavonoids |
| 455 | Clavatol | 3 | C10H12O3 | Terpenoids | 1137 | Quercetin-4'-O-glucuronide | 9 | C21H18O13 | Flavonoids |
| 456 | DL-2-Aminoadipic acid | 4 | C6H11NO4 | Alkaloids | 1138 | 1,8-dihydroxy-2,6-dimethoxy-5-{[(2s,3r,4s,5s,6r)-3,4,5-trihydroxy-6-(hydroxymethyl)oxan-2-yl]oxy}xanthen-9-one | 9 | C21H22O12 | Flavonoids |
| 457 | Diethanolamine | 4 | C4H11NO2 | Alkaloids | 1139 | Lancerin; 4-C-Glucosyl-1,3,7-Trihydroxyxanthone | 9 | C19H18O10 | Flavonoids |
| 458 | N-Oleoylethanolamine | 4 | C20H39NO2 | Alkaloids | 1140 | tenuiflorin C glucoside* | 9 | C22H22O12 | Flavonoids |
| 459 | Sarmentosin | 4 | C11H17NO7 | Alkaloids | 1141 | 3-Methyl-4,8-dihydroxy-3,4-dihydroisocoumarin | 9 | C10H10O4 | Lignans and Coumarins |
| 460 | 1-(4-((3,4,5-trihydroxy-6-(hydroxymethyl)tetrahydro-2H-pyran-2-yl)oxy)benzyl)-1,2,3,4-tetrahydroisoquinoline-5,6,7-triol | 4 | C22H27NO9 | Alkaloids | 1142 | 7-hydroxy-2H-1-benzopyran-2-one* | 9 | C9H6O3 | Lignans and Coumarins |
| 461 | Sinapoylputrescine | 4 | C15H22N2O4 | Alkaloids | 1143 | 7-C-Glucosylcoumarin* | 9 | C15H16O7 | Lignans and Coumarins |
| 462 | Feruloylspermidine | 4 | C17H27N3O3 | Alkaloids | 1144 | 6-Hydroxycoumarin* | 9 | C9H6O3 | Lignans and Coumarins |
| 463 | L-Aspartic acid | 4 | C4H7NO4 | Amino acids and derivatives | 1145 | Meranzin | 9 | C15H16O4 | Lignans and Coumarins |
| 464 | N-Methylglycine | 4 | C3H7NO2 | Amino acids and derivatives | 1146 | Isoscopoletin-β-D-glucoside* | 9 | C16H18O9 | Lignans and Coumarins |
| 465 | Pyroglutamic acid | 4 | C5H7NO3 | Amino acids and derivatives | 1147 | Hexadecanedioic acid | 9 | C16H30O4 | Lipids |
| 466 | 4-Hydroxy-L-glutamic acid | 4 | C5H9NO5 | Amino acids and derivatives | 1148 | Undecylic Acid | 9 | C11H22O2 | Lipids |
| 467 | L-Serine | 4 | C3H7NO3 | Amino acids and derivatives | 1149 | 10,16-Dihydroxypalmitic acid | 9 | C16H32O4 | Lipids |
| 468 | L-Glutamic acid-O-glycoside | 4 | C11H19NO9 | Amino acids and derivatives | 1150 | 11-Octadecanoic acid(Vaccenic acid)* | 9 | C18H34O2 | Lipids |
| 469 | 3-Cyano-L-alanine | 4 | C4H6N2O2 | Amino acids and derivatives | 1151 | Petroselinic acid* | 9 | C18H34O2 | Lipids |
| 470 | N6-Acetyl-L-lysine | 4 | C8H16N2O3 | Amino acids and derivatives | 1152 | 7S,8S-DiHODE; (9Z,12Z)-(7S,8S)-Dihydroxyoctadeca-9,12-dienoic acid* | 9 | C18H32O4 | Lipids |
| 471 | Trans-4-Hydroxy-L-proline* | 4 | C5H9NO3 | Amino acids and derivatives | 1153 | 13-Hydroperoxy-9Z,11E-octadecadienoic acid* | 9 | C18H32O4 | Lipids |
| 472 | DL-Methionine | 4 | C5H11NO2S | Amino acids and derivatives | 1154 | Dihydrosphingosine | 9 | C18H39NO2 | Lipids |
| 473 | Oxamic acid | 4 | C2H3NO3 | Amino acids and derivatives | 1155 | 4-Hydroxysphinganine; Phytosphingosine | 9 | C18H39NO3 | Lipids |
| 474 | L-Asparagine | 4 | C4H8N2O3 | Amino acids and derivatives | 1156 | 3-Dehydrosphinganine | 9 | C18H37NO2 | Lipids |
| 475 | 1-Methylhistidine | 4 | C7H11N3O2 | Amino acids and derivatives | 1157 | Nicotinate adenine dinucleotide phosphate | 9 | C21H27N6O18P3 | Nucleotides and derivatives |
| 476 | 6-Hydroxydopaquinone | 4 | C9H9NO5 | Amino acids and derivatives | 1158 | Isopentenyladenine-7-N-glucoside | 9 | C16H23N5O5 | Nucleotides and derivatives |
| 477 | Epicatechin glucoside* | 4 | C21H24O11 | Flavonoids | 1159 | Guanosine 5'-monophosphate | 9 | C10H14N5O8P | Nucleotides and derivatives |
| 478 | (2S)-Abyssinone II* | 4 | C20H20O5 | Flavonoids | 1160 | Shikimic acid | 9 | C7H10O5 | Organic acids |
| 479 | Naringenin-7-O-Rutinoside-4'-O-glucoside | 4 | C33H42O19 | Flavonoids | 1161 | 2,2-Dimethylsuccinic acid | 9 | C6H10O4 | Organic acids |
| 480 | 3',4',5,7-Tetramethoxyflavone; Luteolin Tetramethyl Ether | 4 | C19H18O6 | Flavonoids | 1162 | 3-Dehydroshikimic acid | 9 | C7H8O5 | Organic acids |
| 481 | Fraxetin (7,8-Dihydroxy-6-methoxycoumarin) | 4 | C10H8O5 | Lignans and Coumarins | 1163 | 2-Amino-5-oxohexanoic acid* | 9 | C6H11NO3 | Organic acids |
| 482 | 3,4-Dihydro-4-(4-hydroxy-3-methoxyphenyl)-3-(hydroxymethyl)-6,7-dimethoxy-(3R,4S)-2-naphthalenecarboxaldehyde | 4 | C21H22O6 | Lignans and Coumarins | 1164 | Urocanic acid | 9 | C6H6N2O2 | Organic acids |
| 483 | β-Nicotinamide mononucleotide | 4 | C11H15N2O8P | Nucleotides and derivatives | 1165 | trans-Aconitic acid | 9 | C6H6O6 | Organic acids |
| 484 | Citicoline | 4 | C14H26N4O11P2 | Nucleotides and derivatives | 1166 | Mandelic acid-β-glucoside | 9 | C14H18O8 | Organic acids |
| 485 | 5,6-Dihydro-5-methyluracil | 4 | C5H8N2O2 | Nucleotides and derivatives | 1167 | 3-Furoic acid | 9 | C5H4O3 | Organic acids |
| 486 | N6-Isopentenyladenine | 4 | C10H13N5 | Nucleotides and derivatives | 1168 | 2,4-Dihydroxy-6-methoxyacetophenone* | 9 | C9H10O4 | Others |
| 487 | Malonic acid | 4 | C3H4O4 | Organic acids | 1169 | Primin* | 9 | C12H16O3 | Others |
| 488 | L-Pipecolic Acid* | 4 | C6H11NO2 | Organic acids | 1170 | Senkyunolide K | 9 | C12H16O3 | Others |
| 489 | 2-Picolinic acid | 4 | C6H5NO2 | Organic acids | 1171 | Noreugenin-7-O-glucoside* | 9 | C16H18O9 | Others |
| 490 | 5-Aminovaleric acid | 4 | C5H11NO2 | Organic acids | 1172 | N-Acetyl-D-glucosamine-1-phosphate | 9 | C8H16NO9P | Others |
| 491 | 3-Ureidopropionic Acid | 4 | C4H8N2O3 | Organic acids | 1173 | 1-(sn-Glycero-3-phospho)-1D-myo-inositol | 9 | C9H19O11P | Others |
| 492 | Citric Acid diglucoside | 4 | C18H28O17 | Organic acids | 1174 | Erythorbic Acid; Isoascorbic Acid | 9 | C6H8O6 | Others |
| 493 | γ-Aminobutyric acid | 4 | C4H9NO2 | Organic acids | 1175 | 2-O-α-D-Glucopyranosyl-L-ascorbic acid | 9 | C12H18O11 | Others |
| 494 | Itaconic acid | 4 | C5H6O4 | Organic acids | 1176 | 4-Nitrophenol | 9 | C6H5NO3 | Phenolic acids |
| 495 | N-Methyl-4-aminobutyric acid | 4 | C5H11NO2 | Organic acids | 1177 | 4-O-Glucosyl-3,4-dihydroxybenzyl alcohol | 9 | C13H18O8 | Phenolic acids |
| 496 | 3,4-Methylenedioxycinnamaldehyde | 4 | C10H8O3 | Others | 1178 | 4-O-(6'-O-Glucosylferuloyl)-3,4-dihydroxybenzyl alcohol | 9 | C23H26O11 | Phenolic acids |
| 497 | 6,7-dimethoxy-2-[2-phenylethyl]chromone | 4 | C19H18O4 | Others | 1179 | Beta-asarone | 9 | C12H16O3 | Phenolic acids |
| 498 | D-Ribose | 4 | C5H10O5 | Others | 1180 | Vanillic acid methyl ester* | 9 | C9H10O4 | Phenolic acids |
| 499 | D-Melezitose | 4 | C18H32O16 | Others | 1181 | 3-Hydroxy-5-Methylphenol-1-O-Glucoside | 9 | C13H18O7 | Phenolic acids |
| 500 | Melibiose | 4 | C12H22O11 | Others | 1182 | 2,4-Dihydroxybenzoic acid* | 9 | C7H6O4 | Phenolic acids |
| 501 | Maltotriose | 4 | C18H32O16 | Others | 1183 | Arillatose A | 9 | C34H40O19 | Phenolic acids |
| 502 | D-Trehalose* | 4 | C12H22O11 | Others | 1184 | 1-O-Feruloyl-β-D-glucose | 9 | C16H20O9 | Phenolic acids |
| 503 | Isonicotinic acid | 4 | C6H5NO2 | Others | 1185 | Methyl 3-O-Methyl Gallate | 9 | C9H10O5 | Phenolic acids |
| 504 | Thiamine (Vitamin B1) | 4 | C12H17N4OS+ | Others | 1186 | Salirepin | 9 | C13H18O8 | Phenolic acids |
| 505 | 1-(4-Hydroxybenzoyl)Glucose; 25545-07-7 | 4 | C13H16O8 | Phenolic acids | 1187 | 1-O-p-Coumaroylquinic acid | 9 | C16H18O8 | Phenolic acids |
| 506 | (E)-3-[(2S,3S)-2-(4-hydroxy-3-methoxyphenyl)-7-methoxy-3-methyl-2,3-dihydro-1-benzofuran-5-yl]prop-2-enal* | 4 | C20H20O5 | Phenolic acids | 1188 | 2,6-Dihydroxy-4-isopropylphenyl-1-O-β-D-glucoside | 9 | C15H22O8 | Phenolic acids |
| 507 | 1-O-Salicyloyl-β-D-glucose | 4 | C13H16O8 | Phenolic acids | 1189 | Elemicin* | 9 | C12H16O3 | Phenolic acids |
| 508 | Ethyl maltol | 4 | C7H8O3 | Phenolic acids | 1190 | 5-Acetylsalicylic acid | 9 | C9H8O4 | Phenolic acids |
| 509 | Salidroside | 4 | C14H20O7 | Phenolic acids | 1191 | 1-O-(p-coumaroyl) 3-Hydroxy-3-methylglutaric acid* | 9 | C15H16O7 | Phenolic acids |
| 510 | Isochlorogenic acid C* | 4 | C25H24O12 | Phenolic acids | 1192 | Chlorogenic acid (3-O-Caffeoylquinic acid)* | 9 | C16H18O9 | Phenolic acids |
| 511 | Verbasoside | 4 | C20H30O12 | Phenolic acids | 1193 | Ferulic acid-4-O-glucoside | 9 | C16H20O9 | Phenolic acids |
| 512 | Grevilloside L | 4 | C21H24O11 | Phenolic acids | 1194 | Malonyl Tachioside | 9 | C16H20O11 | Phenolic acids |
| 513 | 2-Acetyl-3-hydroxyphenyl-1-O-glucoside | 4 | C15H20O7 | Phenolic acids | 1195 | 5-O-p-Coumaroylquinic acid | 9 | C16H18O8 | Phenolic acids |
| 514 | 3-Hydroxy-4-methoxybenzoic acid; Isovanillic Acid | 4 | C8H8O4 | Phenolic acids | 1196 | Grevilloside F | 9 | C15H18O9 | Phenolic acids |
| 515 | Feruloylmalic acid | 4 | C14H14O8 | Phenolic acids | 1197 | Arenarioside | 9 | C34H44O19 | Phenolic acids |
| 516 | Sanguiin H4* | 4 | C27H22O18 | Tannins | 1198 | 4-O-p-Coumaroylquinic acid | 9 | C16H18O8 | Phenolic acids |
| 517 | 6-O-Vanilloylajugol | 4 | C23H30O12 | Terpenoids | 1199 | Lavandulifolioside | 9 | C34H44O19 | Phenolic acids |
| 518 | Reynosin | 4 | C15H20O3 | Terpenoids | 1200 | Raspberryketone glucoside | 9 | C16H22O7 | Phenolic acids |
| 519 | Isoquinoline | 5 | C9H7N | Alkaloids | 1201 | Dunalianoside C* | 9 | C21H22O11 | Phenolic acids |
| 520 | Feruloylcholine | 5 | C15H22NO4+ | Alkaloids | 1202 | Cryptochlorogenic acid (4-O-Caffeoylquinic acid)* | 9 | C16H18O9 | Phenolic acids |
| 521 | L-Leucyl-L-phenylalanine | 5 | C15H22N2O3 | Amino acids and derivatives | 1203 | Trans-5-O-(p-Coumaroyl)shikimate | 9 | C16H16O7 | Phenolic acids |
| 522 | L-Methionine methyl ester | 5 | C6H13NO2S | Amino acids and derivatives | 1204 | Homovanillic alcohol; 4-Hydroxy-3-methoxyphenethanol | 9 | C9H12O3 | Phenolic acids |
| 523 | Chalcone | 5 | C15H12O | Flavonoids | 1205 | (S)-2-Hydroxy-3-(4-Hydroxyphenyl)Propanoic Acid* | 9 | C9H10O4 | Phenolic acids |
| 524 | Liquiritigenin-7-O-apioside-4'-O-glucoside | 5 | C26H30O13 | Flavonoids | 1206 | 3-O-Feruloylquinic acid | 9 | C17H20O9 | Phenolic acids |
| 525 | Apigenin-7-O-glucuronide* | 5 | C21H18O11 | Flavonoids | 1207 | 2-β-D-Glucopyranosyloxy-5-hydroxy-phenylacetic acid | 9 | C14H18O9 | Phenolic acids |
| 526 | Baicalin* | 5 | C21H18O11 | Flavonoids | 1208 | Bis(p-Coumaroyl)tartaric acid | 9 | C22H18O10 | Phenolic acids |
| 527 | Apigenin-7-O-rutinoside-4'-O-Sophoroside* | 5 | C39H50O24 | Flavonoids | 1209 | 3-(3-Hydroxyphenyl)-propionic acid | 9 | C9H10O3 | Phenolic acids |
| 528 | 6-Methoxyquercetin-3-O-Xyloside | 5 | C21H20O12 | Flavonoids | 1210 | 3-O-(p-coumaroyl) 3-Hydroxy-3-methylglutaric acid* | 9 | C15H16O7 | Phenolic acids |
| 529 | Patuletin-3-O-glucoside* | 5 | C22H22O13 | Flavonoids | 1211 | 3-(4-Hydroxyphenyl)-propionic acid* | 9 | C9H10O3 | Phenolic acids |
| 530 | Isofraxidin-7-O-glucoside | 5 | C17H20O10 | Lignans and Coumarins | 1212 | Chlorogenic acid methyl ester | 9 | C17H20O9 | Phenolic acids |
| 531 | Fraxiresinol | 5 | C21H24O8 | Lignans and Coumarins | 1213 | Ellagic acid-4-O-rhamnoside* | 9 | C20H16O12 | Tannins |
| 532 | 5'-Methoxyisolariciresinol-9'-O-glucoside | 5 | C27H36O12 | Lignans and Coumarins | 1214 | Eschweilenol C* | 9 | C20H16O12 | Tannins |
| 533 | Dihydrodehydrodiconiferyl alcohol-4-O-glucoside* | 5 | C26H34O11 | Lignans and Coumarins | 1215 | 6-DeoxyCatalpol | 9 | C15H22O9 | Terpenoids |
| 534 | 12-Hydroxy-16-heptadecynoic acid | 5 | C17H30O3 | Lipids | 1216 | 6,9-Dihydroxy-7-megastigmen-3-one | 9 | C13H22O3 | Terpenoids |
| 535 | Arachidic acid | 5 | C20H40O2 | Lipids | 1217 | 8-Epiloganin | 9 | C17H26O10 | Terpenoids |
| 536 | Palmitic acid | 5 | C16H32O2 | Lipids | 1218 | 2-(Acetylamino)-3-phenyl-2-propenoic acid | 10 | C11H11NO3 | Alkaloids |
| 537 | LysoPC 12:0 | 5 | C20H42NO7P | Lipids | 1219 | o-Carboxy-5-hydroxytryptamine | 10 | C11H12N2O3 | Alkaloids |
| 538 | LysoPE 15:0(2n isomer)* | 5 | C20H42NO7P | Lipids | 1220 | Nicotianamine | 10 | C12H21N3O6 | Alkaloids |
| 539 | 4-Hydroxycyclohexylcarboxylic acid | 5 | C7H12O3 | Organic acids | 1221 | 2-Ethyl-3-methylmaleimide-N-glucoside | 10 | C13H19NO7 | Alkaloids |
| 540 | Creatine | 5 | C4H9N3O2 | Organic acids | 1222 | Guanidinoacetate | 10 | C3H7N3O2 | Alkaloids |
| 541 | 5-Hydroxymethyl-2-furancarboxylic acid | 5 | C6H6O4 | Organic acids | 1223 | 2-Glucosyloxy-2-phenylacetic acid amide | 10 | C14H19NO7 | Alkaloids |
| 542 | 4-Hydroxybenzaldehyde | 5 | C7H6O2 | Others | 1224 | N-Feruloylagmatine | 10 | C15H22N4O3 | Alkaloids |
| 543 | Inositol* | 5 | C6H12O6 | Others | 1225 | Hypaphorine | 10 | C14H18N2O2 | Alkaloids |
| 544 | D-Mannose* | 5 | C6H12O6 | Others | 1226 | Quinine | 10 | C20H24O2N2 | Alkaloids |
| 545 | D-Galactose* | 5 | C6H12O6 | Others | 1227 | Methionylvaline | 10 | C10H20N2O3S | Amino acids and derivatives |
| 546 | D-Fructose* | 5 | C6H12O6 | Others | 1228 | L-Lysine | 10 | C6H14N2O2 | Amino acids and derivatives |
| 547 | Pyridoxal | 5 | C8H9NO3 | Others | 1229 | L-Lysine-Butanoic Acid | 10 | C10H22N2O4 | Amino acids and derivatives |
| 548 | Trollioside | 5 | C19H26O9 | Phenolic acids | 1230 | L-Glutamine | 10 | C5H10N2O3 | Amino acids and derivatives |
| 549 | 3,4-Dihydroxybenzoic acid (Protocatechuic acid)* | 5 | C7H6O4 | Phenolic acids | 1231 | S-(5'-Adenosyl)-L-methionine | 10 | C15H22N6O5S | Amino acids and derivatives |
| 550 | 2,3-Dihydroxybenzoic Acid* | 5 | C7H6O4 | Phenolic acids | 1232 | N-ethyl-leucine | 10 | C8H17NO2 | Amino acids and derivatives |
| 551 | Androsin | 5 | C15H20O8 | Phenolic acids | 1233 | N-Methyl-α-aminoisobutyric acid | 10 | C5H11NO2 | Amino acids and derivatives |
| 552 | Caffeoyl-p-coumaroyltartaric acid | 5 | C22H18O11 | Phenolic acids | 1234 | S-Adenosylmethionine | 10 | C15H23N6O5S+ | Amino acids and derivatives |
| 553 | 4-O-(6'-O-Glucosyl-4''-hydroxybenzoyl)-4-hydroxybenzyl alcohol | 5 | C20H22O9 | Phenolic acids | 1235 | N-Methyl-L-Glutamate | 10 | C6H11NO4 | Amino acids and derivatives |
| 554 | 6'-O-Sinapoylsucrose* | 5 | C23H32O15 | Phenolic acids | 1236 | S-Sulfo-L-Cysteine | 10 | C3H7NO5S2 | Amino acids and derivatives |
| 555 | Oleoacteoside | 5 | C46H58O25 | Phenolic acids | 1237 | Nitroarginine | 10 | C6H13N5O4 | Amino acids and derivatives |
| 556 | 4-p-Cumaroyl-rhamnosyl-(1→6)-D-glucose | 5 | C21H28O12 | Phenolic acids | 1238 | L-Glutamine-O-glycoside | 10 | C11H20N2O8 | Amino acids and derivatives |
| 557 | Isosalicin | 5 | C13H18O7 | Phenolic acids | 1239 | 3-Hydroxyphloretin-4'-O-glucoside | 10 | C21H24O11 | Flavonoids |
| 558 | 3,5-Digalloylshikimic acid | 5 | C23H26O11 | Phenolic acids | 1240 | Epicatechin-3-(3''-O-methyl)gallate | 10 | C23H20O10 | Flavonoids |
| 559 | 7-Hydroxy-costol-glucoside | 5 | C21H34O7 | Terpenoids | 1241 | 3'-O-Methyl-epicatechin | 10 | C16H16O6 | Flavonoids |
| 560 | 7-Hydroxy-costol-malonyl glucoside | 5 | C24H36O10 | Terpenoids | 1242 | Epicatechin-(4β->8)-epigallocatechin | 10 | C30H26O13 | Flavonoids |
| 561 | N-Isobutyl-4,5-epoxy-2E-decaenamide | 6 | C14H25NO2 | Alkaloids | 1243 | Epigallocatechin | 10 | C15H14O7 | Flavonoids |
| 562 | (3S)-1,3-dihydroxy-2,3,3a,4-tetrahydropyrrolo[2,1-b]quinazolin-9(1H)-one | 6 | C11H12N2O3 | Alkaloids | 1244 | Epicatechin gallate | 10 | C22H18O10 | Flavonoids |
| 563 | Benzamide | 6 | C7H7NO | Alkaloids | 1245 | Epigallocatechin-3-O-gallate | 10 | C22H18O11 | Flavonoids |
| 564 | Dicentrine | 6 | C20H21NO4 | Alkaloids | 1246 | 7-O-Galloyltricetiflavan | 10 | C22H18O10 | Flavonoids |
| 565 | 2-Phenylethylamine | 6 | C8H11N | Alkaloids | 1247 | Gallocatechin gallate | 10 | C22H18O11 | Flavonoids |
| 566 | Stepharine | 6 | C18H19NO3 | Alkaloids | 1248 | Eriodictyol-7-O-glucoside* | 10 | C21H22O11 | Flavonoids |
| 567 | Cyclo(Pro-Leu) | 6 | C11H18N2O2 | Amino acids and derivatives | 1249 | Silibinin | 10 | C25H22O10 | Flavonoids |
| 568 | Luteolin-7-O-(6''-malonyl)glucuronide-5-O-rhamnoside | 6 | C30H30O19 | Flavonoids | 1250 | Taxifolin(Dihydroquercetin) | 10 | C15H12O7 | Flavonoids |
| 569 | kaempferol-3-sinapoyldiglucoside-7-glucoside | 6 | C44H50O25 | Flavonoids | 1251 | Acacetin-7-O-galactoside | 10 | C22H22O10 | Flavonoids |
| 570 | Xanthotoxol; 8-Hydroxypsoralen | 6 | C11H6O4 | Lignans and Coumarins | 1252 | 5,7-Dihydroxy-4-methoxyflavone-3-O-xylose-(1-6)-glucose | 10 | C27H30O15 | Flavonoids |
| 571 | 2-[(1s,2r,3r)-1-hydroxy-4-(3-hydroxy-4,5-dimethoxyphenyl)-2,3-dimethylbutyl]-4,5-dimethoxyphenol | 6 | C22H30O7 | Lignans and Coumarins | 1253 | 3'-Demethylnobiletin | 10 | C20H20O8 | Flavonoids |
| 572 | Olivil | 6 | C20H24O7 | Lignans and Coumarins | 1254 | 6-Hydroxyluteolin | 10 | C15H10O7 | Flavonoids |
| 573 | Eicosenoic acid | 6 | C20H38O2 | Lipids | 1255 | Oroxin A | 10 | C21H20O10 | Flavonoids |
| 574 | LysoPC 16:1(2n isomer)* | 6 | C24H48NO7P | Lipids | 1256 | Orientin-7-O-glucoside | 10 | C27H30O16 | Flavonoids |
| 575 | LysoPC 18:2(2n isomer) | 6 | C26H50NO7P | Lipids | 1257 | Azaleatin (5-O-Methylquercetin) | 10 | C16H12O7 | Flavonoids |
| 576 | 5'-Deoxy-5'-(methylthio)adenosine | 6 | C11H15N5O3S | Nucleotides and derivatives | 1258 | Quercetin-3-O-(2''-O-galloyl)galactoside | 10 | C28H24O16 | Flavonoids |
| 577 | Isoguanine | 6 | C5H5N5O | Nucleotides and derivatives | 1259 | Quercetin-3-O-(6''-O-galloyl)galactoside | 10 | C28H24O16 | Flavonoids |
| 578 | 2-Methyl-3-oxosuccinic acid | 6 | C5H6O5 | Organic acids | 1260 | Kaempferol-3-O-(6''-galloyl)glucoside* | 10 | C28H24O15 | Flavonoids |
| 579 | Annuionone D | 6 | C13H20O3 | Others | 1261 | Kaempferol-3-O-(6''-galloyl)galactoside* | 10 | C28H24O15 | Flavonoids |
| 580 | Monoacetyldapsone | 6 | C14H14N2O3S | Others | 1262 | Tamarixetin-3-O-(6''-malonyl)glucoside* | 10 | C25H24O15 | Flavonoids |
| 581 | 3-(4-hydroxyphenyl)-3-oxopropyl beta-D-glucopyranoside* | 6 | C15H20O8 | Phenolic acids | 1263 | 1,8-dihydroxy-4,5-dimethoxy-3-{[(2s,3r,4s,5s,6r)-3,4,5-trihydroxy-6-(hydroxymethyl)oxan-2-yl]oxy}xanthen-9-one* | 10 | C21H22O12 | Flavonoids |
| 582 | Methyl Brevifolincarboxylate | 6 | C14H10O8 | Phenolic acids | 1264 | 5,7,3',4',5'-Pentahydroxyflavan-7-gallate | 10 | C22H18O10 | Flavonoids |
| 583 | 1-O-Galloyl-6-O-p-Coumaroyl-β-D-glucose* | 6 | C22H22O12 | Phenolic acids | 1265 | Fraxidin (8-Hydroxy-6,7-dimethoxycoumarin) | 10 | C11H10O5 | Lignans and Coumarins |
| 584 | Dihydrocaffeic acid | 6 | C9H10O4 | Phenolic acids | 1266 | Piperitol | 10 | C20H20O6 | Lignans and Coumarins |
| 585 | p-Menth-4(5)-ene-1,2-diol-1-O-β-D-(2-O-acetyl)-glucoside | 6 | C18H30O8 | Terpenoids | 1267 | Pinoresinol* | 10 | C20H22O6 | Lignans and Coumarins |
| 586 | (1S,4aS,7aR)-7-(3-hydroxybutyl)-4-((((2R,3R,4S,5S,6R)-3,4,5-trihydroxy-6-(hydroxymethyl)tetrahydro-2H-pyran-2-yl)oxy)methyl)-1,4a,5,6,7,7a-hexahydrocyclopenta[c]pyran-1-yl acetate | 6 | C21H34O10 | Terpenoids | 1268 | Balanophonin | 10 | C20H20O6 | Lignans and Coumarins |
| 587 | N-(4-oxopentyl)-acetamide | 7 | C7H13NO2 | Alkaloids | 1269 | 4-ketopinoresinol | 10 | C20H20O7 | Lignans and Coumarins |
| 588 | Spermidine | 7 | C7H19N3 | Alkaloids | 1270 | fragransol D | 10 | C21H22O6 | Lignans and Coumarins |
| 589 | 2-Amino-4,5-dihydro-1H-imidazole-4-acetic acid | 7 | C5H9N3O2 | Alkaloids | 1271 | 4-HydroxyseSamin | 10 | C20H18O7 | Lignans and Coumarins |
| 590 | Salicylamide | 7 | C7H7NO2 | Alkaloids | 1272 | taiwanin A | 10 | C20H14O6 | Lignans and Coumarins |
| 591 | N',N'',N'''-p-Coumaroyl-cinnamoyl-caffeoyl spermidine | 7 | C34H37N3O6 | Alkaloids | 1273 | Erythro-Guaiacylglycerol-β-O-4'-dehydrodisinapyl Ether | 10 | C31H36O11 | Lignans and Coumarins |
| 592 | N',N''-Diferuloylspermidine | 7 | C27H35N3O6 | Alkaloids | 1274 | (3r,4r)-3-(2h-1,3-benzodioxol-5-ylmethyl)-4-[(4-hydroxy-3-methoxyphenyl)methyl]oxolan-2-one* | 10 | C20H20O6 | Lignans and Coumarins |
| 593 | Caffeoylcholine-3-O-glucoside* | 7 | C20H30NO9+ | Alkaloids | 1275 | 9-hydroxysesamin | 10 | C20H18O7 | Lignans and Coumarins |
| 594 | Caffeoylcholine-4-O-glucoside* | 7 | C20H30NO9+ | Alkaloids | 1276 | Epipinoresinol* | 10 | C20H22O6 | Lignans and Coumarins |
| 595 | N-(p-Coumaroyl)serotonin Glucoside | 7 | C25H28N2O8 | Alkaloids | 1277 | (5R,6S,7R)-5-(3,4,5-Trimethoxyphenyl)-7-methyl-8-oxo-5,6,7,8-tetrahydronaphtho[2,3-d]-1,3-dioxole-6-carboxylic acidethylester | 10 | C24H26O8 | Lignans and Coumarins |
| 596 | N,N-Dimethyl-5-methoxytryptamine | 7 | C13H18N2O | Alkaloids | 1278 | 3-Hydroxyoctadecanoic Acid | 10 | C18H36O3 | Lipids |
| 597 | Indole-3-cyano-2-O-glucoside | 7 | C16H18N2O5S | Alkaloids | 1279 | 9-Hydroxyoctadeca-6,10,12,15-Tetraenoic Acid | 10 | C18H28O3 | Lipids |
| 598 | Tryptamine | 7 | C10H12N2 | Alkaloids | 1280 | (R)-Beta-Hydroxypalmitic Acid* | 10 | C16H32O3 | Lipids |
| 599 | Trigonelline | 7 | C7H7NO2 | Alkaloids | 1281 | 5'-Deoxyadenosine* | 10 | C10H13N5O3 | Nucleotides and derivatives |
| 600 | trans-3'-Hydroxycotinine-O-glucoside | 7 | C16H22N2O7 | Alkaloids | 1282 | Xanthosine | 10 | C10H12N4O6 | Nucleotides and derivatives |
| 601 | Stachydrine | 7 | C7H13NO2 | Alkaloids | 1283 | 2'-Deoxycytidine | 10 | C9H13N3O4 | Nucleotides and derivatives |
| 602 | 5-Hydroxyquinoline | 7 | C11H11NO2 | Alkaloids | 1284 | Uracil | 10 | C4H4N2O2 | Nucleotides and derivatives |
| 603 | 2,4-Dihydroxyquinoline | 7 | C9H7NO2 | Alkaloids | 1285 | 2-Methylsuccinic acid | 10 | C5H8O4 | Organic acids |
| 604 | N-Acetyl-L-glycine | 7 | C4H7NO3 | Amino acids and derivatives | 1286 | 2-Hydroxyhexadecanoic acid* | 10 | C16H32O3 | Organic acids |
| 605 | N-Methylisoleucine | 7 | C7H15NO2 | Amino acids and derivatives | 1287 | D-Malic acid* | 10 | C4H6O5 | Organic acids |
| 606 | Met-Ser-Ala | 7 | C11H21N3O5S1 | Amino acids and derivatives | 1288 | Fumaric acid | 10 | C4H4O4 | Organic acids |
| 607 | γ-Glutamyl-L-valine | 7 | C10H18N2O5 | Amino acids and derivatives | 1289 | DL-Glyceric Acid | 10 | C3H6O4 | Organic acids |
| 608 | L-Glycyl-L-isoleucine | 7 | C8H16N2O3 | Amino acids and derivatives | 1290 | Succinic semialdehyde | 10 | C4H6O3 | Organic acids |
| 609 | N-Alpha-Acetyl-L-Asparagine | 7 | C6H10N2O4 | Amino acids and derivatives | 1291 | 3-Hydroxy-3-Methyl-2-Oxopentanoic Acid | 10 | C6H10O4 | Organic acids |
| 610 | Hydroxy isoliquiritigenin glucoside* | 7 | C21H22O10 | Flavonoids | 1292 | 2-Acetyl-2-Hydroxybutanoic Acid | 10 | C6H10O4 | Organic acids |
| 611 | Okanin-4'-O-glucoside(Marein)* | 7 | C21H22O11 | Flavonoids | 1293 | Ethyl butyrate* | 10 | C6H12O2 | Organic acids |
| 612 | Okanin-4'-O-(6''-O-malonyl)glucoside | 7 | C24H24O14 | Flavonoids | 1294 | Ethyl isobutyrate* | 10 | C6H12O2 | Organic acids |
| 613 | Okanin-4'-O-glucosyl-O-glucoside* | 7 | C27H32O16 | Flavonoids | 1295 | Benzaldehyde | 10 | C7H6O | Others |
| 614 | Cinchonain Id* | 7 | C24H20O9 | Flavonoids | 1296 | Syringaldehyde; 4-Hydroxy-3,5-Dimethoxybenzaldehyde | 10 | C9H10O4 | Others |
| 615 | Catechin-(7,8-bc)-4β-(3,4-dihydroxyphenyl)-dihydro-2-(3H)-one* | 7 | C24H20O9 | Flavonoids | 1297 | xylosyl -5-hydroxychromone-7-O-β-D-glucoside | 10 | C20H24O13 | Others |
| 616 | Cinchonain Ib* | 7 | C24H20O9 | Flavonoids | 1298 | 7-Hydroxy-4-chromone | 10 | C9H6O3 | Others |
| 617 | Catechin-(7,8-bc)-4α-(3,4-dihydroxyphenyl)-dihydro-2-(3H)-one* | 7 | C24H20O9 | Flavonoids | 1299 | 6,7-dimethoxy-2-[2-(4'-hydroxy-3'-methoxyphenyl)ethyl]chromone* | 10 | C20H20O6 | Others |
| 618 | Cinchonain Ia* | 7 | C24H20O9 | Flavonoids | 1300 | Dihydroquercetin | 10 | C15H12O7 | Others |
| 619 | 7,4'-Di-O-galloyltricetiflavan | 7 | C29H22O14 | Flavonoids | 1301 | 3,4-Dihydroxyacetophenone | 10 | C8H8O3 | Others |
| 620 | Cinchonain Ic* | 7 | C24H20O9 | Flavonoids | 1302 | 3-Hydroxy-1-(4-hydroxy-3,5-dimethoxyphenyl)propan-1-one | 10 | C11H14O5 | Others |
| 621 | 8-[2-(3,4-dihydroxyphenyl)-3,5,7-trihydroxy-3,4-dihydro-2h-1-benzopyran-4-yl]-2-(4-hydroxyphenyl)-3,4-dihydro-2h-1-benzopyran-3,5,7-triol | 7 | C30H26O11 | Flavonoids | 1303 | Salicylacyl Glucuronide | 10 | C13H14O9 | Others |
| 622 | Afzelechin (3,5,7,4'-Tetrahydroxyflavan) | 7 | C15H14O5 | Flavonoids | 1304 | 3-Hydroxy-5-Methoxyphenyl-6-O-Galloyl-Beta-D-Galactopyranoside* | 10 | C20H22O12 | Others |
| 623 | Epicatechin-epiafzelechin | 7 | C30H26O11 | Flavonoids | 1305 | D-Threonic Acid | 10 | C4H8O5 | Others |
| 624 | Eriodictyol (5,7,3',4'-Tetrahydroxyflavanone) | 7 | C15H12O6 | Flavonoids | 1306 | 1,5-Anhydro-D-glucitol | 10 | C6H12O5 | Others |
| 625 | Hesperetin-5-O-glucoside | 7 | C22H24O11 | Flavonoids | 1307 | 3-Dehydro-L-Threonic Acid* | 10 | C4H6O5 | Others |
| 626 | Isohemiphloin | 7 | C21H22O10 | Flavonoids | 1308 | beta-L-fucose 1-phosphate | 10 | C6H13O8P | Others |
| 627 | Naringenin-7-O-glucoside (Prunin)* | 7 | C21H22O10 | Flavonoids | 1309 | 2,3-Dihydroxypropanal | 10 | C3H6O3 | Others |
| 628 | Phellodensin D | 7 | C20H20O6 | Flavonoids | 1310 | Protocatechuic acid 4-O-(2''-O-Vanilloyl)Glucoside | 10 | C21H22O12 | Phenolic acids |
| 629 | Pinocembrin-7-O-glucoside (Pinocembroside) | 7 | C21H22O9 | Flavonoids | 1311 | Methyl gallate | 10 | C8H8O5 | Phenolic acids |
| 630 | Hesperetin-3'-O-glucoside* | 7 | C22H24O11 | Flavonoids | 1312 | D-Threo-guaiacylglycerol-7-O-β-D-glucoside | 10 | C16H24O10 | Phenolic acids |
| 631 | Malonyl isoSakuranin | 7 | C25H26O13 | Flavonoids | 1313 | Leonuriside A | 10 | C14H20O9 | Phenolic acids |
| 632 | Eriodictyol-3'-O-glucoside* | 7 | C21H22O11 | Flavonoids | 1314 | 1-O-Caffeoyl-3-O-galloyl-β-D-glucose | 10 | C22H22O13 | Phenolic acids |
| 633 | 3',5,5',7-Tetrahydroxyflavanone-7-O-glucoside* | 7 | C21H22O11 | Flavonoids | 1315 | 3,5-Dihydroxy-4-methoxybenzoic acid; 4-O-Methylgallic Acid | 10 | C8H8O5 | Phenolic acids |
| 634 | Eriodictyol-7-O-glucoronide | 7 | C21H20O12 | Flavonoids | 1316 | 3-O-Galloyl-Glucose | 10 | C13H16O10 | Phenolic acids |
| 635 | Dihydrokaempferide | 7 | C16H14O6 | Flavonoids | 1317 | 1,6-Di-O-Galloyl-D-Glucose* | 10 | C20H20O14 | Phenolic acids |
| 636 | Dihydrokaempferol-3-O-glucoside* | 7 | C21H22O11 | Flavonoids | 1318 | 4-hydroxy-3-methoxyphenyl 1-O-β-D-(6'-O-galloyol)-glucopyranoside* | 10 | C20H22O12 | Phenolic acids |
| 637 | Luteolin (5,7,3',4'-Tetrahydroxyflavone) | 7 | C15H10O6 | Flavonoids | 1319 | Grevilloside G* | 10 | C14H20O8 | Phenolic acids |
| 638 | 6,7,8-Tetrahydroxy-5-methoxyflavone* | 7 | C16H12O6 | Flavonoids | 1320 | 3,4-Dihydroxyphenethyl alcohol-8-O-[β-D-apinosyl(1→3)]-β-D-glucoside | 10 | C19H28O12 | Phenolic acids |
| 639 | Tetahydroxyflavone-7-O-glucuronide | 7 | C21H18O12 | Flavonoids | 1321 | β-Hydroxy-(3,4-dihydroxyphenylethanolyl)-glucoside | 10 | C14H20O9 | Phenolic acids |
| 640 | Apigenin-6-C-fucoside | 7 | C21H20O9 | Flavonoids | 1322 | 4-Hydroxyphenyl 6-O-(2-Methyl-3-Hydroxypropionyl)-Beta-D-Galactopyranoside | 10 | C16H22O9 | Phenolic acids |
| 641 | Diosmetin (5,7,3'-Trihydroxy-4'-methoxyflavone)* | 7 | C16H12O6 | Flavonoids | 1323 | 2-O-(4-Hydroxybenzoyl)-6-O-(Galloyl)-Beta-D-Glucopyranose | 10 | C20H20O12 | Phenolic acids |
| 642 | 6-Hydroxyluteolin 5-glucoside* | 7 | C21H20O12 | Flavonoids | 1324 | 2-O-Salicyl-6-O-Galloyl-D-Glucose | 10 | C20H20O12 | Phenolic acids |
| 643 | 5,7,2'-Trihydroxy-8-methoxyflavone* | 7 | C16H12O6 | Flavonoids | 1325 | Cimidahurinine* | 10 | C14H20O8 | Phenolic acids |
| 644 | Apigenin-7-O-(6''-acetyl)glucoside | 7 | C23H22O11 | Flavonoids | 1326 | 1-O-Galloyl-6-O-Feruloyl-β-D-glucose* | 10 | C23H24O13 | Phenolic acids |
| 645 | Dihydroxy-dimethoxyflavone-7-O-glucoside* | 7 | C23H24O11 | Flavonoids | 1327 | 2,4,6-Tri-O-galloyl-D-glucose* | 10 | C27H24O18 | Phenolic acids |
| 646 | Tricin-4'-O-glucoside* | 7 | C23H24O12 | Flavonoids | 1328 | 1,4,6-Tri-O-galloyl-β-D-glucose* | 10 | C27H24O18 | Phenolic acids |
| 647 | Acacetin-7-O-glucuronide | 7 | C26H20O8 | Flavonoids | 1329 | Methyl 4,6-di-O-galloyl-D-glucoside | 10 | C21H22O14 | Phenolic acids |
| 648 | 5,7,3',4'-Tetrahydroxy-6-methoxyflavone-8-C-[glucosyl-(1-2)]-glucoside | 7 | C28H32O17 | Flavonoids | 1330 | Monogalloyl-diglucose | 10 | C19H26O15 | Phenolic acids |
| 649 | Galangin (3,5,7-Trihydroxyflavone) | 7 | C15H10O5 | Flavonoids | 1331 | 1-O-Galloyl-3-O-p-Coumaroyl-β-D-glucose* | 10 | C22H22O12 | Phenolic acids |
| 650 | Luteolin-7-O-glucuronide-5-O-rhamnoside | 7 | C27H28O16 | Flavonoids | 1332 | 1-O-Galloyl-2-O-p-Coumaroyl-β-D-glucose* | 10 | C22H22O12 | Phenolic acids |
| 651 | Apigenin-6-C-(2''-glucuronyl)glucoside | 7 | C27H28O16 | Flavonoids | 1333 | 3-O-Methylgallic acid | 10 | C8H8O5 | Phenolic acids |
| 652 | Apigenin-6-C-arabinoside-8-C-xyloside* | 7 | C25H26O13 | Flavonoids | 1334 | 1-O-Galloyl-2-O-Feruloyl-β-D-glucose* | 10 | C23H24O13 | Phenolic acids |
| 653 | Apigenin-6,8-di-C-arabinoside* | 7 | C25H26O13 | Flavonoids | 1335 | 1-O-Caffeoyl-4-O-galloyl-β-D-glucose | 10 | C22H22O13 | Phenolic acids |
| 654 | Vitexin-7-O-(6''-p-coumaroyl)glucoside | 7 | C36H36O17 | Flavonoids | 1336 | Coniferyl alcohol* | 10 | C10H12O3 | Phenolic acids |
| 655 | Isovitexin-2''-O-(6'''-feruloyl)glucoside | 7 | C37H38O18 | Flavonoids | 1337 | 1-Feruloyl-sn-glycerol* | 10 | C13H16O6 | Phenolic acids |
| 656 | Chrysoeriol-8-C-arabinoside-7-O-Sophoroside | 7 | C33H40O20 | Flavonoids | 1338 | 1,6-Di-O-galloyl-β-D-glucose | 10 | C20H20O14 | Phenolic acids |
| 657 | Luteolin-7-O-glucoside (Cynaroside)* | 7 | C21H20O11 | Flavonoids | 1339 | Digalloylglucose | 10 | C20H20O14 | Phenolic acids |
| 658 | Luteolin-6-C-arabinoside-7-O-glucoside | 7 | C26H28O15 | Flavonoids | 1340 | Lithospermic acid B* | 10 | C36H30O16 | Phenolic acids |
| 659 | Kaempferol-3-O-glucuronide | 7 | C21H18O12 | Flavonoids | 1341 | Salvianolic acid L* | 10 | C36H30O16 | Phenolic acids |
| 660 | Kaempferol-3-O-(6''-O-acetyl)glucoside | 7 | C23H22O12 | Flavonoids | 1342 | 1-O-Galloyl-4-O-Feruloyl-β-D-glucose* | 10 | C23H24O13 | Phenolic acids |
| 661 | Robinetin | 7 | C15H10O7 | Flavonoids | 1343 | galloyl acetyl glucoside | 10 | C15H18O11 | Phenolic acids |
| 662 | Quercetin 7-O-p-coumaroyl rhamnoside* | 7 | C30H26O13 | Flavonoids | 1344 | 1-O-Galloyl-4-O-p-Coumaroyl-β-D-glucose* | 10 | C22H22O12 | Phenolic acids |
| 663 | Quercetin 5-O-p-coumaroyl rhamnoside* | 7 | C30H26O13 | Flavonoids | 1345 | 1,3,4,6-Tetra-O-Galloyl-D-Glucose | 10 | C34H28O22 | Phenolic acids |
| 664 | Quercetin-4'-O-glucoside (Spiraeoside)* | 7 | C21H20O12 | Flavonoids | 1346 | 2,3-di-O-galloyl--d-glucose* | 10 | C20H20O14 | Phenolic acids |
| 665 | Quercetin-3-O-galactoside (Hyperin) | 7 | C21H20O12 | Flavonoids | 1347 | Syringic acid | 10 | C9H10O5 | Phenolic acids |
| 666 | Ikarisoside D | 7 | C28H30O11 | Flavonoids | 1348 | Protocatechuic acid 4-O-(6''-O-Galloy)Glucoside | 10 | C20H20O13 | Phenolic acids |
| 667 | 6-Hydroxykaempferol-7-O-glucoside | 7 | C21H20O12 | Flavonoids | 1349 | 6-O-Galloyl-1-O-vanilloyl-β-D-glucose | 10 | C21H22O13 | Phenolic acids |
| 668 | Quercetin-7-O-glucoside* | 7 | C21H20O12 | Flavonoids | 1350 | 1,2,6-Tri-O-galloyl-β-D-glucose* | 10 | C27H24O18 | Phenolic acids |
| 669 | 3-Methylkaempferol | 7 | C16H12O6 | Flavonoids | 1351 | 1,4-Di-O-Galloyl-D-glucose | 10 | C20H20O14 | Phenolic acids |
| 670 | Isorhamnetin 3-galactoside | 7 | C22H22O12 | Flavonoids | 1352 | 3,4-Digalloylshikimic acid | 10 | C21H18O13 | Phenolic acids |
| 671 | 6-C-Methylquercetin-3-O-rhamnoside | 7 | C22H22O11 | Flavonoids | 1353 | 4-Methoxyphenylpropionic acid* | 10 | C10H12O3 | Phenolic acids |
| 672 | 3,5,4'-Trihydroxy-7-methoxyflavone (Rhamnocitrin)* | 7 | C16H12O6 | Flavonoids | 1354 | 1,3,6-Tri-O-galloyl-β-D-glucose* | 10 | C27H24O18 | Phenolic acids |
| 673 | Quercetin-3-O-glucoside (Isoquercitrin) | 7 | C21H20O12 | Flavonoids | 1355 | 3,4,5-Trimethoxyphenol-1-O-β-D-glucoside | 10 | C15H22O9 | Phenolic acids |
| 674 | Quercetin-5-O-β-D-glucoside | 7 | C21H20O12 | Flavonoids | 1356 | 1-(3,4-dihydroxyphenyl)-7-(4-hydroxy-3-methoxyphenyl)hepta-1,6-diene-3,5-dione | 10 | C20H18O6 | Phenolic acids |
| 675 | Phellatin | 7 | C26H30O12 | Flavonoids | 1357 | Antiarol; 3,4,5-Trimethoxyphenol | 10 | C9H12O4 | Phenolic acids |
| 676 | Quercetin-3,7-Di-O-glucoside* | 7 | C27H30O17 | Flavonoids | 1358 | Dicaffeoylshikimic acid | 10 | C25H22O11 | Phenolic acids |
| 677 | Myricetin-3-O-rhamnoside (Myricitrin) | 7 | C21H20O12 | Flavonoids | 1359 | Galloylprocyanidin B4 | 10 | C37H30O16 | Tannins |
| 678 | Myricetin-3-O-arabinoside | 7 | C20H18O12 | Flavonoids | 1360 | Theaflavin | 10 | C29H24O12 | Tannins |
| 679 | Hesperetin-6-C-glucoside-7-O-glucoside | 7 | C28H34O16 | Flavonoids | 1361 | Galloylprocyanidin C2 | 10 | C52H42O22 | Tannins |
| 680 | Quercetin-3-O-glucoside-7-O-rhamnoside* | 7 | C27H30O16 | Flavonoids | 1362 | Procyanidin C1 3'-O-gallate | 10 | C52H42O22 | Tannins |
| 681 | Quercetin-7-O-rutinoside* | 7 | C27H30O16 | Flavonoids | 1363 | 3,3',4-O-Trimethylellagic acid | 10 | C17H12O8 | Tannins |
| 682 | 6-Hydroxykaempferol-7,6-O-Diglucoside | 7 | C27H30O17 | Flavonoids | 1364 | 3,4,5,3',4',5'-Hexahydroxydiphenoyl Acid-glucose | 10 | C20H18O14 | Tannins |

**Supplementary Table S4.** Quality of the transcriptomes of different tissues of *Machilus nanmu*.

| Sample | Raw Reads | Clean Reads | Clean Base(G) | Error Rate(%) | Q20(%) | Q30(%) | GC Content(%) |
| --- | --- | --- | --- | --- | --- | --- | --- |
| L-1 | 61765630 | 60073684 | 9.01 | 0.03 | 98.18 | 94.59 | 47.14 |
| L-2 | 48389686 | 46900470 | 7.04 | 0.02 | 98.23 | 94.67 | 47.02 |
| L-3 | 46710012 | 45582862 | 6.84 | 0.02 | 98.2 | 94.63 | 46.9 |
| R-1 | 65376890 | 63435134 | 9.52 | 0.03 | 98.06 | 94.28 | 45.23 |
| R-2 | 64917156 | 63051076 | 9.46 | 0.03 | 98.01 | 94.18 | 45.3 |
| R-3 | 67434510 | 65411388 | 9.81 | 0.03 | 97.99 | 94.14 | 45.15 |
| S-1 | 56396922 | 54456694 | 8.17 | 0.03 | 98.15 | 94.52 | 45.63 |
| S-2 | 54291706 | 52496178 | 7.87 | 0.03 | 98.1 | 94.41 | 45.68 |
| S-3 | 53224050 | 51624940 | 7.74 | 0.03 | 98.17 | 94.53 | 45.7 |

**Supplementary Table S5.** Assembly result of the transcriptomes of *Machilus nanmu*.

| Type | Number | Mean Length | N50 | N90 | Total Bases |
| --- | --- | --- | --- | --- | --- |
| Transcript | 319687 | 1001 | 1751 | 386 | 320140114 |
| Unigene | 168717 | 1321 | 1981 | 575 | 222878599 |

**Supplementary Table S6.** Annotation Statistics

| Database | Number of Genes | Percentage (%) |
| --- | --- | --- |
| KEGG | 71313 | 42.27 |
| Nr | 98130 | 58.16 |
| SwissProt | 66303 | 39.3 |
| TrEMBL | 99381 | 58.9 |
| KOG | 57048 | 33.81 |
| GO | 86000 | 50.97 |
| Pfam | 67499 | 40.01 |
| Annotated in at least one Database | 104186 | 61.75 |
| Total Unigenes | 168717 | 100 |

**Supplementary Table S7.** The information of 41 encoded genes.

| NO. | Gene | R-1 | R-2 | R-3 | S-1 | S-2 | S-3 | L-1 | L-2 | L-3 | Enzyme |
| --- | --- | --- | --- | --- | --- | --- | --- | --- | --- | --- | --- |
| 1 | Cluster-19489 | 6.89 | 2.8 | 2.63 | 0 | 0 | 0 | 0 | 0 | 0 | 4CL |
| 2 | Cluster-38926 | 7.93 | 2.23 | 6.43 | 16.51 | 6.29 | 8.61 | 0.82 | 1.04 | 0.18 | 4CL |
| 3 | Cluster-61524 | 28.18 | 22.06 | 18.67 | 36.05 | 23.27 | 25.04 | 5.09 | 3.08 | 8.65 | 4CL |
| 4 | Cluster-67559 | 24.9 | 21.95 | 21.84 | 23.81 | 26.01 | 29.8 | 21.1 | 27.05 | 33.43 | 4CL |
| 5 | Cluster-76735 | 164.27 | 200 | 138.63 | 116.71 | 151.48 | 108.7 | 6.17 | 4.72 | 5.92 | 4CL |
| 6 | Cluster-85842 | 13.93 | 11.39 | 7.94 | 20.87 | 17.01 | 12.65 | 4.71 | 4.02 | 2.3 | 4CL |
| 7 | Cluster-8699 | 5.38 | 0.95 | 2.04 | 0 | 0 | 0 | 0 | 0 | 0 | 4CL |
| 8 | Cluster-90712 | 28.6 | 37.79 | 27.29 | 45.75 | 57.97 | 68.3 | 7.04 | 5.84 | 10.13 | 4CL |
| 9 | Cluster-99983 | 12.31 | 20.32 | 14.59 | 17.6 | 23.6 | 26.93 | 2.69 | 2.6 | 3.54 | ANR |
| 10 | Cluster-75571 | 392.17 | 515.7 | 433.46 | 539.21 | 711.55 | 894.8 | 119 | 109.8 | 182.5 | ANS |
| 11 | Cluster-66706 | 15.73 | 14.71 | 15.44 | 50.1 | 41.97 | 54.61 | 29.1 | 22.58 | 27.98 | BZ1 |
| 12 | Cluster-73806 | 2.42 | 2.32 | 1.85 | 2.97 | 1.73 | 2.22 | 1.81 | 2.06 | 3.05 | BZ1 |
| 13 | Cluster-89239 | 313.48 | 368.1 | 407.69 | 426.95 | 520.42 | 658.1 | 130 | 118.8 | 137.1 | CHI |
| 14 | Cluster-71750 | 453.98 | 695 | 529.93 | 600.14 | 766.64 | 922.4 | 178 | 192.3 | 276.6 | CHS |
| 15 | Cluster-74929 | 541.04 | 657.2 | 730.47 | 404.09 | 675.82 | 1020 | 45.4 | 31.02 | 81.12 | CHS |
| 16 | Cluster-44193 | 3.05 | 1.24 | 1.26 | 3.36 | 2.47 | 2.28 | 4.36 | 2.94 | 3.67 | CYP71D9 |
| 17 | Cluster-86197 | 0.27 | 0 | 0.08 | 4.93 | 5.89 | 9.09 | 1.05 | 0.89 | 2.57 | CYP71D9 |
| 18 | Cluster-21503 | 6.38 | 2.51 | 2.4 | 0 | 0 | 0 | 0 | 0 | 0 | CYP93C |
| 19 | Cluster-86814 | 252.59 | 323.3 | 366.68 | 304.2 | 390.1 | 489.1 | 75.6 | 77.23 | 97.61 | DFR |
| 20 | Cluster-101131 | 0 | 0 | 0.18 | 27.86 | 7.62 | 35.59 | 0.19 | 0 | 0.51 | F3'5'H |
| 21 | Cluster-90905 | 41.51 | 59.45 | 43.18 | 61.33 | 93.38 | 100.6 | 59.3 | 69.55 | 62.75 | F3H |
| 22 | Cluster-92931 | 30.32 | 50.72 | 61.09 | 33.32 | 58.8 | 82.68 | 10.7 | 11.28 | 20.74 | F3H |
| 23 | Cluster-93340 | 143.93 | 208.3 | 167.82 | 219.91 | 262.02 | 314.4 | 38.4 | 41.39 | 42.5 | F3'H |
| 24 | Cluster-96821 | 2.54 | 3.97 | 1.84 | 18.46 | 22.67 | 23.55 | 1.65 | 1.1 | 2.54 | FG2 |
| 25 | Cluster-65392 | 1.49 | 1.5 | 1.74 | 1.56 | 1.04 | 0.91 | 0.21 | 0.21 | 0.14 | FLS |
| 26 | Cluster-74649 | 0 | 0 | 0 | 2.34 | 1.95 | 1.11 | 74.4 | 35.22 | 26.4 | FLS |
| 27 | Cluster-82106 | 2.43 | 1 | 1.17 | 4.92 | 4.09 | 3.82 | 9.47 | 8.52 | 6.4 | FLS |
| 28 | Cluster-71358 | 11.9 | 16.7 | 9.55 | 9.7 | 10.38 | 13.13 | 1.88 | 1.09 | 2.56 | FLS |
| 29 | Cluster-41824 | 56.45 | 48.91 | 34.18 | 7.75 | 5.8 | 4.2 | 17.8 | 12.79 | 18.61 | I2'H |
| 30 | Cluster-97008 | 113.5 | 134.9 | 130.2 | 147.72 | 149.54 | 157.2 | 210 | 226.3 | 258.3 | LAR |
| 31 | Cluster-99888 | 76.58 | 89.72 | 85.03 | 57.27 | 73.47 | 85.75 | 7.66 | 3.85 | 6.53 | LAR |
| 32 | Cluster-76990 | 157.03 | 262.3 | 208.02 | 160.98 | 276.97 | 287.4 | 27.5 | 15.93 | 34.3 | PAL |
| 33 | Cluster-95188 | 39.78 | 59.2 | 62.6 | 40.97 | 59.09 | 90.99 | 8.63 | 5.68 | 15.57 | PAL |
| 34 | Cluster-97647 | 28.67 | 16.48 | 12.78 | 7.72 | 8.02 | 7.26 | 10.4 | 9.39 | 9.97 | PAL |
| 35 | Cluster-52077 | 9.6 | 1.55 | 5.89 | 5.07 | 5.95 | 5.52 | 9.03 | 21.01 | 16.05 | UGT75C1 |
| 36 | Cluster-101448 | 10.12 | 9.23 | 7.3 | 20.25 | 13.58 | 23.47 | 2.28 | 1.34 | 1.87 | PGT1 |
| 37 | Cluster-69292 | 10.57 | 11.25 | 6.17 | 6.18 | 2.75 | 2.32 | 20.7 | 13.75 | 13.33 | PGT1 |
| 38 | Cluster-71935 | 100.53 | 64.1 | 66.54 | 62.57 | 59.78 | 54.21 | 110 | 112.4 | 121.3 | PGT1 |
| 39 | Cluster-91635 | 0.41 | 1.94 | 2.96 | 4.62 | 1.71 | 0 | 59.6 | 41.91 | 39.55 | PGT1 |
| 40 | Cluster-94732 | 18.85 | 9.75 | 6.13 | 9.52 | 21.26 | 6.41 | 9.05 | 29.53 | 8.11 | PGT1 |
| 41 | Cluster-98790 | 2.04 | 2.68 | 3.14 | 2.02 | 1.77 | 1.44 | 0.18 | 0.45 | 0.43 | PGT1 |

**Supplementary Table S8.** Structural genes and transcription factors screened out by WGCNA.

| Structural genes | Transcription factors |
| --- | --- |
| Cluster-66706 (BZ1) | Cluster-80252 (MYB) |
| Cluster-61524 (4CL) | Cluster-89108 (MYB) |
| Cluster-67559 (4CL) | Cluster-99927 (bHLH) |
| Cluster-85842 (4CL) | Cluster-61136 (MYB) |
| Cluster-72851 (CHI) | Cluster-100521 (bHLH) |
| Cluster-63826 (CHS) | Cluster-92801 (bHLH) |
| Cluster-52077 (UTG75C1) |  |
| Cluster-82106 (F3H) |  |
| Cluster-41824 (I2'H) |  |
| Cluster-97008 (LAR) |  |
| Cluster-69292 (PTG1) |  |
| Cluster-71935 (PTG1) |  |
| Cluster-91635 (PTG1) |  |
